# Mapping gene regulatory circuitry of Pax6 during neurogenesis

**DOI:** 10.1038/celldisc.2015.45

**Published:** 2016-02-09

**Authors:** Sudhir Thakurela, Neha Tiwari, Sandra Schick, Angela Garding, Robert Ivanek, Benedikt Berninger, Vijay K Tiwari

**Affiliations:** 1 Institute of Molecular Biology (IMB), Ackermannweg 4, Mainz, Germany; 2 Institute of Physiological Chemistry, University Medical Center of the Johannes Gutenberg University Mainz, Hanns-Dieter-Hüsch-Weg 19, Mainz, Germany; 3 Department of Biomedicine, University of Basel, Basel, Switzerland; 4 Focus Program Translational Neuroscience, Johannes Gutenberg University Mainz, Langenbeckstr. 1, Mainz, Germany

**Keywords:** Chromatin, Gene regulation, Neural progenitors, Neurogenesis, Transcription Factors

## Abstract

Pax6 is a highly conserved transcription factor among vertebrates and is important in various aspects of the central nervous system development. However, the gene regulatory circuitry of Pax6 underlying these functions remains elusive. We find that Pax6 targets a large number of promoters in neural progenitors cells. Intriguingly, many of these sites are also bound by another progenitor factor, Sox2, which cooperates with Pax6 in gene regulation. A combinatorial analysis of Pax6-binding data set with transcriptome changes in Pax6-deficient neural progenitors reveals a dual role for Pax6, in which it activates the neuronal (ectodermal) genes while concurrently represses the mesodermal and endodermal genes, thereby ensuring the unidirectionality of lineage commitment towards neuronal differentiation. Furthermore, Pax6 is critical for inducing activity of transcription factors that elicit neurogenesis and repress others that promote non-neuronal lineages. In addition to many established downstream effectors, Pax6 directly binds and activates a number of genes that are specifically expressed in neural progenitors but have not been previously implicated in neurogenesis. The *in utero* knockdown of one such gene, *Ift74*, during brain development impairs polarity and migration of newborn neurons. These findings demonstrate new aspects of the gene regulatory circuitry of Pax6, revealing how it functions to control neuronal development at multiple levels to ensure unidirectionality and proper execution of the neurogenic program.

## Introduction

The paired box protein, Pax6, is a highly conserved transcription factor of 422 amino acids comprising two DNA-binding domains, an amino-terminal paired domain and a homeodomain along with a carboxy-terminal proline/serine/threonine-rich transactivation domain [1, 2]. Pax6 was first discovered to be required for proper segmentation in *Drosophila* [3, 4] and later shown to be essential for eye development in *Drosophila* [5], a role that was further found to be conserved in human and mouse eye development [6, 7]. During mammalian brain development, Pax6 is expressed in a specific spatiotemporal manner and is restricted to mainly neuronal tissues [2, 8]. Pax6 is now established to be essential for maintaining the pool of neural stem cells (NSCs) and thereby regulating embryonic as well as adult neurogenesis, as shown by its expression in neuroepithelial and radial glial cells, which can divide symmetrically to produce NSCs or asymmetrically to become a NSC and a neuron [9, 10].

The discovery of a plethora of known Pax6 functions has been facilitated by various Pax6 mutants. One such very useful mutant, the small eye (*Sey*) mouse mutant, contains a single-base substitution [11], resulting in the production of a functionally inactive and truncated Pax6 lacking the DNA-binding homeodomain and the C-terminal activation domain. Importantly, the Sey mutant mouse phenotype is similar to that of an artificially targeted *Pax6*-deficient mouse (*Pax6*^−ax^), showing small eyes and numerous neural defects, including reduced neurons in the cerebral cortex [11–13]. These phenotypic similarities in the Sey mutant and *Pax6*^−ax^ substantiate the use of *Sey* homozygous mutant mice as *Pax6*-null mutants. It was further shown that Sey mutant embryonic stem (ES) cells generate misspecified neurons that undergo death because of high expression of the neurotrophin receptor p75NTR [14].

It is well established that Pax6 is crucial for the development of the central nervous system, eyes, nose, pancreas and pituitary gland [13, 15, 16]. Recent studies have shown that Pax6 functions upstream of gene networks involved in brain patterning, neuronal migration and neural circuit formation [17]. Despite the established role of Pax6 in neurogenesis, its genomic targets, their chromatin status and its cooperativity with other transcription factors during neurogenesis remain unclear. Furthermore, while a number of players functioning downstream of Pax6 have been identified, these are not enough to explain the plethora of functions Pax6 is known for. Here we reveal that in neural progenitors, Pax6 binds a large number of gene promoters that exhibit epigenetic state that is hallmark of open chromatin. Many Pax6-bound promoters are also targeted by Sox2 and functionally cooperate in gene regulation underlying neuronal specification. Pax6 directly binds and silences genes important for mesoderm and endoderm development as they get de-repressed in progenitors lacking Pax6. In addition, Pax6 targets that are downregulated in mutant progenitors are known to be critically involved in neuronal development. Pax6-driven gene-expression program further induces activity of neurogenic transcription factors and repress others that promote non-neuronal lineages. Importantly, our analysis also revealed a number of Pax6-induced genes that are highly expressed during brain development but their function has not yet been explored during neurogenesis. Here we show that one such gene, *Ift74*, which is directly bound and activated by Pax6 in NP cells, is required for the proper migration of newborn neurons. Furthermore, our analysis revealed that Pax6 directly targets the promoter of Notch signaling components and induces their expression, which then further contribute to Ift74 expression. These observations reveal the manner by which Pax6 controls multiple components of the network underlying neuronal development and uncovers Ift74 as a novel regulator of neurogenesis.

## Results

### Pax6 binds to a number of gene promoters in neural progenitors cells

We first determined the expression patterns of Pax6 in various embryonic tissues and cortical layers. As shown previously, Pax6 is specifically highly expressed in ventricular zone (VZ) and is gradually lost as cells progress through the subventricular zone (SVZ) to the cortical plate (CP; Supplementary Figure S1A). An analysis of other ectoderm (epidermis), mesoderm (heart and mouse embryonic fibroblasts) and endoderm tissues (lung and pancreas) showed relative absence of Pax6 expression, with the exception of the pancreas that exhibited low levels of Pax6, confirming previous reports [18] (Supplementary Figure S1A). We next use a highly refined and established differentiation model of neurogenesis, in which mouse ES cells first differentiate into Pax6-positive NP (radial glial-like) cells (also referred as celllular aggregates, in short CA_D8) and subsequently into terminally differentiated glutamatergic pyramidal neurons (TN) with high purity (>95%) and synchrony and is known to closely recapitulate the stages of embryonic neurogenesis [19–21]. The expression analysis of Pax6 in this system revealed its highest expression in cellular aggregate cells, thereby presenting a system for investigating Pax6 function *in vitro* (Supplementary Figure S1B).

**Figure 1:**
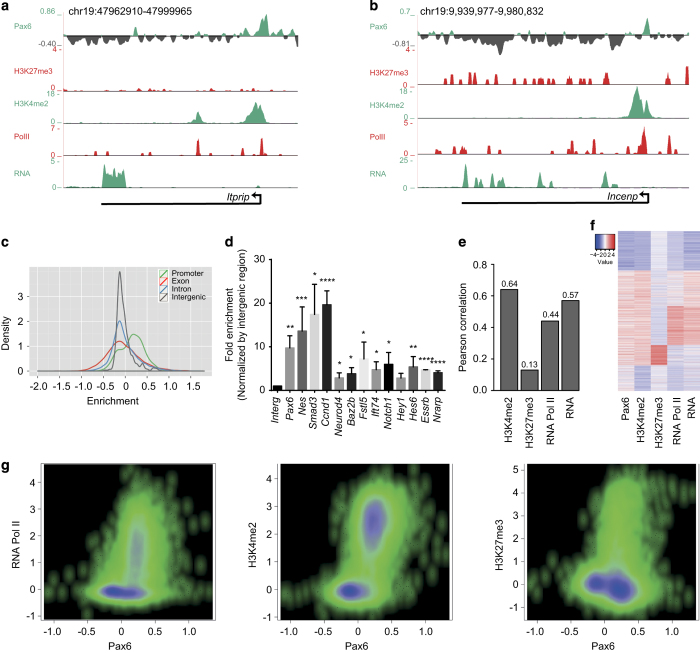
Pax6 targets many gene promoters that largely exhibit an active chromatin state. (**a**, **b**) Representative examples of genome browser tracks showing specific occupancies of Pax6 at promoter and its relative de-enrichment at other regions of a selected gene (*Itprip*-A*, Incenp-*B). UCSC browser tracks from ChIP-seq datasets for H3K4me2, H3K27me3 and RNA Pol II as well as RNA-seq from neural progenitor cells are also shown. (**c**) Density plot showing genome-wide enrichment of Pax6 at promoters compared with other genomic regions on chromosome 19. To calculate the relative enrichment for each region, Pax6 enrichment was normalized with respect to the total size of that region. (**d**) ChIP-quantitative PCR validations of selected target genes showing the enrichment of Pax6 at their promoters. Pax6 and Nes are used as a positive control while intergenic region is a non-target (negative) control. Error bars reflect s.d. Statistical significance were calculated with an unpaired t-test (*P<0.05; **P<0.01; ***P<0.001; ****P<0.0001). (**e**) Bar plot showing Pearson correlation coefficients between Pax6 occupancy and H3K4me2, H3K27me3 and RNA Poll II RNA levels. (**f**) Heat map showing a comparison of Pax6 occupancy with H3K4me2, H3K27me3 and RNA Poll II RNA levels. Red indicates high values and blue low values. (**g**) Scatter plot comparing enrichment of Pax6 occupancy at promoters with enrichments of RNA Pol II, H3K4me2 and H3K27me3 in the same *in vitro* differentiation system. Each dot represents a promoter and *x* axis shows the enrichment of Pax6, while *y* axis represents enrichment of RNA Pol II, H3K4me2 or H3K27me3. Higher density of data points is represented as dark blue, while relatively less density is shown as light green.

To shed light on Pax6 function, we performed Pax6 chromatin immunoprecipitation (ChIP) in NPs and investigated its genome-wide binding pattern using a previously described ChIP-chip platform in biological replicates [22]. These arrays cover 10% of the mouse genome, including all well-annotated promoters, several large multigene loci and the complete chromosome 19 [23]. The visual inspection of the genomic regions suggested that Pax6 is targeted to distinct genomic sites and also occupies a number of promoters (*n*=5086, promoter enrichment >0.25; Figure 1a and b, Supplementary Figure S1C and D, Supplementary Table S1). A comprehensive and unbiased analysis of Pax6 binding along the fully tiled chromosome 19 revealed its relatively high enrichment at promoters (Figure 1c). These observations were validated at selected gene promoters in independent ChIP assays (Figure 1d). Such targeting of Pax6 to gene promoters prompted us to investigate its relationship with the chromatin state of target sites and the transcriptional states of associated genes at the progenitor stage. We analyzed the ChIP-seq data sets for RNA Pol II, H3K4me2, which is an established active histone modification, and the Polycomb group repressive mark, H3K27me3, at the NP stage in the same differentiation system, and correlated these data with Pax6 occupancy at the target gene promoters (Figure 1e and f). Further analysis showed that Pax6 occupancy was most strongly correlated with the active mark H3K4me2 (*R*^2^=0.64; Figure 1e). In addition, a large number of Pax6-bound promoters were RNA Pol II bound (*R*^2^=0.44) and actively transcribed (*R*^2^=0.57; Figure 1e). Furthermore, Pax6 target promoters were mostly devoid of the repressive mark H3K27me3 (*R*^2^=0.13; Figure 1e). Heat map visualization at promoters supported these observations, revealing that the majority of Pax6-bound genes displayed the H3K4me2 mark, a significant fraction of which were Pol II bound and actively transcribed (Figure 1f). Furthermore, comparison of Pax6 occupancy at promoters with enrichment of RNA Pol II, H3K4me2 and H3K27me3 in the same differentiation system showed similar patterns (Figure 1g). A comparison with promoter targets of Pax6 recently identified by ChIP-seq assay in E12.5 forebrain tissue [24] showed that out of 240 promoter targets discovered in this study, 141 promoters were also detected as Pax6 targets in our study (data not shown), supporting the comprehensiveness of our data.

### Pax6 targets are misregulated in Pax6 mutant NPs

**Figure 2:**
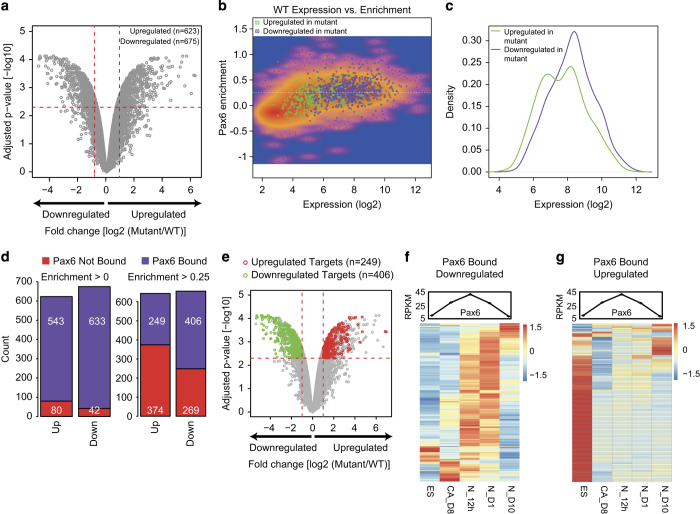
Pax6 targets are misregulated in the absence of *Pax6*. (**a**) Volcano plot showing changes in expression of genes in WT and *Pax6* mutant (mutant). *x* axis represents fold change in log2 scale between WT and mutant, and the *y* axis shows level of significance as in –log10 of adjusted *P*-value. Vertical dotted red lines reflect a two-fold cutoff for expression while horizontal dotted red lines represent a *P*-value cutoff of 0.005. (**b**) Scatter plot showing log2 of expression levels in WT (*x* axis) and Pax6 enrichment on *y* axis. Upregulated (green) and downregulated (purple) genes in *Pax6* mutant are highlighted as dots. (**c**) Density plot to show the WT expression of upregulated and downregulated genes shown in **b**. *y* axis represents the density while *x* axis represents the expression levels. (**d**) Stacked bar plots showing that the substantial subset of differentially regulated genes in Pax6 mutants are Pax6 targets (two different Pax6 promoter enrichment cutoff: >0 (low stringency, left panel), >0.25 (high stringency, right panel). (**e**) Volcano plot as described in **a** but highlighting only upregulated (red dots) and downregulated (green dots) genes that are Pax6 targets in WT cells. (**f**, **g**) Heat maps showing the expression in ES, CA, N_12h (neurons at 12 h), N_D1 (day 1 neurons) and N_D10 (neurons at day 10) of Pax6-bound genes that are significantly downregulated (**f**) and upregulated (**g**) in Pax6 mutant progenitors. Line plots above the heat maps show expression of Pax6 in the same stages.

To further investigate the genes under the direct transcriptional control of Pax6, we differentiated Pax6 mutant ES cells (isolated from the blastocysts of homozygous *Sey* mutants, referred to thereafter as ‘mutant cells’) [14] into NPs and performed genome-wide transcriptome profiling. Sey mutant ES cells generate misspecified neurons that undergo death owing to high expression of the neurotrophin receptor p75NTR [14]. Comparing the transcriptome of Sey mutant cells with that of wild-type (WT) NP cells revealed 675 differentially downregulated and 623 differentially upregulated genes exhibiting enrichment for the nervous system development and metabolism related gene ontologies (GO), respectively (Figure 2a, Supplementary Figure S2A and B and Supplementary Table S2). Promoters of most of the genes downregulated in mutant progenitors were bound by Pax6 and very highly expressed in the WT progenitors, suggesting their robust transcription in the presence of Pax6 (Figure 2b and c). By directly comparing Pax6 binding at promoters to the transcriptional changes in mutant progenitors, we found that nearly all differentially expressed gene promoters (90%) were Pax6 bound (promoter enrichment >0) in the WT cells (Figure 2d, left bar plots; Supplementary Figure S2C) (hypergeometric *P*-value, upregulated genes: 1.71e-265; downregulated genes: 0). To retain only those target promoters that were highly bound by Pax6, we increased the cutoff to a higher level (promoter enrichment >0.25) for Pax6 enrichment, which revealed 406 downregulated and 249 upregulated genes (Figure 2d and e; hypergeometric *P*-value, upregulated genes: 1.70e-22; downregulated genes: 1.05e-99). The observation that a large fraction of genes downregulated in the mutant progenitors were highly enriched for Pax6 in the WT cells (*n*=406) is consistent with our previous observation that the majority of Pax6 targets were highly expressed in the WT NPs (Figure 2b and c). Interestingly, during the differentiation of ES cells into neurons, a large number of Pax6-bound/mutant-downregulated and Pax6-bound/mutant-upregulated genes were either majorly expressed and repressed in the NPs or an early neurogenesis stage, respectively (Figure 2f and g). In summary, Pax6 directly binds at the regulatory elements of many genes to govern their proper transcriptional dynamics during neuronal development.

### Pax6 activates neuronal development genes and represses genes from other lineages

**Figure 3:**
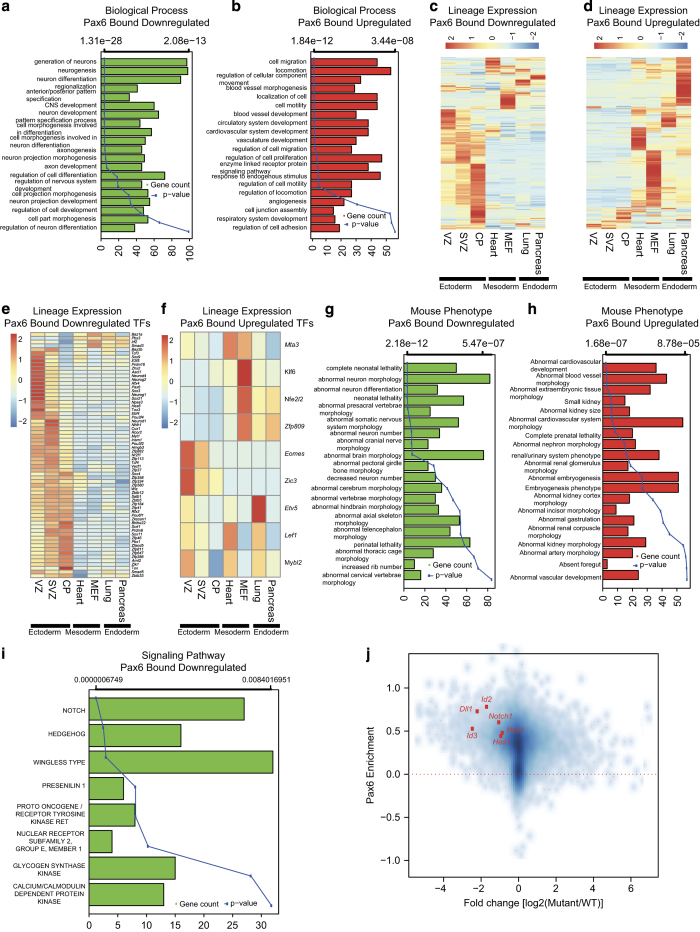
Pax6 activates neuronal genes while repressing mesodermal and endodermal genes. (**a**, **b**) Bar and line plots showing GO term enrichment analysis of Pax6-bound genes that were downregulated (**a**) or upregulated (**b**) in *Pax6* mutant progenitors. Bar plots show number of genes for each enriched GO term (main *x* axis), and lines represent *P*-values for corresponding GO terms (alternate *x* axis). (**c**, **d**) Expression of upregulated (**c**) and downregulated (**d**) genes in tissues from different germ layers. (**e**, **f**) Same as in **c** and **d** but only for differentially expressed transcription factors. (**g**, **h**) Same as in **a** and **b** but an enrichment analysis was performed for mouse phenotypes enriched in Pax6-bound downregulated (**g**) and upregulated (**h**) genes. (**i**) Similar bar plot as in **a**, but the enrichment analysis was performed for signaling pathways using Genomatix. (**j**) Scatter plot showing changes in expression of core Notch signaling pathway components in WT and *Pax6* mutant cells and their enrichments for Pax6. The *x* axis represents the fold change (log2) between WT and *Pax6* mutant cells, and the *y* axis shows Pax6 enrichment.

We next performed a GO enrichment analysis for genes that are Pax6-bound (promoter enrichment >0.25) and differentially expressed between the WT and mutant NPs. Genes bound by Pax6 and downregulated in mutant cells were exclusively enriched for neuronal development (Figure 3a). Interestingly, genes bound by Pax6 and upregulated in mutant progenitors showed enrichment for terms related to mesoderm (cardiovascular system development) and endoderm (for example, respiratory system development) development (Figure 3b). Considering these GO term enrichments, we further analyzed expression of these Pax6-bound differentially expressed genes in representative embryonic tissues from the three lineages. A majority of downregulated genes were expressed in the three layers of the embryonic cortex (VZ, SVZ and CP) [25], while upregulated genes were much higher expressed in tissues from mesoderm (heart and mouse embryonic fibroblast) [26, 27] and endoderm (lung and pancreas) lineages [28, 29] (Figure 3c and d). Furthermore, Pax6-bound upregulated and downregulated transcription factor genes also showed similar patterns in different lineages, as well as during *in vitro* neurogenesis where downregulated factors are mainly expressed in NPs or an early neurogenesis stage while upregulated factors show high expression in other lineages and ES cells (Figure 3e and f and Supplementary Figure S3A). To further substantiate these observations, we performed enrichment analysis on bound and differentially expressed genes based on known phenotypes associated with these genes. The downregulated genes were significantly associated with phenotypes related to brain development (Figure 3g), while upregulated genes were linked to phenotypes related to abnormal development of various mesodermal or endodermal tissues (Figure 3h). This further substantiates our previous observations and also provides additional insights into how Pax6 contributes to the gene expression program underlying neurogenesis.

To further uncover other aspects of the Pax6-dependent regulatory network, we performed a signaling pathway enrichment analysis. Genes regulated by Notch signaling, a pathway that is established to be critical for self-renewal of NSCs, were most highly enriched among the Pax6-target mutant-downregulated genes [30–32] (Figure 3i and j). This was followed by the Hedgehog signaling pathway, which is also shown to be important for specification of NPs [33] (Figure 3i and Supplementary Figure S3B). By contrast, Pax6-target mutant-upregulated genes showed enrichment for FGF signaling, that has been shown to be involved in mesodermal and endodermal specification [34–36] (Supplementary Figure S3C and D). Although previously Pax6 has been indirectly implicated in the control of Notch pathway [10], our analysis revealed that Pax6 directly binds at the promoters of a large number of genes associated with Notch signaling. Furthermore, these genes were downregulated in mutant NPs, indicating that Pax6 has a direct role in the activation of Notch signaling in NP cells (Figure 3j). This targeting by Pax6 at Notch signaling components provides potential mechanism regarding how this master transcription factor acts at multiple levels to define progenitor identity and differentiation towards neurons. Overall, these analyses identify a dual role for Pax6, in which it mediates the activation of neuronal (ectodermal) genes while concurrently represses the mesodermal and endodermal genes.

### Pax6 influences transcription factor network to confer unidirectionality towards neuronal differentiation

**Figure 4:**
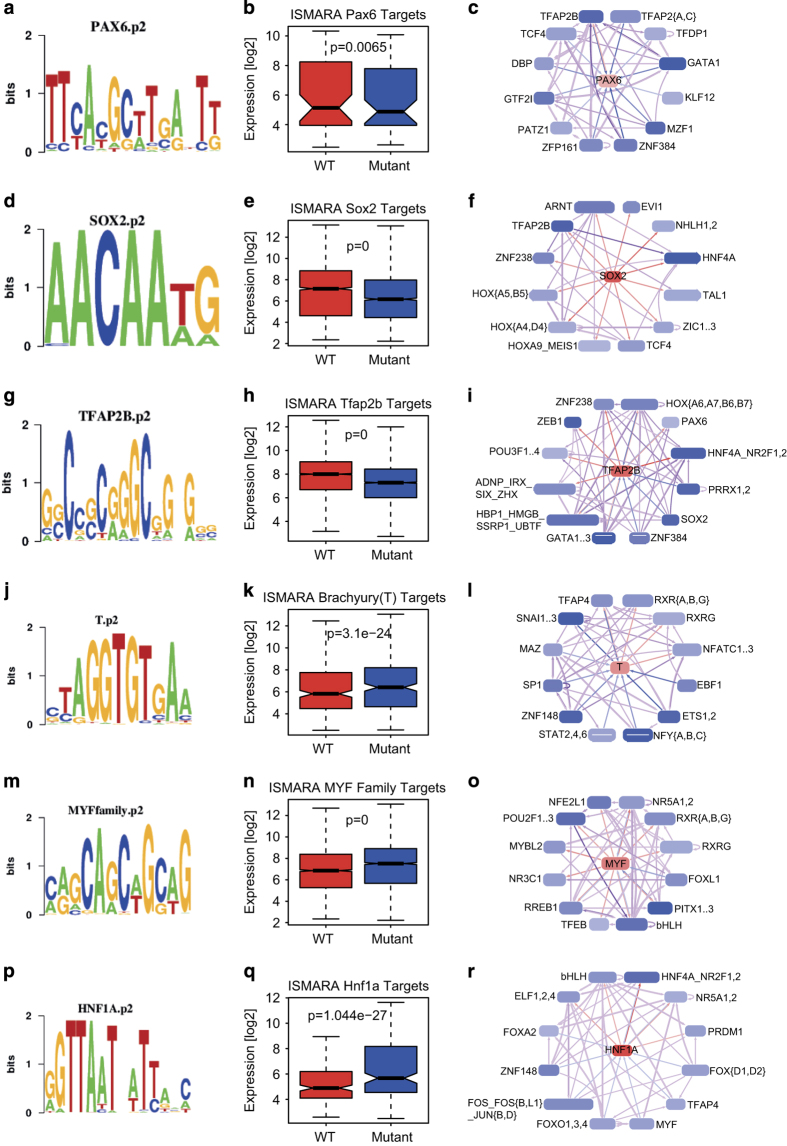
Pax6 is critical for inducing activity of transcription factors that elicit neurogenesis and repress others that promote non-neuronal lineages. (**a**) Pax6 motif identified by ISMARA. (**b**) Box plot showing expression in WT and mutant of Pax6 targets predicted by ISMARA. (**c**) First-level interaction network of Pax6 and its potential targets as predicted by ISMARA. (**d**) Sox2 motif identified by ISMARA. (**e**) Box plot showing expression in WT and mutant of Sox2 targets predicted by ISMARA. (**f**) First-level interaction network of Sox2 and its potential targets as predicted by ISMARA. (**g**) Tfap2b motif identified by ISMARA. (**h**) Box plot showing expression in WT and mutant of Tfap2b targets predicted by ISMARA. (**i**) First-level interaction network of Tfap2B and its potential targets as predicted by ISMARA. (**j**) Brachyury (T) motif identified by ISMARA. (**k**) Box plot showing expression in WT and mutant of Brachyury (T) targets predicted by ISMARA. (**l**) First-level interaction network of Brachyury (T) and its potential targets as predicted by ISMARA. (**m**) Myf family motif identified by ISMARA. (**n**) Box plot showing expression in WT and mutant of Myf family targets predicted by ISMARA. (**o**) First-level interaction network of Myf family and its potential targets as predicted by ISMARA. (**p**) Hnf1a motif identified by ISMARA. (**q**) Box plot showing expression in WT and mutant of Hnf1a targets predicted by ISMARA. (**r**) First-level interaction network of Hnf1a and its potential targets as predicted by ISMARA. All *P*-values are calculated using Wilcoxon test.

On the basis of our observations that Pax6 mutant cells showed upregulation of non-neuronal and downregulation of neuronal genes, we next probed whether activities of any particular transcriptional factors are altered in the absence of Pax6 that in turns could explain part of gene-expression program alterations. Towards this we applied integrated system for motif activity response analysis (ISMARA), which predicts the transcription factors that can potentially regulate the differentially expressed genes on the basis of binding motifs at the promoters of these genes [37,38]. ISMARA analysis predicted a number of transcription factors whose activity significantly changed in Pax6 mutant NPs. This included Pax6 and Sox2 that showed downregulation in their activity (Figure 4a–f). In line with these findings, ISMARA predicted targets of Pax6 and Sox2 were also found to be significantly downregulated in Sey cells (Figure 4b and e). Furthermore, ISMARA also predicted interaction networks of Pax6 and Sox2 with other transcription factors many of which are known to be important for neurogenesis (Figure 4c and f). Two transcription factors, TFAP2B and TCF4, were commonly identified in both Pax6 and Sox2 interaction networks (Figure 4c, f and g). Surprisingly, predicted targets of TFAP2B or TCF4 were highly enriched for genes related to neurogenesis (Supplementary Table S3). The role of TFAP2B in neuronal development as well as its interaction with Pax6 and Sox2 is unknown, however, our prediction provides potential insights of how cooperativity between different transcription factors contributes to neurogenesis. Targets of TFAP2B were also very significantly downregulated in Sey cells (Figure 4h) and network analysis further predicted its interaction with Pax6 as well as Sox2 in addition to many other interesting factors known to be required for neurogenesis (for example, Zeb1; Figure 4i). Overall, these observations suggest a cooperative function of transcription factors Pax6, Sox2 and TFAP2B in WT progenitors in gene activation as their activity and consequently their targets are downregulated in mutant cells.

Furthermore, ISMARA analysis also revealed upregulation in the activity of a number of transcription factors that are known to be important for non-neuronal lineages such as T (brachyury), Hnf1a and members of the Myf family (Figure 4j–r). Brachyury is an established mesoderm transcription factor [37,38], while Hnf1a is critical for liver differentiation [39] and Myf family of transcription factors are known to be crucial for heart development [37]. Target genes of these three transcription factors were significantly upregulated in mutant cells (Figure 4k, n and q). GO enrichment analysis showed that these target genes are involved in the development and function of non-neuronal tissues (Supplementary Table S3). Furthermore, the network for each of these factors mostly consisted of a non-overlapping set of transcription factors (Figure 4l, o and r). Overall, these findings show that Pax6-dependent gene regulatory circuitry induces activity of transcription factors that induce neurogenesis and repress others that promote non-neuronal lineage.

### Sox2 targets a large number of Pax6-bound gene promoters

**Figure 5:**
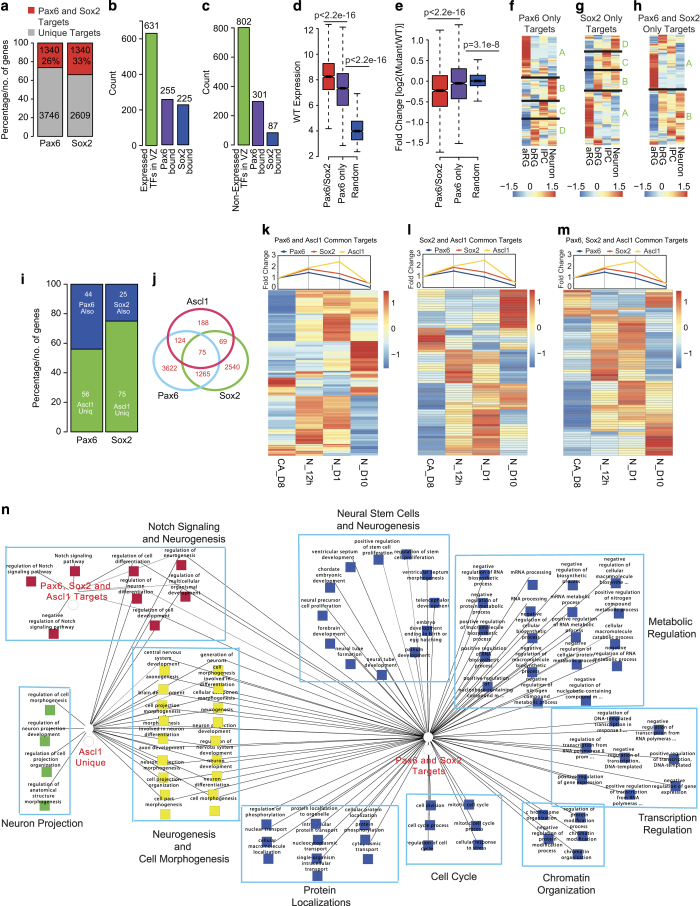
Pax6 and Sox2 act cooperatively to drive neurogenesis. (**a**) Stacked bar plot showing percentage of overlapping Pax6 and Sox2 targets. *y* axis represent percentage of Pax6 and Sox2 targets. (**b**) Bar plot showing expressed transcription factors in VZ as well as the ones targeted by either Pax6 or Sox2. (**c**) Same as in **b** but for not-expressed transcription factors. (**d**, **e**) Box plot showing expression and changes in expression of Pax6 and Sox2 targets, Pax6 only targets and random genes in WT (**d**) and mutant progenitors (**e**). *y* axis in **d** represents expression in WT cells while *y* axis in **e** shows fold change of expression between WT and mutant cells. *P*-value is calculated using Wilcoxon test. (**f**–**h**) Expression of Pax6 only (**f**), Sox2 only (**g**) and Pax6 and Sox2 targets (**h**) during several stages of neurogenesis. aRG, apical radial glial; bRG, basal radial glial; IPC, intermediate progenitors. (**i**) Stacked bar plot showing the overlap of Ascl1 targets with Pax6 and Sox2. *y* axis represent percentage of Ascl1 targets. (**j**) Venn diagram showing the number of overlapping targets of Pax6, Sox2 or Ascl1. (**k**–**m**): Heat maps showing expression of Pax6 and Ascl1 common targets (**k**), Sox2 and Ascl1 common targets (**l**) and Pax6, Sox2 and Ascl1 common targets during different steps of *in vitro* neurogenesis. Line plots above heat maps show expression of Pax6, Sox2 and Ascl1 in the same stages. Fold change is with respect to CA day 8 (CA_D8). (**n**) Comparative GO enrichment analysis of different set of genes represented as a network. Each square represent a GO term associated with a particular list, while edges provide information about the list to which the particular GO term is associated. If a GO term was found to be present in all three lists, it got connected with all three list nodes by edges. Pax6 and Sox2 target genes functions were further divided into sub-clusters based on similar GO terms.

We were intrigued by our observations that Sox2 activity is significantly reduced in Pax6 mutant NP cells. Both Sox2 and Pax6 are known to be important for the maintenance of the proliferative and developmental potential of NSCs [40]. Although it is known that Pax6 and Sox2 form a complex [41, 42], it remains to be investigated whether they function together in gene regulation at the same targets sites in the genome. We, therefore, compared our list of Pax6 target promoters with that of Sox2-bound promoters in NP cells derived from mouse ES cells in a previous study [43]. This analysis revealed that both Pax6 and Sox2 co-occupy a noticeable set of gene promoters, suggesting a potential cooperativity between these two transcription factors in gene regulation (Figure 5a and Supplementary Figure S4A; hypergeometric *P*-value: 1.28e-65). We next classified the genes encoding transcription factors, which were either expressed or repressed in the NPs *in vivo* (based on the transcriptome analysis of the E14.5 VZ cells) [25] and analyzed their promoter occupancies by Pax6 and Sox2. Pax6 and Sox2 were bound at the promoters of ~40% of the transcription factors expressed in the VZ (Figure 5b; hypergeometric *P*-value: 1.04e-22, Pax6 and 6.92e-27, Sox2). To our surprise, of the transcription factors that were not transcribed in the VZ, Pax6 occupied nearly 3.5-fold more targets compared with Sox2 (~37%, Hypergeometric *P*-value: 1.02e-20 Pax6, versus ~10%, Hypergeometric *P*-value: 1 Sox2; Figure 5c). In line with our previous observations, these results also suggest that Pax6-Sox2 complex preferentially bind to expressed transcription factors while without Sox2, Pax6 acts as a repressor. We were next curious to investigate whether the expression of Pax6 only bound target genes differs with respect to those bound by both Pax6 and Sox2 in WT and Pax6 mutant progenitors. Interestingly, genes bound by both Pax6 and Sox2 were significantly higher expressed in WT as compared with Pax6 alone or a random set of genes (Figure 5d). In line with these observations, these Pax6 and Sox2 common target promoters show higher accessibility as compared with Pax6 only and random promoters (Supplementary Figure S4B). Further supporting these findings, genes bound by both were more severely downregulated in mutant relative to Pax6 alone bound genes (Figure 5e). Together with previous observations, these results argue for an active cooperativity between Pax6 and Sox2 in regulating transcription of distinct set of genes in NP cells.

To further delineate and substantiate the expression dynamics of Pax6 and Sox2 targets, we explored recently published transcriptome data sets for distinct progenitor subpopulations (aRG, apical radial glial; bRG, basal radial glial; IPC, intermediate progenitors) as well as neurons from developing mouse neocortex [44]. Comparison of NP markers in our *in vitro* neuronal differentiation system and the above data sets showed that our ES-derived progenitors are apical in nature (Supplementary Figure S4C and D). Further comparison revealed an interesting pattern of expression for Pax6 only and Sox2 only bound genes compared with Pax6 and Sox2 co-occupied genes during neurogenesis (Figure 5f and h). The set of genes bound by either Pax6 or Sox2 and expressed in aRG were repressed in the immediate next stage (bRG) and remained repressed throughout neurogenesis (cluster A in Figure 5f and g). However, the genes bound by either Pax6 or Sox2 and repressed in aRG showed transcriptional activation in a stage-specific manner during neurogenesis (cluster B, C and D Figure 5f and g). In contrast, genes co-occupied by both transcription factors and expressed (cluster A) or repressed (cluster B) in aRG were immediately repressed or activated in bRG, respectively, and maintained this state throughout neurogenesis (Figure 5h). Overall, these observations suggest that the gene regulatory function of Pax6 at its target sites may be influenced by co-factors such as Sox2.

We next wondered whether other transcription factors expressed later during neurogenesis could function at Pax6 and Sox2 target sites when Pax6 and Sox2 are no longer available. To test this hypothesis we chose Ascl1, which is shown to be essential for the transition from neuronal progenitors to a neuronal state [45, 46] and neurogenesis is severely impaired in the absence of Ascl1 [47–49]. During neuronal differentiation from ES cells, Pax6 and Sox2 are simultaneously highly expressed in NP cells and following onset of neurogenesis, their levels decrease while Ascl1 levels are further increased (Supplementary Figures S4E). Using a recently published genome-wide binding data set for Ascl1 during neurogenesis [50], we found that Ascl1 shared 44% of Pax6 (hypergeometric *P*-value, 1.55e-22) and 25% (hypergeometric *P*-value, 3.24e-13) of Sox2 targets (Figure 5i and j). Interestingly, the targets common between Pax6, Sox2 and Ascl1 (*n*=75) included classical Notch pathway (*Id1*, *Id2*, *Hey1*, *Hes6* and *Dll1*) and neuronal (*Tubb2b*, *Robo1*, *Mapt* and *Pcdh10*) genes. We next explored how Pax6 and Sox2 targets that are also bound by Ascl1 are expressed during *in vitro* neurogenesis. Heat map visualization of these sets showed that such genes that are bound by Pax6 and/or Sox2 and also by Ascl1 mostly maintain their transcription state as cells exit NP state (higher Pax6/Sox2 and lower Ascl1 levels) towards initiating neurogenesis (lower Pax6/Sox2 and higher Ascl1 levels; Figure 5k and m). Furthermore, most of these genes acquire an opposite expression state in terminally differentiated neurons (no Pax6, Sox2 or Ascl1 expression). This suggests that a distinct sets of Pax6/Sox2 target genes might be targeted by other transcription factors in subsequent stages of neurogenesis to facilitate maintenance of their transcription state despite the later absence of Pax6/Sox2 itself.

To further explore the functional differences between the genes occupied by Ascl1 uniquely or Ascl1 along with Pax6 and/or Sox2, we performed a comparative GO term analysis to reveal their possible involvement in any specific biological processes (Figure 5n). The set of genes that were targeted by Pax6 and Sox2 only (blue squares) were enriched for a broad range of functions related to neural precursor or neural tube formation, cell cycle, transcription regulation, protein localization, metabolic processes and chromatin organization (Figure 5n). These genes were also enriched for functions related to neuronal differentiation and maturation (yellow squares). Interestingly, Ascl1 unique target genes were also enriched for these functions (yellow squares) indicating towards a functional takeover of neuronal development by Ascl1 (Figure 5n). The set of genes that were bound by Pax6/Sox2 complex and also by Ascl1 were exclusively enriched for Notch signaling and neuronal differentiation (Figure 5n, red squares). We also observed that the functional class ‘neuronal projection’ was uniquely attributed to targets that were also targeted by Ascl1 only (green squares; Figure 5n), supporting its known role in early neuronal development. Overall these data indicate that a subset of neurogenesis-related genes that are acted upon by Pax6 and/or Sox2 in NPs may also be targeted by other transcription factors such as Ascl1 for gene regulation during neuronal development.

### Pax6 directly induces expression of many known and novel NP-specific transcription factors

**Figure 6:**
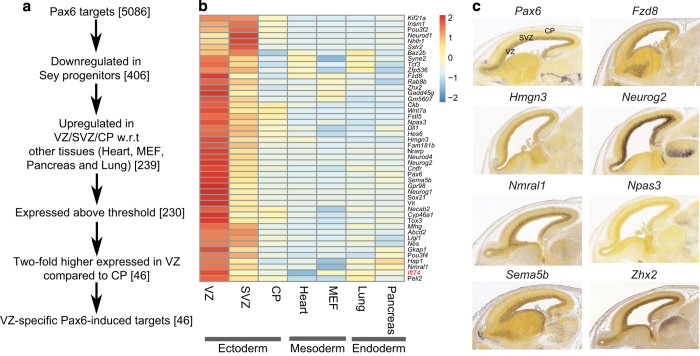
Pax6 directly induces expression of a large number of known and novel neural progenitor-specific transcription factors. (**a**) Flow chart showing the identification of genes that are specifically upregulated in neural progenitors *in vivo* and are regulated by Pax6. (**b**) Heat map showing the expression patterns of 46 genes identified in **a**. Red means high expression while blue means lower expression. (MEF, mouse embryonic fibroblast). (**c**) *In situ* hybridization images for known and novel Pax6 targets as derived from the Allen Brain Atlas (http://developingmouse.brain-map.org/).

We next attempted to further investigate the role of Pax6 in regulating the expression of NP-specific genes by performing a series of stepwise analyses. First, we selected the Pax6-bound mutant-downregulated genes that were significantly higher expressed in E14.5 cortical layers compared with other tissues (heart, embryonic fibroblasts, lung and pancreas). Then, we selected those factors that were at least two-fold upregulated in the VZ compared with the CP (Figure 6a). Interestingly, this final list of 46 genes primarily consisted of transcription factors, including established Pax6 targets and known regulators of NP identity (for example, Nestin, *Neurog1/2*, *Neurod1/4* and Notch pathway components, such as *Dll1* and *Hes6*; Figure 6b and Supplementary Figure S5A). Pax6 was also bound to its own locus likely for autoregulation as shown previously [12]. Of these 46 Pax6-target gene promoters, 17 were also co-occupied by Sox2 (data not shown). This analysis also identified many novel factors that have not been previously shown to function in regulating progenitor identity (Supplementary Figure S5B). The expression pattern of many of these genes was further validated by their *in situ* hybridization analysis in the embryonic cortex (Figure 6c) [51]. This analysis revealed how Pax6 functions as an upstream regulator of many known critical neurogenesis-related transcription factors, at the same time identified many previously unknown Pax6 targets that are specifically expressed in the cortex and warrant further investigation.

### Ift74 is a novel Pax6 target that contributes to neuronal migration

**Figure 7:**
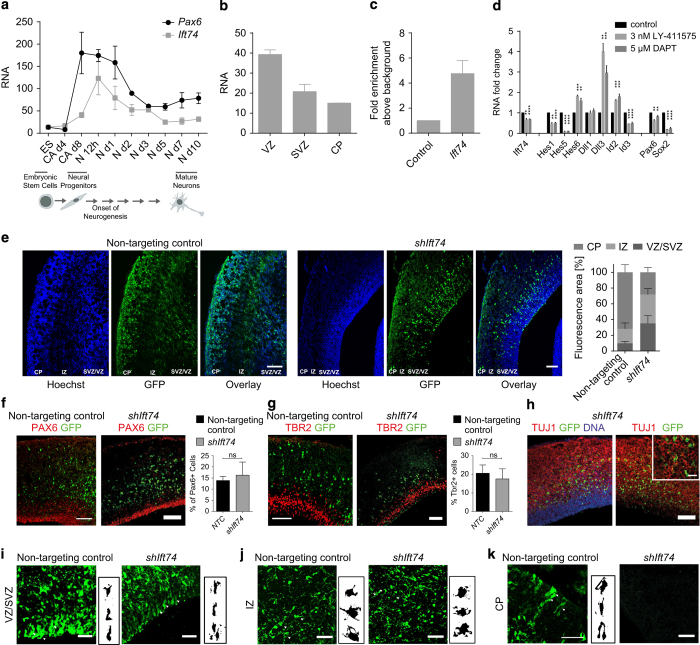
Ift74 contributes to neuronal migration. (**a**) Expression of *Pax6* and *Ift74* during neuronal differentiation of ES cells derived by real time quantitative PCR (RT-qPCR). Expression is shown for various stages of neuronal differentiation (ES cells, CA at day 4 before adding RA (CA d4), CA at day 8 (CA_D8)) and various time points during neurogenesis (TN at 12 h and day 1, 2, 3, 5, 7 and 10). mRNA expression is normalized to the housekeeping gene *Rpl19* (*n*=3, error bars show S.E.M). (**b**) Expression of *Ift74* (in RPKM) in VZ, SVZ, and CP dissected from mouse embryos at E14.5 derived by RNA-sequencing (GSE30765). (**c**) Pax6 ChIP-qPCRs to validate Pax6 binding at the promoter (−200±0 bp region) of *Ift74* at CA day 8 (CA_d8) (*n*=3, error bars shown as s.e.m.). Average enrichments are plotted normalized to input and further to an intergenic control region (control). (**d**) Fold change in mRNA levels in CA_d8 cells treated with γ-secretase inhibitor as compared with non-treated cells. ES cells were induced to undergo neuronal differentiation and treated every other day with 5 μM N-[N-(3,5-Difluorophenacetyl)-L-alanyl]-S-phenylglycine t-butyl ester (DAPT) (*n*=5), 3 nM LY-411575 (*n*=3) or dimethylsulfoxide as control from CA_d4 stage onwards. Expression of the shown genes were normalized to Rpl19 levels (ΔCT) and fold change with respect to the control is plotted (error bars show s.e.m.). Statistical significance were calculated with an unpaired *t*-test (*P<0.05; **P<0.01; ***P<0.001; ****P<0.0001). (**e**) Left panel: representative immunofluorescence images from coronal brain sections at E16.5 stained for DNA using Hoechst (blue). Green fluorescent protein (GFP; green) marks *shIft74* or non-targeting control electroporated cells; brains were electroporated at E12.5 and analyzed after 4 days. Scale bar: 100 μm. Right panel: quantifications of fluorescence signal in the GFP channel using ImageJ in the lower Hoechst dense region (VZ/SVZ), the intermediate less DNA dense region (IZ) and the upper Hoechst dense region (CP). Error bars reflect s.e.m. of three representative regions from two independently electroporated brains. (**f**) Representative immunofluorescence images of cortical brain slices electroporated at E12.5 with non-targeting control or *shIft74* and analyzed at E16.5. GFP (green) and PAX6 (red) stain is represented and the scale bar: 100 μm. All GFP-positive cells have been counted and the percentage of cells that also displayed PAX6 signal is plotted on the *y* axis *n*=2, error bars show s.e.m.). (**g**) Same as in **f** but co-stained with TBR2 (red) and accordingly quantified as above for TBR2-positive electroporated cells. (**h**) Representative immunofluorescence images of cortical brain slices electroporated at E12.5 with *shIft74* and analyzed at E16.5. GFP (green), TUJ1 (red) and DNA (blue) stain is represented and the scale bar: 100 μm. (**i**–**k**) GFP images to analyze cell shape and polarity for non-targeting control and *shIft74* electroporated brains in VZ/SVZ (**i**), IZ (**j**) and CP (**k**) region. White arrowheads point to multipolar roundish cells in the IZ and bipolar cells in VZ, SVZ and CP of non-targeting control and *shIft74* electroporated brains. Scale bar: 50 μm. On the right side of each image representative thresholded cells of the particular layer are represented.

We were next interested to deeply explore the function of novel genes that were directly bound and activated by Pax6 and whose expression was restricted to NPs. We focused on *Ift74* (intraflagellar transport (IFT) 74 homolog), which is a component of the IFT complex but remains a rather uncharacterised protein in the context of mammalian biology. *Ift74* forms a tubulin-binding module together with IFT81 that specifically mediates transport of tubulin within the cilium required for ciliogenesis [52]. To precisely map the kinetics of its expression with respect to *Pax6*, we analyzed their expression at various time points during the differentiation of ES cells into neurons via a NP state. As expected, this fine time course analysis during neuronal differentiation revealed that *Pax6* was most highly induced upon commitment to NPs and downregulated as soon as neurogenesis progressed (Figure 7a). Interestingly, analysis of *Ift74* at same time points showed that it reached its maximum expression levels few hours after highest *Pax6* expression, a stage that marks the transition of NP cells to neurons, and subsequently its expression was reduced upon neuronal maturation (Figure 7a).

To further substantiate our observations of *Ift74* induction in the context of *Pax6* expression *in vivo*, we next analyzed transcriptome data derived from the three layers of the E14.5 cortex (VZ, SVZ and CP) that showed the prominent expression of *Ift74* in the VZ of the developing mouse brain [25], which is where *Pax6* is also most highly expressed (Figure 7b and Supplementary Figure S1A). In order to confirm the direct binding of Pax6 at the promoter of *Ift74*, we performed ChIP assay in NP cells using Pax6-specific antibody. Real-time PCR analysis confirmed a high enrichment of Pax6 at a region upstream of the transcription start site of *Ift74* gene (Figure 7c). Given that Notch signaling is known to be essential for the self-renewal and identity of NP cells [30–32] and Notch effector transcription factor RBPJ showed occupancy at *Ift74* promoter in NSCs (D. Castro. Personal communication), we studied the effects of blocking Notch signaling on *Ift74* levels. To test whether Ift74 expression is regulated by Notch pathway, we inhibited Notch signaling using two independent inhibitors (LY-411575 and (N-[N-(3,5-Difluorophenacetyl)-L-alanyl]-S-phenylglycine t-butyl ester (DAPT)) and analyzed the expression of Ift74. These analyses showed that under both inhibitor treatments, Ift74 was significantly downregulated (Figure 7d). In addition, such blockage of Notch pathway also led to expected changes in the expression of Notch signaling components (Figure 7d) and is in agreement to previous studies [53]. Importantly, further in line with a critical role of Notch signaling in regulating NSC self-renewal and identity, we also found that the loss of Notch signaling also led to significant reduction in the expression NP markers (Pax6 and Sox2; Figure 7d). Given these observations, an alternative explanation for the downregulation of Ift74 by Notch inhibitors is that the NPs differentiate into more mature cell types that do not express Ift74. Since our earlier observations showed a direct induction of Notch signaling components by Pax6, the decrease in Ift74 expression upon Notch inhibition also suggests a potential functional cooperativity between Pax6 and Notch signaling in regulating the downstream gene-expression program.

Given the known function of *Ift74* in the transport of tubulin within the cilium that is required for ciliogenesis [52], we were tempted to investigate whether its induction by Pax6 serves to promote neuronal migration during later stages of neurogenesis. Towards this, we performed *in utero* electroporation using a plasmid encoding a tested shRNA against *Ift74* (Supplementary Figure S6A) at embryonic stage E12.5 and sacrificed the embryos for characterization at E16.5. Depletion of Ift74 via this shRNA *in vitro* does not result in cell death or impaired the cell-cycle progression (Supplementary Figure S6B and SC). In the control shRNA electroporated brains, the majority of electroporated cells were detected in the cortical plate and very few such cells were retained in the ventricular zone, reflecting proper cortical migration of newborn neurons (Figure 7e). *Ift74* shRNA electroporated brains, in contrast, showed a distinct phenotype where the majority of Ift74-depleted cells failed to migrate to the cortical plate (Figure 7e). Having observed such mislocalization of *Ift74*-depleted cells we were interested to uncover at which stage of neurogenesis these cells are perturbed. A staining of electroporated brain sections with Pax6 (Figure 7f) and Tbr2 (Figure 7g) showed no defect in the early neuronal maturation processes since the percentage of *shIft74* and non-targeting control electroporated cells showing similar Pax6 and Tbr2 expression. We next assessed whether *Ift74*-depleted cells migrating above the Tbr2 layer, express neuronal markers. A co-staining with Tuj1 revealed a clear overlap with *shIft74* electroporated cells (Figure 7h) indicating that these cells achieve neuronal identity but fail to fully migrate towards upper cortical layers. Although having a closer look into these images, many cells appeared to have multiple small processes and suggested that in the absence of *Ift74*, cells accumulate in the multipolar phase in SVZ/intermediate zone (IZ) region instead of migrating to CP.

For a detailed look into this phenomenon we took images at higher magnification of non-targeting control and *shIft74* electroporated cells from different cortical layers and further investigated the morphological appearance of such cells (Figure 7i–k). We observed that, in the ventricular/subventricular zone, control as well as *Ift74*-deficient cells displayed a comparable elongated polar appearance (Figure 7i). In the IZ, cells electroporated with either non-targeting control or *shIft74* again looked very similar and displayed a more roundish multipolar morphology (Figure 7j). However, while control cells subsequently transform back into a bipolar shape while migrating towards cortical plate, *Ift74*-depleted cells failed to do so and reside as multipolar cells in the IZ (Figure 7k). These observations suggest that cells deficient for *Ift74* are able to normally differentiate until the stage of immature projection neurons that enter the IZ and become multipolar but are not able to reorient into elongated, bipolar shape essential for proper migration to the cortical layer. Since neuronal migration is driven via epithelial to mesenchymal transition (EMT)-like mechanisms [54], we analyzed the role of Ift74 in cellular migration using mammary epithelial cells as an established cellular model of EMT [54–56]. We induced EMT and at the same time transfected shRNA against Ift74 in the epithelial cells and then measured cellular migration 4 days later. We find that shRNA-mediated depletion of Ift74 led to a significant reduction in the migration capacity of these cells (Supplementary Figure S6D and E). Taken together, these observations suggest that Pax6 and Notch signaling may cooperate in regulating gene expression (for example, of Ift74) as well as identify *Ift74* as a new Pax6 target that potentially has a role during neurogenesis likely via contributing to the migration of newborn neurons.

## Discussion

**Figure 8:**
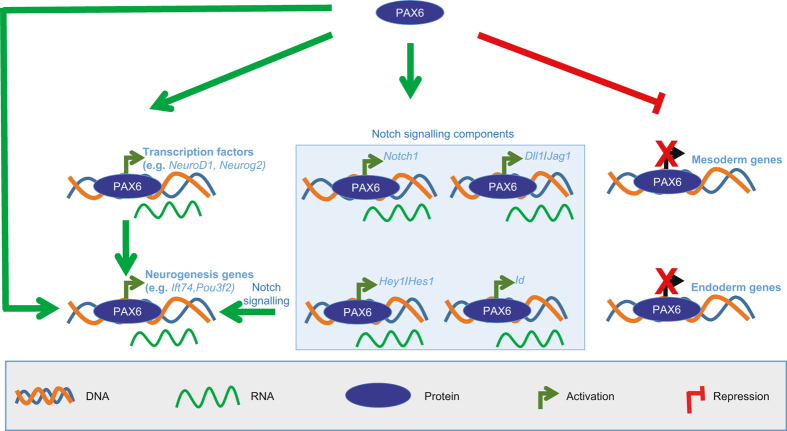
Pax6 regulates the gene-expression program at multiple levels to promote neuronal differentiation. Pax6 mediates the activation of neuronal (ectodermal) genes while concurrently represses the mesodermal and endodermal genes, thereby ensuring the unidirectionality of lineage commitment towards neuronal differentiation. Pax6 directly binds and activates expression of critical transcription factors and components of signaling pathways, all of which then function in concert to orchestrate downstream gene-expression program that drives neurogenesis.

Pax6 is a known master regulator of NP identity [8, 9, 15, 16, 57–63]. In this study, we attempted to uncover genes under the transcriptional control of Pax6 in NPs and identified downstream transcription factors that contribute to neurogenesis. We found that in NPs, Pax6 is targeted to many promoters that showed a distinct epigenetic state of open chromatin. Interestingly, many Pax6 sites are also occupied by Sox2, suggesting that they function together in gene regulation. Pax6 deficiency causes defects in the expression of its target genes, linking its binding to a function in transcriptional regulation. Strikingly, our analysis also revealed a dual role for Pax6, in which it activates the neuronal (ectodermal) genes while concurrently represses the mesodermal and endodermal genes. Importantly, Pax6 also directly induces the expression of a number of known as well as novel genes including transcription factors that are specifically expressed in NPs. We further show that one of the novel Pax6 target gene, *Ift74*, may have an important role during neurogenesis, likely via regulating migration of newborn neurons. Furthermore, our results also provide indication that Notch signaling contributes to the transcriptional induction of *Ift74* in NPs. Interestingly, Pax6 also directly binds at the promoters of many Notch signaling components and functions in their activation, suggesting the functional cooperativity between Pax6 and Notch signaling in regulating a downstream gene-expression program. Overall, our findings reveal how Pax6 regulates the gene-expression program at multiple levels to ensure proper execution of the neurogenic program, and at the same time ensures the unidirectionality of neuronal differentiation (Figure 8).

The complexity of Pax6 function has been suggested to arise from its interaction with various transcription factors to synergistically regulate target gene expression. In lens development, the transcriptional regulation of several crystallin genes by Pax6 is achieved in coordination with other transcription factors, such as Sox2 and Maf [64, 65]. Pax6 has been shown to form a complex with Sox2 to transcriptionally activate the *δ-* **c*rystallin* gene [41]. Sox2 is of further relevance because it is expressed in the developing mouse central nervous system from an early stage [66] and regulates the expression of fibroblast growth factor 4 (*Fgf4*) and Nestin, which are important in maintaining NSCs [67]. Our ISMARA analysis showed that the activity of Sox2 was significantly reduced in Pax6 mutant NP cells. Moreover, Sox2 co-occurred with Pax6 at many promoters in NP cells. Furthermore, these data also reveal that critical NP genes, such as Nestin, are co-regulated by Pax6 and Sox2. We also observed that the target genes co-occupied by Pax6 and Sox2 are expressed at higher levels, including those encoding important transcription factors, as compared with Pax6 only targets. These findings highlight the importance of the interplay between Pax6 and Sox2 in cooperative transcriptional regulation during neurogenesis, and at the same time also imply that critical neurogenesis genes may require co-activation by more than one stage-specific transcription factor. Furthermore, these observations also indicate that the gene regulatory potential of Pax6 may be determined by its partners and in this specific case, Sox2 occupancy drives it more towards a transcription activating role.

We also observed distinct expression dynamics of Pax6 and Sox2 unique target genes compared with those co-occupied by both factors during subsequent stages of neurogenesis. The genes bound by either Pax6 or Sox2 and expressed in aRG showed transcriptional activation in a stage-specific manner during neurogenesis where different gene-sets were found to be expressed in bRG, IPC and neurons. It may reflect availability of distinct factors or signaling pathways that become active at each of these stages to induce a set of genes critical for that particular stage of neuronal differentiation. On the other hand, all genes co-occupied by both Pax6 and Sox2 acquired changes in their expression state immediately after transition from aRG to bRG and this transcription state was maintained during later stages. This may also imply that genes that are required to be immediately switched on or off during differentiation of NPs may in some way benefit from being targeted by both Pax6 and Sox2.

Pax6 is believed to exert its effects by regulating critical downstream effectors during neurogenesis. A number of such examples have already been described, such as Fabp7, Neurog2, p27^kip1^, cell adhesion molecules (for example, L1 optimedin A, R-cadherin, δ-catenin and tenascin C), patterning molecules (for example, secreted frizzled-related protein 2 (sFRP2) and T-cell factor 4 (Tcf4)), Nkx2.2, Hoxd4, as well as other transcription factors, including Nfia, AP-2γ, NeuroD6, Neurog2, Tbr2, and Bhlhb5 [9, 15, 16, 57–63, 68, 69]. Our study is in line with the previous reports of a direct regulation of neurogenic transcription factors by Pax6 [17]. Importantly, our data also identified several additional genes including transcription factors that are directly induced by Pax6 in NP cells but have not been studied in the context of neuronal development (for example, *Bazb2*, *Hmgn3*, *Peli2*, *Vit*). Furthermore, although the role of Pax6 and Notch signaling in neuronal development is known for long, our data provide the first evidence that Pax6 also promotes Notch signaling by directly inducing the expression of critical components of this pathway (Figure 8).

Our data suggest that although Pax6 activates genes related to neuronal development, it represses the transcription of mesodermal and endodermal genes. Furthermore, ISMARA analysis also showed that Pax6 is required for the induction of transcription factors and their targets that elicit neurogenesis (for example, Sox2 and Tfap2b) and repress others that promote non-neuronal lineages (for example, Brachyury, Hnf1a and Myf family of transcription factors). These observations strongly imply that Pax6-driven gene regulatory program functions to ensure the unidirectionality towards neuronal differentiation.

During neurogenesis, NP cells undergo massive morphological and spatial changes that are tightly linked to cytoskeleton changes. For example, neocortical neurons arise by asymmetric division of radial glia progenitors (RG) in the VZ with a bipolar morphology and gradually become multipolar as they reach SVZ/IZ zone and move erratically. Subsequently, these cells undergo a multipolar to bipolar transition and move rapidly along RGs to the top of CP [70]. Defective ciliogenesis has been shown to accompany defects in neuronal migration in human ciliopathy phenotypes such as Meckel–Gruber syndrome [71]. We find that one of the Pax6-induced genes, *Ift74*, is highly expressed in NP cells as compared with other cell types. It has been shown in human cells that Ift74 and Ift81 build a tubulin-binding module whose binding to tubulin is important for ciliogenesis [52]. Depletion of *Ift74* by *in utero* electroporation during cortical development led to a retention of migrating cells in the lower layer of the cortex. Furthermore, some cells showed multiple small processes, indicating that the knockdown cells are in the multipolar phase in SVZ/IZ region and fail to migrate to the CP. These observations collectively suggest that the regulation of ciliogenesis and/or axonogenesis via *Ift74* might be essential for cortical development. Moreover, *Ift74*-depleted cells showed significantly reduced migration capacity during EMT *in vitro*. Importantly, as Notch signaling is known to be essential for proper radial migration of cortical neurons [72] and since we also found that Notch signaling is required for proper transcriptional induction of *Ift74*, it is likely that the previously observed defects in neuronal migration in the absence of Notch signaling are, at least in part, contributed by a loss of *Ift74* expression.

Taken together our findings provide novel insights into genomic localization and gene regulatory function of Pax6 during cortical development. Here we show that Pax6 targets a distinct class of epigenetically marked gene promoters, a number of which are co-occupied by other critical transcription factors such as Sox2. Our results suggest a model for a dual function of Pax6 upon neuronal commitment where it mediates the activation of neuronal (ectodermal) genes while concurrently represses the mesodermal and endodermal genes to ensure the unidirectionality towards neuronal differentiation. In addition, Pax6 also induces critical signaling pathways that further work together with Pax6 in guiding critical neurogenic events. Our findings highlight how the gene regulatory circuitry organized by a single factor is able to contribute to neuronal development at multiple levels. In addition to many established downstream effectors, this study has identified many novel targets that are bound and activated by Pax6 and warrant further investigation in cortical development. The *in utero* knockdown of one such gene, *Ift74*, during brain development resulted in impaired neuronal polarity and migration of newborn neurons. Overall, these findings reveal how Pax6 functions in the control of neuronal development at multiple levels to ensure unidirectionality and proper execution of the neurogenic program.

## Materials and Methods

### Cell culture

WT and Sey ES cells derived from blastocysts (3.5 PC) of mixed 129-C57Bl/6 background (called 159.2) were cultured and differentiated as previously described [19].

### Quantitative RT-PCR

Total RNA isolation, cDNA synthesis and quantitative RT-PCR were performed according to the manufacturer’s (Qiagen, Hilden, Germany) guidelines. Primer sequences will be provided upon request.

### ChIP assay

ChIP experiments were performed as previously described [73]. In brief, crosslinked chromatin was sonicated to achieve an average fragment size of 200 bp. Starting with 70 μg of chromatin and 5 μg of antibodies, 1 μl of ChIP material and 1 μl of input material were used for quantitative real-time PCR using specific primers. Primers covering an intergenic region were used as the control. The efficiencies of the PCR amplifications were normalized to those of the PCR products of the intergenic regions. The following antibodies were used: anti-Pax6 (Covance, Munich, Germany), anti-RNA Pol II: N-20 (Santa Crutz, Heidelberg, Germany), anti-H3K4me2: 07–030 (Millipore, Darmstadt, Germany), and anti-H3K27me3. Primer sequences will be available upon request. The ChIP material for Pax6 was used for ChIP-chip and for H3K4me2 and H3K27me3 was used for ChIP-seq as described later.

### *In utero* electroporation and imaging

The plasmids containing control shRNA (5ʹ-CAACAAGATGAAGAGCACCAA-3ʹ) or shIft74 shRNA (5ʹ-CGAGATCAAATGATTGCAGAA-3ʹ) were injected into the lateral ventricle of E12.5 mouse brains, which were given an electrical stimulation (34 V with 950 mA). Four days later, mice were killed and embryonic brains were fixed in 4% paraformaldehyde, dehydrated in 30% sucrose and embedded in O.C.T. (Tissue-Tek, Staufen, Germany ) on dry ice. The brains were frozen-sectioned into 12 μm slices with Leica CS3050S. After DNA staining using Hoechst and marker proteins (TBR2-ab23345 from abcam (Cambridge, UK), PAX6-PRB-278P-100, and TUJ1-D13AF00117 from Covance) confocal images were achieved through Leica TCS SP5 confocal microscope (Biberach, Germany) and analyzed using ImageJ (NIH, Bethesda, MD, USA).

### Cell viability analysis

NMuMG cells were transfected with shIft74 green fluorescent protein using lipofectamine according to manufacturer’s instructions and trypsinized after 48 h. After washing the cells two times with cold PBS, 1.5 million cells were resuspended in 100 μl Annexin binding buffer (0.1M hydroxyethyl-piperazineethane-sulfonic acid buffer (HEPES; pH 7.4), 1.4M NaCl and 25 mM CaCl_2_) supplemented with 5 μl Annexin V antibody labeled with APC (BD Pharmingen, Heidelberg, Germany) and incubated 5 min at room temperature in dark. Then cells were washed twice with 1 ml Annexin binding buffer, taken up in 400 μl Annexin binding buffer supplemented with PI and subsequently measured using the BD LSRFortessa Cell Analyser with BD FACSDiva software (Heidelberg, Germany).

### Migration assay

NMuMG cells were transfected using Lipofectamine 2000 following manufacturer’s protocol with shControl or shIft74 constructs during TGFβ-induced EMT. Cells were again transfected on the second day and fresh TGFβ was added to the culture. On the third day, wound was created using a 200 μl pipette tip. Light microscope images were taken at time 0 and 23 h and the derived data was further analyzed using ImageJ software to quantify closed area after 23 h compared with 0  h.

### Microarray expression data

The data from the Affymetrix GeneChip Mouse Gene 1.0 ST Arrays were imported into R (ver. 2.11.1), normalized with RMA [74] and annotated with annotation packages from the Bioconductor repository version 2.6 [75]. A modified version of the *t*-test [76] was used to identify the differentially expressed genes. The obtained *P*-values were corrected for by multiple testing using the Benjamini and Hochberg method. The data is deposited in GEO database with accession number GSE75256.

### ChIP-chip data analysis

The Nimblegen array intensity files from the GEO data set GSE30204 were imported into R (ver. 2.11.1), and the log2 enrichments (log2 bound/input ratios) for each individual probe were calculated using the package Ringo [77]. The Arrays were loess-normalized using the normalizeWithinArrays function of the Limma package. For each promoter, we calculated the average log2 enrichment values using data obtained from the overlapping probes. Promoters were defined as 900- bp windows (−700, +200) around the transcription start sites for genes defined in the Ensembl database (version 58_37k, http://www.ensembl.org) and the Refseq db (downloaded on 2010-05-28 from http://genome.ucsc.edu). The data is deposited in GEO database with accession number GSE75256.

### Selection of differentially expressed Pax6 targets

To derive a list of *bona fide* Pax6 targets, we compared the changes in expression of all Pax6 targets in the Pax6 mutant compared with those of the WT. We considered only those Pax6 targets that were at least two-fold differentially expressed with an FDR cutoff of 0.005, which provided a list of genes that were targeted by Pax6 and differentially expressed in the absence of Pax6.

### RNA-seq analysis

Tissue-specific data sets were obtained from the Gene Expression Omnibus with the following GEO accession numbers: GSE43194 (Heart E11.5), GSM723775 (mouse embryonic fibroblast E13.5), GSE49581 (Lung E14.5), GSM1150322 (Pancreas E15.5) and GSE30765 (VZ, SVZ and CP; E14.5). The reads were aligned to the mouse genome (mm9) using TopHat [78] with default parameters. The aligned reads were then provided as an input for the HTSeq_count utility from the HTSeq package. The raw read count files obtained from HTSeq-count were then processed for differential expression using the DESeq package [79]. The absolute quantification of the transcripts was performed using Cufflinks with default options. The expression data for apical, radial and intermediate progenitors and neurons were taken from Florio *et. al*. [44].

The data sets of VZ, SVZ and CP were obtained from described reference [25] in which authors have used laser microdissection (LSD) technique to separate out the three main layer of cortex from E14.5 embryos. In brief, dorsolateral and medial pallium areas were dissected to obtained progenitor cells residing in VZ layer. This layer mainly contains apical progenitor cells. Basal and intermediate progenitors reside in the SVZ-IZ regions and laser cuts were consistently performed at the border line of VZ and CP, which resulted in exclusion of subplate neurons. The CP layer neurons comprise of all differentiated neuronal subtypes present in adjacent layers, for example, Cajal–Retzius layer, VIb layer neurons.

The RNA-seq data for *in vivo* cortical neurogenesis contained well-defined populations of apical, basal and intermediate progenitors [44]. Authors in this study used fluorescence-activated cell sorting to isolate different cell populations from mouse neocortex. Apical radial glial were isolated based on cells that were positive for Dil, Prom1 and negative for Tubb3 while basal radial glial were Dil+, Prom− and Tubb3−. Intermediate progenitors (bIPs) were required to be negative for all three markers and neurons were isolated from Dil+, Tubb3+ but Prom− populations. These pure populations of progenitors and neurons were important for our analysis to analyze expression dynamics of Pax6/Sox2 target genes in NSCs in later stages of neurogenesis.

### ChIP-Seq analysis

The ChIP-Seq data sets for Sox2 were downloaded from GEO (GSE33059). In this study Sox2 ChIP-seq was performed on NPs derived from mouse ES cells. The reads were mapped to the mouse genome (build mm9) using Bowtie (version 0.12.9) [80] with default parameters. The mapped files were processed using MACS (version 2.0.10.2013071) [81] for peak identification using default parameters. Peaks falling at promoters were used to define Sox2 target promoters. H3K4me2 and H3K27me3 ChIP-Seq data was taken from GSE25533. The genes bound by Ascl1 in the differentiating NPs were obtained from Raposo *et al.* [50]. Briefly, in this study authors performed ChIP-Seq for Ascl1 after 18 h of ectopic expression of Ascl1 in NSCs. Pax6 ChIP-Seq peaks were obtained from a recent study [24]. We assigned each peak to the nearest gene and then shortlisted only those peaks that were found to be within ±1 kb around the transcription start site. These genes were then compared with our Pax6 targets.

### Enrichment analysis

GO cluster and phenotype enrichment analysis was performed using the ToppGene package [82, 83]. Only the top 20 enriched terms from the GO analysis were plotted. Pathway enrichment was performed using Genomatix.
