# Supplementary material for: Mapping gene regulatory circuitry of Pax6 during neurogenesis
Source: Cell Discov. 2016 Feb 9;2:15045–. doi: 10.1038/celldisc.2015.45 (PMC4860964; doi:10.1038/celldisc.2015.45)
Supplement: Supplementary Information [file celldisc201545-s1.pdf]

LEGENDS FOR SUPPLEMENTARY FIGURES AND TABLES

Supplementary Figure 1. Pax6 is expressed in neuronal progenitors *in vivo* and *in vitro*. **A.** Bar plot showing normalized RPKM values of Pax6 in several cell types (VZ; Ventricular Zone, SVZ; Sub-Ventricular Zone, CP; Cortical Plate, MEF: mouse embryonic fibroblasts). **B.** qRT-PCRs showing that *Pax6* is specifically upregulated in neuronal progenitors during *in-vitro* differentiation of ES cells into neurons (ES; Embryonic Stem Cells, CA D8; Cluster aggregates or Neuronal Progenitors (NP), TN; Terminal Neurons). Error bars represent SEM. **C-D.** Browser plots showing Pax6, H3K4me2, H3K27me3, Poll **II** and RNA levels at Suvar20h1 (C) and Dusp5 (D) promoters.

Supplementary Figure 2. A-B. Bar and line plots showing GO term enrichment analysis of all downregulated (A) and upregulated (B) genes in Pax6 mutant progenitors. Bar plots show numbers of genes for each enriched GO term (main x-axis), and the line represents the P-value for corresponding GO term (alternate x-axis). **C**. Scatter plot showing expression changes in *Pax6* mutant in comparison with Pax6 enrichment with 0 as the cut-off (red horizontal line). The x-axis represents log2-fold change between WT and *Pax6* mutant cells, and the y-axis represents Pax6 enrichment. Red vertical lines represent 2-fold change in expression.

Supplementary Figure 3. A. Heat maps showing expression of transcription factors that were found misregulated (down or up regulated) in Pax6 mutant cells during *in vitro* neurogenesis stages of wild-type ES cells. **B.** Network of Hedgehog pathway components among Pax6 target genes that are downregulated in *Pax6* mutant progenitors derived using Genomatix. **C.** Bar and line plots showing signalling pathway enrichment analysis of upregulated genes in *Pax6* mutant progenitors. Bar plots show number of genes for each enriched GO term (main x-axis), and the line represents the p-value for corresponding GO

term (alternate x-axis). **D.** Network of FGF pathway components among Pax6 target genes that are upregulated in *Pax6* mutant progenitors derived from Genomatix.

Supplementary Figure 4. A. ChIP-qPCR validations of common Pax6 and Sox2 target genes showing the Pax6 and Sox2 enrichment at their promoters. **B.** Box plot showing accessibility (FAIRE enrichment) of Pax6/Sox2 common, Pax6 only and random target promoters. **C-D.** Expression of selected progenitor genes during *in vitro* (C) and in *vivo* neurogenesis (D). **E.** Line plot showing the normalized RPKM values for Pax6, Sox2 and Ascl1 during i*n vitro* neurogenesis. The data is plotted as fold change in relation to CA_D8 stage.

Supplementary Figure 5. A-B. List of Pax6 target genes whose function in neuronal progenitors are known (A) or unknown (B). These data were derived using the list from Fig. 4B, followed by a literature survey.

Supplementary Figure 6. A. shRNA against *Ift74* significantly depletes its level in NMuMG cells. Knockdown efficiency of the shRNA against *Ift74* was tested using RT-qPCRs. An shRNA against luciferase was used as control. **B.** NMuMG cells were transfected with *shIft74*-GFP and sorted for GFP after 48h. For GFP positive (shIft74 transfected) and negative (non-transfected) populations Annexin V/PI FACS was performed and percentage of early apoptotic (Annexin V positive), late apoptotic (Annexin V and PI positive), dead (only PI positive) and alive cells (double negative) was plotted. Error bars reflect S.E.M. of biological replicates. **C.** NMuMG cells were transfected 48h with NTC or *shIft74* and cell cycle was monitored by measuring BrdU incorporation (1 h pulse treatment with BrdU after 47 h of transfection) and total DNA content determined by Hoechst. Percentage of cells in S-phase, G2/M-phase and G0/G1-phase was accessed by FACS and is plotted on the y-axis. Error bars reflect S.E.M. of two biological replicates. **D.** qPCR is performed to analyze the

expression of Ift74 after transient transfection of shControl and shIft74 constructs in NMuMG cells upon TGFb-induce EMT. **E.** Wound healing assay to assess the migration capacity of Ift74 depleted NMuMG cells upon TGFb-induced EMT.

Supplementary Table 1. All Pax6 target promoters above enrichment 0.

Supplementary Table 2. All differentially expressed genes.

Supplementary Table 3. GO enrichment analysis for Tfap2b, Tcf4, T, Myf Family and Hnf1a target genes.
