# Supplementary figures and images for: Mapping gene regulatory circuitry of Pax6 during neurogenesis
Source: Cell Discov. 2016 Feb 9;2:15045–. doi: 10.1038/celldisc.2015.45 (PMC4860964; doi:10.1038/celldisc.2015.45)

A

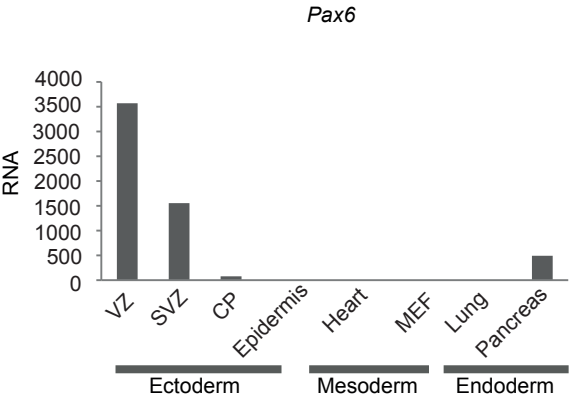

B

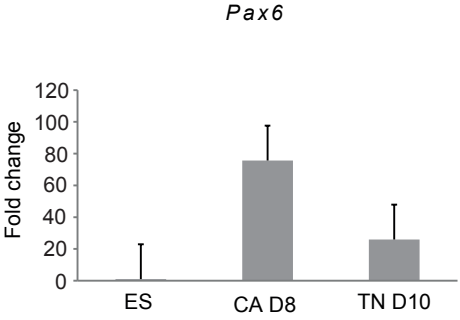

C

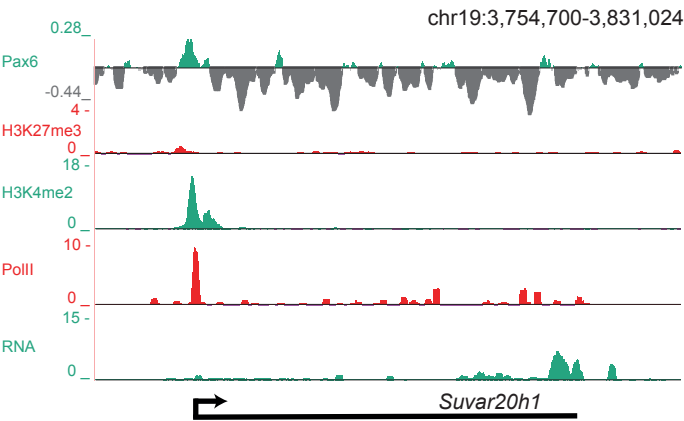

D

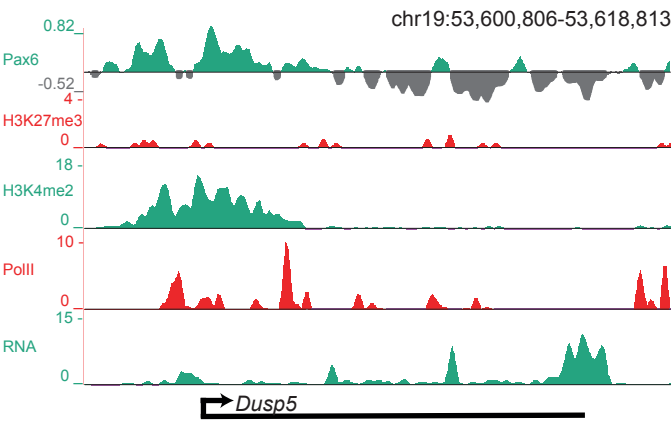

Supplement: Supplementary Figure S1 [file celldisc201545-s2.pdf]

A

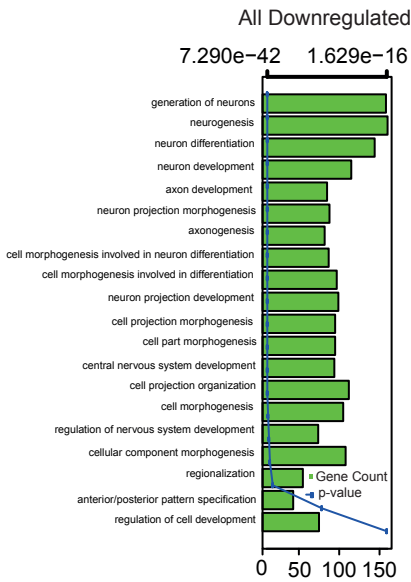

B

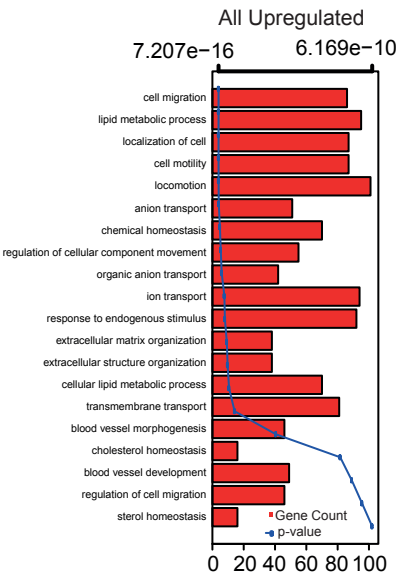

C

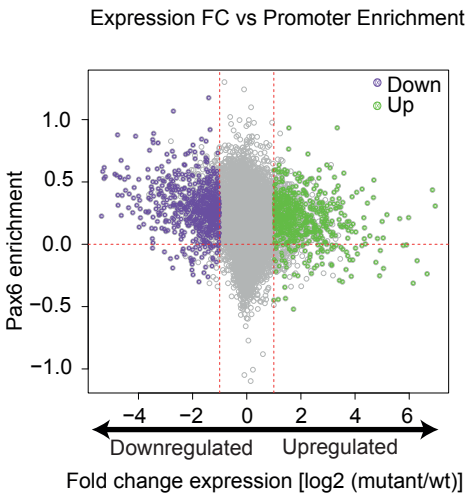

Supplement: Supplementary Figure S2 [file celldisc201545-s3.pdf]

Supplementary Figure 3

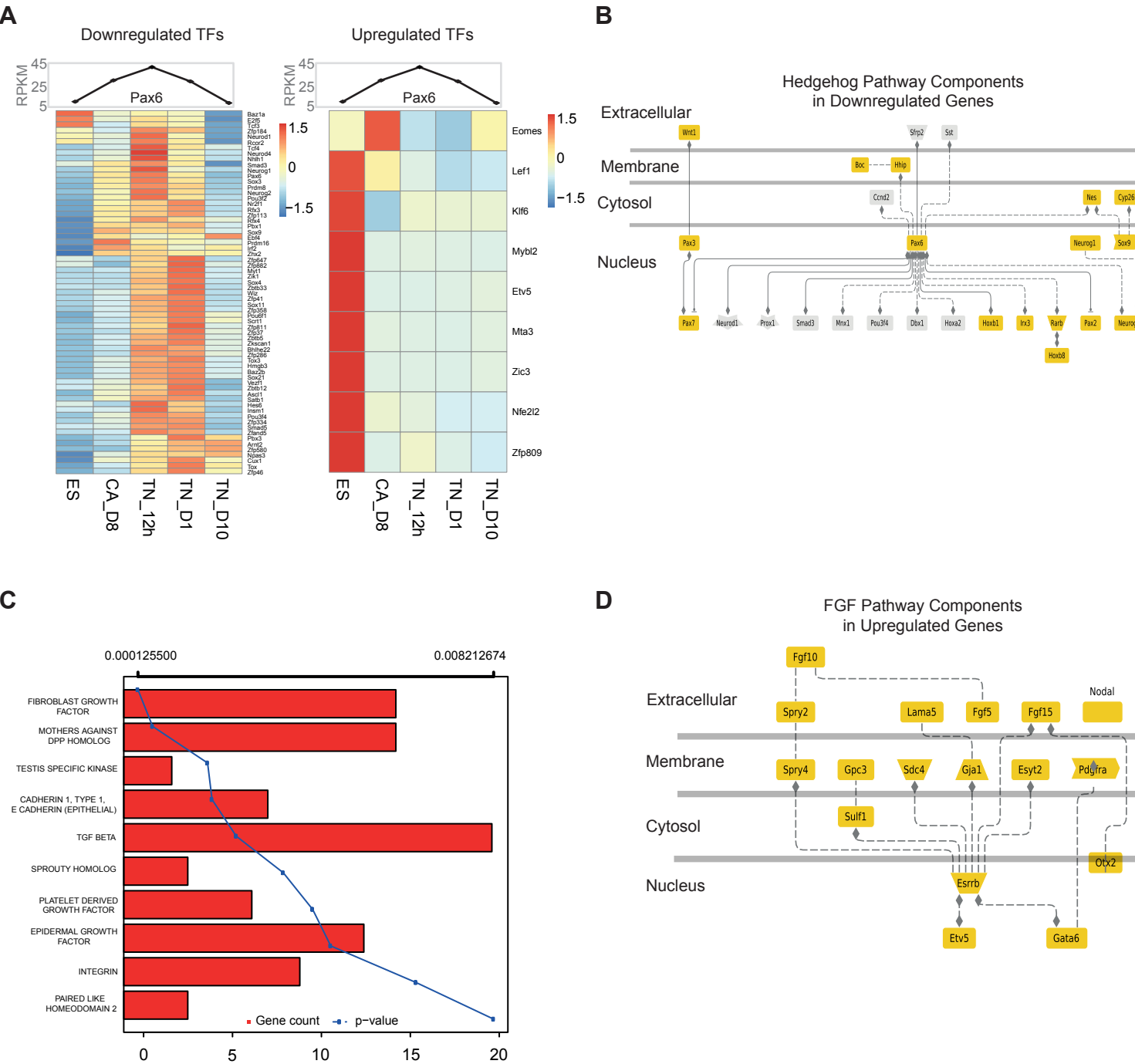

Supplement: Supplementary Figure S3 [file celldisc201545-s4.pdf]

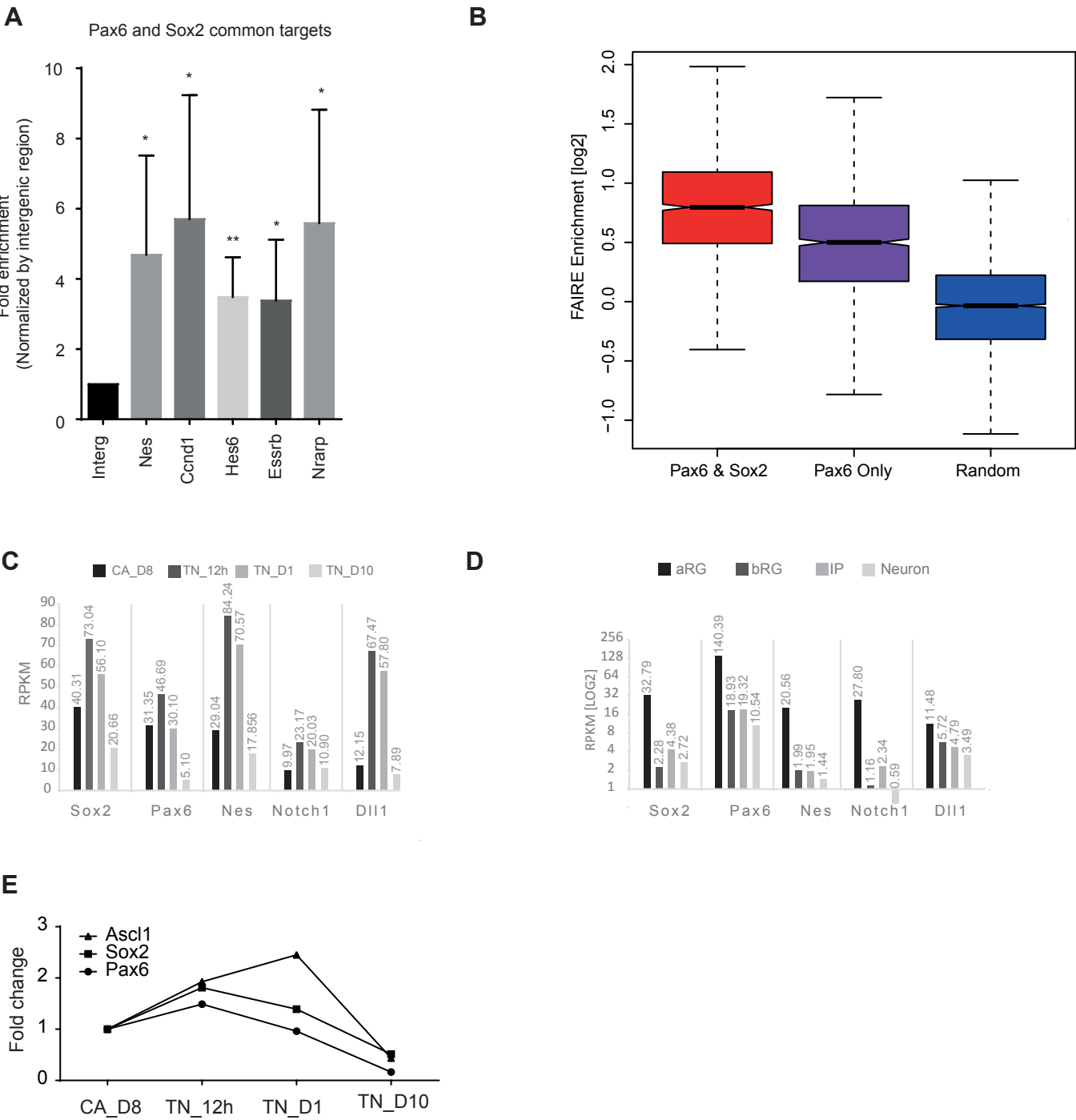

Supplement: Supplementary Figure S4 [file celldisc201545-s5.pdf]

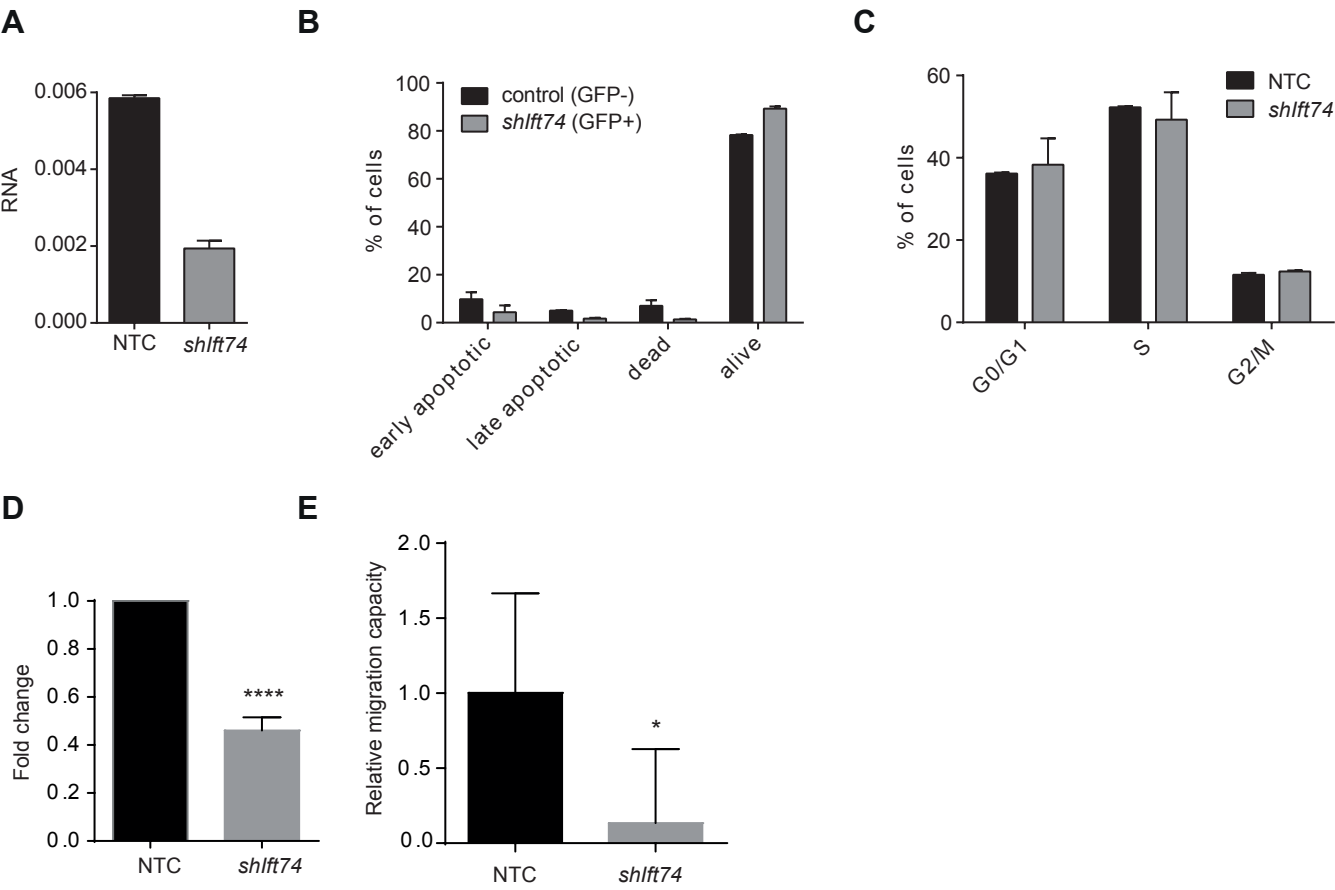

Supplement: Supplementary Figure S6 [file celldisc201545-s7.pdf]
