# Supplementary material for: Mapping gene regulatory circuitry of Pax6 during neurogenesis
Source: Cell Discov. 2016 Feb 9;2:15045–. doi: 10.1038/celldisc.2015.45 (PMC4860964; doi:10.1038/celldisc.2015.45)
Supplement: Supplementary Figure S5 [file celldisc201545-s6.pdf]

Supplementary Figure 5

Thakurela & Tiwari et al.

A

Function known in progenitors
Cntfr
Dll1
Fzd8
Gadd45g
Gpr98
Hap1
Hes6
Insm1
Mfng
Nes
Neurod1
Neurod4
Neurog1
Neurog2
Nhlh1
Npas3
Nrarp
Pax6
Pou3f2
Sema5b
Sox21
Sstr2
Tcf3
Wnt7a
Zhx2

B

Function not known in progenitors
Abcd2
Baz2b
Ckb
Fam181b
Fstl5
Gkap1
Gm5607
Hmgn3
Ift74
Kif21a
Nmral1
Peli2
Pou3f4
Rab8b
Syne2
Vit
Cyp46a1
Llgl1
Necab2
Tox3
Zfp536
