# Supplementary material for: Mapping gene regulatory circuitry of Pax6 during neurogenesis
Source: Cell Discov. 2016 Feb 9;2:15045–. doi: 10.1038/celldisc.2015.45 (PMC4860964; doi:10.1038/celldisc.2015.45)
Supplement: Supplementary Table S1 [file celldisc201545-s8.pdf]

geneName	Pax6.EnrichmentAverage
Phox2b	1.301556
Srp54c	1.242718
Slc6a5	1.175398
Dnah2	1.099468
Tubb2b	1.068407
Mab21l1	1.021732
Pax9	0.996935
Pcdh17	0.996529
Arid3b	0.976643
Wipi2	0.974852
Hmgn5	0.965801
Chchd5	0.962664
Ogt	0.948055
Nid1	0.934445
Arid5b	0.933248
Zbtb33	0.920008
Alx1	0.913766
Cdh6	0.889365
Dhrs3	0.889011
Dbx1	0.877794
Psmc1	0.876295
Prdm8	0.867375
Pigz	0.867261
Tob2	0.864305
Emx1	0.863583
Zdhhc15	0.862255
Lrfn5	0.86051
Prdm13	0.843637
Hspa1b	0.840717
Itgb8	0.839651
Mief1	0.836287
Fanci	0.827663
Nr2f2	0.827307
Car14	0.827063
Lhx9	0.821348
Pla2g6	0.820405
Spsb4	0.814583
Hyal2	0.814237
Dusp6	0.813841
Neurod1	0.813319
Gli1	0.804035
Cyr61	0.799893
Lhx1	0.796209
Kcnk5	0.795305
Gltscr1	0.793444
Tank	0.793394

Fezf1	0.788744
Plekhf1	0.781974
Id2	0.781523
Actn4	0.781416
Hexim1	0.780348
Dct	0.779797
Osr2	0.779217
Pcdh10	0.778654
9430023L2	0.776807
Six6	0.775691
Tmem167b	0.770072
Grrp1	0.769322
Tmem47	0.769179
Tbx20	0.764088
Hes7	0.764067
Smarcal1	0.759592
Hes3	0.755383
Cdh8	0.754125
Gm5136	0.752834
Wibg	0.752172
Zwint	0.749887
Sde2	0.749761
Gcnt4	0.749228
Zfp503	0.748776
Zfp516	0.746876
Marcks	0.746857
Tfap2a	0.743312
Litaf	0.7425
Ap3b2	0.740748
Jmjd8	0.737795
Fezf2	0.73662
Aifm1	0.735385
Klk10	0.735339
Ebf1	0.734728
Mapk11	0.732869
Dll1	0.729626
Pnpt1	0.727586
Pim2	0.724545
Zfp608	0.724388
Nedd9	0.722694
Zfp36l1	0.722009
Tal1	0.721726
Rangap1	0.721627
Ensa	0.719997
Ppox	0.718668
Cdk5rap2	0.718346
Tmtc2	0.712957

Gga1	0.710671
Atn1	0.710542
Abca7	0.707327
Zmiz1	0.707051
Ppp1cc	0.706299
Rgs2	0.706093
Nr2e1	0.703942
Gadd45g	0.703909
Chm	0.695762
Ireb2	0.694614
Glo1	0.694573
Edn1	0.693822
Efna1	0.693064
Vcan	0.692166
Cdh11	0.690111
Cbx8	0.686803
Slc25a21	0.686736
2610005L0	0.686288
Zfp865	0.686262
Evx1	0.685868
Gdpd5	0.684401
Bcl2l13	0.68397
Top1mt	0.682867
Gli2	0.682725
Fam173a	0.68208
Rell1	0.681323
Trib1	0.681147
Folh1	0.678574
Plekhg3	0.678419
Lphn1	0.67635
Dpf3	0.676062
Spag7	0.675749
Rad23b	0.674865
Robo1	0.674006
Crabp2	0.673126
Cngb3	0.672386
Syp	0.672036
Wnt11	0.671073
Nek7	0.669393
Robo2	0.669273
Ndufa4l2	0.669011
Ebf2	0.66898
Fbxo16	0.668231
Rpp25l	0.668225
Dus3l	0.66795
Mapk8ip2	0.665803
Dnaja3	0.665719

Tmem211	0.665535
Npas3	0.664578
Grpel1	0.664557
C1ql1	0.664527
Tfb1m	0.664527
Eif4ebp1	0.664224
Cacnb3	0.66258
Spata13	0.660689
Cd164l2	0.660496
Mycn	0.660346
Sema3a	0.659278
Rab33a	0.658286
Casq2	0.657473
Itprip	0.656568
Gad1	0.656298
Taf1c	0.655534
Tmeff2	0.655415
Slco5a1	0.655229
Atp5l	0.654604
Zfp12	0.651808
Cbll1	0.65056
Pold2	0.649896
Med12	0.649337
4930427A0	0.649004
Fos	0.646779
4632428N0	0.646731
Prelp	0.646038
Arhgef15	0.645788
Kdm6b	0.644756
Uvssa	0.641917
Pianp	0.641862
Clstn1	0.639836
Pcdh18	0.639835
Shisa7	0.639761
Sema5b	0.639554
Sh3pxd2b	0.639184
Gpr139	0.637942
Gja1	0.637922
Spp2	0.637523
Has2	0.636684
Olfml3	0.635834
BC052040	0.635811
Nckap5l	0.634511
Ccdc94	0.634157
1110038F1	0.63376
Myl6b	0.632693
Prr12	0.632619

Cabp1	0.631758
Slc2a4	0.630837
Adh5	0.630387
Rprd2	0.63007
Kcnj10	0.63003
Wdr13	0.630007
Snapc2	0.629641
Scn2b	0.629566
Draxin	0.628682
Mecom	0.628663
Fignl1	0.628567
Nlgn1	0.628489
Pim1	0.627856
Cipc	0.626718
Hmx2	0.626275
Unc93a	0.625801
Srgap3	0.624799
Ccnd1	0.623613
Sp8	0.622044
Snx33	0.621933
2700094K1	0.621766
Ier2	0.621074
Xrcc5	0.620984
Gtf2ird2	0.620785
Ercc6l	0.620408
Zmynd8	0.619947
4931428F0	0.619417
Cd24a	0.619352
Bcl2l11	0.619273
Ap3m2	0.619231
Ptpn21	0.618979
Isl1	0.61847
Gm5089	0.618091
0610011F0	0.617985
Dmrta2	0.617457
Mrpl38	0.617214
Chad	0.617101
Gad2	0.61635
Hoxd4	0.615836
Eif3d	0.614682
Pax3	0.614336
Eomes	0.613875
Usp19	0.613599
Ccno	0.613476
Pan2	0.613339
Fbxo32	0.612769
Trpm3	0.612233

Ypel4	0.611584
Cited2	0.611453
Atoh1	0.610623
Nudt18	0.609737
Jam2	0.609732
Msx1	0.609387
Snrnp70	0.609188
Ptpn14	0.608224
Ppp1r12a	0.607781
Slc27a3	0.606073
Plat	0.606064
Hoxa2	0.605472
Slc9a8	0.605456
Lhx8	0.604371
Ankrd40	0.60375
Ap1g2	0.603335
1700025G(	0.602527
Rap2a	0.602517
Gadd45b	0.602441
Ext1	0.602064
Sh3bp4	0.601886
Ptms	0.601619
Notch1	0.601598
Atf6	0.601456
Apc2	0.600961
Bahcc1	0.600723
Msx2	0.600636
Tagln3	0.600567
Wnt5a	0.600453
Smarcd3	0.600103
Kpna2	0.59987
Lrig3	0.597681
Zbtb16	0.597339
Tars	0.597244
Asf1b	0.597219
Klf6	0.596925
Mt2	0.596744
Cbx4	0.596194
Vps28	0.596047
Atp5k	0.59544
Bcl11a	0.595272
Ubald1	0.594755
Dnmt3a	0.59438
Zfp428	0.594136
Dbp	0.593635
Ephb4	0.593581
Sim1	0.59311

BC005537	0.592812
Tdrkh	0.592301
Efhc1	0.592076
Mgst3	0.591645
Tmem98	0.591637
Prrx1	0.591592
Hnrnph1	0.591413
Blvra	0.590687
Grasp	0.590254
Zfp609	0.589827
Zfp143	0.58937
Msn	0.58836
Tenm4	0.587749
G2e3	0.587165
Gstt2	0.586482
Set	0.586362
Ppp1r14a	0.586317
Asb4	0.586285
Epha7	0.586208
Gps2	0.586099
Pdzrn4	0.585833
Dnajb5	0.585248
Zmym2	0.585229
Hs3st3b1	0.584835
2810055G2	0.584377
Grm2	0.583681
Nynrin	0.583658
Hoxc12	0.583596
Vit	0.582793
Plin3	0.582481
Lyst	0.582151
Olig2	0.582079
Abhd17a	0.581624
2900060B1	0.580704
Hoxb1	0.580479
Ndp	0.580289
Barx1	0.580128
Rrm2	0.579982
Acp6	0.579688
Prr14l	0.579163
Cpt1c	0.579149
Myl12b	0.57867
Arhgap27	0.578296
Lrrc61	0.57807
Mtfp1	0.577889
Adpgk	0.577117
Pnpla6	0.577097

Hey2	0.576817
Ciz1	0.576664
Plekhh3	0.576536
Tap2	0.576399
Cltc	0.576194
Spag9	0.575935
Kxd1	0.575922
Mid1ip1	0.575885
Prpf4b	0.575762
Lzts2	0.575403
Mars	0.575232
Scnn1a	0.575141
Lrrn3	0.575125
Aloxe3	0.5751
Slc16a13	0.57465
Lrrtm3	0.574565
Tti2	0.57432
Leprel2	0.573853
Upk2	0.57368
Ell2	0.573091
St3gal2	0.572963
Efna4	0.572491
Aftph	0.571521
B4galnt2	0.57113
Ropn1l	0.571025
Glt28d2	0.570999
Pbx4	0.570694
Polr3k	0.570673
Timeless	0.57067
Nkx2-9	0.570526
5830415F0	0.570178
Epha4	0.569786
Maml3	0.569497
Adamts5	0.569311
Coasy	0.568922
0610009B2	0.568715
Cyb5r1	0.568669
Zc3h4	0.568531
Otp	0.568314
Mlx	0.567533
Vim	0.567347
Gapdh	0.567303
Pick1	0.56727
Neil1	0.566957
Mmp14	0.566823
Per1	0.56592
Arid3c	0.56579

Esyt1	0.565662
Llgl1	0.565473
Cep164	0.565352
Efnb3	0.565338
Cd2bp2	0.565032
Igbp1	0.564921
Hdac9	0.564906
Zhx2	0.564432
Shisa3	0.564187
Rab39b	0.56383
Vamp8	0.56312
Samd14	0.56282
Hoxb2	0.562702
Erbb4	0.562496
Plp2	0.56248
9-Sep	0.562077
Cxcl12	0.561733
Dcaf11	0.561637
Cntfr	0.561366
Ptges2	0.560372
Ifnar2	0.560358
Smim15	0.559829
Stc1	0.559821
Glis3	0.559539
Aff2	0.559526
Sox17	0.559308
Fmn2	0.558993
Gm13271	0.558936
Rpl8	0.558287
Rps19	0.558269
Pkp2	0.558002
Cand1	0.557964
BC061194	0.557774
Elovl6	0.557719
Kif21a	0.557217
Wfikkn1	0.55633
Tnik	0.556158
Dll3	0.55586
Car11	0.555519
Btg2	0.555159
Spon1	0.555107
Cs	0.554586
Ephb1	0.55434
Ube2c	0.554275
Hoxb8	0.554075
Dusp4	0.553985
Tuba1b	0.552824

Dbt	0.552582
Katnal2	0.552514
Pla2g15	0.552368
Gbx2	0.552079
Fam115a	0.550689
P4ha1	0.550647
Psmb8	0.550516
Ccdc14	0.550509
Pja1	0.550296
Tppp3	0.550165
Cul7	0.549959
Gdf11	0.549852
Atf3	0.549852
Arhgap23	0.548235
Zfp882	0.54814
Kcnh7	0.547893
Mmp16	0.547797
Tenc1	0.547275
Foxb1	0.54707
Ddn	0.546482
Foxg1	0.54546
Lpar6	0.54526
Tmem168	0.545185
Clpp	0.543765
Ankrd37	0.543517
Eml6	0.543128
Zfp60	0.543033
Adamts20	0.542499
Ptgr2	0.542174
Hoxc4	0.541909
Kif21b	0.541647
Ttk	0.541496
Tmed1	0.541423
Lamb2	0.541368
Urm1	0.54091
Apbb2	0.540859
Tecr	0.54062
Esrp2	0.540408
Gabarap	0.540395
2810408M	0.540322
Ap1b1	0.540217
Slc43a2	0.539817
Fam84a	0.539771
Hoxa3	0.539727
Cpne7	0.539487
Plscr3	0.539485
Dpysl3	0.539451

Epha3	0.538778
Acbd5	0.538749
Kazald1	0.538656
Dgka	0.538172
Emb	0.538145
Setd6	0.537777
Cstf3	0.536571
Txn2	0.536402
Tmem107	0.535987
Crlf3	0.535942
Sema6a	0.535768
Hs3st5	0.535543
Ednrb	0.535508
Nup153	0.535463
Slitrk1	0.535279
Ntng2	0.535198
Kirrel	0.53464
Plekha2	0.534196
Nipbl	0.53404
Arrdc3	0.534027
Sprn	0.533743
Irs1	0.533618
Mnt	0.533614
Spred2	0.533583
Kcnk13	0.533129
Polr2e	0.533038
Ctgf	0.533012
Apool	0.532584
Foxl1	0.53202
Ubb	0.531951
Msx3	0.531388
Pdk2	0.531193
Elp6	0.531124
En1	0.531077
Oaf	0.531074
Exosc9	0.53075
Gata4	0.530502
Pvrl1	0.530449
Ppm1l	0.529578
Junb	0.528849
Mat2b	0.528689
Cherp	0.528356
Rarb	0.528213
Id3	0.528176
Pnkp	0.528018
Polr2k	0.527968
Usp32	0.527606

Dscaml1	0.527284
Gzf1	0.527215
Lama1	0.527202
Sash1	0.527174
Tex26	0.527008
Rps13	0.526773
Zbtb9	0.526605
1110001J0	0.526563
Cit	0.526556
Tom1l1	0.526367
Rhbdf2	0.526263
Hoxb9	0.525926
Fbxw17	0.525764
Fbf1	0.525727
Pkm	0.525469
Mak16	0.525112
Smpd3	0.525102
Ajuba	0.524871
Mab21l2	0.524843
Smim4	0.524697
Itga5	0.524558
Hist3h2ba	0.524313
Lgals1	0.52413
Mark3	0.52376
Tcp11l2	0.523658
Zfhx4	0.523613
Gfra1	0.523506
Thy1	0.52314
Rmnd5b	0.523075
Tia1	0.522848
Vasn	0.522809
Wfikkn2	0.52226
Vwc2	0.522145
Mllt10	0.522096
Cyp26b1	0.521113
Asb1	0.520735
Pax6	0.520701
Psen1	0.520625
Ltb	0.520562
Zfp566	0.520561
Dctn3	0.519709
Ap1s1	0.519543
Agbl5	0.519395
Marveld3	0.519189
Meox2	0.51876
Tfap2b	0.518586
Hivep1	0.518202

Slc2a12	0.51806
Hook2	0.517966
Rnf122	0.517931
Rrh	0.517501
Chrnb4	0.517345
Trp53bp2	0.517231
Suv420h2	0.517204
Egln3	0.516985
Dmrt3	0.516559
Ptch1	0.51653
Traf7	0.516488
Elmo2	0.516387
Araf	0.516324
Tmod3	0.51613
Dgkb	0.516121
Stmn4	0.516047
Mybbp1a	0.516011
Otx2	0.515365
Sphk1	0.515155
Taf2	0.515128
Chd9	0.515008
Txnip	0.514666
Ckap2	0.514349
Evx2	0.51412
Neurog2	0.514072
Bmf	0.513938
Ppp1r9b	0.513918
Sugt1	0.513726
Ndufs8	0.513388
Dnaic2	0.513289
Bcas2	0.512796
Hspa2	0.512691
Slain1	0.512414
Rbm24	0.511813
Slc25a39	0.511678
Sacm1l	0.511326
Sfmbt2	0.511076
Gsx1	0.510955
Flrt2	0.510216
Ercc6	0.509907
Celsr2	0.509748
Onecut1	0.509346
Porcn	0.509265
Churc1	0.508635
Abhd3	0.50855
Cdkn2a	0.507965
Mmp23	0.507957

Lef1	0.507769
Dap	0.507706
Osbpl7	0.50768
Nptx2	0.507552
Tspan7	0.507417
Coq3	0.507381
Nkain3	0.507375
Soga3	0.506946
Spint2	0.506578
Wbp2	0.506532
Leprotl1	0.506367
Isoc2b	0.506238
Slc25a14	0.50605
Ubxn2b	0.505968
Cdca7	0.505782
Dmbx1	0.505529
Psmd3	0.505468
1110034G2	0.505154
Sfrp2	0.505094
Syvn1	0.505058
Hap1	0.504972
Sall1	0.50478
Calr	0.5047
Pde4c	0.504591
Boc	0.504258
Gpx1	0.503705
Mb21d2	0.503665
Hmgcr	0.503358
Kctd11	0.503218
Nkx2-3	0.503146
Ahcy	0.50276
Cad	0.50256
Stxbp6	0.502518
Pqlc3	0.502487
Espl1	0.502407
Lrch1	0.502399
Tbx3	0.502386
Gls2	0.50232
Kcnj2	0.502299
Cpsf2	0.502227
Banp	0.502205
Klhl26	0.502006
Gpr98	0.501784
Cox5a	0.501514
Dnase2a	0.501453
Gramd3	0.501265
Cd2ap	0.501065

Fgf9	0.500945
Tfe3	0.50035
Nol4	0.500321
Rwdd1	0.500184
Tomm6	0.500127
Fig4	0.500021
Mapk9	0.499892
Pax5	0.499726
Tmem33	0.499582
A430105I1	0.499481
Zfp689	0.499356
Snrpc	0.499003
Col1a1	0.498923
Dusp5	0.49885
Nfatc1	0.498768
Atoh7	0.498717
Ttc9b	0.498665
Tagln2	0.498653
Birc5	0.498525
Pdpn	0.498495
Rhob	0.498164
Gpr156	0.498127
Aqp5	0.498072
Abcd2	0.498064
Zfp386	0.49803
Zic5	0.497932
Mkx	0.497867
1700030J2	0.497681
Bend5	0.497078
Zfp41	0.496941
Rnasel	0.4959
Cryge	0.495682
Cdh24	0.495584
Atp2a1	0.495537
Cald1	0.495463
Ppfia2	0.495167
Onecut2	0.495034
Supt4a	0.495001
Smad3	0.494929
Ccdc90b	0.494862
Cdc20	0.494556
Pif1	0.494293
Scd2	0.493833
Rpl7l1	0.493831
Zc2hc1c	0.493776
Abtb1	0.493754
Adsl	0.493609

Lypd1	0.493538
Ngdn	0.493476
Kcnj3	0.493455
Nab2	0.493373
Mlst8	0.493278
Zbtb45	0.493227
Stk38	0.492965
Cnn2	0.492523
Cdh7	0.492522
Selk	0.4919
Slc25a10	0.491887
Alad	0.491768
Rnf215	0.491535
Dcps	0.491447
Bicd2	0.49129
Ift27	0.491241
E130309F1	0.491066
Kcnc3	0.490902
Tspan31	0.49088
Brd2	0.490768
Dsp	0.490697
Fam222b	0.490635
Mon1b	0.490301
Brpf1	0.490231
Pias4	0.489971
Dennd2a	0.489798
Sh2b1	0.489536
Ltbp4	0.48951
Tgfbi	0.489305
Fam183b	0.489303
Sf3a3	0.489282
Lyrm7	0.489221
Ccdc6	0.489036
Arrb2	0.488879
Nup50	0.488735
Ppp1r18	0.488675
Dcdc2a	0.488612
Mllt3	0.488608
Srrt	0.4885
Sntb2	0.488438
Ldoc1	0.4883
Gabrb2	0.488261
Sos2	0.488161
Rtn4rl2	0.487908
Anp32a	0.487886
Tll1	0.487289
B3gnt9	0.487197

Osr1	0.486966
Wdr35	0.486876
Pipox	0.486367
Sema4c	0.485771
Utp3	0.485747
Katnb1	0.485675
Hmgn3	0.485628
Ccdc177	0.48532
Alcam	0.485168
Fam92b	0.485101
Psma6	0.485073
Atat1	0.484953
Nsun4	0.484883
Pea15a	0.484831
Ptprk	0.48474
Bcas3	0.484434
Nr2c1	0.484332
Fzd5	0.484271
4930404N1	0.484147
Prkci	0.484092
Igsf8	0.483973
Ulk4	0.483664
Stk25	0.483596
Hoxa6	0.483587
Ush1g	0.483585
Unc5c	0.483542
Pou4f2	0.483523
Rere	0.483432
Zfp62	0.482647
Reep2	0.482628
Adm	0.482381
Lgr4	0.482067
Abhd2	0.481952
Cpt1a	0.481857
Col2a1	0.481833
Serpinh1	0.48145
Chst8	0.481381
Keap1	0.4813
Lifr	0.481249
Sptbn1	0.481164
Mfsd3	0.480981
Zmynd10	0.480752
Phka1	0.480536
Lin28b	0.480473
Rraga	0.480423
Hoxb7	0.480297
Rdh5	0.479941

Ccdc84	0.479767
Ndufa6	0.47973
Ngfr	0.479705
Hapln4	0.479629
Grtp1	0.479475
Zfp51	0.479093
Hey1	0.479078
Nup85	0.478834
Six2	0.478697
Myg1	0.478613
Cdc14a	0.478595
D2hgdh	0.478533
Zfp207	0.478306
Ndufaf4	0.478218
Fam73b	0.478148
Ndufs7	0.478102
Rps26	0.478086
Zdhhc17	0.478044
Khk	0.478004
Cenpc1	0.477944
Trim2	0.477911
Tlx3	0.477839
Ccnl2	0.477833
Plscr1	0.477831
Ankrd33b	0.477778
Crebl2	0.47768
Runx1t1	0.477519
Katnal1	0.477517
Ilf3	0.477437
Runx1	0.477319
Slc38a3	0.477197
Scamp1	0.47696
Neurog3	0.476952
Gtf3c5	0.476918
Sin3a	0.476726
Opa3	0.476652
2310047M	0.476642
Fpgs	0.476498
Gata2	0.476475
Stc2	0.476464
Vcl	0.476455
Ptgir	0.476383
Nrcam	0.476334
Ttc21b	0.476088
Swsap1	0.475624
Pcgf1	0.475484
Ttyh2	0.475442

Grhl2	0.475321
Mxd3	0.474951
Large	0.47487
Dlx1as	0.474709
Zfp811	0.474691
Tesk2	0.47437
Arid5a	0.474025
Nxph1	0.473904
Pacsin1	0.473875
Scamp3	0.473741
Htra4	0.473727
Khsrp	0.473708
Timm10	0.473597
Mtmr11	0.473584
Grb2	0.47338
Tagap1	0.473327
H2-Q7	0.473286
Clns1a	0.473156
Abracl	0.47312
Tssc4	0.473055
Nbas	0.47292
Slc39a11	0.472889
Gfra2	0.472753
Sp1	0.472746
Msi2	0.472718
Adam19	0.472709
Nprl3	0.472541
Uba1	0.472467
Abt1	0.472461
Tctn1	0.47242
Zfp28	0.472243
Ccdc171	0.472165
Dhrs7	0.472038
Igfbp2	0.472031
Csmd3	0.471713
Mrps23	0.471706
Dpysl4	0.471626
Ddx56	0.471477
Tmem136	0.471346
C030039L0	0.471337
Pcdh9	0.471041
Irx3	0.470949
Tubb3	0.470903
Fzr1	0.470634
Rrp36	0.470566
Fes	0.470505
Fam65a	0.470473

Rbm15	0.470251
Gpr180	0.470184
Siva1	0.470129
Aim1	0.469907
Caly	0.469882
Tmem11	0.469553
Dab1	0.469395
Dlx1	0.469177
Agk	0.468975
Nmral1	0.468909
Bcam	0.468861
Gramd1b	0.468462
Rpl34	0.468405
Tmem259	0.468396
Ywhae	0.468326
Hoxb13	0.467851
D10Jhu81e	0.467787
Kcnn2	0.467729
F11r	0.467725
Casq1	0.467691
Rab11fip1	0.467419
Sh3rf3	0.467249
Csrp1	0.466868
Fam120b	0.466368
Atp13a2	0.466347
Wdr95	0.466144
Isl2	0.466102
Limd1	0.466081
Plxna2	0.465846
Wdr18	0.465698
Maml1	0.465634
Zfp87	0.465304
Tceb1	0.465226
Grn	0.465044
Cep152	0.465029
2510009E0	0.464802
Hoxc6	0.464762
Loxl2	0.464729
Hoxd10	0.464652
Tle6	0.4646
Foxa1	0.464495
Lsm4	0.464467
Cflar	0.464264
Cenpv	0.464232
Cdk12	0.464134
Gltscr2	0.463944
Nupl1	0.463781

Ndufa10	0.463778
Id4	0.463776
Otop1	0.463762
Tshz2	0.463684
Gpc6	0.463267
Tomm40	0.463229
Traf5	0.463049
Egln1	0.463015
Tcf3	0.46283
Etv1	0.462668
Gss	0.462439
2700060E0	0.462398
Ranbp9	0.462344
Slc12a4	0.462013
Olig3	0.461763
Usp44	0.461358
Egr3	0.461113
Kif2b	0.460677
Hs1bp3	0.460518
Cpa5	0.460481
Troap	0.460421
Tldc1	0.460174
Carhsp1	0.460152
Eef1g	0.460069
Ralb	0.460051
Pak6	0.459922
Lrrcc1	0.459705
Ccdc60	0.459624
Atg5	0.45961
Rab3c	0.459542
Cdc42ep4	0.459524
Auts2	0.459325
2010107G2	0.458948
Trmt61a	0.458896
Olfm4	0.458843
Jun	0.458673
Zfp397	0.458519
Wnt1	0.458173
Zfp36	0.457976
Slc35g2	0.457799
Tmem220	0.457658
Tyk2	0.457439
Psmd1	0.457252
Gdnf	0.457225
Mrpl39	0.45704
Ntrk2	0.456955
Flrt3	0.456893

9430008C0	0.456667
Adssl1	0.45634
Fam53c	0.456091
Ppan	0.456022
Lars2	0.455941
Lphn3	0.45583
Hist2h4	0.455567
Prox1	0.455534
Rprm	0.455471
Tbr1	0.455415
Sln	0.455323
Prmt8	0.45531
Ldlr	0.455251
Nkx6-2	0.454741
Mpp2	0.454542
Yipf6	0.454495
Ptchd4	0.454321
Ache	0.454243
Mef2d	0.454234
Rplp1	0.454174
Sesn3	0.454079
Frs3	0.454076
Pcdh8	0.453898
Chrm4	0.453844
Ror2	0.453735
Foxd1	0.453662
Tekt4	0.453583
Nup133	0.453486
Bhlhe41	0.453467
Nek4	0.453388
Derl1	0.453331
Bnip3l	0.453327
Smdt1	0.453316
Myl6	0.453225
Hyls1	0.453012
Gabrb1	0.452765
Antxr1	0.452681
Fam136a	0.452523
Ptprg	0.45205
S1pr1	0.452041
Arid3a	0.45177
Timm21	0.451702
Patz1	0.451583
Prmt1	0.451184
Akr1b3	0.451148
Riok3	0.45114
Ralgps2	0.451124

Fam207a	0.451092
Flywch1	0.451004
Tpbg	0.450744
Rassf5	0.450678
Nadk2	0.450671
Enho	0.450632
Acly	0.450612
Nr5a2	0.450503
Foxb2	0.450326
Pde1b	0.450322
Mrpl28	0.449972
Ppcdc	0.449903
Nup107	0.449824
Thap7	0.449686
Vangl1	0.449588
Cnot8	0.449324
Cacybp	0.44932
Pitpnc1	0.4493
AI464131	0.449149
Khdrbs1	0.449145
Zfp949	0.449123
Tnpo2	0.448901
Mapk6	0.448717
Tars2	0.448702
2610034B1	0.44862
Cecr2	0.448401
Baz2a	0.448314
Anxa11	0.448295
Zfp637	0.448243
Lect1	0.448202
Slc6a4	0.448097
Gtpbp2	0.447974
Pfkp	0.447959
Slit2	0.447922
Crip1	0.447876
Bcat1	0.44777
Npr1	0.447726
Slc6a15	0.447695
Stard3	0.447565
Cnih1	0.447482
Ttc12	0.447366
Kcnc2	0.447321
Cyp26a1	0.447306
Arrdc4	0.447072
Ak1	0.447028
Pcbp4	0.447024
Kif6	0.446975

Ttc7b	0.446657
Syt11	0.446614
Rassf1	0.446607
Amph	0.446531
Rgs20	0.446365
Serpinb8	0.446258
Zfp691	0.446244
Robo3	0.446197
Sox14	0.44614
Xbp1	0.446105
Gxylt1	0.445797
Ppif	0.445792
Tbc1d9	0.445737
Hnf1b	0.445733
Alyref2	0.445522
Snx15	0.445499
Psmd12	0.445493
Cxcr4	0.445399
Tbcd	0.445337
Lactb	0.445091
Adra1a	0.44505
1700007G1	0.444994
Fam96b	0.444919
Gstm1	0.444876
Tsfm	0.444764
Dsg2	0.444685
Map3k14	0.444516
Trex1	0.444485
Sptlc1	0.444444
Rnf24	0.444291
Cep97	0.444267
Arl6ip1	0.444005
Fdft1	0.443865
Ccdc28b	0.443862
U2surp	0.443672
Rhof	0.443654
Orai2	0.443639
Cct8	0.443633
Prnp	0.443559
Alg3	0.443554
Narf	0.443472
Stt3a	0.443447
Dab2	0.443117
Phf12	0.443029
Pum1	0.442966
Frzb	0.442951
Lysmd3	0.442941

Vwa7	0.442881
Hspa9	0.442871
Tsc22d4	0.442819
Wnt9a	0.442774
Zbtb18	0.442525
Eya4	0.442476
Vps36	0.442462
Mthfsd	0.442405
Nrde2	0.442399
Cactin	0.442335
Cep70	0.442307
Mast3	0.442131
Deptor	0.442057
Kat7	0.441689
Hes1	0.441645
Scarb2	0.441642
Ccdc134	0.441609
Sox15	0.441451
Npr3	0.441392
Ndnf	0.44139
Emc4	0.441382
Smim14	0.441222
Pold4	0.441148
Apmap	0.441091
Rnf169	0.440971
Secisbp2	0.440933
Wbscr25	0.44092
Arhgap11a	0.440537
Slc25a19	0.440449
Ccdc57	0.440115
Spred1	0.439858
Anapc15	0.43968
Sox11	0.439673
Nkain2	0.439648
Aaas	0.439622
Ets2	0.439549
Abcc4	0.439285
Tbl1x	0.439066
Hand1	0.439051
Fn3krp	0.438945
Spock2	0.438877
Ispd	0.438659
Cnnm4	0.438463
Nrp2	0.438426
Rasip1	0.438273
Spg21	0.438159
Rcan1	0.438155

Sh3d21	0.438004
Eif4a3	0.437988
Chordc1	0.437969
Stat6	0.437922
Pcyox1l	0.437917
Cryba2	0.437901
Tpi1	0.437729
Leng9	0.437588
Zcchc7	0.437506
Ly6e	0.437156
Dlx3	0.436797
Rps6ka5	0.436741
Vps25	0.436658
Myoz1	0.436641
Calca	0.436507
Tpp1	0.436381
Sf3b5	0.436288
Zfp27	0.436273
Fam43a	0.436252
H2-T23	0.436154
Slc1a3	0.436059
Usp16	0.436043
Apob	0.436037
Ramp2	0.435938
Errfi1	0.435795
Slc35d2	0.435793
Ypel2	0.435787
Trit1	0.435771
Hn1	0.435699
Zfp335	0.435664
1810010H2	0.43553
Zfp78	0.435522
Gpr162	0.435509
Igfbp4	0.435416
Trib2	0.435254
Rasd1	0.435164
Nefl	0.434939
Enah	0.434908
Tm7sf2	0.434876
Mtmr4	0.434829
Cdkn1b	0.434592
Lmx1a	0.434582
Psmb10	0.434471
Dcaf17	0.43444
Bai3	0.434316
Plaa	0.434228
Slc43a1	0.433915

Capn7	0.433904
Csdc2	0.433885
Slc17a7	0.433817
Thumpd3	0.433724
Usp28	0.43372
Srrm4	0.433636
Prr7	0.43361
Fbxl22	0.433471
Moxd1	0.433428
Ddit4	0.433268
Mrm1	0.433177
Btbd9	0.433166
Ifrd2	0.433125
Mrpl52	0.433117
Rpp25	0.432957
Dlec1	0.432854
Ccng2	0.432736
Kidins220	0.432597
Elk3	0.432577
Cacna1e	0.43255
C87436	0.432325
App	0.432292
Acp5	0.4322
Hoxb5	0.432198
Gnai2	0.432046
Scyl3	0.432038
Hnrnpul1	0.432019
Tjap1	0.431998
Ptprcap	0.431856
Scube2	0.431532
Actg1	0.431285
Osbpl3	0.431259
Zfp532	0.431217
Stag1	0.430813
Cdc6	0.43077
Ecel1	0.430427
Fzd1	0.430353
Scube1	0.430303
Fam189b	0.430209
Pigv	0.430145
Peli1	0.429962
Adamts6	0.429902
Zfp568	0.429732
Chek1	0.429537
Rexo2	0.429509
Vax1	0.42941
Pax8	0.429309

Mzf1	0.429245
Pde5a	0.429197
Cish	0.428966
Wnt3	0.428893
Lrr1	0.428843
Lhx6	0.428809
Zfhx3	0.428728
BC029214	0.428635
Cox14	0.428447
Mrpl42	0.428362
Klhl13	0.428227
Tbc1d7	0.428172
Hes6	0.428167
Glra1	0.428138
Ano5	0.427992
Rap2b	0.427932
Sort1	0.427896
Ddx10	0.42778
Mtss1	0.427434
Bsdc1	0.427403
Acta1	0.427347
Ccpg1	0.427154
Tha1	0.426973
Sox21	0.426914
Pcyt2	0.426843
0610031J0	0.426734
Dpy30	0.426689
Gtf2h4	0.42664
Srebf1	0.42651
Htr1d	0.426504
Barx2	0.426502
Clasp1	0.426436
Rgs13	0.426351
Ppp1r15b	0.426343
Fras1	0.426247
Plxnb1	0.426146
Rfx5	0.42601
Amdhd2	0.425914
Caap1	0.425875
Mtmr10	0.425827
Eml5	0.425823
Panx1	0.425821
Ackr3	0.425768
Med16	0.425722
Rpl22	0.425686
Chid1	0.425586
Smg7	0.425443

Lrrtm1	0.425435
2810474O1	0.425367
Rel	0.425234
Pdp2	0.425168
Slu7	0.42516
Efcab10	0.425147
Znhit3	0.425142
Rgmb	0.425016
Sox6	0.424975
Mcrs1	0.424605
Stk33	0.424584
Morn4	0.424555
Dpagt1	0.424555
Plekho2	0.424518
Prcc	0.424421
Dmrt2	0.424363
Daxx	0.424223
Ormdl3	0.424167
Pip5kl1	0.424076
Cadm4	0.423991
Kirrel3	0.42369
Esrrb	0.423689
Fam13c	0.423678
Rgl3	0.423575
Phldb3	0.423559
Dennd6b	0.423547
9330182L0	0.423534
Myt1l	0.423483
Vgll3	0.423294
Podxl	0.42306
Hoxa13	0.423028
Rnf121	0.422849
Tle3	0.422691
Eva1a	0.422653
Ppt1	0.422545
Pelp1	0.422502
Fgd4	0.422313
Cox6b2	0.422065
Fam167b	0.421769
Cntnap5b	0.421757
Rtkn	0.421724
Xrn2	0.421664
Fads2	0.421654
Rcbtb2	0.421582
Brf1	0.421477
Arhgef1	0.421416
Rab3a	0.421374

Rad9a	0.421319
Poc1a	0.421308
Lpl	0.42064
Slc25a4	0.420583
Gyltl1b	0.420529
Nans	0.420333
Prdm14	0.420332
Gli3	0.4202
Plagl1	0.41996
Ublcp1	0.419941
Slc46a3	0.419926
Srp9	0.419914
Gal3st4	0.41973
Champ1	0.419621
Tspyl1	0.419355
Dpy19l4	0.419296
Hsf4	0.419114
Ttc8	0.419107
Trappc3	0.418938
Actb	0.418852
Ift74	0.418529
Pbx1	0.418507
Reep3	0.418486
Lrrc66	0.418471
Timm13	0.418394
Irgq	0.418298
Tkt	0.418238
Dcaf7	0.41806
Mrpl12	0.417967
Sox2	0.417857
Rnase13	0.417835
Plekha1	0.417777
Pitrm1	0.417732
Lamb1	0.417707
Car4	0.417293
Ryk	0.417192
Angel1	0.41719
Dleu2	0.41718
Apoc1	0.417157
Eif3f	0.417147
Gpr84	0.416941
Scarb1	0.416932
Slc46a1	0.416874
Tmem163	0.41686
Srgap1	0.416716
Jund	0.416676
Raf1	0.416607

Fam46a	0.416467
Col12a1	0.416464
Trim7	0.416444
Clcnkb	0.416372
Rftn1	0.416338
Trim37	0.416292
Ankrd42	0.41621
Gipc1	0.416176
Traip	0.416134
Gdf5	0.416078
Poc5	0.415945
Tmem37	0.415936
Tmem218	0.415883
Tmem97	0.415822
Rxfp3	0.415727
Usp53	0.415527
Rac3	0.415503
Fabp5	0.415418
Hoxa7	0.41535
Vgll2	0.415334
6530411M	0.415196
St5	0.414989
Fam83h	0.414959
Mmp15	0.414944
Cbfa2t2	0.41492
Adcy8	0.414893
Mif	0.414876
Snapc3	0.414841
Syngr1	0.414808
Rnf130	0.414801
Inip	0.414762
Gata6	0.414577
Eno1	0.414561
Baiap2l2	0.41445
Knstrn	0.414265
Sntg1	0.414124
Kif13b	0.413794
Pmf1	0.413523
Rasa3	0.413427
Agpat4	0.413353
Clp1	0.413294
Nudt16	0.413214
Minos1	0.41319
Vdac1	0.413186
Mink1	0.413162
Lztfl1	0.412904
Flywch2	0.412901

Arl6ip5	0.412866
Six1	0.412798
Zfpm2	0.412789
Prkab2	0.412656
9530068E0	0.412529
Cdk19	0.412244
Acat2	0.412233
Cenpo	0.412217
Hoxd3os1	0.41219
Tril	0.412165
Otx1	0.412162
Gnb1l	0.412062
Kcnf1	0.411999
Ppia	0.411952
Mfsd12	0.411907
Papd4	0.411885
Nr6a1	0.41184
Helt	0.411642
6330403A0	0.41163
Slc9a3r1	0.41161
Nxph4	0.411572
Alkbh5	0.411481
Polr3h	0.411107
Bpgm	0.411073
Gaa	0.411047
Cers6	0.410764
Hexim2	0.410547
Cox5b	0.410413
Usp48	0.410403
Foxc2	0.410389
Gsta4	0.410389
Fndc5	0.410263
Sdr39u1	0.410212
Slc25a17	0.409782
Clptm1	0.409775
Fabp3	0.409516
Chd1	0.409454
Ctsf	0.409351
Gabbr1	0.409327
Alms1	0.408928
Mtss1l	0.408925
Htr1a	0.408897
Lin28a	0.408844
Tuft1	0.408781
Gpx8	0.408462
Fgfr2	0.408408
Ndrg4	0.408286

Cabin1	0.408262
Ggt1	0.408214
Rab8a	0.408173
Bcl7b	0.408069
Klf5	0.407908
Gpkow	0.407803
Denr	0.407557
Wdr81	0.407501
Suclg1	0.407327
Poli	0.407198
Slc22a17	0.40709
2200002D(	0.407012
Isyna1	0.406989
Pigg	0.406901
Tmem242	0.406755
Sp5	0.406385
Hoxc10	0.406314
Fbxl19	0.406249
Uqcrc2	0.406131
AI413582	0.406127
Tfg	0.405867
Flcn	0.405803
Mthfs	0.405792
Tmem44	0.40579
Nfkbie	0.405762
Zbed3	0.405532
Ccdc67	0.40549
Foxp4	0.405465
Pik3r3	0.405371
Skap2	0.405365
Gabpb2	0.405345
Tbx2	0.405268
Wwox	0.405245
Gtse1	0.405228
Prr15	0.405212
Ist1	0.405181
Nrn1l	0.405147
Ptpn3	0.405091
Acot1	0.405043
Tefm	0.40503
Ppm1k	0.405003
Zfp623	0.40484
Mbp	0.404819
Foxd4	0.404695
Ntng1	0.404623
Dhx35	0.404559
Zscan2	0.404474

Uhrf1	0.404463
BC089491	0.404345
Itfg3	0.404257
Cyp24a1	0.404247
Slc26a10	0.404235
Appl2	0.404211
Rfx6	0.404155
Scaper	0.404137
Csde1	0.404135
Pigo	0.404031
Rimbp3	0.403984
Trdmt1	0.403889
Smurf2	0.40379
Hoxa1	0.403766
Plec	0.403758
Tpd52l2	0.403708
Rhot2	0.403567
Gpr153	0.403522
Smn1	0.403493
Rabac1	0.403454
Rbl2	0.403273
Eps15l1	0.403251
Mnx1	0.403129
Cecr5	0.402945
Ndn	0.402871
Pxmp4	0.402851
Ddost	0.402685
Rpl31	0.402671
Sv2b	0.40266
Cdkn1a	0.402639
Mavs	0.402514
Ggt5	0.402499
Arhgap20	0.402372
Nrd1	0.402196
Zfp593	0.40215
Vat1l	0.402017
Mfsd2a	0.401851
Bmpr1a	0.40185
Mmp9	0.401818
Nr1h2	0.401688
Tmco1	0.401469
Col27a1	0.401362
Bloc1s1	0.401324
Steap1	0.401188
Prima1	0.401077
Insig1	0.401062
Chst11	0.400961

Arhgef25	0.400952
Tpst2	0.400896
Wsb2	0.400711
Chrm1	0.400705
Cotl1	0.400612
Fstl1	0.400601
3110035E1	0.400417
Rpl19	0.400386
Cfl2	0.400353
Pcnp	0.400314
Ino80c	0.400285
Gareml	0.400268
Ddx24	0.400241
Prdm1	0.400119
Parp6	0.400109
Gpr6	0.399855
Pcnxl4	0.399845
Hsd17b12	0.399781
F2r	0.399737
Lmbrd1	0.399571
Sdhb	0.399531
Myl3	0.399505
Meis2	0.399482
Slc19a2	0.39941
Mtif3	0.399379
Wbscr16	0.399328
Tasp1	0.399239
Rrp1	0.399186
Syt5	0.398954
Zfp46	0.398949
Auh	0.398944
Smug1	0.398885
Cd47	0.398809
Cdt1	0.398805
Chic2	0.39872
Ocel1	0.398666
Stk32a	0.398653
Nr4a3	0.398647
Exd2	0.398535
Unc5b	0.398522
Als2cr12	0.398443
Abca1	0.398401
Zfp382	0.398379
Lpar4	0.398281
Tmem120b	0.398141
Trim59	0.397957
Pltp	0.397857

Kank2	0.397841
Ddx1	0.397782
Ddr1	0.397761
Zbtb32	0.397631
Fst	0.397622
Zmat4	0.39753
Hnrnpc	0.397504
Lyrm2	0.397419
Kifap3	0.39734
Mpped1	0.396962
Golga7	0.396833
Ephb3	0.396758
Lgr5	0.396744
Xylt1	0.396636
Ldha	0.396627
Drd4	0.396579
Adck5	0.396443
Esam	0.396313
BC024139	0.396298
Manf	0.396221
Grwd1	0.396204
Greb1	0.39609
Ank3	0.395844
Lrp4	0.395842
Tbl2	0.395705
Isy1	0.395527
Fkbp11	0.395484
Tmem230	0.39546
Tcf21	0.395447
Traf3ip2	0.395395
Pcmtd1	0.395333
Racgap1	0.395301
Ddx23	0.395205
Igsf9b	0.395086
Efhd1	0.394981
Foxj1	0.394798
Commd4	0.394701
Spdl1	0.394697
Crebrf	0.394669
Wasf2	0.394654
Hoxb6	0.39459
Ksr1	0.394524
Uprt	0.394524
Exo5	0.394417
Mapk1	0.394275
Snai3	0.394263
Irx4	0.394239

Pvrl2	0.394218
Def6	0.394211
Enpp1	0.39403
St3gal1	0.394007
Spry2	0.393873
Pdk1	0.393783
Nek3	0.393768
Cdk7	0.393684
Polg	0.393638
Myeov2	0.393613
Zfp580	0.393484
L3mbtl3	0.393458
Fjx1	0.393439
Gmppa	0.393389
Idi1	0.39337
Acrbp	0.393315
Ech1	0.393296
Nhej1	0.393293
Padi2	0.393243
Eif6	0.393155
Tubgcp5	0.393124
Ccbl1	0.39297
Pfkl	0.392937
Hist1h4d	0.392931
Igf2bp1	0.392895
Homer3	0.392875
Itgb1	0.392872
Cables2	0.392823
Atp6v1g1	0.392668
Dis3l2	0.392621
Zfp961	0.392563
Abhd10	0.392483
Myoz3	0.392368
Slc25a36	0.392272
Aadat	0.392269
Adamts10	0.392055
Gtpbp4	0.392042
Dcc	0.391946
Ophn1	0.391828
Ezr	0.391702
Grin2b	0.391693
Mad2l2	0.391576
Drd2	0.391567
Ppp2r5b	0.391564
Tuba1a	0.391513
Cspg4	0.391503
Mllt6	0.391223

Nes	0.391159
Upk3bl	0.391087
Tpm4	0.391049
Nup93	0.39099
Efcc1	0.390938
Gjb5	0.390656
Ppp1r14b	0.390649
Dgke	0.390482
Dda1	0.390478
Ap3b1	0.390416
Mettl7a1	0.390326
Wipf2	0.390282
Zfp113	0.390272
Lekr1	0.390125
Spen	0.390089
Atf4	0.390073
Fads6	0.389903
Acss3	0.389782
B3galnt2	0.389685
Tacr1	0.389624
Nrn1	0.389594
Rhobtb3	0.389534
Cnpy2	0.389456
Cdk20	0.389295
Selm	0.389259
Lrrk1	0.389183
Aaed1	0.389094
Xpo7	0.388993
Gtl3	0.388883
Psmb6	0.388812
Slc35d3	0.388716
Taok2	0.388693
Ywhah	0.388641
Eapp	0.388513
Pcolce	0.388402
St8sia4	0.388393
Plekhm3	0.388376
Senp2	0.388365
Arg1	0.388314
C1qtnf6	0.388214
Atp7a	0.388059
Mgat4b	0.388
Sh3rf1	0.38787
Igfbp5	0.387841
Lat	0.387762
Txnrd1	0.387744
BC068157	0.387737

Prr5l	0.387636
Igfbp6	0.387483
Stoml2	0.387446
Hoxa4	0.387237
Aars	0.387009
Ghitm	0.386957
Slc23a2	0.386815
Ebp	0.386805
Zfp462	0.386658
Pde10a	0.38665
Gfi1	0.386637
Gdi2	0.386634
Tmed9	0.386626
Ppp1r8	0.386422
1810011O1	0.386233
Rdm1	0.386196
Gcsh	0.386105
Zfp354a	0.386073
Neurog1	0.386036
Stk10	0.385939
Cilp2	0.385928
Wnk1	0.385927
Ctso	0.385893
Hist1h2bm	0.385796
Tanc2	0.385795
Nhlh1	0.385757
Gopc	0.385677
Gtpbp10	0.385653
Itgb5	0.385632
Lemd1	0.385588
Pabpn1l	0.385459
Heatr1	0.385374
Zfp84	0.385341
Pet112	0.385307
Nedd4	0.385136
Fam178a	0.385028
Lrrn2	0.38497
Cdr2l	0.384885
Ldhb	0.384814
Zfp871	0.384712
Anxa5	0.384691
Tmem161b	0.384522
Lemd2	0.384457
Nek9	0.384397
Akap8	0.384253
Prrxl1	0.384196
Cpsf6	0.384157

Cdk5rap3	0.384122
Tox3	0.384016
Alox12b	0.384004
Srebf2	0.383976
Epha10	0.383974
Crkl	0.383881
Ints4	0.383815
Snx27	0.38374
Mterfd2	0.383708
Esrp1	0.383668
Pfdn5	0.38366
Zfp26	0.383479
Tmco6	0.383473
Kctd17	0.383463
Tmem87b	0.383425
Myh9	0.383394
Gm5607	0.383337
Prdm6	0.383284
Rnf126	0.383226
Pknox2	0.383114
Ucp2	0.383067
Dscr3	0.382985
Eif3h	0.382948
Rps6ka2	0.38279
Mri1	0.382784
Mylk2	0.382759
Slc1a5	0.382728
Triobp	0.382696
Cdpf1	0.382674
Zfp933	0.382525
Egr2	0.382524
Catsper1	0.382511
Tpgs1	0.382507
Snapc1	0.382444
Hip1	0.382429
Asb16	0.382297
Otop3	0.38223
Tfap4	0.382171
Smarcd1	0.382154
1600002K0	0.382034
Mad1l1	0.381979
Atox1	0.381834
Prr5	0.381712
Crygd	0.381579
Etv5	0.381482
Egln2	0.38147
Dcaf8	0.381405

Zfat	0.381355
Ankle1	0.38134
Nxph3	0.381313
Flnc	0.381292
Plxdc1	0.381184
Cyp26c1	0.381175
Tex9	0.381097
Slit3	0.381071
Slc12a7	0.381025
Fscn1	0.380907
C1ql4	0.380801
Hmbs	0.380772
Rab11fip4	0.380676
Atf7	0.380576
Fbln1	0.380404
Rasl11a	0.380241
Dnajc6	0.380191
Arl10	0.380136
Cdc42se2	0.380013
Rem2	0.379968
Trappc6b	0.379922
Cuedc2	0.379915
AI118078	0.379857
Ddx19a	0.379628
Sfxn3	0.379518
Lsm2	0.379376
Tnnt2	0.37934
Ankrd26	0.379261
Kif13a	0.379256
Wdr62	0.379208
Tmem126b	0.379135
1700008J0	0.379129
Xkr5	0.379044
Map2k2	0.37899
Slc1a4	0.378905
Map2k1	0.378886
Dapk1	0.3788
Mterf1a	0.378765
Sema4b	0.378697
Rab39	0.378623
Naca	0.378493
Gsc	0.378397
Atoh8	0.378388
Hand2	0.378297
Clta	0.378281
Anln	0.378257
Zfp422	0.378122

Nkx2-5	0.377988
Asic1	0.377959
Galnt16	0.377883
Rin1	0.377774
Galt	0.377751
Sp9	0.377731
Casc4	0.377702
Elovl4	0.377666
4931414P1	0.37766
Dchs1	0.377555
Ppm1h	0.377475
Shb	0.377418
Scrt1	0.377372
Nqo2	0.377276
Pigyl	0.377174
Cdh15	0.377151
Upf2	0.377133
Zfp14	0.377125
Heg1	0.377113
Sdf2l1	0.377112
Galr2	0.376995
Zfand3	0.376918
Pdxp	0.376869
Sass6	0.376844
Aldh1a2	0.376796
Arhgef12	0.376741
Ap5z1	0.376694
Lhx5	0.376689
Arfgap2	0.376614
Gpt	0.376412
1190002N1	0.376208
Cdk3-ps	0.376162
Arf2	0.376157
Zfand4	0.376107
Sp4	0.376104
Rapgefl1	0.376051
10-Mar	0.375952
Efna2	0.375917
Cap1	0.375908
Ccnd2	0.375802
Rps27l	0.375801
Plcl1	0.375761
Pth2	0.375629
Atg10	0.375617
Car10	0.375604
Acvr2a	0.375421
Dlx2	0.375302

Kdelr2	0.375187
Spata20	0.375182
Cask	0.375058
Pef1	0.375027
Bmp4	0.374942
Dbx2	0.374942
Slc7a7	0.374922
Arl4a	0.374916
Pgls	0.374852
Baiap2	0.374745
Rgl2	0.374728
Rai14	0.374724
Grm4	0.374649
Sac3d1	0.374593
Rbm38	0.37453
Acyp1	0.374523
Hnrnpll	0.374522
Mapt	0.374286
Pknox1	0.374278
Slc32a1	0.374221
Rab5b	0.374208
Cant1	0.373993
Hpgds	0.373944
Ipo13	0.37389
Apeh	0.373881
Srrm1	0.373769
Krtap10-4	0.37355
Mrps25	0.373547
Neurl3	0.373457
Arhgap8	0.373401
Ptger4	0.373359
Sepw1	0.373352
Xrn1	0.373329
Wbscr17	0.373324
Baz2b	0.373243
Ept1	0.37324
Parp1	0.373204
Arhgef10l	0.373187
Hbp1	0.373103
Echdc3	0.373073
Zfp667	0.372882
Msl1	0.372881
Nf1	0.37282
Hsd11b2	0.372752
Mtfr1	0.372749
Gypa	0.372711
Sox9	0.372636

Rbx1	0.372593
Ifitm1	0.372555
H2-Q6	0.372488
Slc25a20	0.372444
Rgs16	0.372384
Egflam	0.372362
Brip1	0.372348
Wdr37	0.372218
Irf9	0.372191
Itpr1	0.372087
Tnfrsf21	0.372029
Fzd9	0.371985
Fzd3	0.371979
Dtnbp1	0.371975
Rps21	0.371951
Slc16a9	0.371728
Atp1b2	0.371641
Eif4e3	0.371615
Rnf32	0.371512
A130010J1	0.371454
Nell2	0.371451
Mpped2	0.371431
Tspan14	0.371364
Vps13b	0.371332
Sstr1	0.371324
Fbxo22	0.371226
Sik1	0.371212
Timm50	0.3712
Mknk2	0.371159
Crabp1	0.371131
Gdf15	0.370976
Tert	0.370889
Doc2a	0.370784
Gm608	0.37076
Stk40	0.370751
Sesn1	0.370742
Fam149a	0.370713
Pomt1	0.370706
Ube2l6	0.370695
Cstad	0.370625
Tmem123	0.3706
Arl6	0.370543
Fbxo46	0.370468
B9d2	0.370421
Grem1	0.370381
Fadd	0.370377
Il1r1	0.370277

Slc13a5	0.370243
Ppap2c	0.370186
Zdhhc9	0.370085
Itpripl1	0.37003
Hsdl2	0.36989
Cirbp	0.369836
Dars	0.369622
Aldh3a2	0.369586
Slc25a48	0.36957
Kdr	0.369529
Ndrg2	0.369495
Ift81	0.369467
Sc5d	0.369385
Eif2b5	0.369298
Lmo4	0.369248
Fam198b	0.369123
Bcl6	0.369054
Mrps16	0.368969
Akap12	0.368905
Pik3r1	0.368892
Slc2a8	0.368888
Rtfdc1	0.368864
Psmg4	0.368837
Plk2	0.368697
Arc	0.368619
Prkg1	0.368586
Wif1	0.36857
Cbs	0.368496
Amh	0.368459
Chchd2	0.368452
Dtd2	0.368417
Hspbp1	0.368336
Prrc2a	0.368131
Fyco1	0.368119
Fzd7	0.36791
Uqcrfs1	0.36788
Mettl21a	0.367822
Tjp2	0.367812
Map2k5	0.367777
Camk2d	0.367703
Oacyl	0.3677
Spsb2	0.367683
Odf3l2	0.367676
AI462493	0.36763
Psmd8	0.36752
Fgf10	0.367486
Nmt1	0.36748

Tmem50a	0.367445
Fzd2	0.367389
Dyx1c1	0.367275
4833439L1	0.3672
Ifi30	0.367193
Klhl30	0.367079
Apoe	0.367027
Mrpl45	0.366981
Terf1	0.366973
Efr3b	0.366922
Ripply3	0.366892
Mc5r	0.366545
Afap1l1	0.366472
Gtf3c6	0.366385
Mob3b	0.366376
Negr1	0.366364
Fam129b	0.366326
Acvr1	0.366258
Tmem106c	0.366162
Ralgds	0.36601
Acp2	0.366003
Myrip	0.365954
Tmem260	0.365888
Thsd4	0.365865
Prickle1	0.365858
Rab6a	0.365845
Fam83a	0.36583
Crmp1	0.365795
Gmfb	0.365634
Atp6v0a2	0.365631
Athl1	0.365596
Nudt12	0.365572
Pdlim1	0.365457
Cdadc1	0.365381
Nat10	0.365346
Dmap1	0.365334
Lnpep	0.365256
Klf7	0.365194
Edc4	0.365173
Camk1g	0.365163
Hpse2	0.365116
Tmem191c	0.365084
Ulk3	0.365032
Psmg1	0.365005
Sh3bp5l	0.36494
Cpe	0.364897
Zfp868	0.364756

Hlf	0.364737
Slc30a3	0.364661
Lpar2	0.36465
Engase	0.364626
Nicn1	0.364571
Slc15a1	0.364552
Efs	0.36455
Kcng4	0.364511
Nfil3	0.364277
Atf1	0.36424
Rap2c	0.364128
Ifngr1	0.364077
Polq	0.363897
Fance	0.363825
Snn	0.363823
Bicd1	0.363816
Rps7	0.363638
Btg1	0.363616
Lta4h	0.363555
Pcf11	0.363552
Aatf	0.363534
Ccdc150	0.363421
Gpr31b	0.363387
Dlgap5	0.363219
Tmem132a	0.363197
Nck1	0.363079
Sh3glb2	0.363059
Dzip1	0.362957
C2cd2	0.362927
Tbcc	0.362898
Cnbp	0.362878
AW146154	0.362854
Nub1	0.362815
Yae1d1	0.362801
Rnft1	0.362799
Slc2a1	0.36277
Prkd2	0.362726
Klhdc9	0.362708
Hdac11	0.362535
Prpsap1	0.362467
Gm5544	0.362465
Cacng4	0.362432
Dnajc22	0.36243
Cystm1	0.362322
Nptn	0.362294
Ldlrap1	0.362229
Lsm8	0.362167

Hmx3	0.362127
Strip1	0.362049
Itpr3	0.362038
Glul	0.362035
Tle4	0.362003
Rsl1d1	0.362001
Alg6	0.361978
Pax7	0.361971
Asic4	0.361777
Tnfaip8l3	0.361756
Ptchd2	0.361751
Rbm26	0.36172
Kif26a	0.361705
Gatsl2	0.361685
Gpr12	0.361623
Chst12	0.361622
Rnh1	0.361601
Hmox1	0.361538
Fam117a	0.361513
Snrpg	0.36146
Ntrk3	0.361409
1110004E0	0.361394
Hoxc8	0.361286
Malat1	0.361016
Cars2	0.360827
St6gal1	0.360815
Itga9	0.360734
Zfp191	0.360565
Atf2	0.360551
Aspg	0.360473
Braf	0.360457
Acbd6	0.36043
Ssr3	0.360425
Lmln	0.360346
Bola1	0.360327
Fnbp4	0.360304
Commd3	0.360284
Zfp384	0.360224
Sost	0.360169
Atp1a1	0.360088
2310011J0	0.360065
Slc26a6	0.360037
Amn1	0.359857
Frs2	0.359849
Armc8	0.359808
BC026585	0.359785
Cks2	0.35977

Trpm7	0.359759
Rnf152	0.359723
Npdc1	0.359695
Ppp1r11	0.359674
Mapk15	0.359557
Epb4.1l4a	0.359477
Usp36	0.35944
Map1lc3b	0.359386
Ccdc8	0.359357
Zdhhc2	0.359352
Zfp536	0.359338
Fh1	0.359086
Elf3	0.359048
Yeats4	0.358972
Nfatc4	0.358915
Rapgef6	0.358831
Acpl2	0.358803
Tspyl4	0.358798
Rrm1	0.358698
Agfg2	0.358602
Cdyl	0.358564
Asap1	0.358546
Parvb	0.358511
Ndufc2	0.358488
Nod2	0.358475
Zfp3	0.358458
Ep300	0.358447
Ganab	0.358439
Dact3	0.358416
Anp32e	0.358312
Zfp867	0.358278
Terf2	0.35805
Nhp2	0.357964
Wnt2	0.357902
Ano6	0.357837
Rrad	0.357791
Ust	0.357774
Got1	0.357626
Fndc3a	0.357615
Slc50a1	0.357589
4930402H2	0.357564
Mrps36	0.357527
Hsp90ab1	0.357451
Naprt1	0.357438
Pgm1	0.357374
Snf8	0.357257
Hspa13	0.35724

Zmynd11	0.357216
Vps26a	0.357208
Sh3bgr	0.357186
Thnsl2	0.357156
Wnt7a	0.357154
Actr6	0.357145
Ift46	0.357137
Gck	0.357006
Gjb4	0.356935
Ccdc174	0.356928
Gnb2	0.356922
Samd12	0.356842
Epas1	0.35682
Tbc1d30	0.356727
Mfng	0.356617
Setbp1	0.35656
Gramd1a	0.356496
Tmem192	0.35638
Optc	0.356357
Necab1	0.356193
Hs6st1	0.356081
Gas2l2	0.356057
Ebag9	0.355917
Kcnq4	0.355901
Rnf8	0.355867
Dio1	0.355837
Kcne1	0.355822
Snap25	0.355736
Cox8a	0.355637
Cfd	0.355612
Fyn	0.355557
Adrb3	0.355557
Sst	0.355534
Rsph6a	0.355495
Myog	0.35546
Rnf7	0.355401
Kif1b	0.355313
Zdhhc14	0.355274
Celf2	0.355226
Tpm1	0.35519
l7Rn6	0.35512
Cep250	0.355098
Ctnnd1	0.354979
Tmem41b	0.35496
Ptpn23	0.354919
Lmbr1	0.354835
Sbk1	0.354824

Senp7	0.354756
Gdf7	0.354684
Smarcb1	0.354611
Zfp804a	0.354488
Gkap1	0.354446
Brwd1	0.354391
Rrm2b	0.354344
Phactr2	0.354303
Adcy3	0.354187
Zcchc8	0.354174
Arhgdia	0.35417
Slc38a7	0.35417
Smim11	0.354152
Mdga2	0.354064
St6galnac2	0.353988
Sec23ip	0.35397
Phb	0.353962
Klhl1	0.353877
Ticrr	0.353841
Ccdc130	0.353823
Bcl2l2	0.353723
Lhx4	0.353636
Puf60	0.353626
Cmtr2	0.353531
Ndufa12	0.353512
Skor1	0.353507
Dnajb9	0.353455
Shroom3	0.353452
Wscd1	0.353293
Raly	0.353121
Pin4	0.352978
Mrpl19	0.352972
Eif4a1	0.352922
Hspg2	0.352894
Cyth1	0.352828
Hpcal4	0.352752
Wnt10a	0.352711
Ccdc30	0.352651
Ssh2	0.352646
Hoxb3	0.352614
Sh3bp5	0.352552
Eif3l	0.352489
Slc2a3	0.352474
Mdn1	0.352317
Exoc7	0.352002
Ube2t	0.351896
Dhx16	0.351828

4930483J1	0.351817
Trim11	0.351757
Srp68	0.3517
Prkar2a	0.351658
Cox20	0.351559
Atad2	0.351441
Rarg	0.351432
Bai1	0.351325
Tfam	0.351261
Cpd	0.351255
Rad51	0.351154
Sorbs3	0.351134
Cpeb4	0.351095
Kdm5a	0.351004
Usf1	0.350963
E430018J2	0.350917
Wdr6	0.350803
Uimc1	0.350731
Zfp346	0.350716
Wnt6	0.350707
Foxs1	0.350649
Mocs2	0.350547
Cyb5r3	0.350517
Vstm2l	0.350508
Spryd3	0.350488
Brinp2	0.350459
Rhobtb2	0.350357
Spock1	0.350288
Snrnp35	0.350205
Atxn3	0.350204
Dscc1	0.350162
Gcgr	0.350158
Slc38a8	0.350058
Bbs2	0.350057
Use1	0.350008
Alx3	0.349984
Mat2a	0.349977
BC100451	0.349946
Afg3l1	0.349942
Sf3b4	0.349925
Rnf26	0.349847
Hdac2	0.349834
Rfx7	0.349699
Dnajb8	0.349648
Cyp27b1	0.349642
Laptm4a	0.349529
Spire2	0.349433

Rcsd1	0.349388
Bsx	0.349371
Acy1	0.349362
Nphp3	0.34935
Myo7a	0.349302
Hcn3	0.349295
Ddx39b	0.349239
Tbc1d25	0.349221
Derl3	0.349106
Cenpu	0.349081
Lrrc51	0.349053
Fgf15	0.349027
Nubp1	0.348998
Cdh10	0.348841
Zfp629	0.34884
Acvr2b	0.348661
2210016L2	0.348579
Ccdc106	0.348575
Dnajb12	0.3485
Dek	0.348375
Ing2	0.348371
Smtn	0.348275
Stab1	0.34827
Rnf145	0.348217
Pyroxd1	0.348185
Map7	0.348179
Tanc1	0.348091
Pdss2	0.347943
Slc6a6	0.347882
Trim28	0.347825
Eif2ak3	0.347784
Slc26a4	0.347673
Ckb	0.347645
Adamts15	0.34764
Tmem235	0.347571
Gosr1	0.34757
Maff	0.347558
Lrig1	0.347547
Rbm25	0.347416
Tex30	0.3474
Lancl2	0.347353
Atp6v0c	0.347352
Stam2	0.34734
Cxcl14	0.34732
Opa1	0.347239
Cdkn2aip	0.34718
Sumo3	0.347073

Rnf6	0.347058
Spaca4	0.347053
Cyp1b1	0.347044
Unc13a	0.347012
Igfbp3	0.346976
Scrt2	0.346897
Smim20	0.346865
D230025D1	0.346835
Gnb5	0.346815
Dstyk	0.346745
Gabarapl2	0.346704
Pars2	0.346437
Cd97	0.346378
Zscan12	0.346294
Cntn6	0.346217
Zfp597	0.346215
Tspan13	0.346211
Tmprss6	0.346207
Fxyd6	0.346143
Rgs7bp	0.346119
2900005J1	0.346063
Nradd	0.346035
Kdelr1	0.346017
P4hb	0.345852
Olfm2	0.3458
Mta2	0.345795
Arvcf	0.345781
Serhl	0.345719
Gng8	0.345703
Leo1	0.345641
Smc2	0.345603
Yipf1	0.345572
Ppil2	0.345567
Vegfa	0.345502
Kansl1	0.345377
2310061I0	0.34533
Clpx	0.345315
Zfp266	0.345297
Gas2l3	0.345295
Tprgl	0.345253
Kdm2b	0.345222
Herpud1	0.34516
Trmt61b	0.345159
Bin1	0.345124
Trappc5	0.345121
Klhdc10	0.345089
Lst1	0.344991

6430548M	0.344973
Akap8l	0.344961
Myo5a	0.344914
Tspan5	0.344912
Smtnl2	0.344904
Tmem115	0.344891
Gm16515	0.344782
Shc3	0.344755
Mettl17	0.344672
Pde12	0.344636
Amacr	0.344627
Lpin1	0.344552
Tnk2	0.344407
Crlf1	0.344337
Gnptab	0.344335
Rpf2	0.344331
Cldn15	0.344327
Bard1	0.344275
Ost4	0.34417
Syt3	0.34414
Pitx1	0.344002
Fgf12	0.343988
Pola1	0.343981
Fancg	0.343961
Stil	0.343865
Ywhaq	0.343837
Calcoco1	0.343786
Snx19	0.343739
Ccdc104	0.343692
Cbr4	0.343673
Hibch	0.343669
Mesdc2	0.343617
Hhip	0.34358
Cnot4	0.343554
Rsu1	0.343514
Arx	0.343502
Adrm1	0.343499
Itpkc	0.343393
Wiz	0.343389
Rassf4	0.343328
Ubtd2	0.343247
Zc3h18	0.34319
Lztr1	0.343149
Fgfr1	0.34307
Cox6a1	0.343048
Fam98a	0.342983
Ager	0.342935

Nsf	0.342771
Kansl2	0.342757
1700037H(	0.342728
F3	0.342724
Fam131a	0.342711
Sox12	0.342687
Gmip	0.342674
Irak1bp1	0.342667
Saal1	0.342665
Atad5	0.342589
Enc1	0.342563
Syt6	0.342428
Creb3l2	0.342393
Kcns2	0.342262
Dner	0.342094
Cluh	0.342094
Enpp4	0.342076
Grifin	0.342066
Slc35b1	0.342059
Sgca	0.342051
Zfyve16	0.34202
Tmem63a	0.341977
Decr2	0.341909
Lrif1	0.341872
Csrp2	0.341809
Msln	0.341754
Mapkbp1	0.341654
Aurkaip1	0.341594
Egfl8	0.341592
Cgrrf1	0.341592
Rasl11b	0.341482
Zdhhc20	0.341476
Sec14l1	0.34146
Phlda1	0.341431
Bnip3	0.341418
Zscan18	0.341365
Riok2	0.341304
Gucy2e	0.341297
Zfp184	0.341276
Ap1m1	0.341172
B4galnt4	0.341119
Hdac10	0.341111
Nek2	0.341105
Nr1d1	0.340962
Zdhhc5	0.340955
Arl2bp	0.340948
Celf4	0.34091

Psma1	0.340808
2810403A(	0.340752
Afap1	0.340742
Nhsl1	0.340722
Alk	0.340655
Snupn	0.340615
Raet1a	0.3406
Fut10	0.340593
Nagk	0.340585
Slc16a3	0.340545
Ccnb1	0.34054
Commd5	0.340508
Rsad1	0.340465
Cmtm6	0.340376
Katna1	0.34027
H2-DMa	0.340237
Cox7a2	0.34023
Tfap2d	0.340216
Scamp5	0.340132
Ptgds	0.340068
Baiap2l1	0.340059
Inf2	0.339987
Nvl	0.339973
Ahdc1	0.339965
Sipa1l1	0.339941
Sertad2	0.339911
Tufm	0.339909
Dpp3	0.339878
Pdk3	0.339847
Extl3	0.339823
Smarcc1	0.33978
Eif4e2	0.339737
BC031181	0.339733
Cep85	0.339555
Cep350	0.339535
Foxred2	0.339423
Etf1	0.339409
Kdm7a	0.33925
3632451O(	0.33921
Cdc34	0.33918
Golim4	0.339129
Scaf11	0.339103
Tmem132c	0.339074
Klhdc8a	0.339026
Fam64a	0.338944
Zc3hav1	0.338936
Actr10	0.33883

Fbxl3	0.338693
Tnfaip3	0.338526
Gzmm	0.338471
Pvrl3	0.33844
B3gntl1	0.338373
Frem2	0.338356
Sec61g	0.338329
Acadm	0.338308
Iffo1	0.338279
Lrrc4c	0.338263
1700049G1	0.338197
Phyh	0.338175
Pcm1	0.338109
Erbb2	0.338106
Ptov1	0.338067
Islr	0.337967
Cdx2	0.337846
Zfr	0.337694
Klf14	0.337679
Ctcf	0.337662
Nfkbia	0.337654
Barhl2	0.337399
Sfn	0.337361
Frg1	0.337294
Cyp2u1	0.337216
Gins2	0.337215
Kat8	0.337206
Hsd17b1	0.337157
Mdfic	0.337027
Cse1l	0.337014
Eif4g3	0.33697
Zfp354b	0.336959
Snrpb	0.336831
Blmh	0.336664
B3gat2	0.336653
Coro7	0.336606
Slc48a1	0.336537
Elovl7	0.336504
Gbe1	0.33647
Tomm5	0.336458
Ggta1	0.336444
Akap1	0.336407
Gng7	0.336344
Ywhag	0.336334
Ttc33	0.336327
Haus4	0.336227
Pfdn4	0.336193

Nudt13	0.336188
Pcna	0.336176
Glb1	0.336175
BC027231	0.336151
Tmx3	0.336135
Gid8	0.336133
Slc4a8	0.336056
Grik1	0.336053
Ppp1r3b	0.336006
Slc38a1	0.335982
Nid2	0.335973
Gpi1	0.335956
Hs3st1	0.335943
Kctd1	0.335926
Rps15	0.335912
Stk30	0.335846
Wdyhv1	0.335829
Arhgap9	0.335825
Stam	0.335798
Eva1c	0.335622
Ndufb7	0.335534
Abhd6	0.335504
Pold1	0.335493
Fam46c	0.335484
Fam160a2	0.335475
Park7	0.335463
Herc1	0.335393
Osbpl9	0.335332
Cdca7l	0.335212
Pip5k1a	0.335156
Tfr2	0.335151
Abhd4	0.335096
H2-K1	0.335089
Ndel1	0.335087
Got2	0.335065
Pdcd7	0.335064
Clcnka	0.335044
Dhx9	0.335037
Ppm1d	0.334932
Slc25a24	0.334919
Ildr2	0.334848
Eif5a	0.334838
Lasp1	0.334814
Afg3l2	0.334747
Grin2d	0.334653
4930451I1	0.334644
Zfp174	0.334631

Cnot10	0.334627
Zfp292	0.334617
Tmem59l	0.334604
Kcnk15	0.334571
Rab11fip3	0.334555
Fgf17	0.3345
S1pr2	0.334443
Slc4a11	0.334424
Fbxo27	0.334412
Gpc3	0.334369
Ptprq	0.334342
Rtn3	0.334299
Gclc	0.334274
Pdlim7	0.334228
Tubb4a	0.334206
Pacsin3	0.334185
Sapcd1	0.334172
Chga	0.334139
Pygo2	0.334062
Ankrd28	0.334018
Dnlz	0.333972
Tra2b	0.333824
Csrnp3	0.333809
Chrna5	0.333778
Fsd1	0.333678
Pear1	0.333603
Mpp3	0.333495
Nhlh2	0.333468
Ccdc97	0.333443
Cd248	0.333441
Zfp809	0.33344
Fancc	0.333405
Cercam	0.333378
Pfn2	0.333311
Cep41	0.333283
Bcl11b	0.333263
Airn	0.333216
Nckipsd	0.333171
Mx2	0.333118
Prrc2c	0.33311
Rae1	0.333109
Zswim4	0.333089
Ctxn1	0.332989
Orai1	0.332965
Thra	0.332921
Hk2	0.332911
Tmed8	0.33288

Lca5	0.332845
Elovl5	0.332802
Nhp2l1	0.332798
Ifitm5	0.332778
Tra2a	0.332718
Ubap2	0.332686
Mms22l	0.332656
Etnk2	0.332623
Rab8b	0.332524
Tnfrsf1a	0.332488
Irf2	0.332443
2700029M	0.332395
Ripk1	0.332383
Col14a1	0.332326
Il11ra1	0.332311
Pxdn	0.332271
Actr3	0.332217
B830017H(	0.332202
Gabrg2	0.332176
Tspan3	0.332173
Col23a1	0.332151
Amer1	0.332125
Cx3cl1	0.332029
Uvrag	0.332008
Zfp521	0.331987
Axin1	0.331963
Rasgef1a	0.331808
Zfp663	0.33177
Asl	0.331751
Soga2	0.331728
Rbpms2	0.331722
Gipr	0.331718
Erap1	0.331687
Mrps22	0.331681
1700001O2	0.331568
Glg1	0.331531
Cenpe	0.331462
Cmss1	0.331424
Capn2	0.331252
Socs2	0.331241
Znhit6	0.331218
Dnajc5	0.331193
Nedd1	0.331183
Pfn3	0.331162
Sqle	0.331159
Pycrl	0.331108
Irx1	0.331062

Shd	0.331048
Dlst	0.330984
Irx6	0.330965
Pafah2	0.330934
Grb7	0.33089
Paqr4	0.330842
Tgds	0.330837
Fam103a1	0.330813
Hk1	0.330806
Ptf1a	0.330804
Ube2d1	0.330713
Ccdc50	0.330703
Megf9	0.330677
Fam76a	0.330568
Ppil1	0.330554
Hnf1a	0.33051
Fam171a2	0.330496
Vars	0.33036
Zfp35	0.330329
Pdgfra	0.3303
R3hcc1	0.330237
Rqcd1	0.330115
Alg10b	0.330081
Aen	0.330017
Suds3	0.330013
Cdc42ep1	0.32998
Spcs3	0.329921
Vars2	0.329884
Tango2	0.329786
Tctn2	0.329651
Srxn1	0.329621
Cog1	0.329595
Cnih3	0.329537
Slc9a1	0.329397
Cmtm8	0.329382
1700017B0	0.329373
F2rl1	0.329329
Mex3b	0.3293
Vimp	0.329223
Akap13	0.329217
Slain2	0.32919
Unc13d	0.329152
Ctnnd2	0.329125
Sfxn1	0.329124
Spop	0.329111
Eif4h	0.32911
Vash1	0.329041

Nktr	0.329035
Magi3	0.329027
Rplp0	0.328992
Plcb1	0.3289
Nom1	0.328858
Galnt10	0.328828
H2afv	0.328792
Ing3	0.328742
Kpna6	0.328713
Snx4	0.328689
Zbtb7b	0.328613
Gas2l1	0.328598
Tep1	0.32855
Kctd12	0.328512
Prodh	0.328488
Pitx2	0.32846
Sim2	0.328424
Zfp260	0.32842
Adam12	0.328411
Top2a	0.328363
Zfp160	0.328355
Ifitm3	0.32834
Psors1c2	0.32824
Cttnbp2nl	0.328153
Cd164	0.328145
Ppard	0.328115
Sap25	0.328073
Ptprn2	0.328066
Dstn	0.328066
Enox1	0.328064
B3galt4	0.328003
Fbrs	0.32786
Akna	0.327798
Slc39a8	0.327738
Mtmr9	0.327704
Sult2b1	0.327695
Sall2	0.327669
Atp5g3	0.327633
Slc35e4	0.32762
Dnajc13	0.327601
Cln6	0.327528
Polr2j	0.327518
Zswim8	0.327492
Slc6a20a	0.327364
11-Sep	0.327352
Zfp768	0.327329
Fgf5	0.327256

Adamts1	0.327156
Lsp1	0.327113
Actr3b	0.327105
Stx18	0.327042
Slc39a14	0.326987
Sec22c	0.32698
Asah1	0.326971
Get4	0.326931
Cthrc1	0.326918
Lbh	0.326857
Rnf168	0.326848
Kctd21	0.326817
Impdh1	0.32676
Chrdl2	0.326675
Sgk1	0.32666
Mc1r	0.326656
Fdxr	0.326648
Pdik1l	0.326499
Hn1l	0.326498
Btbd16	0.326487
Fam98b	0.326458
Tarsl2	0.32643
Abcd3	0.326375
2010015L0	0.326263
Dlg1	0.326231
Dcxr	0.326189
Cdv3	0.326172
Sox5	0.326172
Pmepa1	0.326104
Rasl10a	0.32601
Ints3	0.326004
D430041D(	0.326
Dusp9	0.325976
Rrp15	0.325945
Fgr	0.32588
Lace1	0.32588
Igfals	0.325873
Myo1e	0.325849
Klhdc7a	0.325829
Zfp64	0.325818
Sema3c	0.325736
Sdc4	0.32568
Stk11	0.325657
Eef2k	0.325645
Sv2c	0.325545
G3bp1	0.325334
Hsd3b7	0.325305

Ercc5	0.325251
Timmdc1	0.325177
Greb1l	0.325167
Madcam1	0.325141
Trp53inp2	0.325139
Slc37a4	0.325058
Nsg1	0.325048
Chmp4b	0.32501
Trp53inp1	0.324897
Rab3gap2	0.324859
Ccng1	0.324805
Sec23b	0.32472
Srsf12	0.324571
Zfp213	0.324468
H2-Q8	0.324414
Pkp1	0.324407
Rbm45	0.324395
Nsun3	0.324375
Atxn1	0.324326
Atp5g1	0.324323
Bcl9l	0.324244
T	0.324234
Ndufb4	0.32417
Taf15	0.324122
Uchl1	0.324011
Rragc	0.323997
Cxadr	0.32399
Pop5	0.323828
Adora1	0.323783
Antxr2	0.32378
P2rx4	0.323772
Arfip1	0.323748
Ptk2b	0.323738
Elac2	0.32372
Prex1	0.323698
Ppp3r1	0.32368
Bub3	0.323655
Fdps	0.323642
Aprt	0.323622
Ccar1	0.323619
Mertk	0.32349
Eif3b	0.323436
Pank1	0.323415
Rassf3	0.323393
Aasdh	0.32337
Opcml	0.3233
Amotl1	0.323262

Tet2	0.323257
Fli1	0.32321
Syndig1	0.323145
Fam181b	0.323144
Ybx1	0.32305
Lfng	0.323044
Nacad	0.322981
B330016D1	0.322926
Kdsr	0.322868
Hes2	0.322829
Tcf7l2	0.322811
Lamtor4	0.322779
Vwa8	0.322771
Ocln	0.322675
Bcat2	0.322645
Zfp639	0.322585
Idua	0.322559
Nuak1	0.322469
Tbxa2r	0.322445
Rprd1b	0.322442
B230118H0	0.322425
Capn13	0.3224
Arhgap30	0.322277
Hax1	0.322261
Il27	0.322157
Slc35d1	0.32209
3110062M	0.322084
Slc6a2	0.322084
Pik3c2b	0.322076
Gdf2	0.322039
Vps11	0.321959
Zfp90	0.321949
Grip2	0.321912
Lsamp	0.321908
Ncoa3	0.321887
Rfx2	0.321849
Coq7	0.321629
Rbfox3	0.321626
Dck	0.321604
Lbr	0.3216
Tusc2	0.321491
Dpysl5	0.321451
Creb1	0.321419
Ptprs	0.32138
Mex3c	0.321289
Gprc5a	0.321282
Prkce	0.32124

Zfp108	0.321181
Copz1	0.321095
Lhx2	0.321093
Rltpr	0.320949
Pgf	0.320889
Dhx34	0.320868
Cacng1	0.320793
Sdk2	0.32076
Fosb	0.320759
Hoxd12	0.32066
Wtap	0.320639
Mtus1	0.320619
Pelo	0.320523
Mtap7d3	0.320499
Rtkn2	0.320406
Arf4	0.320398
Tfip11	0.320225
Gtf2e2	0.320215
Esyt2	0.320202
Stmn1	0.320182
Nme2	0.320124
Tox	0.319982
Leprot	0.319958
Phldb2	0.31995
Fam214b	0.319937
Wwc2	0.319908
Ak2	0.319878
Zfp81	0.31983
Comtd1	0.319823
N6amt2	0.319754
Jup	0.31975
Cox7a1	0.319734
Nudt9	0.319729
Sc4mol	0.319654
Surf6	0.319651
Trp53rk	0.319554
Nono	0.319376
Sec14l5	0.319352
Angptl4	0.319347
Abcg4	0.31924
E2f7	0.319127
Pappa	0.319111
Bcl10	0.319109
Pck2	0.319079
Cdc42bpb	0.319068
Ccdc136	0.318911
Dmtf1	0.318902

Trim35	0.318888
Alg1	0.318877
Cic	0.318847
Arih1	0.318801
Ypel1	0.318707
Kbtbd11	0.318667
Dkk1	0.31864
Pola2	0.318569
Id1	0.318569
Ell	0.318522
Ap1m2	0.318455
Arl2	0.318423
Dlx5	0.318396
Endou	0.318215
Tpd52l1	0.318189
Ubxn8	0.318128
Chrac1	0.318102
Zc3h13	0.318085
Arhgap31	0.318077
Csgalnact1	0.317997
Daam1	0.317996
Cldn10	0.317958
Dzip1l	0.317951
Ppm1g	0.317927
Vps72	0.317854
Rdh11	0.317828
Ccdc88c	0.317795
Srsf3	0.317762
Irf2bpl	0.317745
Rnf13	0.317735
Eprs	0.317732
Lrp1	0.317724
Ifitm2	0.317672
1200014J1	0.317603
9030624J0	0.317599
Rala	0.317561
Shc2	0.317519
Ptges3	0.317509
Clic4	0.31749
Stk11ip	0.317456
Ankhd1	0.317427
Slc29a3	0.317351
Crip2	0.317332
Lrrfip1	0.317311
Wdr89	0.317171
Cacna2d3	0.317108
Vamp4	0.317064

Cdip1	0.316988
Myo1c	0.316974
Fut7	0.316949
Ubr5	0.316775
Cetn3	0.316712
Fam13a	0.316699
Rbbp8	0.316576
Nkrf	0.316484
Mis18a	0.316453
Clip3	0.316438
Cacnb1	0.316402
Usp15	0.316375
Mospd3	0.316355
Rgs12	0.316267
Stx16	0.316263
Cdc25b	0.316243
Slc25a15	0.31623
Fat4	0.316204
Nnt	0.316117
Nuak2	0.31602
Herc2	0.316
Brsk1	0.315909
1190007I0	0.315906
Cyb5r4	0.315866
Inmt	0.315856
Coro1b	0.315841
Nuf2	0.315758
Letm2	0.315692
Nol3	0.315671
Med6	0.315638
Ahi1	0.315634
Ufl1	0.315619
Gdpd1	0.315619
Hr	0.315589
Uncx	0.315487
Foxo3	0.315384
Pdia4	0.315354
P2ry13	0.315253
Rhobtb1	0.315247
Fem1b	0.315205
Kctd20	0.315102
Ccnf	0.31507
Nkpd1	0.315026
Sat2	0.315006
Ctu1	0.314987
Ttf1	0.314982
Sidt2	0.314975

Golga4	0.314974
Kcnip2	0.31489
Cebpe	0.314884
Kif23	0.314857
Vav2	0.314834
Fzd8	0.314731
Sgol1	0.314715
Med21	0.314712
Sdad1	0.314702
Gchfr	0.31464
Tlx2	0.314592
H6pd	0.314584
Skp1a	0.314467
Klf11	0.314402
Casz1	0.314338
Fn1	0.314304
B3gnt8	0.314277
Shmt2	0.314234
Maf	0.314137
Zfp622	0.314107
Gstp2	0.314079
BC005764	0.314012
Supt20	0.313952
Mtmr2	0.313911
Pdcl	0.313907
Nfat5	0.313866
Erbb2ip	0.313751
Lipt1	0.313634
Ap2a1	0.313616
Smyd3	0.313588
Krt18	0.31355
Clcn3	0.313512
Ephx1	0.313493
3110040N1	0.313476
Twf2	0.313475
Snd1	0.313442
Popdc3	0.31343
Lrrk2	0.313366
Kcnj4	0.31333
Sypl	0.313305
Nrarp	0.313263
Chrna3	0.313123
Kif26b	0.313058
Ecsit	0.313034
Ltn1	0.313015
Ubac1	0.312992
Phf21b	0.312846

Zc3h3	0.312758
Lclat1	0.312697
Neurod4	0.312684
Bud13	0.312673
Frmd6	0.312668
Abhd1	0.312599
Incenp	0.312547
Rps19bp1	0.312519
Rars	0.312474
5730522E0	0.31238
Zfp329	0.31238
Bik	0.312341
Cox7a2l	0.312174
Prkar2b	0.312094
Tstd3	0.312082
Adamtsl1	0.312052
Dkk2	0.312022
Pde7b	0.311983
Sall4	0.311952
Slc29a1	0.311928
Ptpn13	0.311871
Tmem132e	0.311691
Rce1	0.311607
Pmvk	0.311496
Creb5	0.311323
Trip11	0.311319
Snph	0.311234
Trap1	0.311152
Extl1	0.311081
Sp7	0.311051
Prrt3	0.31098
Perp	0.310941
Adar	0.310934
Fgf22	0.310905
Cep57l1	0.310852
Ggcx	0.310848
Krt14	0.310816
Pag1	0.310745
Tbc1d31	0.310694
Yars2	0.310664
Htr1b	0.310649
Llph	0.310632
Otud7b	0.310621
Usp46	0.310535
Ube2j1	0.310527
Gpr107	0.310512
Ninl	0.310511

Col11a2	0.310431
Uchl3	0.310393
Telo2	0.310349
Gpr37	0.310322
Txndc15	0.31031
Zbtb48	0.31018
Nudcd3	0.310173
Snw1	0.31009
Hivep2	0.31006
Atg16l2	0.310051
Fchsd2	0.310032
Cdh3	0.310003
Smarce1	0.309988
Cux2	0.309895
Nudt14	0.309796
Agtr1a	0.309788
Zfp7	0.309746
Zc2hc1a	0.309745
Cdc7	0.30962
Socs3	0.309582
Cyba	0.309531
Cat	0.309522
Lgalsl	0.309495
Mrc2	0.30943
Slc8a2	0.309415
Loxl1	0.309358
Sap30l	0.309345
Cpsf4l	0.309336
Rasgrf1	0.309314
Akt1	0.309306
Pa2g4	0.309239
Zscan21	0.309208
Sparc	0.309165
Ccdc53	0.309148
Adora2a	0.309136
Spock3	0.309112
Luc7l2	0.309104
Rcn1	0.308959
3-Mar	0.308876
Scn4a	0.30886
Tbc1d32	0.308819
Spata7	0.308814
Ndc1	0.308803
Ret	0.308771
Stk24	0.308768
Zdhhc21	0.308741
Rnf144b	0.308704

Tsga10	0.308688
Bend7	0.308638
Dnajc10	0.308635
Sncb	0.3086
Rapgef3	0.308514
Tnrc6b	0.308422
Topbp1	0.308296
Pccb	0.308287
Skil	0.308233
Midn	0.308228
Csrnp1	0.308089
Map4k4	0.30804
Atrip	0.308021
Fam155a	0.30793
Lrp6	0.307918
Tulp4	0.307915
Nxpe3	0.307912
Arhgap28	0.307783
Lipt2	0.307777
Lipg	0.307717
Pdcd5	0.307712
Ush2a	0.307704
Pik3c2a	0.307687
Mkks	0.307599
Ttc25	0.307594
Faah	0.307553
Notch2	0.307528
Pebp1	0.307524
Hnrnpl	0.307496
Ppp6r1	0.307485
Tcea1	0.307482
Sec22a	0.307474
Hspa4	0.307469
Fech	0.307459
Mettl20	0.307425
Gtpbp8	0.307411
Ttc26	0.307397
Xpo1	0.307372
Slc30a6	0.307342
Crtc2	0.307322
Oraov1	0.307298
Irak3	0.307293
Fnbp1	0.307277
Mcam	0.307274
Cmpk1	0.30724
Tmbim6	0.307095
Tdgf1	0.307077

Bcl2	0.307067
Kcnh8,	0.307051
Cldn3	0.307018
AI593442	0.306938
Slc16a11	0.306915
D10Wsu10	0.30691
Cntn4	0.306895
Akap7	0.306886
Lrrc20	0.306869
Lingo4	0.306798
Tomm70a	0.306712
Capn15	0.306705
Ankrd17	0.306682
U2af2	0.306676
Ptp4a2	0.306667
Ogg1	0.306648
Rgs6	0.30663
Hpcal1	0.306602
Dhps	0.306526
Mtdh	0.306502
F10	0.306499
Aoc2	0.306494
Hbs1l	0.306471
Slc39a5	0.306456
Wnt10b	0.30643
Mcm5	0.306424
Nsmce4a	0.306336
Mafb	0.306213
S100a6	0.306123
4931429I1:	0.30606
Tbc1d9b	0.305978
Dusp22	0.305916
6-Mar	0.305858
Prmt5	0.305825
Skida1	0.305788
Spata24	0.305737
Isg20	0.305735
Sec14l2	0.305735
Nfyb	0.305722
Maea	0.305717
Acnat2	0.305691
Ccdc102a	0.305564
Gja4	0.305555
Wwtr1	0.305545
Myo10	0.305542
Phc2	0.305502
Tmem64	0.305479

Igsf3	0.305456
Cpne6	0.305432
Zfp869	0.305416
Picalm	0.305413
Anapc4	0.305411
Wdr82	0.305393
Pnrc2	0.30535
Taf13	0.305293
BC004004	0.305263
BC017158	0.305252
Spg20	0.305227
Batf3	0.30521
2310067B1	0.305183
Pitx3	0.305182
Sbf1	0.305166
Ndrg3	0.305166
Gap43	0.305094
2410137M	0.304982
Dazap1	0.304963
Cwf19l2	0.304953
Cyth2	0.304952
Ctps	0.304852
B3gnt4	0.304841
Adamts18	0.304824
Uqcc2	0.304798
Dmxl2	0.304793
Slc25a42	0.304747
Rdh14	0.304741
Foxn1	0.304675
Sgta	0.304669
Eef1a1	0.304668
Lss	0.304662
Sec24c	0.304574
5830418K0	0.304471
Cdr2	0.30439
Man1a2	0.304301
Zfp259	0.304278
Plxdc2	0.304218
Zfp53	0.304197
Pspc1	0.30414
Zfp715	0.304118
Abcc5	0.3041
Spg11	0.303905
Tmed4	0.303817
Phf2	0.303805
Msl2	0.303791
Rbms3	0.303627

Khdrbs3	0.303612
Ciart	0.3036
Fem1a	0.303435
Rab26	0.303347
Tango6	0.303343
Fdx1l	0.303326
Gosr2	0.30331
Pcbp3	0.303297
Sdcbp	0.303287
Rassf2	0.303199
Ebf3	0.303177
Prdm5	0.303177
Eif2b2	0.303055
Plcd1	0.303044
Neu3	0.303037
Gsx2	0.30302
Usp1	0.303016
Wdr90	0.303001
Ctage5	0.30299
Cnksr3	0.302979
Lrrc1	0.30297
Pdgfrb	0.302875
Col26a1	0.302865
Radil	0.302836
Immt	0.302812
Pnmal2	0.302794
Commd8	0.302743
Bcap29	0.302727
Rps6ka1	0.302599
Hsp90aa1	0.302531
Tiam2	0.302497
Rnaseh2c	0.30247
Ric8b	0.302407
Slitrk4	0.302357
Hnrnpm	0.302325
Olfr71	0.302272
Fgf4	0.302257
Prss33	0.302228
Pdlim5	0.302215
Twist1	0.302195
Pias3	0.302163
Pcgf6	0.302155
Fsip1	0.30214
Rnf181	0.302086
Fam107b	0.302075
Fam78a	0.302071
Tifa	0.301888

Acsf3	0.301883
Steap2	0.301821
Mvb12a	0.301755
Zfp658	0.301754
Ankrd13c	0.301741
Myocd	0.301729
Rexo4	0.30158
Ints2	0.301571
Ctnnbl1	0.301527
Akirin2	0.301364
B9d1	0.301362
Suv420h1	0.30133
Mzt2	0.301309
Rcbtb1	0.301304
Jtb	0.301272
Rax	0.301211
Tnni2	0.30121
Spata32	0.301177
Hint1	0.301172
Ndrg1	0.301154
Efna3	0.301152
Trhde	0.301131
C1qtnf4	0.301103
Dtx3	0.30109
Impa2	0.30101
Nipal1	0.301001
Hsf2	0.300943
Zfp217	0.300937
Nob1	0.300925
Cyc1	0.300669
2700081O1	0.300596
BC022687	0.300439
Ntmt1	0.300428
Rnase11	0.300428
Ing1	0.300414
Matr3	0.300385
Dhrs13	0.300369
Mpi	0.300355
Pcdh1	0.300335
Pcolce2	0.300238
Nme1	0.30022
Ccdc69	0.300156
Actn1	0.299945
Them6	0.299905
Mip	0.299677
Reln	0.299553
Notum	0.29955

Rsg1	0.299533
Nufip2	0.299508
C1ql3	0.299387
Vapb	0.299379
Hhipl1	0.299325
Cnn3	0.299299
Skor2	0.299295
Hsd17b11	0.299282
Chd3	0.299226
Pigs	0.29918
Syne2	0.299177
Bhlha15	0.29914
Pmp22	0.299127
Sh3pxd2a	0.299101
Ptger1	0.299098
Syt1	0.299072
Slmo1	0.299031
Top2b	0.299004
Cd300lg	0.298979
Nt5e	0.298917
Jarid2	0.298827
Zc3h6	0.298792
Ythdf1	0.298789
Acvr1b	0.298758
Trim45	0.298658
Gucy1b2	0.29863
Ppih	0.298601
Ube2a	0.298568
Naa60	0.298554
Tbce	0.298497
Ppp2r1b	0.29849
Hsph1	0.298475
Gna12	0.298408
Krt23	0.29837
Nt5c	0.298361
9930013L2	0.298358
Gsk3a	0.298346
Zw10	0.298293
Klf4	0.298289
Tsta3	0.298247
Cep55	0.298178
Ephb6	0.29815
Pmaip1	0.298125
Trmt10b	0.298104
Hnrnpab	0.298062
Lurap1	0.297987
Snx30	0.297966

Bcar3	0.297958
H2afy2	0.297837
Flrt1	0.297805
Glis2	0.297799
Slitrk2	0.297796
Adi1	0.297735
Supt3	0.297734
Klhl12	0.297666
Akap6	0.297642
Tbx18	0.297605
Pik3cd	0.297579
Aurkb	0.297568
B630005N1	0.297431
Mctp1	0.297408
Yap1	0.297375
B230217C1	0.297356
Inpp1	0.297273
Ubr2	0.297207
Mob4	0.297178
Naa35	0.297162
Mis18bp1	0.297155
Fcho1	0.297091
Card10	0.296941
Nelfa	0.296912
Atp6v0e2	0.296904
Fgf13	0.296792
Psma3	0.296762
4632415L0	0.296752
Cd151	0.296676
Zfp930	0.29667
Ggnbp2	0.296629
Rnf44	0.296624
Wdr4	0.296607
Tead1	0.296581
Cds2	0.296571
Etfa	0.296559
Eef1a2	0.296541
BC017643	0.296526
Tfap2c	0.296513
Alg14	0.296507
Grik5	0.296501
Hnrnpf	0.296481
Zkscan1	0.296455
Tcp11l1	0.296454
Bub1b	0.296437
Slc16a14	0.296434
Mybphl	0.296386

Purb	0.296378
Crcp	0.296367
Mdfi	0.296342
Pde4a	0.296301
Celsr3	0.296256
1810043G(	0.296088
Nae1	0.296083
Edf1	0.296065
Il6st	0.296062
Prlhr	0.296003
Lck	0.296002
Pot1a	0.29597
Tceb2	0.295945
Col7a1	0.295939
Vwa2	0.295939
Adam17	0.295816
L3mbtl2	0.295743
Arhgef10	0.295673
Fbrsl1	0.295673
Lsm14a	0.295652
Pi16	0.295622
Mecr	0.295594
Gstm4	0.295575
Mettl16	0.295568
Klc1	0.295543
Pim3	0.295539
Clpb	0.295539
Usp40	0.295526
Tox2	0.295499
Aco1	0.295467
Wfdc1	0.295467
Pdxdc1	0.295446
Kcnn1	0.295445
Adprh	0.295443
Gpd1	0.295418
Mgat5	0.29539
Praf2	0.295353
Gale	0.295327
C1rl	0.295317
Tppp	0.295294
Xpot	0.295287
Lmnb2	0.295237
Agl	0.295206
Myb	0.295166
Ccnjl	0.295139
Fscn2	0.295084
Cul9	0.29508

Irs3	0.295074
Cdc25c	0.295062
Mei4	0.295024
Mob1b	0.295023
Epb4.1l5	0.294946
Mast2	0.294916
Clptm1l	0.294809
Rilpl2	0.294788
Mvd	0.294763
Fntb	0.294744
Sirt7	0.294737
Dnm1	0.294718
Ssbp2	0.294706
Gpihbp1	0.294677
Hpgd	0.294531
Llgl2	0.294526
Stxbp5	0.294491
Mpzl1	0.294472
Pls1	0.294287
Gde1	0.294124
Tpk1	0.294122
Adra2b	0.294079
Scyl1	0.294002
Lrrc15	0.293944
Psen2	0.293873
Rspo3	0.293858
Unk	0.29384
Glipr1l1	0.293827
Lhfpl2	0.293807
Fut11	0.293789
Rnpepl1	0.293785
Il2rb	0.29375
Mettl3	0.293749
Dpys	0.293697
Ccdc109b	0.293643
Rif1	0.293569
Kdelr3	0.29356
Smg8	0.29354
Zfp945	0.293496
Bmi1	0.293424
2210018M	0.293407
Unc5a	0.293363
Prr14	0.293358
Rasa2	0.293356
Nodal	0.293345
Amz2	0.29317
Atg2a	0.29309

Zbtbd6	0.29307
Peli2	0.293066
Rab3ip	0.293035
Tinf2	0.292989
Nnat	0.292925
Gjd3	0.292918
Lrrn4	0.292892
Mcts2	0.292871
Slc26a2	0.292863
Zfp157	0.292824
Slc29a2	0.292784
Cend1	0.292777
Rad50	0.292751
Fbxo7	0.292738
Upp1	0.292721
Mbnl2	0.292696
Nt5m	0.29269
D8Ertd82e	0.292658
Bace2	0.292646
Nkx3-2	0.292643
Chfr	0.29258
Ccdc138	0.292572
Mblac1	0.29256
Nt5dc3	0.292522
Uck2	0.292514
Spata2l	0.292498
Dact2	0.292481
Rab36	0.292469
Dcst1	0.29242
Slc16a6	0.292407
Siah3	0.292399
Zfp385c	0.292389
Mterf1b	0.292368
Atp8a1	0.29223
Clec12b	0.292224
Mical1	0.292188
Ppp1r14c	0.292184
Zbtb41	0.292173
Metrn	0.292138
Olig1	0.29204
Snapin	0.292032
Baz1b	0.291955
Tmem219	0.291863
Dusp7	0.291842
Akirin1	0.291749
Laptm4b	0.29174
Ndufv1	0.291672

9230110C1	0.291662
Kazn	0.291653
Cyp20a1	0.291615
Matn1	0.291608
Tmod1	0.291608
Tns3	0.291574
Slc16a10	0.291523
Dcbld1	0.291389
Mfsd5	0.291374
Tmem108	0.291365
Gtf2f1	0.291347
Strn	0.291297
Tgfa	0.291266
Qtrt1	0.291208
Prkag1	0.291184
Kcnh6	0.291184
Rab31	0.291166
Per2	0.291117
Bcdin3d	0.291106
Snrpb2	0.291077
Slc25a35	0.291072
Rpl24	0.291048
Prtg	0.291045
Stat3	0.291037
Gm996	0.290982
Rora	0.290955
Map3k9	0.290912
Pcmtd2	0.290902
Ttll1	0.290879
Enpp3	0.290874
Nhlrc1	0.290853
Map10	0.290841
Fam21	0.290737
Gata5	0.290705
Gar1	0.290696
Gpr39	0.290664
Mrpl1	0.290641
5830417I1(	0.290608
Dlat	0.29056
Sepn1	0.290453
Tmem120a	0.290446
Ube2s	0.290435
Rab21	0.2904
Dpy19l1	0.290391
Barhl1	0.290389
Dopey2	0.290347
Ccrn4l	0.290267

Slbp	0.290236
Trim65	0.290217
Hyou1	0.290195
Tmem147	0.290181
Kdm5b	0.290159
Zfp358	0.290142
Arhgap12	0.290067
Luc7l	0.290026
Atp5o	0.290023
Cenpf	0.289949
Nudt16l1	0.289873
Eral1	0.289835
Ercc1	0.289831
Creld1	0.289791
Nudt10	0.289742
Chmp7	0.28972
Sgk3	0.289661
Hcrtr2	0.289649
Ywhaz	0.289603
Nfatc3	0.289572
Rpl29	0.289505
Ap5s1	0.289499
Nol12	0.28948
Tom1	0.289458
Ddx27	0.289435
1600014C1	0.289362
Aga	0.289303
Rnaseh2b	0.289254
Arsb	0.28922
Dusp23	0.289199
Nup155	0.289197
Rbm5	0.289184
Ptpn5	0.28909
Tmem170b	0.289057
Foxa2	0.289015
Nudt19	0.288954
3-Sep	0.288899
Dcakd	0.28884
Ggt6	0.288823
Cln8	0.288821
Klhl42	0.288819
Mlec	0.288801
Zfp317	0.288782
Ube2v1	0.288777
Rnf4	0.288753
Chmp2b	0.28875
Slc39a13	0.288665

Sertad3	0.288633
Pnn	0.288579
Ppdpf	0.288538
Slc38a10	0.288526
Adamts12	0.28852
Kctd6	0.288439
Haus5	0.288437
Dfna5	0.288421
Sned1	0.288371
Ppp2r5a	0.288371
Ftl1	0.288324
Efcab4a	0.2882
Bbc3	0.288187
Qrich1	0.288175
St6galnac4	0.288167
Las1l	0.288148
Zbtb24	0.288135
Klhdc7b	0.288054
Zbtb7a	0.287978
C77370	0.287914
Rbm39	0.28788
Il13	0.287867
Pank2	0.287857
Pla2g12b	0.287842
Ilvbl	0.287799
Plk1	0.287794
Rragd	0.28779
Fasn	0.287773
Vgf	0.287766
Abcb6	0.287719
Lsg1	0.287609
Mxd4	0.2876
Tead3	0.287589
2700062C0	0.287586
Eif3c	0.287567
Nrros	0.287559
Mettl22	0.287526
Zfand2b	0.287517
Pramef8	0.287436
Scrib	0.28741
Zscan20	0.28738
Tbrg4	0.287363
Kcnh2	0.287338
Sfrp1	0.28733
Gpsm1	0.287257
Bola3	0.28722
Tenm3	0.287218

Susd3	0.28714
Rock2	0.287121
Stac2	0.287121
Wbscr28	0.287103
Me2	0.287046
Hoxa10	0.286987
Tmem154	0.286983
Clstn3	0.286873
Hnmt	0.28686
Eppk1	0.286822
Btn1a1	0.286775
Mars2	0.286761
Sema6d	0.28673
Ubxn7	0.286705
Mapk12	0.28654
St8sia1	0.286452
Nabp1	0.286444
Ttc32	0.286423
Eepd1	0.286399
Lrrc58	0.286391
Akr1b10	0.286379
Cep68	0.286368
Aqp6	0.286319
Clvs1	0.286209
Dlx4	0.286206
Egfl7	0.286194
Thbs1	0.286166
Scgb3a1	0.28614
Nanp	0.286075
Cdca4	0.286057
Ubtf	0.286047
Cckbr	0.286003
Slc16a1	0.285972
Foxd3	0.285943
Gmps	0.285932
Ddhd1	0.28593
Galr3	0.285883
Neo1	0.285786
Angel2	0.285785
Lmna	0.28577
Zbtb44	0.285688
Helb	0.285604
Ncam1	0.285597
Thop1	0.285584
Rccd1	0.285523
Fancf	0.285497
Tspan18	0.285491

Parl	0.28539
Ctsd	0.285365
Gpr97	0.285331
Map4k2	0.285326
Tonsl	0.285321
Arf1	0.285311
Grid1	0.285286
Fbxo47	0.285274
Fam188b	0.285245
Abl1	0.285232
Pepd	0.285207
Ldlrad3	0.285148
Dusp14	0.285135
Ccdc66	0.285085
Hpca	0.285071
Gm2382	0.285044
Zfp706	0.285007
Wdsub1	0.285
Scaf8	0.284968
Erbb3	0.284902
Rbp1	0.284892
Cnnm2	0.284747
Scx	0.28467
Slc35b4	0.284608
Slc25a30	0.284594
Ccdc126	0.284562
Zfp703	0.284546
Qser1	0.284541
Mphosph6	0.28447
Qrfp	0.284351
Wnt3a	0.284299
Ly6a	0.284257
Phactr4	0.284186
D4Wsu53e	0.284141
Sox4	0.284109
Igdcc3	0.284101
C030034I2	0.284093
Arhgef2	0.284068
Vegfb	0.28403
Thrap3	0.283998
Misp	0.283993
Dbnl	0.283955
Hspb7	0.283929
Gprin1	0.283787
Gpatch1	0.283739
Prkcq	0.283714
Sumo2	0.283704

Itm2b	0.283685
Me3	0.283681
Med4	0.283657
Pold3	0.283619
Grm8	0.283609
Tmbim4	0.283604
Adat1	0.28357
Cdc23	0.283491
Bcl7a	0.283483
Fuca1	0.283455
Gramd4	0.283442
Rho	0.283437
Brk1	0.283388
Grin2a	0.283385
Vstm2a	0.283342
Usp3	0.283309
Rbpms	0.283279
1810058I2	0.283268
Gtf2a1	0.28314
Mlh3	0.283125
Dnal4	0.283084
Rfx1	0.283014
Slc33a1	0.283006
Sod1	0.282988
Apln	0.282944
Flnb	0.282927
Mgarp	0.282908
Zbtb10	0.282906
Fbxo30	0.282874
Tcam1	0.28287
2610044O	0.282826
4922501C	0.282821
Sdhaf1	0.282795
Zfp111	0.28271
Them4	0.282671
Lman1	0.282659
Arl5c	0.282636
Btbd10	0.282596
Gnat1	0.282558
Sntb1	0.282481
Celf3	0.282279
Cadm1	0.282269
Nupr1	0.28217
Acsl6	0.282135
Nol7	0.282129
Ip6k2	0.282116
Cops3	0.282067

Tex2	0.282006
Hprt	0.28196
Sh3bgrl	0.281947
Stt3b	0.281945
Sync	0.281936
Ano7	0.281906
Sike1	0.281873
Samm50	0.281853
Kctd15	0.281792
C1ql2	0.281719
Suco	0.281686
Zfp825	0.281681
Kdm1a	0.281643
Aanat	0.28163
Fam195a	0.281596
Dennd6a	0.28147
2310039H(	0.281459
Umps	0.281419
Pgam2	0.281415
Rbm34	0.281399
Gtf3c2	0.281395
G6pc3	0.281201
Tbpl1	0.281164
Azi2	0.281154
Sorcs3	0.281116
Med7	0.281115
Scrn1	0.281097
Dolpp1	0.281059
Mmaa	0.281047
Caml	0.281016
Mybl1	0.280948
Habp4	0.280864
Diap3	0.280858
Dhrs4	0.280827
Rab34	0.280811
Lad1	0.28078
Nmt2	0.280716
Cartpt	0.280688
Prkca	0.280623
Bclaf1	0.280565
Aldh9a1	0.280556
Ptgr1	0.280541
Nxpe4	0.280511
Bbs9	0.280451
Tulp3	0.280385
Klc2	0.280374
Kcnip3	0.280358

Sap30	0.280314
Dusp19	0.280304
Coq5	0.280289
Faf2	0.280255
Fam60a	0.280252
Oscp1	0.280252
Gpr114	0.280219
Sema3g	0.280206
Tnfsf13b	0.280193
Psme3	0.280166
Mfsd10	0.280165
E4f1	0.280049
Ate1	0.280042
Thbd	0.280036
Commd9	0.27995
Brf2	0.279949
Sord	0.279931
Arap1	0.279905
Kmt2b	0.279883
Treml1	0.279839
Tob1	0.279832
Rgma	0.279777
Ttc14	0.279773
Spag1	0.279709
Zcchc2	0.279686
Rad21	0.279664
Jade1	0.279651
Ufsp1	0.279599
Nup214	0.279539
Tmem150a	0.27951
Tmem39a	0.279464
Angptl6	0.279451
Zfp30	0.279424
Fam83d	0.279377
Zfp560	0.279361
Vcpip1	0.279343
Naa25	0.279288
Gpr137b-p	0.279277
Tnfaip2	0.27927
Zfp202	0.27923
Trim27	0.279196
Epc1	0.279135
Klhdc2	0.279074
Aplf	0.278911
Ints6	0.278909
Ank	0.278862
Rbm4b	0.278848

Ptk7	0.278804
Shank2	0.278774
Mcph1	0.278679
Mga	0.278659
R3hdml	0.278546
Zp1	0.278489
Abtb2	0.27839
Nkd2	0.278279
Hsd17b7	0.278276
Stard4	0.278217
Tcf7	0.2782
Slc37a2	0.27809
Snrpa	0.278015
Stx12	0.277971
Fam20a	0.27795
Fabp7	0.277947
Plcb2	0.277938
Penk	0.277932
Dimt1	0.277858
Sulf1	0.277832
Arhgap17	0.277796
Atp10d	0.277789
Uri1	0.277784
Suox	0.277722
Adck2	0.277713
Insm1	0.277703
Fads1	0.277652
Gjb3	0.277599
Shkbp1	0.277576
Olfml2a	0.277527
Bnip1	0.277511
Mrgprf	0.2775
Cyp4f13	0.27746
Ifnar1	0.277434
Cc2d2a	0.277425
Osbpl2	0.277419
4930523C0	0.277406
Cbl	0.277383
Srd5a3	0.277339
Grm3	0.277334
Mcfd2	0.277305
Ercc3	0.277124
Smek2	0.276859
Klhl36	0.276807
Canx	0.276704
Fign	0.276692
Necap2	0.276663

Megf8	0.276639
Zfp398	0.276633
Sstr2	0.276603
Nfkb1	0.27658
Hsd17b4	0.276575
Fubp3	0.276543
Gorasp2	0.276471
Setd8	0.276468
Mrap2	0.276414
Zfp958	0.276392
Pias1	0.276299
Samd4	0.276239
Gch1	0.276232
Lmo7	0.276195
Sertad1	0.276174
Dach2	0.276125
Exoc2	0.276085
Srpk2	0.276079
Pcbp1	0.276068
Icam1	0.276043
Krt86	0.276033
Ube2ql1	0.275974
Gprc5c	0.275966
1700101E0	0.275962
Gtf2a2	0.275959
Mrps15	0.275917
Rpl38	0.275911
Ccnc	0.2759
Bglap2	0.275895
Bdkrb2	0.275861
Zbed4	0.275793
1810022K0	0.275785
Vwa5b2	0.27578
Pvalb	0.275686
Ampd2	0.275661
Ckap2l	0.275606
Dtwd1	0.275541
Zfp738	0.275524
Cyp46a1	0.275476
Irf8	0.275471
Vps45	0.275453
Ssb	0.275417
Qk	0.275411
Nlk	0.275394
C2cd2l	0.27538
Ripk4	0.275348
Zfpm1	0.275346

Dbh	0.275261
Med17	0.27526
Prkab1	0.275252
Glrx	0.275214
Gna13	0.275208
Vamp3	0.275155
Tmub2	0.27511
Ntf3	0.275067
Rbm6	0.275055
Tex264	0.275033
Vldlr	0.275026
Pdzd3	0.274984
Nptx1	0.274958
Ttc9	0.274956
Tulp1	0.274933
1700039E1	0.274873
2300005B0	0.274864
Fanca	0.2748
Immp2l	0.274784
Tcl1b4	0.274779
Haus6	0.274748
Trmt12	0.274735
Akap11	0.274655
Vkorc1l1	0.274627
Klhl5	0.274596
P4htm	0.274577
Tubb4b	0.274569
Kif3c	0.274517
Cep170b	0.274504
Fstl5	0.274369
Ccdc88a	0.274352
Plxnc1	0.274328
Lhb	0.274286
Ficd	0.274158
1110007C0	0.274155
Ascc2	0.274143
Car6	0.274107
Mier2	0.274077
Ppp1r37	0.274062
Slc39a10	0.274022
Nubpl	0.273989
H2afy	0.27394
Sec11a	0.273934
Bche	0.273755
Ropn1	0.273746
Echs1	0.273741
Mgmt	0.273665

Iqcc	0.273541
Galnt18	0.273509
Ada	0.273482
Tm2d3	0.273456
Ghsr	0.273456
Preb	0.273454
Letmd1	0.273446
Wdr31	0.273434
Bbx	0.273431
Map3k5	0.273421
C2cd5	0.273376
Abca3	0.273335
Rps17	0.273316
Thoc2	0.273282
Xylt2	0.273235
Dctn6	0.273192
Xkr7	0.273172
Ppp6c	0.273146
Pard6g	0.273128
Gng10	0.273053
Phip	0.272989
Dock5	0.272966
Calr3	0.272926
Tfeb	0.272913
Psap	0.272821
Plekhg4	0.272821
Ptrf	0.272789
Bcr	0.27267
Ovol1	0.272652
Zfp747	0.272638
Atxn7l3	0.272594
Usp22	0.272568
Mrpl17	0.272561
Fan1	0.272537
Ppid	0.272531
Mad2l1	0.272516
Dynll1	0.272464
Vcp	0.272424
Atp1b1	0.272378
Cnep1r1	0.272332
St8sia5	0.27228
Anapc7	0.272265
Brd4	0.272257
Edem2	0.272198
Hinfp	0.272154
Slc22a5	0.272139
Sh3bgrl2	0.272122

Nudt3	0.27212
1700016H1	0.272071
Opn5	0.272047
Pop7	0.272039
Ube2q1	0.272037
Ascl4	0.272002
Rnf146	0.271971
Ppm1m	0.271968
Trim14	0.271952
Pax1	0.271912
Pkia	0.271809
Pdcd6	0.271799
Bdh2	0.271728
Map6	0.271722
Spats2	0.271686
Ubr1	0.271636
Tmem55a	0.271608
Csnk1e	0.27157
Hnrnpd	0.271524
Pax2	0.271522
Rwdd2b	0.271511
Ralgapb	0.271473
Cuta	0.271455
Zfp414	0.271441
0610037L1	0.271405
Arl15	0.271379
Cops7a	0.271375
Aqp3	0.271327
2210404O(	0.271308
Guca1b	0.271282
Wdr55	0.271273
Tfdp1	0.271134
Mrpl24	0.271113
Slc19a1	0.271113
Dohh	0.271102
Tbx10	0.271078
Phyhip	0.271071
AW549877	0.271051
Ift172	0.271045
BC037034	0.270909
D930020B1	0.270877
Pom121l2	0.270871
Sh2d7	0.270842
Akip1	0.270818
Ulk2	0.270815
Ippk	0.270807
B3gnt5	0.270807

Guf1	0.270762
Vps4a	0.270728
Pnp	0.270708
Tspyl5	0.270703
Fopnl	0.27064
Mex3d	0.270599
Dhx58	0.270579
Il12rb1	0.270562
Cdc25a	0.270538
Slc9a6	0.270536
Gna14	0.270536
Rnd1	0.270496
Actl11	0.270494
Ptprz1	0.270383
Itfg2	0.270378
Tmcc3	0.270262
Trpc3	0.270197
Grip1	0.270178
Mbnl1	0.270151
Nyap1	0.270101
Zfp438	0.270058
Nfkbiz	0.270041
Lif	0.270028
Nefm	0.269999
Icosl	0.269999
Psmd6	0.269995
Ets1	0.269985
Ipo4	0.269885
Polr1e	0.269882
Fam160b1	0.269879
Mgat5b	0.269866
Hif3a	0.269856
Prtn3	0.269851
Hdgfrp2	0.269773
Shc4	0.269726
Rpp14	0.269661
Ahr	0.269605
Zfp444	0.269597
Myh3	0.269528
Mkrn3	0.269507
Car5a	0.269493
Atp6v1c2	0.269474
Dyrk3	0.269437
Dok3	0.269421
Ddx6	0.269405
Lama5	0.269387
Med24	0.269354

Pttg1ip	0.269339
Jph4	0.269338
Grk6	0.269297
Cdh13	0.269279
Gstm7	0.269202
CK137956	0.269125
Tma16	0.26908
Mmd	0.269034
Chst14	0.269012
Tmem185b	0.269
Slc16a5	0.268999
Lpar3	0.268892
Echdc1	0.268874
Avp	0.268783
Ralgapa1	0.268755
Mad2l1bp	0.268743
Tmem45a	0.268725
Atg16l1	0.268689
Arhgap26	0.268667
Mbd2	0.268573
Nras	0.268566
Armc7	0.268534
Hs6st3	0.268524
Ly96	0.268464
Tnpo3	0.268442
Slc35a3	0.268426
Cnot3	0.26842
Gins1	0.268368
Th	0.268334
Mrpl20	0.268261
Tmem141	0.268207
Tmem119	0.268181
Exoc3l4	0.268157
Gins4	0.268148
4833420G1	0.268066
Rsl24d1	0.268052
Hmgcl	0.268025
Dazap2	0.267986
A4galt	0.267973
Mier3	0.26797
Carm1	0.267957
Capn11	0.267869
Lgi3	0.267816
Fktn	0.267783
Stat5a	0.267773
Gnpnat1	0.267756
Kif16b	0.267743

Ccdc42	0.267735
Camk2a	0.267722
Amer3	0.267688
Rnf19a	0.267518
Arhgef18	0.267472
Rnf217	0.267418
Scmh1	0.267397
Tmem50b	0.26733
Gja3	0.267191
Gamt	0.267167
Prpf38b	0.267115
Capn10	0.267008
Prph	0.266963
Ppa1	0.26694
Gins3	0.266932
Lrp11	0.266926
Cstf2t	0.266861
Eif4enif1	0.266793
Met	0.266734
Pak7	0.266671
AF529169	0.266642
Erlin2	0.266632
Atp8b2	0.266626
Atxn7l2	0.266598
Secisbp2l	0.266555
Megf11	0.266545
Pcnt	0.266517
Casp8	0.266504
Sptssb	0.266497
Lrrc8d	0.266456
Papd7	0.266431
Pald1	0.266401
Hspa12b	0.266371
Map1lc3a	0.266295
Prss12	0.266284
Gm5878	0.266277
Tpp2	0.266266
Itm2a	0.266225
Lrrc59	0.2662
P2ry6	0.266175
Commd1	0.266094
Hook1	0.266087
Ercc6l2	0.265982
Ptpn18	0.26597
Mlxipl	0.265962
Ncs1	0.265943
Igsf21	0.265931

Ttpal	0.265928
Fev	0.265919
Ccbl2	0.265889
Trim3	0.265886
Calu	0.265842
Tubb2a	0.265832
Serbp1	0.265804
Trhr2	0.265797
Kcna1	0.265741
Kdm8	0.265715
Lrrc4	0.265713
Tial1	0.265678
Slc25a33	0.265627
Dnph1	0.265576
Cd109	0.265534
Alpl	0.26552
Arap3	0.265484
Rexo1	0.265466
Mtg2	0.265462
Sertm1	0.26546
Foxi3	0.265446
Pex16	0.265416
Flna	0.265403
Peli3	0.265384
Abcg1	0.265367
Rab11b	0.265361
Epha2	0.265317
Scoc	0.265217
Pskh1	0.26521
Zfp354c	0.265118
Mthfd2l	0.265087
Sae1	0.265066
Gucy1a2	0.265062
Fam170b	0.265013
Alg9	0.265003
Bnip2	0.264967
Ankrd6	0.264937
Tmem221	0.264907
Cenpm	0.264885
Coa7	0.264861
E330009J0	0.26485
Mon2	0.264784
Wdr45b	0.264726
Nfkb2	0.264726
Ipo8	0.264706
Kif18b	0.264689
Rab6b	0.264559

Dnajc27	0.264549
Phlda2	0.264529
Trim36	0.264486
2700097O(	0.264475
Elf4	0.264376
Emilin2	0.26436
Il10ra	0.264318
Aig1	0.264287
Eif4b	0.26422
Aldh5a1	0.2641
Sgpl1	0.264059
Vapa	0.264036
Rbl1	0.264015
Ckmt1	0.264004
Alkbh8	0.263989
Pip4k2b	0.263978
Polr3e	0.2639
Dock6	0.26382
Cox17	0.26381
3010026O(	0.263808
Tmem151b	0.263803
Ckap4	0.263779
Hapln3	0.263768
Timm44	0.263748
Irf1	0.26372
Ccnt2	0.263644
Map2k6	0.263637
Sfswap	0.263551
Kit	0.263519
Cerk	0.263502
Cdc42ep5	0.263473
Bcar1	0.263472
Lypd3	0.263396
Acvrl1	0.263317
Sos1	0.263312
Pou3f2	0.26329
Adnp2	0.263256
Npc1	0.263253
Zbtb26	0.263246
Blm	0.26324
Ar	0.26324
Ppap2b	0.263234
Hus1	0.263218
Slc35b2	0.263168
Spry4	0.263165
Arfgef1	0.263153
Tesc	0.263104

Npy5r	0.263095
Zfand1	0.263086
Cggbp1	0.262921
Dync1li2	0.262919
Add1	0.26291
Cpne8	0.262891
Cops6	0.262847
Relt	0.262688
Ccdc124	0.262667
Crtc3	0.26265
Chl1	0.262648
Cdk10	0.262606
Defb35	0.262592
Ecm1	0.262576
Fbxo33	0.262571
Bmp7	0.26254
Tex22	0.262457
Tcaim	0.262443
Lactb2	0.262426
Znrf3	0.262387
Fam211b	0.262354
Lpcat3	0.262344
Rab33b	0.262262
Copg1	0.26226
Ddhd2	0.262244
Xpo4	0.261988
Enpp5	0.261959
Efcab14	0.261952
Ppp1r12b	0.261867
Rps5	0.261863
Dhcr24	0.261854
Ipo9	0.261828
Gcdh	0.261789
Stau1	0.261788
Aym1	0.261748
P2rx1	0.261733
Ffar1	0.261683
Als2	0.261677
Map4	0.261648
AI467606	0.261643
Cyp4v3	0.261643
Eed	0.261593
Rab5c	0.261591
Tor1b	0.261567
Rgs17	0.261541
Gm6498	0.261528
Arntl	0.261473

Tmem229a	0.261434
Pex10	0.261404
Elavl4	0.261376
Dvl2	0.261365
Rps12	0.261299
Csnk1a1	0.261282
Caprin1	0.261243
Pkd2l2	0.261242
Azin1	0.261201
Syde1	0.261197
C78339	0.261195
Ccni	0.261194
Ankrd10	0.261126
Mrps14	0.261121
Pitpnm2	0.261008
Tmem62	0.260956
Kndc1	0.260945
Sod2	0.260847
Reep4	0.260839
Arrdc2	0.260825
Zfp59	0.260822
Col13a1	0.260749
Flot2	0.260717
Kdm4a	0.260699
Plxnb2	0.260677
Cyb5	0.260662
Tsku	0.260654
Necap1	0.260625
Farsa	0.260619
Shbg	0.260579
Kif3b	0.260573
Zmpste24	0.260559
Zfp273	0.2605
Tmed10	0.260481
Pak4	0.260448
St3gal5	0.26044
Pdia5	0.260433
Psmb11	0.260394
Zdhhc7	0.260341
Prss23	0.260333
Gpr157	0.260308
Ap5b1	0.260271
Ppil4	0.260215
Phospho1	0.260168
Mtr	0.260111
Muc6	0.260098
Alg8	0.260075

Tmem2	0.260029
Atp6v0e	0.259977
Elof1	0.259899
Fem1c	0.259861
E130012A1	0.259854
Dock11	0.259823
Leng1	0.259807
Rbck1	0.259781
Slc12a9	0.259769
Uba6	0.259755
Fam110a	0.259594
Zmat2	0.259538
Brinp1	0.259431
Lrrc55	0.259361
Pafah1b2	0.259332
Atp1a2	0.259259
Galc	0.259223
Sars	0.259186
Zfp389	0.259181
Nr2f6	0.259096
Dtx2	0.259077
Snx22	0.25904
Shmt1	0.259038
Rlim	0.259018
Rnf10	0.259004
Abcb7	0.258912
Crk	0.258891
Tbc1d22a	0.258888
Rfesd	0.258867
Nasp	0.258801
Sdccag8	0.25871
Lrmp	0.258595
Tmem255a	0.258577
Plcxd3	0.258468
Det1	0.258468
Ift57	0.258407
Actr1b	0.258378
Nek11	0.258347
Trp53i11	0.258209
Smarcc2	0.258144
Zfp870	0.258143
Ubxn4	0.258117
Sh3bgrl3	0.258109
Mapk8ip1	0.258099
Chmp6	0.258079
4930452B0	0.25807
Tmem42	0.258053

Ppm1e	0.257898
Scfd1	0.257891
Dnase1l3	0.257874
Tipin	0.257858
Cops7b	0.257821
Sav1	0.257791
Gria2	0.257747
Pigh	0.257725
Zfp385a	0.257721
Wdr34	0.257697
Pde7a	0.257563
Nrtn	0.257561
Npas2	0.257528
Tbx6	0.257514
Rap1gap	0.2575
Wdr24	0.257446
Zfp704	0.257436
Rcc1	0.25737
Narfl	0.257356
Setd2	0.257334
Fam174b	0.257302
Pwwp2a	0.257281
Dnajc15	0.257279
Irf4	0.25726
Tctex1d2	0.257212
Zfp286	0.257209
Vamp1	0.257199
Frmd4b	0.25714
Zfp120	0.25714
Fbxo5	0.257117
Bace1	0.2571
Hist2h3c2	0.25709
Vps33b	0.257089
Rnd3	0.257075
Usp12	0.25706
Vma21	0.257041
Hcfc1	0.257024
Crot	0.257021
Cwc22	0.256994
Rundc3a	0.256949
Hipk1	0.256923
Atad1	0.256898
Atp11b	0.256889
4-Mar	0.256888
Psmc3ip	0.256885
Ascl2	0.256824
Ssx2ip	0.256766

Crb1	0.256724
Atxn1l	0.25669
Mapk7	0.25663
Nsmaf	0.256617
Trpc6	0.256603
Etnk1	0.256502
Lypd2	0.256473
Arhgap24	0.256397
Rabl2	0.256339
Golph3	0.256327
1700067K0	0.256316
Cbfa2t3	0.256313
Caprin2	0.256295
Dgki	0.256248
Wdr20	0.256147
Zfyve1	0.256129
Rb1	0.256066
Prorsd1	0.256057
Nkd1	0.256046
Phax	0.255884
Degs2	0.25584
Mxra7	0.255819
Ffar4	0.255796
Hist1h4b	0.255767
Doc2b	0.255721
Kif2c	0.255702
Pigc	0.255699
Sptssa	0.255688
Zfp790	0.255669
Clmp	0.255657
Rpl10a	0.255569
2810008D0	0.25556
Hmha1	0.255557
2610001J0	0.255533
Odf1	0.255528
Pou3f4	0.255326
Hmgxb3	0.255286
Ednra	0.255191
Wdr47	0.25516
Arpc3	0.255147
S100a11	0.255084
Slc35c2	0.255052
Aptx	0.254981
Fam189a1	0.254972
Wnk4	0.254925
Tcf20	0.254921
Nfatc2	0.2549

Rhbdd2	0.254874
Fbxo6	0.254865
Zfp39	0.254848
Hoxc13	0.254834
Lrrc10b	0.254804
Pdcd4	0.254799
Rps14	0.254706
Msrb2	0.254695
Amn	0.254632
Pde8a	0.254628
Clu	0.254615
Ndnl2	0.254579
Ufm1	0.254552
Khdrbs2	0.254526
Dync2h1	0.254522
Heatr6	0.254465
Kpnb1	0.254421
Scp2	0.2544
Tpm2	0.254373
Plekho1	0.254342
Rab20	0.254319
Tbkbp1	0.254239
Ern1	0.254228
Chchd6	0.254216
Gucy1b3	0.254145
Tcirg1	0.254144
Cmtm7	0.254109
Foxp2	0.254021
Pank3	0.254008
Mdk	0.253926
Hoxd3	0.253886
Osbp	0.253879
Nfatc2ip	0.25386
Tuba1c	0.253856
Tmtc1	0.253836
Zmym5	0.25377
Plrg1	0.253705
Max	0.253683
Calm3	0.253591
Cdh2	0.25358
Nefh	0.253527
Ccdc80	0.253503
Pld3	0.253487
Abcc1	0.253436
Mast1	0.253435
Zhx3	0.253395
Map3k1	0.253279

Ano10	0.25327
Insr	0.253185
Zfp148	0.253152
Dpy19l3	0.253139
Ifnlr1	0.253138
Prkx	0.25307
Igf1	0.253052
Tbc1d4	0.253015
Tmem131	0.25299
Nudt4	0.252963
Comp	0.252901
Kpna1	0.252839
Jph1	0.25282
Tusc3	0.252789
Smc5	0.25273
Hunk	0.252725
Shroom4	0.252712
Cnp	0.252707
Neurod2	0.252641
Rnf180	0.252625
Trim47	0.252497
Sytl1	0.252489
Dpp7	0.252476
Fam228a	0.252393
Tbx15	0.252284
P2ry12	0.252228
Acot2	0.252219
Asb2	0.252204
Ap2a2	0.252204
Megf6	0.25218
Lgmn	0.252137
Chrm3	0.252046
1810041L1	0.25203
Rabep2	0.252012
Malt1	0.251992
Uap1l1	0.251971
Hif1a	0.251965
Tcf15	0.251948
Samd5	0.251938
Ranbp2	0.251873
Ppfibp1	0.251831
Entpd2	0.251734
Glt25d1	0.251715
Hdac5	0.25171
Stk17b	0.25169
Ccdc141	0.251676
Aktip	0.251583

N4bp2l2	0.251534
Ehmt2	0.251529
Coro6	0.251501
Tmpo	0.251487
Adap1	0.251438
Ankrd11	0.251434
Saysd1	0.251386
Dph7	0.251382
Lrcol1	0.251362
Necab2	0.251337
Slc24a5	0.251329
M6pr	0.251308
Tyrobp	0.251282
Pten	0.251268
Pcyt1a	0.251257
Slc7a6	0.251239
Gria4	0.251159
Gcc2	0.251126
Kctd8	0.251115
Plcxd2	0.251078
Rps10	0.251063
Ptdss2	0.251043
Zdhhc8	0.251005
Amhr2	0.250926
0610009D(	0.250886
Agpat5	0.250862
Fbxl4	0.250759
Oma1	0.250686
Trim44	0.250669
Lyrm9	0.250663
B4galt3	0.250659
Yeats2	0.250651
Gpcpd1	0.250649
Zfp395	0.250637
Hdac8	0.250596
Lrdd	0.250546
Il1rapl1	0.250535
Lag3	0.250518
Pnma2	0.250432
Ssbp3	0.25041
Sccpdh	0.250395
Il17ra	0.250378
Mst1r	0.250308
2810417H1	0.250297
Rhpn1	0.250271
Slc22a4	0.250231
Tmem19	0.250203

Mtap	0.25018
Rtca	0.250165
Rab4a	0.250132
Zfp655	0.25013
Slc27a2	0.250116
Gtf2h2	0.250094
Ccnh	0.250068
Coro2a	0.250059
Has3	0.250018
Pnoc	0.250011
Helz	0.250008
Pmpcb	0.24998
Sgpp1	0.249967
Rabggta	0.249963
Cdc42	0.249951
Glrx2	0.249884
Snrnp48	0.24985
Rcn2	0.249811
Fam57a	0.24979
Stambp	0.249768
Txlnb	0.249766
Lyl1	0.249745
Brsk2	0.249679
Smyd5	0.249647
Mapre2	0.249632
Ldlrad4	0.249601
Krt8	0.249592
Hoxd9	0.249584
Haus7	0.249581
Gtf2b	0.249558
Nf2	0.249544
Mlf2	0.249529
Slitrk3	0.249465
Tpx2	0.249465
Sppl3	0.249442
Myl7	0.249439
Fgfr4	0.249425
Qars	0.249367
Ube2r2	0.249359
Zfp61	0.249338
Elavl1	0.249303
Evpl	0.249293
Fosl1	0.249237
Arhgef17	0.249204
Map4k3	0.249155
Lrrc25	0.249095
Foxq1	0.249088

Vsx2	0.249032
Rinl	0.248959
Mthfd1	0.248948
Pfkfb4	0.248948
Aebp1	0.248925
Arl5b	0.248918
Oxa1l	0.248842
Cbr3	0.248806
Rrn3	0.248795
Ino80	0.24879
Bcan	0.248771
Ppm1a	0.248771
Crx	0.248723
Mfsd7c	0.24866
Cep128	0.248596
Ahctf1	0.248582
C2cd4c	0.248581
Slc31a2	0.248506
1810055G(	0.248503
Lrp12	0.248472
Slc37a1	0.248445
Ndufv3	0.248376
Ppp4r4	0.248375
Rbp7	0.248362
Tigd2	0.248359
Plcg1	0.248342
Gpr182	0.248323
Wapal	0.248307
Slc17a5	0.248266
Cry1	0.248165
Thrb	0.248152
Vbp1	0.248146
Ncaph	0.248129
Clk3	0.24812
Gmeb2	0.248111
Spdya	0.248108
Nmrk2	0.248099
Rasl12	0.248098
Mdm2	0.248065
Cachd1	0.247954
Atg4c	0.247943
Cep120	0.247906
Gmpr	0.247891
Cdc14b	0.247885
Grid2	0.247867
Fam212b	0.247865
Nebl	0.24785

Mdm1	0.247845
Lim2	0.24783
Ttc5	0.247829
Ffar2	0.247779
E2f1	0.247774
Stx4a	0.247696
Dffa	0.247682
Lamp5	0.247677
Fhod3	0.247675
Tmem109	0.247515
Pgd	0.247465
Vdac3	0.247432
Afap1l2	0.247431
Casp6	0.247363
Ankrd2	0.247343
Usp8	0.247327
Samd8	0.247266
Ccdc62	0.247199
Elmod1	0.247183
B3galt5	0.247177
Fbxl7	0.247165
Casc5	0.247149
5031414D1	0.247093
Rmi2	0.247055
Crispld2	0.247052
Atp5a1	0.24705
Mum1	0.24705
Arhgap19	0.247041
Trabd2b	0.247037
Tmem159	0.246994
Fbxo3	0.246872
Dpp9	0.24686
Pex5	0.246857
Ccnl1	0.24682
Syt7	0.246771
Inppl1	0.246739
Mt3	0.246736
Treml4	0.246701
Zfp94	0.246688
Kcnj8	0.246625
Mbd3	0.246539
Tnfrsf13c	0.246457
Taf1d	0.246453
Rpl14	0.246414
Ube4a	0.246408
Spz1	0.246389
Sacs	0.246378

Grm6	0.246322
Serinc5	0.246322
Frem1	0.246236
Pdhb	0.246229
Fam169a	0.246161
Hadh	0.24614
Serinc2	0.246092
Plau	0.246019
Ap1g1	0.246002
Cd276	0.245944
Smyd2	0.245919
E2f3	0.245878
Qrfpr	0.245671
Glis1	0.245647
Galnt7	0.24562
Sulf2	0.245618
Mppe1	0.245512
Utp23	0.245488
Kcnn3	0.245432
Lamtor3	0.24533
Shf	0.245226
Wfdc2	0.245217
Ubc	0.245216
Galk1	0.245214
Arl4d	0.245189
Bloc1s5	0.245157
Edil3	0.245116
Gnai3	0.245095
Tef	0.245043
Cpox	0.245029
Gatad1	0.245016
Gtf2f2	0.245015
Mtpap	0.24499
Gm1673	0.244987
Dusp1	0.244954
Asxl1	0.244951
Mrpl23	0.244945
Serinc1	0.244911
Odf2l	0.244881
Tmem121	0.244863
Dhx40	0.244834
Plch2	0.244818
Hopx	0.244817
Ntn5	0.244815
9930012K1	0.244785
Tcea2	0.244687
Fam63b	0.244687

Ap2s1	0.244671
Map1b	0.244668
Slc26a1	0.244668
Krt5	0.244637
Fbxw9	0.244608
Slc39a1	0.24456
Elmo1	0.244538
Rab40c	0.244495
Uqcr11	0.244472
Txn1	0.244467
Mpg	0.244414
Ccdc117	0.244378
Utp11l	0.24437
Zmiz2	0.244336
Pnrc1	0.244332
Pdss1	0.244314
H2afy3	0.244308
Napg	0.244289
Spata25	0.244203
Foxf1	0.244119
Poglut1	0.244072
Phf8	0.244049
Ccdc11	0.244038
Pros1	0.24403
Tmem117	0.243903
Cdsn	0.243893
Kif19a	0.243855
Mlc1	0.243822
Pabpn1	0.243799
Ivns1abp	0.243784
Sar1a	0.243773
Ankrd9	0.243756
Cdkn2c	0.24369
Gdf6	0.243645
Rap1b	0.243607
Amer2	0.243569
Pop4	0.243552
Eci1	0.24354
Dctpp1	0.243523
Bri3	0.243495
Wnt9b	0.243472
Rcan2	0.24344
Psg16	0.243402
Cabp2	0.243218
Synj2bp	0.243132
Mgrn1	0.24304
Rab3b	0.243024

Bhlhe40	0.242988
Itpr2	0.242963
Lemd3	0.242948
Cbln2	0.24294
Cul2	0.242937
Il11	0.24291
A330021E2	0.2429
C1qc	0.242857
Zfp263	0.242819
Mks1	0.242815
Cog7	0.242808
Ier5	0.242792
Cbx1	0.242684
Dennd4a	0.242653
Ddx21	0.242607
Nfam1	0.242588
Glt1d1	0.242561
Ncam2	0.242504
Zmym6	0.242482
D17Wsu10	0.242437
Lrfn4	0.242423
Gpr126	0.24239
Rnf144a	0.242288
Itgav	0.242276
Smagp	0.242251
Rnf39	0.242251
Osmr	0.242153
Rbms1	0.242145
Med15	0.242131
Slc27a4	0.242127
Gldc	0.242052
Ubxn2a	0.24201
Slc25a51	0.242001
Spsb1	0.241948
Klhl25	0.241903
Fam184b	0.241884
Rpl27	0.241871
Cwc25	0.241792
Meis3	0.241781
Phrf1	0.241772
Tmx4	0.241772
Ascl1	0.241721
Foxi1	0.241649
Nr4a2	0.241626
Paip2	0.24153
Slc38a4	0.241472
Gcnt2	0.241225

Pdlim2	0.241206
Parvg	0.241056
Pld6	0.241021
Hist1h1c	0.241004
Rbfa	0.240979
Map2k7	0.240962
Rbpj	0.240959
Slit1	0.240915
Tm9sf1	0.24089
Aldh7a1	0.240868
Lmtk3	0.240847
Tmem125	0.240779
Gtf2i	0.240564
Eno2	0.240548
Snx18	0.240477
Orai3	0.240454
Gan	0.240375
Tnni3	0.240318
Dpep3	0.240312
Gna11	0.240292
Zfand2a	0.240224
Rps4l	0.240221
Man2c1	0.240204
Idnk	0.240193
Aarsd1	0.240173
Mrgpre	0.240149
Rps29	0.240138
Eif4ebp2	0.240101
Psmd7	0.240087
Rab3gap1	0.240053
Wls	0.240013
Lamp2	0.239979
Gadd45a	0.239963
Wdr19	0.23995
Cdh1	0.239946
Coq10a	0.239917
Dhodh	0.2399
Bdnf	0.239888
Pcdh20	0.239845
Cyp4f15	0.239816
Ado	0.239815
Ccdc32	0.23981
Ptch2	0.239764
Rnf157	0.239744
Dixdc1	0.239708
Cry2	0.239691
Hsbp1	0.239679

Ctnna2	0.23967
Rbbp6	0.239669
Cuedc1	0.239545
Bin3	0.239495
Zfp764	0.239446
Mrps35	0.239445
Lbx2	0.239386
Aldh18a1	0.239338
Nr2c2ap	0.239169
Rpia	0.239062
Sh2d5	0.239057
Lhpp	0.239043
Rad54l	0.238986
BC030867	0.238984
Iqgap2	0.238962
Hps6	0.238947
Sel1l	0.238919
Specc1	0.238912
Ppp2r1a	0.238901
Hecw2	0.238858
Zc3h12c	0.238833
Frat1	0.238815
Lrtm2	0.238805
Acer3	0.238765
Cbwd1	0.238734
Nolc1	0.238684
Zfp367	0.238649
Gm1587	0.23859
Cnrip1	0.238587
Tspan12	0.238547
Plod1	0.238509
Calb2	0.238509
Taf3	0.238474
Slc35f5	0.238435
Zfp780b	0.238407
Zfp512	0.23839
Elovl1	0.238361
Traf4	0.23836
Cars	0.238313
Rabepk	0.238304
Bag3	0.238295
Hdac7	0.238276
Lurap1l	0.238241
Tubgcp3	0.238149
Npas4	0.238081
Cltb	0.238052
Foxc1	0.238016

Clk2	0.237952
Myo15	0.237912
Lsm12	0.237875
Gfpt2	0.237859
Sidt1	0.237827
Pcif1	0.237826
Pou5f1	0.237815
Wee1	0.237768
Kptn	0.237731
Kat5	0.237707
Slc25a1	0.237685
Ppp6r2	0.237666
Depdc1b	0.237634
Exosc10	0.23761
Hps1	0.237465
Dtx1	0.237431
Cnpy3	0.237428
Pwp1	0.237395
Vstm5	0.237386
Ikzf3	0.237348
Fgfrl1	0.237311
Prss8	0.237233
Ubn2	0.237175
Rbm28	0.237099
Ip6k1	0.237045
Rasgrp2	0.236986
Tbl3	0.236946
Oxct2a	0.236867
Gsr	0.236853
Trip6	0.236849
Hvcn1	0.236813
Zfp322a	0.236752
Zfp110	0.236728
Tfcp2	0.236667
Adcy5	0.236664
Rgs10	0.236604
Fxyd5	0.236591
Med13l	0.236548
Gfpt1	0.236502
Kcnk10	0.236491
Frat2	0.236458
Nceh1	0.236398
Nagpa	0.236361
Wdr59	0.236354
Gm14137	0.236347
Kbtbd13	0.236296
Gjc2	0.236179

Dmrta1	0.236161
Mprip	0.23608
Chst3	0.236014
Add3	0.236008
Nfe2l2	0.235998
BC003965	0.23599
Krcc1	0.235959
Rbm15b	0.23595
Gk5	0.235913
2010012O(	0.235907
Srrm2	0.235817
Rspo1	0.235771
Pld2	0.235768
Gpd1l	0.235755
Sbsn	0.235739
Ddi2	0.23573
D17H6S53I	0.235701
Sh2d4a	0.235697
Hspa5	0.23569
Tmem231	0.23569
Tor3a	0.235646
Rorb	0.235633
Usp2	0.235598
Tgm5	0.235554
1700112E0	0.235535
Tmem92	0.235479
Adrbk2	0.235455
Ddx50	0.235444
D17Wsu92	0.23543
Cep110	0.235396
Mrps10	0.23534
Csnk1d	0.23533
Sh2d3c	0.235318
3110043O2	0.235242
Dctd	0.235185
Ran	0.235147
Syn3	0.235095
Pcsk2	0.235091
Smarca2	0.23508
Ints10	0.235
Nupr1l	0.234967
Fam203a	0.23493
Gpm6a	0.234858
Zfp182	0.234852
AI661453	0.234782
Zfp513	0.234761
Dhrs7b	0.23475

Gfod2	0.234722
Rcl1	0.234664
Ythdc1	0.234653
St8sia2	0.234644
Tm6sf1	0.234617
Gpsm2	0.234615
Gm6484	0.234585
Zc3h12a	0.234551
Atf7ip	0.234547
Klhl2	0.234543
Zfp827	0.234539
Vezf1	0.2345
Tut1	0.234396
Il17rc	0.234316
Smg9	0.234257
Cdk18	0.234257
Rab40b	0.234247
Tbc1d2b	0.234177
Uevld	0.234101
Cmpk2	0.234096
Cav1	0.233999
Adarb2	0.233991
Rp9	0.23397
Mrps31	0.233933
Tm6sf2	0.233911
C1galt1	0.233904
Dok5	0.233861
Capns1	0.233832
Alx4	0.23383
Actr8	0.23379
Lmf1	0.23378
Apitd1	0.233742
Cenpw	0.233719
Ltc4s	0.233681
Msmp	0.233649
Map2	0.233617
Ift52	0.233579
Tmem106b	0.233579
Ppp1r3c	0.233423
Ccdc34	0.233398
Tram1	0.233343
Ppp1r3g	0.233321
Rnaseh1	0.233272
1700029M	0.233239
Ahsa2	0.233204
Pde6d	0.233198
Ttf2	0.23318

Ripk2	0.23317
Pcsk6	0.23317
Gpatch8	0.233164
Ncoa7	0.233128
Asf1a	0.233055
Pcsk1n	0.23303
Zc3h7b	0.232929
Tmem222	0.232888
Pinx1	0.232882
Gjc1	0.232874
Dad1	0.23284
Igf2bp2	0.232834
Slc40a1	0.232784
Manbal	0.232757
Lingo3	0.232755
Mocos	0.232726
Zranb2	0.232703
Erich1	0.232663
Ttll12	0.232637
Lhfp	0.232569
Scara3	0.232549
Oxct2b	0.232528
D8Ertd738	0.232511
Dut	0.232505
Micu2	0.23249
Tpd52	0.232462
Slc30a2	0.232459
Gcn1l1	0.232377
Cast	0.232373
Ube2l3	0.232355
Trp53bp1	0.232341
Asic2	0.232341
Nrip1	0.232324
Rgs7	0.232274
Pla2g7	0.232183
Dlgap3	0.232174
Prpf8	0.232174
Rhebl1	0.232158
Cd1d1	0.232114
Dhdds	0.232051
Far1	0.232005
Tmem216	0.231976
Pyy	0.231961
Etaa1	0.23194
3110057O	0.231848
Pnpla3	0.231848
Spg7	0.231818

Frmd3	0.231816
Zufsp	0.23181
Tbx1	0.231795
Crebzf	0.231784
Rcn3	0.231784
Slitrk5	0.231781
Zbtb21	0.231773
Cers5	0.231727
Ankrd39	0.231692
Pts	0.231691
G3bp2	0.231683
Ube2n	0.231669
Lefty1	0.231668
Pnpla8	0.231658
Arrb1	0.23163
Atp2b3	0.231567
Esrrg	0.231523
Ncapg	0.231502
Dach1	0.231499
Wdr5	0.231481
Selenbp1	0.231421
Mthfd1l	0.231375
Colec12	0.231363
Epor	0.231363
Grhl3	0.231345
Abcf1	0.231344
Srsf9	0.231334
Atp1a3	0.231332
Nfasc	0.231328
Wwp1	0.231312
Zhx1	0.231304
Baz1a	0.231225
Exoc6b	0.231085
Ccp110	0.231085
Trmt44	0.231078
Med26	0.231064
Agtpbp1	0.231062
Nemf	0.231057
Aif1l	0.231026
Ucn2	0.231012
Tmem248	0.230974
Gm2a	0.230955
B4galt7	0.23093
Fut8	0.230899
Glcci1	0.230856
Kti12	0.230855
Arhgap18	0.230812

Zfyve20	0.230809
Zfp677	0.230762
Tcp11	0.230709
Zfp418	0.230698
Spata16	0.230678
Sptlc2	0.230668
Tmem80	0.230637
Dot1l	0.230632
Tysnd1	0.230629
Sec24b	0.230628
Hectd1	0.230619
Fam220a	0.230608
Eri1	0.230504
2300009A(	0.230374
Ubl7	0.230343
Ppp1r12c	0.230318
Gnb1	0.230306
Tmem246	0.23027
Fam168b	0.230203
Wdr61	0.229975
Bzw1	0.229967
Sf3b1	0.229925
Fus	0.229923
Mmadhc	0.229884
Pole2	0.229863
Angpt1	0.229859
Slc12a8	0.229848
Mybl2	0.229807
Aldoc	0.229791
Spns1	0.229777
Tbcel	0.229693
Arid4a	0.229679
Zfp874a	0.229657
Susd2	0.229618
Ylpm1	0.229544
Sin3b	0.229458
1700010I1·	0.229445
Gphn	0.229352
Egfr	0.229351
Acss1	0.229339
Edrf1	0.229288
Elmod2	0.229238
Figla	0.229236
Enpp7	0.229177
Lnp	0.229171
Diexf	0.229104
Uhrf1bp1l	0.229066

Prrc2b	0.229052
Ndufs4	0.229021
Rasal1	0.229007
Dnm2	0.229001
Zfp846	0.228951
Numa1	0.22895
Lmnb1	0.228948
Slc20a1	0.228942
Slc25a34	0.228932
Cdk5rap1	0.228856
Aldh4a1	0.228835
Rmdn1	0.228827
Dtd1	0.228779
Myo1a	0.228769
Fmo5	0.228736
Psmb7	0.228719
Rian	0.228682
Wrnip1	0.228628
Prokr2	0.228555
Olfr550	0.228532
Reps1	0.228511
Anks3	0.2285
Clmn	0.228498
Axin2	0.228439
Lat2	0.228428
Kitl	0.22842
Smpd4	0.22833
Fam210b	0.228328
Irf2bp1	0.228288
Pdgfd	0.228258
Fam115c	0.228246
Dock10	0.228222
Fam213b	0.228204
Sec14l4	0.228095
Adra2a	0.228064
Slc35f2	0.227951
Vps53	0.227927
Zpbp2	0.227905
Ldb1	0.227885
Ndc80	0.227866
Egr1	0.227858
Fgfr3	0.227853
Klhl21	0.227807
Tgfb1	0.227793
Mesdc1	0.227775
Klk9	0.227747
Oplah	0.227741

Trim71	0.227727
Lrrc16a	0.227719
Tmem243	0.227712
Rdh13	0.227708
Jag2	0.227658
2610528J1	0.227613
Btbd2	0.227587
Fmnl1	0.227554
Man1a	0.227531
Cmtr1	0.227513
Papss1	0.22749
Dph2	0.227469
Lrrc9	0.227413
Sft2d1	0.227403
Ero1l	0.227375
Bcl3	0.227352
Cnot11	0.227318
Rnf150	0.227296
Mpc1	0.227291
Ccdc125	0.227272
Psapl1	0.22724
Nmnat2	0.227176
Akap17b	0.227144
Bst2	0.227108
Smad2	0.227097
Cnot6	0.227073
Zer1	0.227067
Serp2	0.227039
Sdc1	0.227023
Nkx2-4	0.227012
Atp8b1	0.226936
Cpz	0.226932
Gem	0.226898
Mkrn1	0.226897
Sgcd	0.22689
Zfp787	0.226887
Piezo2	0.22688
Tmem184c	0.226875
Lman2	0.226854
Uqcrc1	0.226853
Akr1e1	0.226815
Rasa4	0.226779
Rps9	0.226704
Aldh3b1	0.226697
Hoxd8	0.226685
Pgap2	0.226651
Ackr4	0.226628

Olfr317	0.226581
Mtmr12	0.226562
Gm17296	0.22652
Ptpla	0.226443
Eif2s2	0.22635
Agtrap	0.226333
A530054K1	0.226289
9130011E1	0.226267
Rmdn3	0.226231
Fam135a	0.226212
Hic1	0.226198
Ptplb	0.226073
Sun1	0.226033
Pdgfc	0.225988
Nr2f1	0.225942
D630029K0	0.225904
Gulp1	0.22587
Zfp281	0.225862
Mc4r	0.22586
Nt5c3	0.225833
Mtf2	0.225786
Dusp11	0.225784
Iqsec1	0.225764
Rufy3	0.225657
Dynll2	0.225652
Ttc39b	0.225651
Crhbp	0.225643
Lbx1	0.22564
Smim5	0.225593
Map7d1	0.225577
Glb1l2	0.225564
Cnnm3	0.225534
Itga7	0.225523
Jmy	0.225514
Nrip2	0.225468
Arih2	0.225331
H2-Q4	0.225231
Gipc2	0.225215
Ddt	0.225209
Tle2	0.225198
Klk8	0.225104
Bloc1s6	0.225058
Srsf10	0.225044
Zfp114	0.225039
Aldh1l1	0.224978
Dusp8	0.224976
Ociad2	0.224911

Plk4	0.224898
Adamts8	0.224858
Clip2	0.22483
Spi1	0.224828
Cdk1	0.224749
Armc1	0.224704
Kdm3a	0.224684
BC030476	0.224659
Gpr146	0.224618
Zfp280d	0.224596
Slc30a10	0.224557
Sp6	0.224346
Cplx3	0.224342
Srm	0.224341
Slc9b2	0.224318
Hcn1	0.224301
Med1	0.224158
Wdfy1	0.224157
Ass1	0.224152
2310014L1	0.224137
Prkaca	0.224091
Edaradd	0.224017
Ap4e1	0.224009
Pgbd1	0.223991
Nek1	0.223973
Cdkn2b	0.223936
Tspan32	0.223925
Insm2	0.223903
Uggt1	0.223878
Wipf1	0.223873
Tmem194	0.223844
Cecr6	0.223773
Mchr1	0.223761
Rtn4	0.223739
Fbxo31	0.223731
Klf10	0.223715
Pisd	0.223679
Acvr1c	0.223651
Nek6	0.223627
Bbs1	0.223574
D14Abb1e	0.223563
Jph3	0.223535
Kcna7	0.223488
Gsg1	0.223464
Nsun6	0.223455
Zfp451	0.223442
Plcb3	0.223435

Gab1	0.223408
Lyn	0.223392
Nsg2	0.223345
Lrrc3	0.223334
Hipk4	0.223332
Fkbp2	0.223315
Gpc5	0.223299
8-Sep	0.223235
Ppy	0.223168
Calb1	0.223133
AW209491	0.223118
Gstk1	0.223109
Sgip1	0.223024
Chchd3	0.222991
Rec8	0.222938
Tpsb2	0.222893
Slc4a7	0.222888
Cldn19	0.222887
Stxbp3a	0.222775
Fgfr1op	0.222608
Pard6b	0.222572
Zc3hc1	0.222561
Slc12a5	0.222557
Ing4	0.222529
Phox2a	0.222506
Syf2	0.222484
Cers2	0.222434
Pdlim3	0.22243
Pdcd2	0.222393
Prpf18	0.222363
Tnpo1	0.222362
Ttpa	0.222317
Plin5	0.222303
Tdrp	0.22222
Smap2	0.222131
Limk1	0.222082
Tmem65	0.222054
Cdh5	0.222026
Lpcat2	0.222025
Adra1d	0.222009
Sppl2a	0.221917
Cacna1s	0.221837
Ch25h	0.221784
2410016O(	0.221782
Nudt17	0.221771
Sucla2	0.221759
Cdk2ap1	0.221735

Cd72	0.221724
Fbxo10	0.221686
Apba1	0.221682
Pam	0.221678
Snap91	0.221672
Cacna1c	0.221606
BC030336	0.221599
Aqp11	0.221478
Myef2	0.221402
Grid2ip	0.221368
4933416C0	0.221275
Ank2	0.221261
Mcmbp	0.221255
Tmem101	0.221242
Rcc2	0.22122
C1qtnf1	0.2212
Tnfrsf19	0.221125
Psmb4	0.221117
Baalc	0.221093
B3gnt2	0.221085
Fam105a	0.221053
Rab11a	0.221034
Vwa9	0.220936
Rpa2	0.22092
Ehd1	0.220914
Zkscan2	0.220905
Dgkd	0.22089
Rcor1	0.220733
Zfp2	0.220696
Fbn2	0.22065
Rbm22	0.220642
Msra	0.220623
Nbeal1	0.220562
Ehd4	0.22051
Dnajb2	0.220478
Trim16	0.220446
Anxa4	0.220429
Plbd2	0.220379
C1d	0.220361
9430038I0	0.22034
Ocrl	0.220316
Shq1	0.220312
4921524J1	0.220296
Klhl11	0.220294
Tgfb2	0.220281
Tomm7	0.220266
Pvr	0.220257

Rxra	0.220234
2-Mar	0.22022
Socs7	0.220209
Fbxl18	0.220207
Nmnat3	0.22011
Slc38a2	0.220017
Slc25a37	0.219974
Gprc5b	0.219936
Pkd1l2	0.219912
Phf1	0.219896
Fam222a	0.219884
Edc3	0.219858
Asphd2	0.219848
Smad5	0.219848
Polr2m	0.21984
Pcbp2	0.219826
Abcc12	0.21977
Exo1	0.219727
Galnt11	0.219705
Ccdc85c	0.219673
Epb4.1	0.219647
Mybpc3	0.219593
Farp1	0.219593
Nipsnap1	0.219576
Tnks1bp1	0.219538
Dym	0.219421
Ptplad1	0.219421
Col6a1	0.219342
Bach1	0.219301
Limch1	0.21924
Osbp2	0.219173
Stambpl1	0.219161
Oxr1	0.21915
Pgrmc2	0.21914
Vti1b	0.219131
Eri3	0.219057
Sema3b	0.219037
Bfsp2	0.219018
Dync1li1	0.219009
Synj2	0.218986
Casd1	0.218941
Sycn	0.218886
Abcd4	0.218871
Aff1	0.218849
Rmnd5a	0.218813
Upk1a	0.218804
Ptp4a3	0.218678

Fam58b	0.218661
Nrf1	0.218653
Spice1	0.218643
Kif1a	0.218621
Kcnk9	0.218618
Kcnv1	0.218616
Alox12	0.218613
Depdc5	0.218554
Ncoa4	0.218535
Rusc1	0.218462
St18	0.218461
Klhdc1	0.218429
Pex19	0.218425
Sun2	0.218386
Smarca5	0.218374
Zfp36l2	0.218343
Nfic	0.218343
Zglp1	0.218301
Agbl4	0.21828
Terc	0.21826
Pycr2	0.218197
Tsn	0.218173
Dleu7	0.218092
Tcte1	0.218059
Kat6a	0.21803
P2rx5	0.217997
Trpm5	0.217911
Ccdc83	0.21785
Ttll7	0.217842
2900097C1	0.217839
2010107E0	0.217832
Adck4	0.21783
Cr1l	0.217762
Thoc7	0.217762
Mcl1	0.217731
Dpm2	0.217698
Man2a1	0.217618
Intu	0.217579
Thap1	0.217551
Dnah1	0.217531
Avpr1a	0.217522
Slc7a1	0.21749
Atp6v0a1	0.217473
Tsen2	0.217407
Mycbp2	0.217366
Ppargc1b	0.217354
Mgat3	0.21735

Lin7a	0.217322
Galnt15	0.217308
Cpxm1	0.217296
Nop58	0.217223
Bmpr2	0.217194
Esr2	0.217157
Spr	0.21713
Ndufc1	0.217129
Meiob	0.217061
Limd2	0.217059
1810013L2	0.217046
Cacfd1	0.216968
Ankrd50	0.216912
Rab1	0.216872
Erich2	0.216871
Rprd1a	0.216835
Gipc3	0.216827
Zc3h15	0.21682
0610012H(	0.216772
Hic2	0.216666
Tmeff1	0.21666
Chrnb1	0.216649
Ly6g5c	0.216643
Tubg2	0.216597
Spast	0.216553
Coa4	0.216464
Coq2	0.216386
Mrpl55	0.216369
Zfp523	0.216321
Flt4	0.216308
Arnt2	0.21625
Polr2d	0.216144
H2-T3	0.216108
Eya1	0.216081
Eea1	0.21607
Spty2d1	0.216041
Ptk2	0.215975
Zfp280b	0.215923
Gab2	0.215919
Fut2	0.215841
C1galt1c1	0.21584
Rhbdf1	0.21584
Pygo1	0.215775
Sec31a	0.215749
Fsd1l	0.215731
Slc7a10	0.215681
Commd10	0.215681

Rrp7a	0.215663
Olfml2b	0.215562
Fmnl3	0.215546
Fam19a5	0.215501
Caps2	0.215475
Slc25a28	0.215471
Ehbp1	0.215439
Jagn1	0.2154
Ercc4	0.215335
Krr1	0.215293
Zc3h7a	0.215229
Dhx30	0.215211
Slco3a1	0.215165
Ubash3b	0.215135
Sil1	0.215112
Atp11a	0.215091
Sox8	0.214985
Mt1	0.214899
Mycbpap	0.214876
Slc9a3r2	0.214862
N4bp1	0.214859
Pisd-ps2	0.214847
Trafd1	0.214821
Psmd2	0.214734
Dennd5b	0.21472
Zfp628	0.214649
Kat6b	0.214646
Kcnq5	0.21459
Gspt2	0.214588
Kctd13	0.214554
Ryr2	0.214531
Ect2	0.21441
Ppp4c	0.214389
Mul1	0.214372
Dync1h1	0.214367
AI429214	0.214364
Dgcr6	0.214341
Zfp40	0.214293
Pabpc1	0.214277
Trh	0.214272
Htra1	0.214147
Mafa	0.21414
Ube2g1	0.214129
Olfr646	0.214045
Trmu	0.214038
Galr1	0.21396
Wdr86	0.213942

Snx14	0.213877
Zfp37	0.213822
Rpp30	0.213821
Sptb	0.213804
Papola	0.213777
Hdac1	0.213775
Efnb1	0.213735
Tmem86a	0.213664
Arhgef19	0.213621
Rims2	0.213594
Abr	0.213582
Lctl	0.213581
Rtbdn	0.213557
Arpc1a	0.213492
Flad1	0.213489
Ndst2	0.213373
Ccdc158	0.213309
Snca	0.213232
Zfp82	0.213227
Ilf2	0.213204
Tomm20	0.213199
Hspa12a	0.213187
Tlk1	0.21317
Satb1	0.213149
Adcyap1r1	0.213132
Tmem126a	0.213115
Rac1	0.213104
Elavl2	0.213102
Slco2a1	0.213088
Obfc1	0.213056
Pou6f1	0.213045
Tapt1	0.212987
Pou2f3	0.212955
Atp6v1c1	0.212933
Camk2n2	0.212883
Vprbp	0.212856
Nrep	0.212802
Slc22a12	0.212793
Pkd1	0.212784
Diap1	0.21276
Xkr4	0.212697
Cbx2	0.212695
Zfp74	0.212693
Ppp2r2c	0.212639
Ttc1	0.212571
Dyrk1a	0.212532
Gpr124	0.21246

Gm6787	0.212432
Pcsk9	0.212414
Mccc2	0.212386
Znrf4	0.212356
Wdr1	0.212346
Rtn4r	0.212257
Paxip1	0.212236
4930453N2	0.212233
Gpd2	0.212221
Dgat2	0.212194
Zfp69	0.212192
Plxna3	0.212139
Clic3	0.212131
Cdk5r2	0.21211
Crybb2	0.212068
Zfp947	0.212052
Osm	0.212029
Uhrf2	0.212026
Proz	0.211974
Os9	0.211908
Ptcra	0.211896
Zfp326	0.211883
Fchsd1	0.211846
Otud6b	0.211842
Ntf5	0.211839
Myl12a	0.211824
Sgcz	0.21182
Lrfn2	0.211816
Orc5	0.211812
Ndufb2	0.211806
Ankrd13d	0.211769
Krt7	0.211677
Usp4	0.211643
Srbd1	0.211629
Trim41	0.211621
Parm1	0.211602
Ccdc89	0.211567
Kncn	0.211557
Rbm19	0.211503
Vkorc1	0.211465
Col6a2	0.211412
Palm3	0.211412
Mkrn2	0.211357
Kcnk3	0.211333
Med27	0.211333
Wdfy3	0.211262
Zswim5	0.211247

Mtf1	0.211192
Qrich2	0.211168
Zdhhc1	0.211109
Rcan3	0.211108
Acy3	0.211104
Nit2	0.211099
Gstp1	0.211068
Smad6	0.211004
Nfxl1	0.210978
Faim2	0.210974
Bves	0.210962
Zscan26	0.210919
Grhl1	0.210912
St3gal6	0.210911
Vangl2	0.21091
Cmc1	0.210886
Sox1	0.21088
Tacc1	0.210865
Wwp2	0.210855
Parp11	0.210749
Trim26	0.210714
Kmt2a	0.210685
Dnajc3	0.210674
Ipo11	0.210671
Zfp426	0.21061
Adss	0.210593
Rbm11	0.210547
Polr1b	0.210469
Hipk2	0.210457
Ppic	0.210386
Pth1r	0.210366
Mapk1ip1l	0.210232
Cbfb	0.210178
Slc35f6	0.21017
Ndfip2	0.210148
Cdcp2	0.210133
Nell1	0.210105
Nrsn2	0.210029
Heatr5a	0.210027
Cpeb1	0.210001
Pou2f1	0.209957
6330416G1	0.209942
Pbdc1	0.209872
Fermt3	0.209856
Fgf3	0.209841
Ercc2	0.209826
Bai2	0.209799

Klhl32	0.209778
Lmo1	0.209719
Impa1	0.209695
Brms1l	0.209694
Timm22	0.209662
Ripk3	0.209613
Sec61a1	0.209548
Plxna1	0.209493
Klhl14	0.209468
Fam105b	0.209466
Nacc2	0.209463
Zc3h8	0.209433
Aqp2	0.209415
6430531B1	0.209395
2610008E1	0.209375
Ctdspl	0.209341
Prss22	0.209239
Aacs	0.209222
Lca5l	0.209221
Col5a1	0.209186
Icam2	0.209172
Fcho2	0.209159
Sfr1	0.20915
Cda	0.209147
Sez6l	0.209127
Rnf207	0.209114
Tlr12	0.209061
Dsn1	0.209017
Dvl3	0.208996
Kcnh1	0.208974
Ephb2	0.208923
Ugp2	0.208872
Pitpna	0.208868
Hmx1	0.208863
Clint1	0.208809
Lgals8	0.208807
Lrrc23	0.208651
Cox8b	0.20858
Rab10	0.208531
Nme4	0.208518
Exosc2	0.208512
Slmo2	0.208509
Zfp420	0.208506
Pes1	0.208499
Yif1b	0.208481
Abat	0.208468
Clybl	0.208448

Ctsb	0.208407
Setmar	0.208376
Net1	0.208285
Laptm5	0.208279
Zfp592	0.208264
4933426M	0.208215
Dhx37	0.208167
Tmc5	0.208133
Pip5k1b	0.208071
Pth2r	0.207989
4930550C1	0.207977
Stxbp5l	0.207974
Camk1d	0.207947
Arsi	0.207903
Snx3	0.20787
Ints8	0.207864
Cdh4	0.207849
Abca2	0.20784
Kank4	0.207814
Myh10	0.20773
Fxn	0.207656
Pdpk1	0.207637
Scpep1	0.207636
9430015G1	0.207632
Slc18b1	0.207593
H2-Ab1	0.207551
Scrn2	0.20748
Ppara	0.207464
Renbp	0.207415
Wdr48	0.207402
Sema6c	0.207346
Hmces	0.207334
Hnrnpr	0.207305
Metrnl	0.207284
Fam49b	0.207277
Smim12	0.207248
Shank3	0.207243
Csrnp2	0.207177
Otud4	0.207102
Ephx2	0.207095
Rspo2	0.207091
Aggf1	0.207044
Fam13b	0.20703
Tbx4	0.206988
Tmem261	0.206985
Atp6v1e1	0.206937
Pogz	0.206935

Cd37	0.206925
Numbl	0.206914
Sergef	0.206911
Cct4	0.206902
Creg1	0.206901
Zfp617	0.206898
Smchd1	0.206893
Diras1	0.206883
2310007B0	0.206877
Npm1	0.206863
Nol6	0.206854
Paqr9	0.206791
1700018B0	0.206791
Socs5	0.20675
Sptbn2	0.206696
Vasp	0.20668
Cadps2	0.206678
Vps18	0.206662
Uaca	0.206657
Wdr7	0.206656
A230046K0	0.206649
Fgf8	0.206638
Trmt10c	0.206598
Fis1	0.206597
Snai2	0.206585
6720401G1	0.206582
Mfhas1	0.206544
Sppl2c	0.2064
Mpp6	0.206344
Slc4a4	0.206258
Wfs1	0.206256
Cnot6l	0.206253
D730001G1	0.206239
Hhatl	0.206236
Hdac6	0.206233
Ace	0.206216
B430306N0	0.206195
Ccdc142	0.206192
Apoa1	0.206117
Rffl	0.2061
Myod1	0.206088
Srf	0.206084
Itpk1	0.20604
Srprb	0.206029
Chd2	0.205969
Syngr2	0.205894
Cep78	0.205837

Tns4	0.205824
Smad4	0.205809
Stpg2	0.205773
Fnip2	0.205766
S1pr3	0.205733
Map3k6	0.205693
Ctnnb1	0.205652
Zfp866	0.205632
Rorc	0.205609
Zic2	0.205567
Mcat	0.205543
Tmem214	0.205443
Ints5	0.205422
Wbp1l	0.205402
Itga1	0.205373
Cntnap5a	0.205343
Ncald	0.205319
Omp	0.205201
Syt10	0.205185
Vpreb3	0.205179
Ptpn4	0.205137
Crhr1	0.205102
Nup160	0.205069
Morc3	0.204954
Minpp1	0.204942
Rnf111	0.204895
Scube3	0.204868
Tecpr1	0.204858
Lysmd2	0.204573
Mxra8	0.204573
Ppp3ca	0.204565
Chrna7	0.204565
Wdr36	0.20456
Spopl	0.204538
Pnp2	0.204504
Larp1	0.204494
Wisp1	0.204435
Capn5	0.204407
Dr1	0.20438
Zcchc14	0.204352
Dse	0.204352
Twistnb	0.204348
Mboat2	0.20422
Camk2g	0.204164
Mb21d1	0.204155
Zfp612	0.204115
Layn	0.204062

Lrg1	0.204013
AI314180	0.203997
Cela1	0.203981
Zbtb14	0.203955
Endov	0.203922
Klc3	0.203911
Ssbp1	0.203892
Abca15	0.203848
Vrk2	0.203815
Bbs5	0.20377
Nrxn2	0.203758
Gpr62	0.203727
Actr2	0.203698
Pbxip1	0.203588
Podnl1	0.203534
Atp10a	0.203514
Stard7	0.203502
Zfp369	0.203481
Dennd1a	0.203372
Ict1	0.203257
Zfp251	0.203226
Gm1943	0.203198
Sowahb	0.203195
Acyp2	0.203166
Dcaf5	0.203081
Mroh1	0.203051
Pex11b	0.202983
Nqo1	0.202941
Fbxl16	0.202931
Adamts19	0.202895
Iba57	0.202848
Fndc3c1	0.202819
Stx17	0.202813
Cryaa	0.202797
Zfp445	0.202788
Odc1	0.202786
Tmem206	0.202777
Mamld1	0.20277
Rasgrp1	0.202716
Arl5a	0.202672
Ell3	0.202655
Trim8	0.20263
B4galnt1	0.202573
Npc1l1	0.202549
Anapc5	0.202541
Npff	0.202505
Hapln1	0.202467

Ltv1	0.20246
A530016L2	0.202388
Gpr176	0.202376
Ankrd12	0.202365
Tenm2	0.202363
Cd320	0.20225
Cspg5	0.202228
Bmper	0.202195
Sec11c	0.202152
Mff	0.202142
Podxl2	0.202113
Kdelc2	0.202103
Endod1	0.202087
Stip1	0.202064
Ccdc155	0.202047
Tcf25	0.202035
Cldn7	0.202024
Znrf1	0.20202
Lrch3	0.201965
Mfsd1	0.201927
BC016423	0.201916
Slc25a13	0.201885
Slc35e2	0.201865
Phf14	0.201864
Smek1	0.201851
Agap1	0.201798
Tsg101	0.201744
Acad9	0.201741
Eya3	0.201694
Tubb6	0.201677
Celsr1	0.201664
Rev3l	0.201624
Dync1i2	0.201607
Acap2	0.201507
Ube2g2	0.201434
Prdx1	0.201433
Magoh	0.201336
Trappc9	0.201332
Ccdc77	0.201279
Nxn	0.201263
Cd83	0.201261
1110037F0	0.201247
Dnal1	0.201244
Xpnpep1	0.201237
Tpsab1	0.201237
Pacs2	0.201237
Atp2c1	0.201171

Ubp1	0.201031
Tgfbr1	0.200995
Phykpl	0.200903
Agpat6	0.20084
Nmur2	0.20084
Crygn	0.200823
Mrpl3	0.200795
Pin1	0.200708
Abhd5	0.200701
BC018242	0.200681
Wars2	0.200423
Rapgef4	0.200423
Lrpap1	0.200366
Evi5l	0.200305
Srsf6	0.200296
Prss40	0.200237
2610301B2	0.200229
Krt17	0.200186
Vwa1	0.20015
Slc12a6	0.200118
Rerg	0.200112
Ybx2	0.200064
Neto1	0.200054
Rab5a	0.200015
Marcksl1	0.199999
Cldn9	0.199976
Drg1	0.199953
Myrf	0.199928
Mtmr14	0.199926
Emd	0.199902
Zfp619	0.199876
Itga8	0.199827
Inhba	0.199814
Iqsec3	0.199797
Cdc37l1	0.199787
Kpna4	0.199743
Mterfd3	0.199741
Hivep3	0.199727
Chrna10	0.199721
Zfp112	0.19969
Btbd6	0.199644
Ptpdc1	0.199585
Krtcap3	0.199573
Med9	0.199537
Ccdc79	0.199495
Abi2	0.199448
Sema4g	0.199435

Zfp687	0.199365
Ptma	0.199365
Thg1l	0.199319
Tram1l1	0.199307
Macrod1	0.199297
Utp6	0.199291
Muc5ac	0.199258
Clec2l	0.199252
Apoa1bp	0.199131
Exoc1	0.199034
Pam16	0.199
Polrmt	0.198956
Rfc4	0.198954
Rptor	0.19895
Zfand6	0.198949
Utp20	0.198939
Upf1	0.198931
Dhx8	0.198762
Ptges	0.198733
Krt42	0.198733
Syngr3	0.198707
Cndp2	0.198671
Itga3	0.198659
Rela	0.198647
Slc44a2	0.198642
Mib1	0.198604
Pcca	0.198468
Pdk4	0.198466
Cisd2	0.198442
Strada	0.198432
Rhog	0.198411
Dnmt3b	0.198392
Serf2	0.198345
Blcap	0.198345
Clps	0.19826
Trnp1	0.198235
Ttll11	0.198214
Hspa4l	0.198203
Ccar2	0.198202
Il1rap	0.198195
Tmem55b	0.198167
Usp45	0.198161
Rasa1	0.198112
Fgf18	0.198068
Hps3	0.198003
Eif4g1	0.197985
Syn2	0.197897

Ppp1r1b	0.197764
Spdef	0.19775
Ufc1	0.197722
Crh	0.197656
Faim	0.197603
F5	0.197603
Rngtt	0.197593
Letm1	0.197579
Cyb5b	0.197565
Ctr9	0.197529
1700125H2	0.197529
Cpne2	0.197527
Nalcn	0.197527
Cobll1	0.197479
Cenph	0.197472
Brpf3	0.197471
Simc1	0.19744
Tgfbr3	0.197416
Plk5	0.197385
Nsun5	0.197349
Rbfox1	0.197259
Epc2	0.197258
Krt15	0.197223
Slc30a9	0.197211
Vrk1	0.197184
Zfp938	0.197153
Uap1	0.197129
Hmg20b	0.197124
Spag4	0.197123
Wasf3	0.197031
Cst6	0.196959
Alox5ap	0.196951
Tigd3	0.196846
Gabra1	0.196812
Raph1	0.196779
Por	0.19675
Ndufv2	0.196692
Axl	0.196684
Ttc30b	0.196677
2410089E0	0.19665
Gdf10	0.196623
Masp2	0.196585
Rpp21	0.196572
9130019O2	0.196549
Adamts13	0.196503
Trim67	0.196475
Pthlh	0.196427

Tmx1	0.196399
Ceacam15	0.196399
Ttll4	0.196398
Slc44a5	0.196367
Akap5	0.196347
Rpl37a	0.196321
Prss45	0.196277
Cradd	0.196232
Metap1d	0.196227
Ctrb1	0.196226
Rnf34	0.196206
Wdr38	0.196199
Arhgap39	0.196158
2810459M	0.196154
Ube2k	0.196142
Sbf2	0.19614
Acot11	0.196124
2510003E0	0.196036
Ahcyl1	0.196026
Naglu	0.196016
Chst2	0.195918
Pcbd2	0.195913
Sycp1	0.195837
Dnmt1	0.195816
Tmem74	0.195797
H2afx	0.195794
Zcchc11	0.195771
Echdc2	0.19577
Oser1	0.195747
Proca1	0.19572
Tmem179	0.195693
Gatsl3	0.195692
Kbtbd7	0.195673
Fam81a	0.195671
Magee2	0.19563
C1qtnf9	0.195595
Klf1	0.195582
Cdhr1	0.195543
Pik3ip1	0.195542
Msto1	0.195485
Insig2	0.195423
1700021K1	0.19538
Mrps26	0.195373
Ttc34	0.195371
Cdc42ep3	0.195342
Swap70	0.195333
Col16a1	0.195266

Ago2	0.19525
Phkg1	0.195243
Trim39	0.195235
Zfp1	0.19522
Mbtps1	0.195202
Prmt10	0.195178
Raver1	0.195166
Sstr3	0.195136
Tcp10a	0.195113
Ppp6r3	0.195016
Atxn10	0.19499
Fam219b	0.194967
Vil1	0.194965
Myh13	0.194946
Zfp574	0.194925
E2f8	0.194903
Armc9	0.19484
Yipf4	0.194838
Acer2	0.194821
Foxk2	0.19481
Mtfmt	0.194809
Prss57	0.194798
Focad	0.194733
Mto1	0.194619
Dnah9	0.194616
Elk4	0.194597
Palm	0.194588
Slc38a9	0.194571
Zfp784	0.194548
Wnt4	0.194535
Hdhd3	0.194513
Asb6	0.194507
Ptger2	0.194441
Galnt2	0.194439
Rasgef1b	0.194345
Pcp4l1	0.194253
Lrrfip2	0.19424
Itga4	0.194229
Ugcg	0.194225
Vash2	0.194204
Hint3	0.194182
Ube3a	0.194135
Map3k3	0.194113
Bbs7	0.194011
Sema3d	0.193981
Mrps24	0.19398
Metap2	0.193961

Limk2	0.19393
Tbl1xr1	0.19392
Mrpl44	0.193916
Idh3b	0.193875
Asap2	0.193874
Fam19a2	0.193841
Dcaf10	0.193838
Hamp	0.193819
Cops4	0.193756
Rad52	0.193684
B4galt4	0.19363
Alpi	0.193555
Bbs4	0.193541
Mesp1	0.193492
Grin2c	0.193475
Kcnt2	0.19347
Pnma1	0.193445
Snx6	0.193403
Mtg1	0.19339
Ankrd63	0.193324
Heyl	0.193306
Ccdc15	0.193287
Ndufb6	0.193146
Zfp58	0.193103
Ptbp3	0.193093
Hibadh	0.193075
Wbscr27	0.193034
Ly6c1	0.19302
Bnc2	0.192972
Selo	0.19296
Rhot1	0.192905
Fam174a	0.192904
Il4i1	0.192894
Slc25a16	0.192851
Dhrs11	0.192847
Fbp1	0.192839
Txndc17	0.192799
Mslnl	0.192769
Lima1	0.192768
Thumpd2	0.192747
Tmem171	0.192715
Calm1	0.19271
Ythdc2	0.192706
Gm833	0.192645
Olfr157	0.192628
Scg5	0.192492
Crtac1	0.192469

Pdp1	0.192467
1700020L2	0.19245
B230219D2	0.19242
Itch	0.192405
Capn1	0.192396
Gpn2	0.192355
Masp1	0.192347
Il10rb	0.192333
Tada1	0.192295
5730455P1	0.192283
Cebpa	0.192257
Clk1	0.192231
Tmem161a	0.192212
Rps4x	0.192194
Msantd2	0.192108
Ptpn1	0.192037
Nov	0.191963
Nkapl	0.191962
Pogk	0.191954
Rab28	0.191912
Scaf4	0.191834
Wfdc15a	0.191833
Mafg	0.191804
Ebpl	0.191801
Arnt	0.191798
Maats1	0.191766
Snx13	0.191737
Ier3ip1	0.19173
Qprt	0.191726
Fndc3b	0.191656
Syncrip	0.191572
B3gnt7	0.191566
Haao	0.191516
Rps20	0.191499
Hydin	0.191481
Vwde	0.191461
E2f5	0.191422
Ddx58	0.191421
Pctp	0.191385
Ccny	0.191365
Emilin1	0.191317
Atxn7l3b	0.191288
Slc36a2	0.191259
Trim9	0.191225
Adora2b	0.191178
Ucn	0.191151
Prc1	0.191147

Il17c	0.1911
Mpv17l2	0.191034
Cd14	0.19102
Pou4f3	0.191017
Cybrd1	0.19099
Cdk11b	0.190973
Papd5	0.190944
Naa30	0.190871
Col25a1	0.190847
Zfp36l3	0.190791
Slc9a2	0.190728
Il17rd	0.190709
Dcp2	0.190614
Ctif	0.190611
Spns2	0.190588
Tvp23a	0.190551
Cacna1a	0.19052
Lix1	0.190466
Rbbp9	0.190456
Rnf220	0.190427
Bhlhe22	0.19041
D10Bwg13	0.190405
Rpp40	0.190372
Trio	0.190327
Klhl23	0.190259
Zfp839	0.190255
Pom121	0.190245
Mib2	0.190237
Serpinf1	0.190215
Atp2a3	0.190172
Bcl6b	0.190155
Pomk	0.190106
BC051142	0.190092
Hoxc11	0.190083
Fbxw11	0.190055
Grik2	0.190054
Lig3	0.190019
Slc2a10	0.190012
Arsa	0.190008
Ammecr1l	0.190003
Wdr33	0.189959
Cab39	0.189929
Erc2	0.189896
Neu1	0.189873
Avpi1	0.189863
Sdc3	0.189811
Osbpl6	0.189692

Obscn	0.189673
Slc2a13	0.189637
Fbxw2	0.189626
Erlin1	0.189587
Akap10	0.18958
Gna15	0.189547
Ncf4	0.189531
Parp14	0.18946
Ntpcr	0.189427
Crtc1	0.189402
Pcbd1	0.189396
Gm266	0.18929
Usp49	0.189251
Gm5148	0.18925
Ube2f	0.189198
Dpf1	0.189175
Ap3s2	0.189164
Ergic1	0.189157
Nexn	0.189131
Agbl3	0.189064
Cxxc1	0.189011
Bambi	0.18894
Tmem245	0.188912
Ggct	0.188763
Glp1r	0.188733
Trappc3l	0.188691
Naa15	0.188673
Men1	0.188673
Dmwd	0.188662
Gns	0.188656
Tnfaip8l2	0.18865
Prkar1a	0.188563
Vdac2	0.188549
Eif3m	0.188467
Nfia	0.188461
Klhl15	0.188413
Epha8	0.188302
Brinp3	0.188255
Usp13	0.188234
Rc3h2	0.188215
Pea15b	0.188193
1700120K0	0.188095
Fam86	0.188092
Aplp2	0.18808
Nmd3	0.188075
Dock7	0.188071
Wdr43	0.188052

Zcchc9	0.188042
Zfp710	0.187979
Grlf1	0.187953
Gnb3	0.187951
Mfge8	0.187881
Prf1	0.187855
Nt5dc1	0.187841
Fam221a	0.18775
Nudt11	0.187722
Mtus2	0.187634
Scyl2	0.187628
Mtif2	0.18761
Elp3	0.187609
Psmd9	0.187586
Fstl3	0.187548
Rpl22l1	0.187532
Fam65b	0.187426
Gpr3	0.187311
Nxt1	0.187247
Dctn4	0.187126
Hmga2	0.187119
Cacnb4	0.187107
Hoxd11	0.187022
Hmgn2	0.18697
Ntsr1	0.186916
Vamp2	0.186845
Atad2b	0.186843
Adamts3	0.186827
Stox1	0.186783
Fnbp1l	0.186743
Zkscan4	0.186743
Fam117b	0.18674
Mkln1	0.186731
Mrgprg	0.186728
Nr0b2	0.186728
Mtmr6	0.186715
Ostm1	0.186713
Nrbf2	0.186711
Adcy9	0.18669
Plekhm1	0.186673
Foxk1	0.186661
Rcor2	0.186638
St14	0.18663
Slc41a1	0.186607
Polr2c	0.18659
B4galt1	0.186546
Prkg2	0.186523

Fbln5	0.186513
Mras	0.186436
Lin7c	0.186431
Mettl1	0.186384
Ccbe1	0.186303
Aurkc	0.186298
Npepl1	0.18628
Slc7a2	0.186268
Arpp19	0.186226
Zfp11	0.186214
Ugdh	0.186148
Phf6	0.186098
Pkn1	0.185997
Sec24d	0.18591
Kcnt1	0.185887
Rab27a	0.185886
Herpud2	0.185862
Cog3	0.185861
Gm5134	0.185828
Apoa4	0.185808
Srsf1	0.185801
Coro2b	0.185782
Scimp	0.185759
Rap1gap2	0.18575
Vstm4	0.185685
Npw	0.185638
Hoxd13	0.185583
Kcne2	0.185566
Mtmr7	0.185548
Camsap2	0.185487
Scap	0.185459
Sema6b	0.185427
Mtch2	0.185393
Dnttip2	0.185374
Ctbp1	0.18537
Tacc2	0.18535
Kcnk4	0.185342
Wdr78	0.18527
H2-Q10	0.18524
Cox18	0.185229
Rtp4	0.185212
Snx21	0.185174
Bcl2l1	0.18516
Sepp1	0.185119
Tsks	0.18504
Rusc2	0.185032
Vps13c	0.185026

Slc22a23	0.184995
Napb	0.184992
Aspm	0.184929
Csnk2a1	0.184926
Mapk14	0.184926
C1qbp	0.184892
Chic1	0.184887
Dld	0.184765
Gm13889	0.18476
Zmym1	0.184663
Prmt2	0.1846
Car3	0.184574
Tnxb	0.184523
Tmem144	0.184407
Tmed3	0.184348
Rap1gds1	0.184318
Gpn1	0.184285
Stx2	0.184274
Wdr41	0.184217
Il12b	0.184084
Tmem130	0.184026
Xylb	0.184023
Ogfrl1	0.18396
Cacna2d4	0.183957
Hmgxb4	0.183907
Lrrn1	0.183904
4931440F1	0.18388
Gng13	0.183878
Slc4a1	0.183838
Oaz2	0.183828
Stx1b	0.183793
Shprh	0.183697
Tshr	0.183666
Tgfb1i1	0.183654
Cops8	0.183653
S100a14	0.183638
Sik2	0.183599
Nccrp1	0.183568
Bid	0.18356
Uba2	0.183552
Dera	0.183539
Tspan11	0.183494
Ddx39	0.183484
Psmd4	0.183455
Lmbr1l	0.183349
Leprel1	0.18333
Ambra1	0.183306

Psmf1	0.183263
Psmd14	0.183258
Lynx1	0.183238
Ipp	0.183222
Dpp4	0.183222
AB124611	0.183215
Rnf149	0.183109
Fbxo34	0.183103
Mrps18a	0.1831
Catsperg1	0.183096
Dusp28	0.183082
Acox3	0.183066
Ap3d1	0.183047
Eif3a	0.183018
Arfgef2	0.183008
Lrp3	0.183001
Arhgef16	0.182986
Spin4	0.182984
Sipa1	0.182964
Ttyh3	0.182961
Zfp873	0.182915
Dcaf12	0.182903
Kansl1l	0.182884
Farsb	0.182857
Zdhhc22	0.182845
Soat2	0.182843
Slc13a3	0.182814
Tcf4	0.182796
Shisa4	0.18278
Cadps	0.182756
Dennd5a	0.182756
Acat3	0.182742
Neil2	0.182713
D130040H1	0.182692
Fgf1	0.18265
Usp38	0.182647
Dnajc25	0.182647
Ptprr	0.182628
Ssh1	0.18262
Rbm42	0.182615
Creb3l4	0.182539
Psmg3	0.182506
Stx11	0.182493
Zap70	0.182485
Cnih4	0.182477
Lonrf1	0.182462
Narg2	0.182387

Ephx4	0.182342
Htr2a	0.182322
Plekhh2	0.182308
Rheb	0.182284
Ripply2	0.182267
Wnt2b	0.182236
Twist2	0.182233
Ppp2r5e	0.182179
Nars	0.182178
Osbpl8	0.182174
Stk38l	0.182169
Zfp446	0.18215
Ldoc1l	0.182127
Kifc5b	0.182055
Adamts17	0.182023
Tspyl3	0.181995
Cd40	0.181985
AI987944	0.181958
Fam32a	0.181933
Aplnr	0.181919
Sorcs1	0.181899
Pdcd1	0.181893
Morc1	0.181806
Sephs2	0.181796
B3gat3	0.181775
Pstpip1	0.181768
Slc39a3	0.181737
Arl14ep	0.181724
Pdzd4	0.181721
Nos3	0.181716
Lrp1b	0.181696
Tmem66	0.181621
Slc39a4	0.181586
Kcnj5	0.181578
Pdcl3	0.181511
Nav2	0.18146
Msc	0.181449
Paip2b	0.181449
Prdm15	0.181373
Rhpn2	0.18129
Dtwd2	0.181256
Srsf4	0.181173
Ddx26b	0.181111
Mmgt1	0.181104
Slc34a1	0.181103
Prr15l	0.18109
Sigmar1	0.181041

Chd6	0.181038
Tex261	0.181002
Msi1	0.180989
Prss21	0.180966
Ccdc28a	0.180958
Ppp1cb	0.180945
Mrpl35	0.18093
Kif3a	0.180919
Chmp1b	0.180887
Matk	0.180837
Spryd7	0.180778
Kctd7	0.180758
Stmnd1	0.180732
Asic5	0.180709
Rcvrn	0.180659
Fstl4	0.180643
Hrk	0.180638
Scai	0.180637
Spryd4	0.180576
Kcnmb1	0.180541
Pm20d1	0.180488
Fn3k	0.18048
Car12	0.180466
Zfp239	0.180422
Prokr1	0.180385
Tbrg3	0.18038
Smpd1	0.180352
Igdcc4	0.180307
Pomgnt2	0.180293
Pias2	0.180263
Lgals7	0.180237
Cct2	0.180207
Zbtb39	0.180038
Tchp	0.180036
Chn2	0.180027
Mcm3	0.179995
Cib2	0.179988
Dbr1	0.179971
Stk32b	0.179937
Ddx52	0.179848
Rufy1	0.17976
Fam168a	0.179734
Plaur	0.179732
Acn9	0.179713
Nkap	0.179648
Arhgap22	0.179634
Dst	0.179609

Abcc8	0.179609
Olfr1033	0.179604
Chsy3	0.179584
Hk3	0.179535
Serpine2	0.17953
2310022B0	0.179487
Apc	0.179414
Adamts7	0.179364
Cnn1	0.179327
Lepr	0.179321
Kcnab1	0.179316
Zfp275	0.179314
Uhrf1bp1	0.179307
Kcnmb4	0.179272
Hsd17b14	0.179228
Cacng5	0.179227
Rgl1	0.179163
Shroom1	0.179123
Ankmy1	0.17912
Mthfd2	0.179107
Ppp4r2	0.179014
Pbk	0.178982
Usp7	0.178978
Gpr20	0.178928
Zfp518b	0.178891
Riiad1	0.178881
Kcnk1	0.178863
Tinagl1	0.178848
Hcrtr1	0.178803
Mical3	0.178802
Mcm6	0.178781
AW551984	0.178749
Arf3	0.178746
Dhx33	0.178732
U2af1	0.178677
Cc2d1b	0.178654
Tph1	0.178578
Eif3j1	0.178523
Unkl	0.178436
Fuom	0.178408
Ephx3	0.17838
Prosc	0.17832
Krt9	0.17832
C2cd4b	0.178299
Tmc7	0.178275
Ppp2cb	0.178216
Taf12	0.178171

Ap2m1	0.178146
Cyp2a4	0.178142
Sgol2	0.178084
Ifit2	0.178002
Epx	0.177986
Ubap1	0.177982
Nbl1	0.177968
Zfp831	0.177892
Pkn3	0.177858
Sohlh2	0.177836
Cplx1	0.177768
Pitpnb	0.177718
Tax1bp1	0.177648
Creg2	0.177639
Tmem41a	0.177635
Ccdc110	0.177589
Foxf2	0.177578
1700016K1	0.177568
Snrnp27	0.177536
Fancl	0.177528
Rnpc3	0.177517
Rbp2	0.17751
Zfp952	0.17748
Gpr45	0.177432
Cln5	0.177408
Otud7a	0.177197
Nifk	0.177175
Car7	0.177136
Gnaq	0.177129
Neil3	0.177102
Disp2	0.17704
Mtpn	0.177001
Dguok	0.176996
Ppp2r2a	0.176966
Sybu	0.176961
Ptprm	0.176878
Psme4	0.176808
Smoc1	0.17679
Rab32	0.176655
Cep192	0.176643
Usf2	0.176621
Zrsr1	0.176614
Fhit	0.176595
Akr1a1	0.176475
Lrrc8c	0.176454
1700021F0	0.176443
Cbx7	0.176424

Gpr135	0.176405
Nudt7	0.176381
Gsg2	0.176349
Psmc6	0.176342
Dynlrb2	0.176217
Htatip2	0.176171
Trim24	0.176085
Pafah1b1	0.175966
Cxxc5	0.175951
Lcn2	0.175948
Dscam	0.175905
Psmb2	0.175893
Zbbx	0.175875
Mogs	0.175875
Ggact	0.175859
Slc8a3	0.175858
Stard5	0.175759
Prkaa1	0.175716
Seh1l	0.17568
Usp31	0.175666
Spc25	0.175645
Samhd1	0.175607
Eif3g	0.175603
Cx3cr1	0.175546
Dnajc9	0.175511
Stim2	0.175484
Cgn	0.175399
Meox1	0.175388
Sypl2	0.175377
Hp1bp3	0.17534
Apobec2	0.175251
Drd3	0.175147
Rab3d	0.175142
Cyb561	0.175092
Hcn4	0.175083
Osgin1	0.175052
Papolg	0.175051
Ddx3x	0.175033
Sox30	0.174948
Btbd7	0.174851
Azi1	0.174831
Rest	0.174822
Cox7c	0.174798
Rfc5	0.17479
Mrpl4	0.174775
Dgkg	0.174671
Grsf1	0.174649

Tagln	0.174645
Ptpra	0.174612
Rogdi	0.174593
Supt16	0.174563
Ipo5	0.174497
Fbxl14	0.174457
Stard13	0.174447
Brca2	0.174438
Zcchc10	0.174389
Decr1	0.174327
Aqp7	0.174324
4921504E0	0.174239
Bckdk	0.174201
Cntn2	0.174183
Car2	0.174069
Tcn2	0.174027
Mpp5	0.174023
Thumpd1	0.174002
Enpp6	0.173994
Klhl40	0.173936
Zbtb8b	0.173899
Serpina1a	0.173864
Cpsf1	0.17386
Cnfn	0.173847
Polg2	0.173842
Six4	0.173836
Zfp423	0.173822
Chmp3	0.173805
Ttl	0.173799
Eva1b	0.173778
Gxylt2	0.173719
Pgm2l1	0.173714
Oxt	0.173648
Smoc2	0.173596
Map1s	0.173535
Hbegf	0.173502
Rtn1	0.173469
Hmgb2	0.173461
Nrbp2	0.173456
Tmem184a	0.173416
Ehd3	0.173414
Myadml2	0.173407
Kcnma1	0.173379
Csmd1	0.173369
Impdh2	0.173323
Elane	0.173299
Gpam	0.173295

Smarcad1	0.173267
Tdg	0.173215
Aire	0.17313
Mapkapk2	0.173125
Abcb10	0.173121
Sra1	0.17312
Dpm3	0.173083
Nr2c2	0.173078
Inadl	0.172921
Dusp13	0.172916
Dnajb1	0.172913
Grp	0.172901
P4ha2	0.172852
Tfrc	0.172846
Nog	0.172813
Spata31d1a	0.17276
Tbc1d19	0.172758
Zfp316	0.172756
Mgst1	0.172748
Rpl5	0.172745
Efemp2	0.17273
Trim33	0.172684
Arf6	0.172564
Hebp2	0.172563
Gm6583	0.172562
Sdr9c7	0.172526
Grik4	0.172511
Ube4b	0.172405
Trnt1	0.172391
Rfc1	0.172378
Clec3b	0.172374
Mfsd7a	0.172334
Ferd3l	0.172332
Plgrkt	0.172319
Wnt5b	0.172318
Sh2b2	0.172298
Prrx2	0.172284
Ppig	0.172237
Trim56	0.172221
Card9	0.172197
Gpbar1	0.172181
7-Sep	0.172153
Cyyr1	0.172136
Tbrg1	0.17207
Btrc	0.172054
Topors	0.172025
Ppp1r21	0.171914

Sema7a	0.171878
Iffo2	0.171772
Atg4d	0.171758
R3hcc1l	0.171734
Pcnx	0.171705
Lcat	0.171618
Phlda3	0.171606
Vstm2b	0.17158
Golm1	0.171572
Rras2	0.171568
Trim13	0.171502
Nos1	0.17149
Ccdc43	0.171485
Elovl3	0.171469
Foxn4	0.171461
Ubqln1	0.171457
Med28	0.171409
Paxbp1	0.171395
Ccl1	0.171384
Itpripl2	0.171356
Inpp5d	0.17126
1190005I0(	0.171228
Gm128	0.171193
Slc24a4	0.171128
Mustn1	0.171126
Lyplal1	0.171088
Myo18a	0.171023
Sltm	0.171023
2410127L1	0.170981
Cdkal1	0.170961
Gm10639	0.170872
Eno4	0.170847
Dpep1	0.170813
Nipa1	0.170805
Ttc27	0.170798
Zdhhc12	0.170773
Notch4	0.170753
BC021614	0.17075
Oat	0.170724
Hgfac	0.170702
Drg2	0.17061
Pyroxd2	0.170554
Spc24	0.170538
Grm1	0.170537
Dennd3	0.170481
H2-Q2	0.170425
Prdm16	0.170407

Gtf2ird1	0.170339
Deb1	0.17024
Col8a1	0.170225
Phgdh	0.170224
Qpct	0.170193
Utp14a	0.170149
Mlxip	0.170094
Tpsg1	0.170051
Golga5	0.17005
Cdk6	0.170046
Pawr	0.170042
Thoc5	0.170016
Pdia6	0.169978
Gpx4	0.169968
Magel2	0.169958
Rlbp1	0.169905
Rufy2	0.169886
Mrfap1	0.169876
Zfp819	0.16987
Fam25c	0.169839
Gnmt	0.169811
H2-Bl	0.169798
Mipep	0.169785
Taf1	0.169771
Klhl38	0.169726
Slc17a6	0.16971
Etv4	0.169704
Gse1	0.169659
Fam109a	0.169654
Ntn4	0.169625
Pxdc1	0.169606
Rad18	0.169593
Hcfc2	0.169586
Angptl2	0.169503
Mtrf1l	0.169494
Tnfrsf25	0.169485
Sema3f	0.169472
Srp14	0.169418
Plekha5	0.169403
Gpr56	0.169386
Mgll	0.169363
Tmf1	0.169278
Ptplad2	0.169255
Olfr322	0.169196
Tbc1d15	0.169147
Ints1	0.169135
Zfp93	0.169119

Hdgfrp3	0.169108
Prrg1	0.168996
2610524H(	0.168979
Art4	0.168973
Gabarapl1	0.168969
Polb	0.16892
Polr3b	0.168891
Snrpa1	0.168886
Spidr	0.168858
Casp7	0.168709
Phlpp1	0.168676
Map6d1	0.168645
Cpne3	0.168577
Btaf1	0.168557
Ccdc23	0.16855
Fam196a	0.16852
Cnr1	0.168455
Shcbp1l	0.168454
Elac1	0.168436
Dyrk2	0.16841
Gpr150	0.168382
Gabra2	0.168315
Tbc1d23	0.168307
Mgat1	0.168303
Btbd1	0.168291
Tbc1d10a	0.168271
Ikzf4	0.168267
Catsper4	0.168249
Mtfr2	0.168233
Ror1	0.168217
Acsf2	0.168215
Smad1	0.168205
Slc5a11	0.168196
Wdr75	0.168148
Fam53a	0.168116
Fbl	0.168099
Urah	0.168076
Ppp1r16b	0.168074
Arhgef40	0.16787
Npy2r	0.16785
Fut9	0.167841
Ssu72	0.167784
Prune2	0.167721
Rpn1	0.167706
Stag2	0.167669
Cmtm4	0.167638
Ifi35	0.16763

Akp3	0.167603
Rab1b	0.167561
Slc25a38	0.167554
Jph2	0.167548
Anks6	0.16753
Dnajc18	0.16748
Klk15	0.167477
Darc	0.167455
Mmp28	0.167454
Rbbp5	0.167425
Gp5	0.167423
Ntn1	0.167393
Specc1l	0.167342
Srsf5	0.167326
Tifab	0.167282
Acot6	0.167261
2900026A(	0.167242
Pkig	0.167169
1700084C(	0.167142
C530008M	0.167062
Dock8	0.167036
Xkr6	0.167029
A530064D(	0.167024
P2ry2	0.167018
Ginm1	0.167011
Vgll4	0.16697
Glra3	0.166964
Myo6	0.166953
Zfp457	0.166916
Lrrc47	0.166912
Stk3	0.16689
Noxa1	0.166888
Gusb	0.166877
Prkd3	0.166845
Pla2g12a	0.166829
BC023829	0.166793
Ino80d	0.166755
D16Ertd47:	0.166738
Ndufa4	0.166715
Nkx2-1	0.166708
Coch	0.166681
Slmap	0.166668
Cnot2	0.166636
Pdlim4	0.166573
Farp2	0.166572
Lpcat1	0.166566
Pigt	0.166566

Gpt2	0.166523
Ankle2	0.1665
Cebpd	0.166487
Prkag2	0.166464
Epha5	0.166388
Thada	0.166356
Pkd2	0.166345
Stom	0.166264
Tgif2	0.166214
Kif11	0.166213
Gfod1	0.166202
Capn9	0.166181
Tmem8	0.166174
Srp72	0.166157
Alkbh3	0.166092
Npy1r	0.166065
Ssr1	0.166049
Papln	0.165972
Adamts2	0.165934
Mcm10	0.165874
Synpo	0.16587
Impg1	0.165856
Rock1	0.16585
Pwwp2b	0.165764
Fkbp8	0.165741
Entpd7	0.165661
Xpa	0.165625
Atp13a3	0.165618
2010109I0	0.165601
Ghrh	0.165382
Mreg	0.165365
Pigm	0.165306
Faf1	0.165298
Rb1cc1	0.165181
Emc7	0.165156
Vsig2	0.16514
Sh3gl2	0.165127
Mapk4	0.165123
Map3k7	0.16507
Lrrc40	0.165066
Tmem18	0.165046
4931409K2	0.165027
Tex19.2	0.165006
Mmd2	0.164956
N6amt1	0.164916
Flt1	0.164805
Celf6	0.164802

Ptn	0.16478
Dcun1d5	0.164773
Zfp454	0.16477
Suv39h2	0.164693
Klhl9	0.164681
Npepps	0.164681
Ebf4	0.164668
Rab2a	0.164654
Stard3nl	0.164651
Fam20c	0.164638
Ubac2	0.164601
Piwil1	0.1646
Zbtb6	0.164591
Timp2	0.164552
Sntg2	0.164536
Pon2	0.164534
Npb	0.164525
Klhl22	0.164518
Fam189a2	0.164482
Fam92a	0.164456
Lsm11	0.164404
Cox19	0.164386
Nav1	0.164379
Rnf187	0.164375
Fra10ac1	0.164372
Msrb3	0.164371
Zfp763	0.164357
Pdxk	0.164323
Ogdh	0.164305
Cdc5l	0.164271
Csnk1g3	0.164233
Nck2	0.164169
Gigyf1	0.164069
Spatc1	0.163974
Hecw1	0.163948
Dna2	0.163868
Foxo4	0.163802
Lcorl	0.163766
Rnf2	0.163765
Lypd6	0.163764
Snapc4	0.163746
Map9	0.163733
Ppp1r13b	0.163727
Zfand5	0.163593
Zfp800	0.163577
Thoc3	0.163575
1700019D0	0.163558

Slc30a5	0.163431
Trrap	0.163426
P2ry1	0.163404
Fam101a	0.163305
Wscd2	0.163296
Pgam1	0.163196
Psd2	0.163174
Adap2	0.163149
Wash	0.163113
H3f3a	0.163095
Memo1	0.163062
Mtx2	0.163019
Fermt2	0.162999
Muc20	0.162939
Gmds	0.162926
Kank3	0.162921
Usp25	0.162888
Bean1	0.16288
Zfp788	0.162846
Trim29	0.162826
Snx7	0.162783
Nfix	0.162763
Rab11fip5	0.162727
Mfap4	0.162705
Pou4f1	0.1627
Clic6	0.162666
Klk12	0.162664
Aard	0.162657
Arhgef7	0.162579
Zfp940	0.162551
Txlna	0.162529
St6galnac5	0.162488
Rabep1	0.162453
Cyp2w1	0.162439
Jade2	0.162436
Fmn1	0.1624
Vaultrc5	0.162314
Pex6	0.162314
Prss16	0.162284
Tnfrsf11b	0.162258
Epn3	0.162255
Inpp5k	0.162247
Rabl5	0.162234
Tal2	0.162227
Tff2	0.162201
Acap1	0.162191
Zcchc24	0.162129

Ythdf2	0.162125
Iqcj	0.161999
Fam69a	0.161974
Defa3	0.161896
Mef2a	0.161883
Zfp810	0.161866
Gip	0.161854
Slc2a6	0.161841
Klhdc4	0.161821
Ccdc153	0.1618
Pole4	0.161798
Slc15a3	0.161781
Cacna2d1	0.161777
Krtap17-1	0.161772
Chst4	0.16174
Rsbn1	0.161631
Slc35e3	0.161615
Map3k2	0.161572
Sf1	0.16153
Car15	0.16148
Rin3	0.161476
Pik3cb	0.161475
Gnas	0.161467
Snx11	0.161456
Slc22a15	0.161414
Sec24a	0.161405
Nudt15	0.161403
Esd	0.161342
Trappc10	0.161341
Tab1	0.161242
Slc1a1	0.161217
Maz	0.161203
Kcng1	0.161122
Tug1	0.161119
Tmod2	0.161091
Dgat1	0.161087
Fkbp5	0.161082
Unc119	0.161044
Ppp1r35	0.161003
Uqcc1	0.160995
Vps4b	0.160986
Clcf1	0.160984
Kcnd1	0.160878
Hat1	0.160878
Fam78b	0.160868
Ninj1	0.160772
Wdr70	0.160761

Spata4	0.16074
Rnf11	0.160555
Zcchc4	0.160551
Ppp2ca	0.160525
Entpd3	0.160494
Phf10	0.160446
Uso1	0.160419
Ticam1	0.160349
Napepld	0.160348
Dtx4	0.160339
Pak1ip1	0.160323
2610018G(	0.160278
3110082I1	0.160217
Setd1a	0.160197
Syt2	0.160175
Nap1l5	0.160171
Ccdc91	0.160132
Tmem150c	0.160127
Rasl10b	0.160119
Vhl	0.160109
Ankrd33	0.16006
Wdfy2	0.159993
Kcnd2	0.159937
Lsm10	0.159935
Tie1	0.159934
Rab10os	0.159921
9630033F2	0.15989
Dok6	0.159854
Rab13	0.15984
Rprml	0.159838
Fbxl20	0.159828
Ybx3	0.159808
Cxcl3	0.159789
Gng2	0.159787
Gpr1	0.159701
Lrrc38	0.15969
Zbtb40	0.159679
Bmyc	0.159649
Rnpep	0.159639
Ccdc71l	0.159591
Angpt4	0.159564
Sema3e	0.159555
Ankrd34b	0.159542
E2f6	0.159507
Cyfip1	0.159488
Gdap1	0.159402
Ecscr	0.159367

Sncaip	0.159264
Zfp324	0.159242
Rnase1	0.159163
Tcea3	0.159133
Cacna1h	0.159076
Cabp7	0.159061
Gprin2	0.159049
Dip2b	0.158981
Opn4	0.158939
Cog6	0.158927
Cds1	0.158841
Ppapdc1a	0.158821
Tbc1d13	0.158797
Eml4	0.158751
Atp13a1	0.158695
Taldo1	0.158671
Amt	0.15859
Eef1e1	0.158572
Cdc42bpa	0.158566
Zfp956	0.158563
Cgnl1	0.158475
Rnf141	0.158469
Ppie	0.158415
Ext2	0.158391
Sec63	0.15836
St6gal2	0.158344
Calcrl	0.158328
Il15ra	0.158304
Cep72	0.15827
Orm2	0.15826
Ifna13	0.158245
Nucks1	0.158228
Mmp24	0.158188
Adamts14	0.158128
Ptprf	0.158114
Arid1b	0.158082
Kcna4	0.158076
Cox4i2	0.157966
Slc44a3	0.157947
Dusp15	0.157864
Snx24	0.157743
Prmt3	0.157741
Dip2a	0.157723
3110047P2	0.157688
Tesk1	0.15761
Cxcl5	0.157594
Epsti1	0.157579

Sik3	0.157447
Zfp647	0.157445
Brd1	0.157436
Chst5	0.157406
Col11a1	0.157403
Trim31	0.15735
Rpl6	0.157255
Stac	0.157249
Scarf2	0.157186
Ifitm6	0.157178
Atf7ip2	0.157174
Zfp598	0.157158
Unc5d	0.157143
Dand5	0.157074
Loxl4	0.157024
Trnau1ap	0.157018
Spry1	0.157007
Lmod1	0.156958
Vps39	0.156957
Wnt16	0.15685
Prkrip1	0.156782
Olfr90	0.156698
Zfp101	0.15668
Kpna3	0.156579
Hck	0.156571
Nkx1-2	0.156555
Slc44a4	0.156515
Suz12	0.156515
Fmnl2	0.156491
Taf6l	0.156472
Coprs	0.156447
Zfp280c	0.156331
Bend3	0.156331
Amfr	0.156304
Accs	0.156272
Zfp109	0.156217
Wdr11	0.156188
Cadm2	0.156112
Gpr101	0.156019
Med23	0.156007
Msh2	0.155976
Fgf2	0.15594
Lamc3	0.155908
Furin	0.155902
Sebox	0.155849
Dpf2	0.155841
Slc7a8	0.155838

Fam132a	0.15582
Pfas	0.155736
Adam22	0.155735
Hdgf	0.155705
Megf10	0.155649
Cxcl2	0.155647
Gga2	0.155606
Wdr44	0.155595
Frrs1l	0.155585
Manea	0.155583
Dpp8	0.155582
Ttbk2	0.155581
Pak2	0.15553
Muc5b	0.155446
Trpc1	0.155443
Arap2	0.15543
Cgref1	0.155422
Ezh1	0.155395
Tardbp	0.155385
Apcdd1	0.155335
Fam69c	0.155321
Zfp653	0.155292
Efnb2	0.155283
Clspn	0.155209
Ei24	0.155162
Ankrd46	0.155156
Eif5a2	0.155149
Alas1	0.155143
Klf16	0.155029
Repin1	0.154983
Ankrd61	0.154969
Sar1b	0.154921
Runx3	0.154897
Crip3	0.154895
Krt79	0.15485
Fam3a	0.154842
Coro1a	0.154813
Aoc3	0.154751
Synpo2	0.154734
Clock	0.154701
Drd5	0.154686
Nol11	0.154593
Dyrk1b	0.154518
Nbn	0.154484
Fbln2	0.154417
Fam131b	0.154327
Plxnb3	0.15432

Foxo1	0.154294
Cd22	0.154204
Ttbk1	0.154182
Pstk	0.154145
Galnt3	0.154141
Artn	0.154114
Mfsd6l	0.154111
Fam167a	0.154091
Ccnj	0.154059
D930015E(	0.154023
Zfp236	0.154009
Gdi1	0.153855
Ikzf2	0.153833
Ttc38	0.15383
Paqr5	0.153759
Hhat	0.153747
Rhoq	0.153643
F830045P1	0.153603
Npm2	0.153597
Fam129a	0.153577
Kcna6	0.153565
Usp18	0.153516
Vta1	0.153483
Cacnb2	0.153422
Rhoj	0.153405
Eif2b3	0.153382
Fhdc1	0.153356
Zbtb5	0.153125
Kif17	0.153122
Dhtkd1	0.153115
Tmem132c	0.153109
Phf13	0.153037
Lrrc73	0.152971
Col19a1	0.152969
Pabpc4	0.152819
Pddc1	0.152755
Herc4	0.152751
Pgap3	0.152715
Lrrc2	0.152693
Bspry	0.152673
Prkch	0.152669
Lmx1b	0.152552
Efemp1	0.152549
Hnrnpa0	0.152441
Vcam1	0.152414
Dock1	0.152356
Csf3	0.152332

Tdrd7	0.152289
Dmkn	0.15226
Mical2	0.152239
Slc6a12	0.152201
Otos	0.152156
Kansl3	0.15208
Bmp6	0.152054
Serpinb9	0.152054
Atxn7	0.152016
Parva	0.151931
Klf3	0.151892
Flt3l	0.15189
Cldn1	0.151876
Foxe1	0.151805
Agap2	0.151787
Tc2n	0.151748
Chst10	0.151744
Gemin6	0.151694
Ttc22	0.151682
Ube2o	0.151659
Hexa	0.151596
Arhgap42	0.151585
Dmtn	0.151552
Gstt1	0.15155
Ngef	0.151523
Usp14	0.15149
Appl1	0.151473
Kcnj14	0.151451
Mlycd	0.15145
Aox2	0.15144
Lpar1	0.151395
Lancl1	0.15135
Fam73a	0.151329
Aifm3	0.151318
Ppp4r1	0.151292
Ccdc85a	0.151259
Zfp318	0.151253
Abca8b	0.151228
Rnf112	0.151218
Hhex	0.151207
Nfe2l3	0.151159
Eif1	0.151144
Fxyd2	0.151135
Fxyd3	0.151115
Sft2d2	0.151053
Ddah1	0.151012
Hpdl	0.150983

Pnmal1	0.150936
F8a	0.15092
Fam122a	0.150871
Ppap2a	0.150853
Ifnl2	0.150821
Mta1	0.150809
Bax	0.150798
Gbx1	0.150792
Jag1	0.150791
Pard3b	0.150771
C1qtnf3	0.150751
Cpm	0.150737
Vwa5a	0.150702
Napa	0.150684
Exoc3	0.150629
Ntsr2	0.150607
Sestd1	0.150603
Uggt2	0.150583
Prss36	0.150517
Prdm9	0.150496
BC048403	0.15047
Snai1	0.150437
Esco1	0.150398
Med18	0.150371
Myo5c	0.150365
Ppp3cc	0.150347
Ly75	0.150276
Hltf	0.150217
Bri3bp	0.150195
Nfx1	0.150176
Ccser1	0.150066
Rtf1	0.150065
Macrod2	0.150032
Chsy1	0.149963
Heatr3	0.149946
Gpbp1	0.149896
Trem1	0.149887
Fam69b	0.149869
Stat1	0.149853
Ptx3	0.149786
C130050O1	0.149694
Cul1	0.149687
Vsnl1	0.149662
Msantd3	0.149655
4833403I1!	0.149545
Glrb	0.149509
Gm101	0.149487

Theg	0.149477
Hyi	0.149406
Tlr9	0.149399
Mapre3	0.149347
Tatdn2	0.149339
Ssbp4	0.149267
Emp3	0.149227
Kcns3	0.149201
Pura	0.149182
Oxtr	0.149162
Zfp707	0.14912
St6galnac6	0.149074
Nos1ap	0.149032
Folr4	0.149007
Gpatch3	0.148962
Best2	0.148942
Man1c1	0.14893
Map3k8	0.148827
Ppl	0.148778
Anxa2	0.148749
Nampt	0.148739
Espn	0.148716
Rad23a	0.148686
Atp1b3	0.148657
Ncoa1	0.148607
Etv2	0.148573
Ppp2r5c	0.14854
1600002H(	0.148539
Ythdf3	0.148539
Src	0.148526
Fbxo21	0.148446
Lamc1	0.1484
Rgs8	0.148397
Tnnt1	0.148291
Plin4	0.14827
Aldh1l2	0.148146
Sesn2	0.148128
Hcn2	0.14809
Ube2d2a	0.148063
Ear10	0.148021
Mtrf1	0.147983
Slc41a2	0.147975
Lrrc30	0.147944
Xrcc2	0.147884
Wdr45	0.14787
E130311K1	0.147859
2410004B1	0.147831

Qsox1	0.147815
Suclg2	0.147769
Ezh2	0.14774
Ankrd52	0.147735
Polm	0.147667
Ncoa5	0.147613
Lamb3	0.147592
Erf	0.14758
Fnta	0.147552
Rpusd2	0.147503
3830406C1	0.147493
Nipsnap3b	0.147418
Sdk1	0.147371
Avl9	0.14737
Pgrmc1	0.147365
Srpk1	0.147303
Nxph2	0.147252
Fhl2	0.147247
Atp6ap2	0.147216
Adnp	0.147158
Ykt6	0.147147
Bcmo1	0.147122
Kcnh5	0.146927
Igsf9	0.14687
Atg14	0.146818
Txnl4a	0.146742
Thbs4	0.146647
Dpyd	0.146647
Zfp874b	0.146563
Caskin1	0.146504
Ndufs5	0.146488
Slc35a1	0.146477
Vegfc	0.146477
Clhc1	0.146355
Kdm6a	0.146327
D630023F1	0.146283
Mkl2	0.146253
Txnl1	0.146241
Srms	0.146207
Pkdcc	0.146203
Larp1b	0.146153
Rnf139	0.146144
Gas1	0.146131
Gpr132	0.146075
Stard10	0.146074
Ccdc93	0.145978
Pgpep1	0.145969

Ngrn	0.145848
Mrpl48	0.145821
Ttll9	0.14579
Gpnmb	0.145775
Ttll13	0.145758
C330021F2	0.145735
Mdp1	0.145719
Itsn2	0.145691
Dnaja2	0.14566
Guk1	0.145646
Mycl	0.145646
Abcc10	0.145561
Lrrc16b	0.145514
Edar	0.145504
Efr3a	0.145501
Inpp5a	0.145484
Rab44	0.14545
Gimap5	0.145391
Dip2c	0.145363
Ankrd54	0.145353
Colec11	0.145218
Phf20	0.145202
Mob3c	0.145157
Btnl10	0.145157
Bsn	0.145056
Ago1	0.145053
Fhl3	0.145045
Camk4	0.145045
Ranbp17	0.145028
Galnt13	0.14502
Gyg	0.145004
Jak2	0.14498
Nipal4	0.144954
Prrg4	0.144923
Itpka	0.144879
Grik3	0.144878
Setd4	0.144877
Dennd1b	0.144776
Espnl	0.144764
Syt14	0.144726
Taf5	0.144577
Epo	0.144568
Ntan1	0.144549
Folr1	0.144538
Klhl31	0.144512
Mettl2	0.1445
Bmp2	0.14447

Atl2	0.144428
Eif2ak1	0.144426
Baiap3	0.144423
Vsig10	0.144348
Fnip1	0.144293
Birc6	0.144284
Gucy2d	0.144175
Lsm14b	0.14417
Ccdc33	0.144165
Igf1r	0.144073
Cd9	0.144029
Plvap	0.144019
Gfap	0.14399
Card11	0.143948
Ccdc61	0.143938
Yes1	0.143935
Gpr25	0.143894
Ccr4	0.143893
Wasl	0.143882
Wsb1	0.143881
Fah	0.14385
Pptc7	0.143754
Cd7	0.143731
G0s2	0.143668
Wrb	0.143662
Bmp10	0.143657
Dynlrb1	0.143614
Olfr279	0.143574
Wnk2	0.143568
BC021891	0.143567
Osbpl10	0.143523
Mixl1	0.143499
Mroh5	0.143497
Crocc	0.143428
Ppfia4	0.143413
Usp10	0.143364
Sh3gl3	0.143309
Tmem14c	0.143289
Gm10318	0.143266
Matn2	0.143218
Gnai1	0.143184
Mrps17	0.14312
Mov10	0.143102
Ska1	0.143083
Clrn1	0.143056
Etv6	0.14297
Zkscan6	0.142911

Eif4g2	0.142881
Cwf19l1	0.142863
Pnpla2	0.142849
Ocstamp	0.142799
Dram1	0.142768
Myo1d	0.142756
Lsm5	0.142755
Osgin2	0.142705
Lrp2	0.142678
Krba1	0.142624
Rarres2	0.142564
S1pr5	0.142558
Idh3a	0.142535
Cmtm3	0.142532
Rpusd3	0.142505
Hlx	0.142496
Zfp180	0.142469
Sfpq	0.142439
Mrps33	0.142418
Emc9	0.142308
Luc7l3	0.142257
Ccl17	0.142242
Mturn	0.14212
Cysltr2	0.142111
Tm2d1	0.142102
Pithd1	0.142094
Ep400	0.142062
Mctp2	0.142059
Efcab2	0.142041
Psip1	0.141987
Rilpl1	0.141954
Grhpr	0.141942
Ctdp1	0.141838
4930522H1	0.141816
Tmem184b	0.141766
Olfm1	0.141679
Capn12	0.141675
Mapkapk3	0.141626
Phkg2	0.141603
Ostc	0.141552
Vsig8	0.141546
Pds5a	0.141537
Kdm5c	0.141528
1110017D1	0.141513
Rangrf	0.141513
Ccdc129	0.141484
Fam171b	0.141479

Sp2	0.141475
Sdc2	0.14145
Olfr1342	0.14145
Chd3os	0.141423
Med13	0.141399
Gpr21	0.141379
Fbxo42	0.141353
Ptprj	0.141348
Adc	0.141335
Rab23	0.14129
Slc35c1	0.141287
Kcng3	0.141277
Jam3	0.141234
Sap130	0.141179
Irf7	0.141177
F2rl3	0.141157
Cdk5r1	0.141151
Entpd4	0.141132
Eogt	0.141131
Igf2bp3	0.141066
Adipor1	0.141048
Mal	0.141033
Cpped1	0.141019
Gltpd2	0.14098
Srfbp1	0.140933
Neurl1b	0.140867
Ccna1	0.140768
Foxo6	0.140707
Asprv1	0.140656
Golga3	0.140652
Klf2	0.140649
Krtdap	0.140644
Rad54l2	0.140565
Scamp2	0.140559
Ssh3	0.14053
2310057M	0.140516
Hace1	0.140512
Usp47	0.140502
Mettl5	0.140491
Sox7	0.140463
Mllt1	0.140443
Panx2	0.140388
Rnf182	0.140368
Syt8	0.140355
Zak	0.140107
Ifngr2	0.140106
Fahd2a	0.140073

Uck1	0.140041
Rab14	0.139976
Olfr544	0.139959
Lrrc32	0.139949
Cdc37	0.139921
Spn	0.139905
St3gal3	0.139881
Rrbp1	0.139878
Ivd	0.139874
Cux1	0.139848
Kcnk7	0.139842
Prelid2	0.139821
Ube2v2	0.139753
Nanos1	0.13973
Mcoln1	0.139622
Kif7	0.139532
Insc	0.139512
Atp6v1h	0.139389
Katnbl1	0.139381
Sft2d3	0.139354
4930524B1	0.139349
Cabyr	0.139312
L1td1	0.139309
Gtsf1l	0.139246
Pkp3	0.139225
Ahnak	0.139225
Tm9sf4	0.139164
Zbtb8a	0.139152
Mfsd2b	0.139151
Slc2a5	0.139131
Uxs1	0.139105
Nrm	0.139077
Dus1l	0.139059
Zmym4	0.139024
Cyp51	0.139016
Plekhg2	0.138977
Fam50a	0.138965
Fbxo43	0.138926
Styx	0.138817
Tec	0.138748
Sub1	0.138738
Creb3l1	0.138611
0610040J0	0.13858
Rhoc	0.138577
Dnajc24	0.138565
Cenpb	0.138561
Arntl2	0.13856

Lrig2	0.138549
Cd55	0.138538
Cbr1	0.138508
Gnal	0.138469
Gabra5	0.138434
D630003M	0.138367
Tmem57	0.138351
Gpc1	0.13825
Jakmip3	0.138247
Rab38	0.13824
Ccsap	0.13819
Tmem38b	0.13816
Gnpda2	0.138152
Igsf11	0.138151
Ankrd55	0.138149
Tmprss13	0.138121
Med14	0.138115
Nrsn1	0.138114
C4b	0.13809
Map3k10	0.138079
Ncan	0.138008
1700102P0	0.138001
Apba2	0.137981
Pigk	0.137895
Npas1	0.137883
1300002K0	0.137821
Cldn23	0.137819
Ly6d	0.137743
Gsto2	0.13769
Ip6k3	0.137684
Scara5	0.137651
Sephs1	0.137607
Sumo1	0.137556
Shisa2	0.137552
Aldh1b1	0.137525
Itga10	0.137455
Zscan22	0.137416
Itih1	0.137399
Rsph1	0.137385
Unc13b	0.137362
Arl13b	0.137329
Ogdhl	0.137318
Copb1	0.137253
Olfr412	0.137204
Wipi1	0.137192
Tvp23b	0.13717
Gjb2	0.137131

Itga6	0.137112
9530077C(	0.137074
Adcy2	0.137044
Psat1	0.136984
Cpne9	0.136967
Hdhd2	0.136954
Vcpkmt	0.136951
Batf	0.136946
Cryba1	0.136905
Hmcn1	0.136831
Slc28a1	0.136807
Prim1	0.136725
Uts2r	0.136714
Cfdp1	0.136705
Dcaf15	0.136668
Ddx3y	0.136629
Bmp2k	0.136585
Csf1	0.136555
Micall1	0.136506
C4a	0.136485
Maoa	0.136391
Eya2	0.136385
Pgs1	0.136354
Zfp410	0.136323
Loh12cr1	0.136304
Camkk2	0.136258
Kif5b	0.136233
Sec16b	0.136198
Bag2	0.136128
Mnd1	0.136031
Epha6	0.136001
Manba	0.135998
Rgs9	0.135976
Zfp688	0.135944
Zyx	0.135925
Cpne5	0.135913
Gpr123	0.135866
Tmem82	0.135745
Ubl3	0.135731
Rasd2	0.135583
Aipl1	0.135527
Mbip	0.135397
Ntm	0.135331
Birc2	0.135322
Akt2	0.135316
Kcp	0.135286
Hoxd1	0.135248

Cbr2	0.135222
Fam126a	0.135198
Arfgap3	0.135184
Ptpn12	0.135142
Mark1	0.135123
Igfbp1	0.135075
Nmi	0.135065
Arpc2	0.135061
Tmem110	0.135013
Pja2	0.135002
Gprc5d	0.13499
Clec2d	0.134983
Anxa6	0.134978
Pla2g4e	0.134867
Cd93	0.13484
Ctsl	0.134817
Znrf2	0.134783
Ceacam19	0.134756
Nap1l2	0.134735
Npl	0.134723
Vill	0.134668
Gm11541	0.134665
Rhox7	0.134633
Sh2d2a	0.134551
Taf1b	0.134527
Sox18	0.134503
Foxn3	0.13441
8-Mar	0.134377
Ulk1	0.134334
Slc44a1	0.134253
Aqr	0.134243
Zfp248	0.134151
H3f3b	0.134078
Rai1	0.134046
Clip4	0.133986
Sult5a1	0.133981
Rimkla	0.133945
Syt12	0.13392
Zscan25	0.133918
Ifitm10	0.133807
Stbd1	0.133737
Mpl	0.133712
Slc9a7	0.133701
Raet1d	0.13367
Fzd6	0.133644
Ahrr	0.133641
Pink1	0.133508

Dnaja4	0.133502
Pla2g10	0.133481
Uroc1	0.133456
Trank1	0.133311
Slc28a3	0.133311
Eif2ak4	0.133249
Nat8l	0.133243
Elavl3	0.133241
Cerkl	0.133177
Phlpp2	0.133135
Rnf135	0.133117
Ccdc3	0.133096
Slc35g1	0.133088
Tnks	0.133061
Tspan2	0.132977
Pgr	0.13297
Pus7	0.132876
Eci3	0.132876
Gal3st2	0.13287
Nppb	0.132865
Blnk	0.132863
Mfn2	0.13286
Grk1	0.132814
Dkc1	0.132766
Scn1b	0.132766
Edem3	0.132761
Nelfcd	0.132635
Trub1	0.132611
Zfp488	0.132595
Mrps28	0.13249
Mfn1	0.132431
Tlx1	0.132388
Prx	0.132383
Slc25a12	0.132361
Adam11	0.132345
Rps6kc1	0.132319
Dnmt3l	0.132211
Wdr5b	0.132206
Pacrgl	0.132174
Hgsnat	0.13216
Chrnb2	0.132106
4930505A(	0.132086
Bod1	0.132083
Olfr155	0.132073
Cacna1i	0.132028
Fbxw8	0.131992
Susd4	0.131916

Lypd6b	0.131897
Zic3	0.131846
Ube2e2	0.131826
Gpatch2l	0.131783
Dsel	0.131746
Omt2b	0.131721
Apod	0.131537
Pnck	0.131531
Foxi2	0.131427
Lonrf3	0.131393
Zc3h12b	0.131321
N4bp2	0.131316
Idh2	0.131187
Ggh	0.131182
Xiap	0.131124
Nfrkb	0.131119
Col24a1	0.131047
9530091C0	0.130998
Slc12a2	0.130913
Lrrc8b	0.130835
Triqk	0.130832
Cela3b	0.130769
Cpt2	0.130727
Mdga1	0.13072
Tsc1	0.130718
Ruvbl1	0.130718
Elfn2	0.130649
Tnrc6c	0.130646
Frmd5	0.130615
Ttll6	0.130604
Hspb1	0.1306
2700089E2	0.13059
Vmn1r25	0.130573
Tgfbr2	0.130547
Paqr7	0.130545
Igsf23	0.130425
Lars	0.130403
Trim25	0.130338
Il7	0.130322
Pex14	0.130312
Pdgfb	0.130292
5730508B0	0.130291
Fbxl2	0.130266
Slc7a14	0.130247
Slc35e1	0.130217
Ston2	0.130172
Palm2	0.130166

Hrh3	0.130142
Pcyox1	0.130124
Mapk13	0.130118
D630039A(	0.130118
Pum2	0.130107
Irgm1	0.130062
Gatad2a	0.130055
Chst1	0.130037
Mme	0.130018
Ccdc39	0.130015
Sqstm1	0.130005
1110058L1	0.129991
Adad2	0.129972
Rtcb	0.129898
Fzd4	0.129895
Peg12	0.12981
Flt3	0.129795
Faxc	0.129764
Mcm9	0.129723
Zswim1	0.12972
Serpind1	0.129713
Gfi1b	0.129709
3110002H1	0.129696
Prss41	0.129589
Aph1b	0.129566
Slc17a4	0.129558
St7	0.12953
Zfp330	0.129422
Zfp85	0.129407
Esyt3	0.129365
Gas2	0.129185
Fgfbp3	0.129109
Bet1	0.129066
Ccm2l	0.129047
Chac2	0.128909
Ppapdc1b	0.128896
Dgkq	0.128807
Ear1	0.12874
Arid2	0.12869
Spo11	0.128682
Spon2	0.128679
Scg3	0.12863
8430408G2	0.128594
Gas6	0.12854
Hmgn1	0.128483
Itga2	0.128475
Alox8	0.12846

Arhgef28	0.128409
Tgif1	0.128381
Ube2w	0.128374
Nup54	0.128345
Htr5b	0.128308
Exosc3	0.128297
Rpap1	0.128209
Gm14483,	0.128172
Ehhadh	0.128141
Necab3	0.128131
Zfp583	0.128008
Cdk13	0.127992
Slc16a8	0.127963
Diablo	0.127898
Phf19	0.127851
Atp5j2	0.127819
Dhh	0.127802
Micu1	0.1278
Aplp1	0.127791
Pla2r1	0.127751
Phldb1	0.127739
Tcl1	0.127737
Cd79b	0.127666
Atl3	0.127633
Smim3	0.127591
Krtap4-7	0.127555
Aars2	0.127446
Stx7	0.127339
Cldn25	0.127317
Galm	0.127298
Snx12	0.127275
Zfp872	0.127254
Tecta	0.127209
Rasef	0.127201
Timm17a	0.1272
Usp43	0.127199
Gpr75	0.127157
Slc30a4	0.127139
Gabpb1	0.12711
Plekhg6	0.127088
Rap1a	0.127084
Mecp2	0.12707
Mtch1	0.127067
Tcf12	0.127058
Api5	0.127046
Sh3bp1	0.127019
Chuk	0.127008

Tll2	0.126951
Eng	0.126946
Nr4a1	0.126872
Vwce	0.126836
Tbk1	0.126767
Arl4c	0.126761
Lingo1	0.1267
Adra1b	0.126696
Casp8ap2	0.126635
Sema4d	0.126632
Agpat2	0.126629
Il17b	0.12649
Naaa	0.126471
Vps37d	0.126453
Ier5l	0.126405
Ankfy1	0.126397
Zfp948	0.126321
Dcbld2	0.12629
Mpdz	0.126268
Nr3c1	0.126185
Aspscr1	0.126165
Depdc7	0.126115
Zfp953	0.126088
Rpl11	0.126012
Pdyn	0.126003
Slurp1	0.12597
Ccna2	0.125948
Grk5	0.125855
Prkrir	0.125841
Svip	0.125817
Csf2rb	0.125807
Zfp607	0.125664
Chodl	0.125649
Vps13a	0.125583
Pdx1	0.125576
Plscr4	0.12557
Gmcl1	0.125535
Serpinf2	0.125518
Tsc22d2	0.125506
Coro1c	0.12549
Rbm43	0.125483
Adam10	0.125431
Dcun1d1	0.125325
4930596D(	0.125298
Fastkd1	0.125281
Ddx41	0.125271
Iah1	0.125225

Rpf1	0.125173
Slc22a21	0.125137
Syt13	0.125128
Gulo	0.125117
Fam46b	0.12509
Sowaha	0.125085
Snrk	0.125075
Rpap3	0.125009
Plk3	0.124998
Smap1	0.124997
Aifm2	0.124951
Cblb	0.124948
E230025N2	0.124942
Mapkapk5	0.124918
Ppp2r5d	0.124757
Brd7	0.124687
Kcnk12	0.124687
Tmtc4	0.124669
Smo	0.124513
Med30	0.12445
Ces3b	0.124352
Inhbe	0.124339
Mt4	0.124287
Aph1c	0.124173
Ece1	0.124146
Mob1a	0.124143
Nsmce1	0.123994
Atp2a2	0.123978
Efna5	0.123974
Atp2b1	0.123972
Chn1	0.123932
Pnpla1	0.123928
5730559C1	0.123889
Mdm4	0.123836
Ankk1	0.123666
Rag1	0.123632
Atp5e	0.123623
Tmem241	0.123587
Igf2r	0.123443
Trmt11	0.123353
Atp2b2	0.123319
Gmeb1	0.123212
Tcerg1l	0.123206
Il4ra	0.123161
Cmip	0.123129
Slc1a6	0.123103
Dtnb	0.123056

Taf7	0.123017
Dpep2	0.123
Cd300a	0.122985
Tm7sf3	0.122985
Mov10l1	0.122924
Selplg	0.122916
Rnf219	0.122906
Usp21	0.122824
Gpr4	0.122792
Entpd6	0.122776
Ibsp	0.122699
Krt83	0.122584
Taco1	0.12257
Fam188a	0.122494
Pde9a	0.122491
Ngf	0.122415
Nkain4	0.122406
Gatm	0.122324
Slc16a2	0.12231
Whamm	0.122242
Cd300lb	0.122211
Ogfr	0.122169
Myof	0.122167
Colgalt2	0.122153
Pkmyt1	0.122148
Smndc1	0.122146
Cox6c	0.122071
Grin3b	0.122055
N4bp2l1	0.122026
Olfr16	0.121978
Gnb4	0.121967
Atic	0.121953
Prss29	0.121942
Fam214a	0.121876
Smarca1	0.121838
Sod3	0.121832
Krt35	0.121749
Gmnn	0.121739
Kctd18	0.121724
Pdzrn3	0.121665
Fam150b	0.121619
Kcnh3	0.12157
6030419C1	0.121536
Acads	0.121509
Scn3b	0.121384
Klf15	0.121304
Gls	0.121291

Ube2q2	0.121199
Ncor2	0.121184
Shisa6	0.121157
Raver2	0.121104
Nxf1	0.120852
Alox12e	0.120833
Dph6	0.120752
Htr6	0.120741
Has1	0.120741
Lgals3bp	0.120738
Cd46	0.120702
Gpr125	0.120687
Adam15	0.120632
Ccne1	0.120621
Strap	0.120621
Lrp5	0.120552
Emp2	0.12049
Igsf10	0.120459
Six5	0.120389
Aox3	0.120357
Tmem237	0.120347
Arcn1	0.120276
Pde3a	0.120269
Pcnxl2	0.120256
Cul5	0.120225
2310003H(	0.120124
Rtp3	0.120114
Slc2a9	0.1201
Aff4	0.120069
Trabd	0.120056
Wdr72	0.120032
Emid1	0.119949
4-Sep	0.11989
D15Ertd62	0.119883
Pus1	0.119875
Syce2	0.11983
Mrpl33	0.119812
Kcnc4	0.11975
A830018L1	0.11974
E130309D(	0.119727
Arhgap33	0.119668
Orm1	0.119636
Mmp25	0.119619
Phf20l1	0.119606
Rpe	0.119556
Usp30	0.119449
Nol10	0.119443

Ccl20	0.119438
Ptpru	0.119395
Ccdc147	0.119357
Nup210	0.119285
Kremen1	0.119213
Bms1	0.119192
Plcg2	0.119113
Eml1	0.119109
Tmprss9	0.119071
Icos	0.11907
Lcmt1	0.119033
Ppib	0.118985
Nup98	0.118954
Ssfa2	0.11889
Ctnna1	0.118888
A230050P2	0.118887
Klhl8	0.11885
Anxa7	0.118842
Des	0.118811
Adm2	0.118807
Pwp2	0.118753
Ndfip1	0.118691
1100001G2	0.118679
Rabl6	0.118624
Tbc1d2	0.118569
Wfdc5	0.118473
Iqcf3	0.118452
Tmod4	0.118447
Ubr3	0.118419
Sla2	0.118395
Stx6	0.118376
Gpr160	0.118273
Commd6	0.118163
AI317395	0.11804
Serpinb1c	0.11799
Lgr6	0.11793
Slco4a1	0.117922
Nploc4	0.117886
Glrp1	0.117788
Nr5a1	0.117769
Rabif	0.117733
Fblim1	0.117706
Gstt3	0.117684
Nup205	0.117664
Sgpp2	0.117664
Man2a2	0.117664
Tbc1d1	0.117457

Cd38	0.117427
H2-Oa	0.117395
Mark4	0.117392
Lgi4	0.117354
Rab42	0.117336
Sh3glb1	0.117334
Fam83g	0.117254
Fbxl5	0.117252
Gpr88	0.117126
Clcn4-2	0.117097
Bpifb3	0.11708
Thbs2	0.117043
Fibcd1	0.116991
Zbtb34	0.116805
Epn1	0.116638
Rps6kb2	0.116637
Kdm3b	0.116428
Il22ra1	0.11637
Smarcd2	0.116344
Dclk3	0.116203
Ftsj1	0.1162
Etv3	0.116193
Senp5	0.116157
Icmt	0.116102
Psmc3	0.116091
Ubxn6	0.11604
Clic5	0.116002
2010204K1	0.115998
Tnfaip8l1	0.115965
Gigyf2	0.115865
Dis3l	0.115853
Dnpep	0.115833
C2	0.115784
Cdk8	0.115686
Atp8a2	0.115682
1-Sep	0.115666
Vps37b	0.115623
Trim17	0.115617
Mettl13	0.115598
Upf3a	0.115592
Ubxn11	0.115536
AI846148	0.115443
Ltbp3	0.11542
Rnf19b	0.115347
Mta3	0.115238
Chpf2	0.115225
Ehmt1	0.115223

Sbno1	0.115163
Unc80	0.115102
Tmprss3	0.11505
Dbndd2	0.115031
D1Ertd622	0.114999
Wwc1	0.114992
Slc6a11	0.114992
Lypla2	0.114935
2510002D2	0.11484
Sv2a	0.11481
Hells	0.114805
Tsen15	0.11474
Asrgl1	0.11467
Sparcl1	0.114651
Hfe2	0.114634
Prep	0.114632
Tnrc6a	0.11461
Rab37	0.114539
Fgd5	0.114519
Celf5	0.114499
Tmem17	0.114496
Bod1l	0.114485
Cd302	0.114464
Ppp1r42	0.114457
Tmem170	0.114454
H13	0.114422
Mki67	0.114358
Diras2	0.114338
Tor1a	0.114285
Dpcr1	0.114275
Trim34a	0.114275
Mrpl11	0.114214
Gpc4	0.114194
Rtel1	0.114166
Mpp1	0.11416
Anp32b	0.114149
Hamp2	0.11404
Tdrd6	0.113985
P2rx7	0.113944
Aebp2	0.113909
Prkd1	0.11387
Tmem177	0.113838
Ank1	0.113786
Pqlc1	0.113778
Zfml	0.11369
Sema4a	0.113662
Syn1	0.113659

Sapcd2	0.113612
Kdm4b	0.113584
Cdh22	0.113548
Ly6h	0.113443
Egr4	0.113355
Gfm1	0.113292
H1fnt	0.113269
1700001K1	0.113255
Pllp	0.113214
Mmp17	0.113212
Tspan15	0.113194
Sardh	0.113096
Chil1	0.113072
Epm2a	0.113016
Ncf2	0.112993
Nrip3	0.112981
Tekt1	0.11298
Gp9	0.112975
Galnt14	0.112914
Ppp1r9a	0.112901
Car8	0.11287
Nadk	0.11287
Tjp1	0.112869
Zfp362	0.112782
Gm4787	0.112775
Lrtm1	0.112732
Mocs1	0.112725
11-Mar	0.112682
Rttn	0.112652
Meaf6	0.112625
Umodl1	0.112615
Zbtb43	0.112568
Actn2	0.112505
Pycr1	0.112381
Fbp2	0.112361
C1qb	0.11236
Dusp16	0.112266
Fam133b	0.112245
Inpp5e	0.112208
Arhgap44	0.112205
Klhl7	0.112175
Tbca	0.112168
Chp2	0.112168
Calm2	0.112144
Krtap12-1	0.112108
Slk	0.112077
Agmat	0.112028

Mrpl9	0.112025
Defb36	0.11202
Prdm4	0.112007
Trem2	0.112004
Fam163b	0.112001
Armc2	0.11196
Klk14	0.111915
Gypc	0.111764
Tctn3	0.111748
Rfwd3	0.111731
Foxn2	0.111705
Spef2	0.111676
Slc7a11	0.111672
Abcb4	0.111626
Snx2	0.111595
Cilp	0.11158
Lhx3	0.111571
Gm1564	0.111568
Onecut3	0.111543
Mrps9	0.111519
Prkaa2	0.111509
Ica1l	0.111467
Abcb1b	0.111452
Tdrd3	0.111433
Cpa2	0.111409
Sbspon	0.111404
Gpr27	0.111387
Sspn	0.111372
Stxbp1	0.111296
Atp6v1e2	0.111263
Cd27	0.111262
Morn3	0.111255
Upf3b	0.11115
Gab3	0.111139
Ptpn9	0.111131
4930526D(	0.111095
Chgb	0.111074
Prodh2	0.111068
Rictor	0.111035
Cdcp1	0.1109
Slc6a19	0.110868
Svop	0.110847
Ccne2	0.110826
Ccer1	0.110786
Cldn5	0.110754
Tmem178	0.110669
Sirt1	0.110588

Zfyve27	0.110542
Slc25a47	0.110515
Fam184a	0.110437
Srcin1	0.110406
Ddit4l	0.110361
Bmp3	0.110233
Maged1	0.110201
Gsap	0.110148
Tmem89	0.110114
Me1	0.1101
Rit1	0.11009
Izumo2	0.110035
Cav2	0.110005
Hist1h4n,	0.109983
Mybph	0.109949
Plekha6	0.109749
Plb1	0.109744
Ppp1r32	0.109706
Gnl2	0.1097
Cldn11	0.109648
Jak1	0.109609
BC048562	0.109589
Ssu2	0.109418
Dnajc21	0.109405
Amigo1	0.109382
Yy1	0.109342
Impad1	0.109295
Crim1	0.109277
1700110M	0.10921
Cdh20	0.109177
Dpp10	0.109167
Tgm1	0.10915
Lman2l	0.109068
Fgf14	0.109068
Slc35b3	0.109001
Rgcc	0.108998
Bcor	0.108964
Plscr2	0.108922
Ermp1	0.10892
Gnaz	0.108879
Supt5	0.108868
Agpat9	0.10886
Traf2	0.108856
Syt9	0.108847
Lman1l	0.108821
Slc36a3	0.108819
9130230L2	0.108756

Crebbp	0.108754
Fryl	0.108708
Tlr2	0.108535
Myzap	0.108531
Ppp1r26	0.108499
Fam83f	0.108405
Ptgfrn	0.108399
Fam178b	0.108265
Tmem128	0.108254
Uba3	0.108162
2410018L1	0.108145
Ndufaf6	0.108088
4933432I0!	0.108026
Chaf1b	0.108026
Reck	0.107976
Slfn2	0.107886
Pla2g3	0.107876
Rph3a	0.107866
Cped1	0.107852
Ubxn1	0.107837
Lin54	0.107836
Unc93b1	0.10783
Car13	0.107773
Nenf	0.107724
Setdb1	0.107604
Rab7l1	0.107563
Sec61a2	0.107499
Mlph	0.107492
Myo5b	0.107487
Cntn5	0.107428
Ica1	0.107355
Mnat1	0.107304
Npr2	0.107297
Def8	0.107287
Mpv17	0.107256
Zfp185	0.107232
Tacstd2	0.10721
Fxr1	0.10716
Tff3	0.107131
Prnd	0.10712
Zdhhc18	0.107111
Zfp644	0.1071
Dlgap2	0.107069
Siah2	0.10702
Fcer1g	0.106991
Ptpmt1	0.106969
Ypel3	0.10696

Jmjd1c	0.106944
Tdrd5	0.1069
Cnga4	0.106899
Rnf113a2	0.106869
Mpzl2	0.106817
1700001P0	0.106816
3300002I0	0.106719
Asns	0.1067
Mycbp	0.106693
4922505E1	0.106684
Hddc2	0.106655
F11	0.106635
Insl6	0.106625
Scn8a	0.106591
Uhmk1	0.106523
Tmem88b	0.106518
Slc36a1	0.106477
Chrng	0.106439
Lrch2	0.106436
Bub1	0.106341
Col20a1	0.106334
Arl9	0.106305
Tmem160	0.106286
Tmem247	0.106264
Kdm2a	0.106162
Donson	0.106159
Fbxo41	0.106124
Csf2ra	0.106096
Ldhd	0.106038
Gm1123	0.105996
Rbm17	0.105989
Ubl4	0.105916
Cdc73	0.105908
Mansc4	0.105904
Dclk1	0.105821
Mien1	0.105781
Dcp1b	0.105772
Gpr85	0.105712
Ermn	0.105691
Med12l	0.105533
Myh11	0.105387
Mon1a	0.105377
Dusp12	0.10537
Tbc1d14	0.105309
Bfsp1	0.105267
Bphl	0.105246
Mboat1	0.105237

Tmed2	0.105183
Fam20b	0.10509
Agfg1	0.105078
Kras	0.105047
Pigb	0.105015
4933427D(	0.104944
Cpne4	0.104849
Nus1	0.104787
Nsfl1c	0.104745
Sult1b1	0.104713
Ddx20	0.104704
Fggy	0.104697
Tmem30a	0.104694
Hmgb1	0.104664
Cblc	0.104638
Ovol2	0.104607
Clasrp	0.104605
Ccnb2	0.104553
Msantd1	0.104389
Tbx21	0.104371
Mrs2	0.104366
Pcgf3	0.104329
1810037I1	0.104302
Kbtbd8	0.104267
Lgi2	0.104265
Kcnip1	0.104264
Rhbdl3	0.104263
Depdc1a	0.104124
Gbf1	0.104122
Dnah11	0.104085
Htra3	0.104069
Hexb	0.104056
Gdpd3	0.104027
Kcnv2	0.103982
Tnip1	0.103906
Fth1	0.103876
A430078G	0.10378
Kif20b	0.103766
Plxnd1	0.103749
Xaf1	0.103629
Htr2c	0.103378
Pds5b	0.103329
Rfxap	0.103303
Nsun7	0.103188
Gm13011	0.103156
C77080	0.103152
Rnf138	0.103106

Gpx7	0.103078
Slc17a9	0.103058
Tnfrsf14	0.103015
Sumf1	0.102981
Heca	0.102979
Capzb	0.102923
Slc6a9	0.1029
Fam110b	0.102874
Bloc1s2	0.102842
Scel	0.102832
Gtf2a1l	0.102637
D830046C2	0.102606
Myo16	0.102599
Cited1	0.102578
Dlk1	0.102557
Mfsd8	0.10255
Dapp1	0.10254
Zfp341	0.102526
Atp8b5	0.102515
Nap1l4	0.10247
Pan3	0.10244
Efcab4b	0.102439
Gm13051	0.102276
Lrrc7	0.102267
Foxj2	0.102216
Fgd2	0.102215
4930447C0	0.10221
Selt	0.10221
Aldh1a3	0.102193
Twsg1	0.102114
Elovl2	0.102065
Mab21l3	0.102006
H2-Q1	0.101972
Lamp1	0.101907
Yaf2	0.101896
4921507P0	0.101788
Dhx36	0.101754
Ilkap	0.101732
Dmc1	0.101636
Ryr1	0.101632
Cdkn3	0.10152
Lmcd1	0.101455
Kcmf1	0.101404
Pxk	0.101363
Rsph4a	0.101291
Arhgap25	0.101259
Olfr1349	0.101217

D5Ertd579	0.101203
Pip4k2c	0.101158
Plin2	0.101117
Olfr414	0.101087
Snrnp25	0.101085
Nppc	0.101084
Noto	0.10096
Glyctk	0.100935
Wfdc13	0.10085
Zfp618	0.100742
Scn9a	0.100688
Pbx3	0.100587
Ano1	0.100553
2-Mar	0.100547
Xpo6	0.100497
Pygb	0.100451
7-Mar	0.10044
Atxn2l	0.100426
1110008P1	0.100379
Actl6a	0.100332
Zfp54	0.100273
Naaladl1	0.100262
Adam23	0.100201
Atp2c2	0.100144
Slc6a3	0.100123
Rhbg	0.100108
Bcorl1	0.099998
Fam71b	0.09999
2510039O1	0.099976
Gm10406	0.099805
Nrgn	0.099801
Nme8	0.099764
Zfp52	0.099758
Ebi3	0.099727
Dhdh	0.099717
Dennd2c	0.099707
Cd63	0.099626
Dlgap4	0.099598
Cyp2s1	0.099591
Kbtbd2	0.099588
Naa11	0.099462
Grb14	0.099451
2310033P0	0.099403
Osbpl11	0.099295
Plekhf2	0.09929
Ppp5c	0.099252
Ldlrad2	0.099187

Arl8b	0.099177
Trpv1	0.099065
Lefty2	0.099051
Rab17	0.099049
Tfcp2l1	0.099042
Sorl1	0.099009
Upk3b	0.099007
Hkdc1	0.098944
Tbc1d12	0.098899
Ncf1	0.098899
Atp8b3	0.098894
Rybp	0.098858
Aqp1	0.098857
Piwil4	0.098851
Rgs19	0.098809
P4ha3	0.098789
Tor4a	0.098786
Ocm	0.098782
Vav1	0.098715
Irak2	0.09871
Fbll1	0.098681
Fbxo4	0.098536
Cyp7b1	0.098497
Gpr113	0.098484
A930018P2	0.098464
Mns1	0.098429
Lama2	0.098419
Mtnr1b	0.098407
Apoa5	0.098405
Gpr137b	0.09835
Tpo	0.098313
Cd82	0.098283
Ttll8	0.098156
BC048507	0.09814
Lgals12	0.098136
Ihh	0.098101
Otub1	0.09803
Rimbp2	0.097886
Sqrdl	0.097886
Tnnt3	0.097818
Gcnt1	0.09768
Tbc1d21	0.09765
Olfr324	0.097577
Zbp1	0.097547
Parp8	0.097543
Mcur1	0.097513
Nkx6-1	0.097499

Cdkn1c	0.097344
Mbnl3	0.097343
Gng4	0.097238
Cdan1	0.097219
Zcchc6	0.097174
BC006965	0.097166
Emr1	0.097114
Cplx2	0.097021
Ssr2	0.097005
Kcnq1	0.097004
Gcfc2	0.096973
Zmynd19	0.096972
Cyp4f40	0.096957
Caln1	0.096868
Tekt5	0.096761
Ptbp2	0.096628
Wbp2nl	0.096626
Cntln	0.096568
Magix	0.096501
Sgsm3	0.096425
Trpv4	0.096418
Abi1	0.096397
Adipor2	0.096371
Taok1	0.096286
Timp3	0.096284
Dvl1	0.09627
Rhou	0.096211
Clpsl2	0.096207
Dmxl1	0.096177
Calhm1	0.096158
Mapk8	0.096148
Trpc4	0.096143
Camkk1	0.096001
Astn2	0.095988
Epha1	0.095975
Arhgef39	0.095971
Apoc4	0.095866
Fam71e2	0.095849
Ankrd35	0.095803
Ltk	0.095725
Becn1	0.0957
Accsl	0.095697
Agbl2	0.095644
Kif5c	0.095585
Tbc1d20	0.095547
Cdkn2aipnl	0.095542
Fgf23	0.095444

Kif12	0.095401
Myl9	0.095331
Ghdc	0.095304
Abca13	0.095295
Cdk15	0.095284
Crtap	0.095205
Pde4b	0.095157
Rbmx2	0.095155
Sox3	0.095091
Tmc2	0.095023
Rc3h1	0.094817
Abca5	0.094787
Zfp820	0.094728
Nipal2	0.094722
Pnpla5	0.094701
Exoc4	0.094621
Lap3	0.094591
Chd1l	0.094586
1110051M	0.094573
Vamp5	0.094515
Spata6	0.094469
Lamtor5	0.094456
1700066B1	0.09443
Bglap	0.094423
Lcn10	0.094361
Sowahd	0.094285
Homer1	0.094182
Gal	0.094164
Ankrd53	0.094077
Zar1	0.094017
Ccr7	0.093979
Trim62	0.093893
Gm527	0.093869
Lrrc4b	0.093825
Igfbp7	0.093781
Mapk3	0.093748
Prpsap2	0.093634
Slc6a1	0.093549
Hiatl1	0.093452
Slc4a3	0.093435
Astn1	0.093356
Asb10	0.093341
Htt	0.093307
Apbb1	0.093285
Actr5	0.09323
Osbpl5	0.093198
Srl	0.093119

Pomc	0.093118
Mogat2	0.093117
BC051628	0.093113
Mtfr1l	0.093098
Aldh8a1	0.093032
Egfem1	0.092994
Rnase6	0.092949
P2rx2	0.092922
Gpr133	0.09281
Slc24a2	0.092772
Rsl1	0.092675
Slc25a26	0.092674
Clip1	0.092669
Ddx11	0.092647
Pdcd2l	0.092557
Klk1b22	0.092551
Ccdc112	0.092537
Olfr109	0.092428
Dhrs2	0.092395
Atp6ap1	0.092395
Lime1	0.092287
Spint1	0.09226
Clvs2	0.092257
Fbxo25	0.092181
D130043K2	0.092127
Nlrp10	0.092098
Hoga1	0.092084
Rnf186	0.092045
Adamtsl4	0.092039
Mettl14	0.092033
Fam180a	0.092029
Zfp606	0.092024
Gm12597	0.092014
B930041F1	0.092008
Pclo	0.091986
Scgn	0.091982
Ccdc88b	0.091956
Pitpnm3	0.091942
Nt5c1a	0.091892
Ube2u	0.091855
Trim68	0.091823
Golga1	0.091778
Plekhs1	0.091776
Irf6	0.091719
Tat	0.091705
Hpn	0.091683
Gldn	0.09163

Mmp21	0.091611
Slc10a3	0.09161
Strip2	0.091504
Nox4	0.09146
Entpd1	0.091453
Rimklb	0.091401
Tmem100	0.09137
Cobl	0.091331
Tmem9b	0.091311
Mx1	0.091289
Tusc5	0.091218
Mgea5	0.091211
Kif22	0.091156
Fam163a	0.091135
Slc6a8	0.091032
Sstr5	0.090989
Celf1	0.090911
Tbx5	0.090899
Bok	0.090811
Pygm	0.09081
Larp4b	0.09081
Cacul1	0.090808
Klhl20	0.090798
Pex7	0.090745
Cand2	0.090707
Tas1r3	0.090689
Npy	0.090663
Gpr158	0.090643
Myom3	0.090555
Cacng2	0.090542
Snta1	0.090488
Grin3a	0.090413
Tecrl	0.09039
Prpf40b	0.090299
Grm7	0.090287
Med11	0.090246
Zfp641	0.090234
Cd19	0.090182
Arglu1	0.090147
Arhgef37	0.089993
Erp27	0.089984
Fgf20	0.089965
Rps15a	0.08987
Cd52	0.089852
Fuca2	0.089812
Pomp	0.089761
Bhlhe23	0.089739

Ccdc63	0.089635
Phc3	0.089498
Haus1	0.089476
Sys1	0.089461
Gpr83	0.089415
Nudc	0.089396
Pkn2	0.089357
Stim1	0.089236
Pdzd8	0.089232
Slc51a	0.08917
Bahd1	0.089057
A430005L1	0.089044
Kcnb1	0.088954
Odf4	0.088936
9930021J0	0.088927
Gsn	0.088909
Krt82	0.088888
4932438H2	0.088879
Prok2	0.088845
Olfr370	0.088835
Gsg1l	0.088802
Vmn1r47	0.088733
Spib	0.088729
Apoc3	0.088558
Rac2	0.088549
Fkbp9	0.088548
Zfp579	0.088532
Zfp771	0.088504
2300002M	0.088471
Cyfip2	0.088389
Zfp169	0.088356
Elfn1	0.088325
Lypla1	0.088315
Tnfrsf22	0.088218
Prrg3	0.088107
Lipa	0.088104
Tmem189	0.0881
Slc18a2	0.088085
C330007P0	0.088078
Prss50	0.088055
2310009B1	0.08799
Crls1	0.087984
Anxa8	0.08785
Cd1d2	0.087837
Paqr3	0.087796
Myo1g	0.087785
Itgb4	0.087777

Usp24	0.087681
6330403K0	0.087619
Lcn12	0.087583
Gm216	0.087565
Cd8b1	0.087561
Kcnj12	0.087412
Ltf	0.087393
Cnnm1	0.087361
Carns1	0.087308
Gm8884	0.087304
Gpr89	0.087068
Fam166b	0.087056
Ctbs	0.087016
Chac1	0.087009
Ncoa2	0.086967
Aldh2	0.086964
Tmcc2	0.08696
Hykk	0.086891
Dnajc28	0.08687
Pfdn1	0.086837
Sbk2	0.086749
Gpha2	0.086743
Tiprl	0.086703
Chml	0.086632
Prdx3	0.086527
Sall3	0.086479
Eras	0.086447
Dysf	0.0864
Tmem14a	0.086245
Dag1	0.086212
Myf5	0.086207
Zfp697	0.086205
Krtap13	0.086205
Clec14a	0.086168
Tas1r2	0.086139
Gm15698	0.086085
Dedd	0.086085
Lrp10	0.086042
Mtmr3	0.086038
Hgf	0.085977
Ceacam2	0.085933
Slco2b1	0.08592
Stau2	0.085898
Acat1	0.085852
Slc6a7	0.085834
Clec4g	0.085804
Pcp4	0.08573

Tm4sf5	0.08565
Kctd5	0.085598
Sec16a	0.085439
Arhgap29	0.085438
Zc3hav1l	0.085378
Cntnap5c	0.085374
Sohlh1	0.08534
Tnnc2	0.085261
Cnksr1	0.085221
Cryl1	0.085156
Ak5	0.085135
Mtx3	0.085038
Gm973	0.085011
Lama4	0.085003
Ss18	0.084982
Mysm1	0.084968
Hectd2	0.084877
2610507B1	0.084845
Gnpda1	0.084822
Cited4	0.084816
Lrrc63	0.084802
Sult4a1	0.084799
Prlh	0.084724
Gltp	0.084718
Krt72	0.084661
Ticam2	0.084639
Larp6	0.084627
Tada2a	0.084584
Syce1l	0.084537
Cdkl2	0.08447
Rbm14	0.084422
Mknk1	0.084205
Rims1	0.084122
0610010F0	0.083969
Abca9	0.08396
Ly6g5b	0.083703
Dcp1a	0.083587
Alox5	0.083573
Jdp2	0.083449
Exoc6	0.083393
Olfr524	0.083335
Galp	0.083328
Adcyap1	0.083307
Rnf38	0.083305
Cyp21a1	0.083295
Trim38	0.083006
Cntnap3	0.082968

Micall2	0.082867
Fas	0.082856
Tmem196	0.082745
Zfp946	0.082732
Coq10b	0.082727
Snx31	0.082718
Mag	0.082664
Eid2	0.082611
Usp33	0.08257
D030056L2	0.082551
Zar1l	0.082495
Gfra4	0.082481
Shfm1	0.082422
Itih5	0.082322
Zyg11b	0.082266
Rtdr1	0.082254
1700011A1	0.082196
Crlf2	0.082119
Ywhab	0.082112
Zfp282	0.082105
Mcidas	0.081999
Cdo1	0.081964
Kcnk18	0.081956
Pdpr	0.08192
Gtf3c3	0.081887
Snx16	0.081812
Atrnl1	0.081807
Neurl1a	0.081779
Serpina11	0.08164
Dcaf12l1	0.081632
Chst7	0.081612
Tmem158	0.081598
Tnfrsf1b	0.081424
Ptpre	0.081392
4930539E0	0.081329
Sfmbt1	0.081271
Nek5	0.08119
Pex26	0.081153
Per3	0.080903
Mier1	0.080829
L1cam	0.080803
Nup35	0.080698
Mybpc2	0.080698
Prkcdbp	0.080655
Gabrr3	0.080649
Paqr6	0.080539
Rint1	0.080528

Cyp4a12b	0.080408
Aqp12	0.080367
Usp6nl	0.080185
Pate4	0.080125
Degs1	0.080125
B4galt5	0.080028
Pi4kb	0.079954
Cyb561d1	0.079938
Taf4b	0.079892
Slc25a46	0.07989
Slc25a41	0.079838
Eddm3b	0.079802
Ftcd	0.079695
Enpp2	0.079687
Rasgef1c	0.079684
Acot5	0.079667
Tprn	0.079573
Ago4	0.07955
Nfib	0.079547
Tmem56	0.079506
Mrps30	0.07949
Chd5	0.079484
Mettl9	0.079394
Tspan33	0.079393
Tuba8	0.079388
Pxt1	0.079335
Vps33a	0.07932
Col8a2	0.07932
Pid1	0.079272
Olfr1350	0.079228
Krtap9-1	0.079226
Nfyc	0.079139
Slc35a2	0.079138
Trim12c	0.079124
Myl2	0.07883
B4galt6	0.078828
Gars	0.078786
Rab43	0.078734
Zfp105	0.07867
Rps6ka4	0.078604
Zfp57	0.078603
Il17rb	0.078586
Slc25a43	0.078568
Fam131c	0.07847
Kank1	0.07844
Tpcn2	0.078397
Slc17a3	0.078379

Xkr8	0.078325
Magi2	0.078325
Gba	0.078256
Nts	0.07824
Slc45a1	0.078147
Olfr522	0.078088
Igfbpl1	0.078074
Vps41	0.078049
Tnfaip6	0.077912
Tmem232	0.077898
AK129341	0.077856
1700071K0	0.077548
Ttc4	0.077547
Tpst1	0.07748
Arhgef26	0.077439
Odf3l1	0.077425
Slc46a2	0.077326
Tmc4	0.077319
Setx	0.077301
Myoc	0.077277
Ksr2	0.077276
Smu1	0.07727
H2-Aa	0.077138
Henmt1	0.077064
Bmp8a	0.077053
Prdx6	0.077024
Abhd12	0.076949
Taar5	0.076819
Epb4.1l3	0.076805
Arhgef11	0.076752
Slc35f1	0.076738
Trappc8	0.076726
Gimap3	0.076666
Ckm	0.076643
Ifih1	0.076637
Slc15a4	0.07663
Ms4a15	0.07657
Itgb3	0.076491
Cnga3	0.076425
Abl2	0.076306
Lypd5	0.076278
Ermap	0.076263
Bend6	0.076225
Ccdc101	0.076138
Magee1	0.0761
Ccl26	0.07606
Vps54	0.076026

Spns3	0.075995
Zxdc	0.075985
AU022751	0.075945
Zfp365	0.075778
Pstpip2	0.075691
Tspan9	0.075601
Mal2	0.075596
Eefsec	0.075594
Gclm	0.07556
Olfr1344	0.075476
Man2b2	0.075451
Tmem70	0.075433
Rabgap1	0.075362
Psg17	0.075263
Ikzf1	0.075253
Hesx1	0.075182
Ube3c	0.075164
Mn1	0.075089
Pik3r5	0.075069
Ift88	0.075012
Hnrnpu	0.074937
Ranbp3	0.074856
Stk39	0.07482
Ranbp6	0.074765
Rsad2	0.07476
Paqr8	0.074745
Zfp385b	0.074725
Tnfrsf18	0.074674
Gadd45gip	0.074631
Angpt2	0.074534
Gja5	0.074487
Papl	0.07448
Scd3	0.074318
Tmem183a	0.074307
Aox1	0.074277
Tab2	0.074267
Ankfn1	0.074233
Gm597	0.074176
Zfp692	0.074127
Cela2a	0.074027
4833424O1	0.073991
St8sia6	0.07397
Tusc1	0.073936
Gpr50	0.073923
Tex12	0.073776
Rd3	0.073772
Cth	0.073751

Klf9	0.073741
En2	0.073708
Tdo2	0.073703
Lpcat4	0.073627
Zfyve28	0.073564
S100a10	0.073485
Bnipl	0.073474
Syt16	0.073454
Klhl24	0.073446
Eid1	0.073425
Cyp2d22	0.073337
Adora3	0.073195
Cttnbp2	0.07319
Adra2c	0.073172
Uty	0.073063
1810030O(	0.073045
Spef1	0.072899
Ifi27l2a	0.072861
Galnt12	0.072856
Prpf19	0.072799
Pygl	0.072732
Il15	0.072695
Homez	0.072694
Dab2ip	0.072612
Klk6	0.072565
Lpgat1	0.072535
Cldn26	0.072528
Ky	0.07243
Pi4k2a	0.072412
Lysmd4	0.072388
Zfp760	0.07226
Txndc5	0.072089
Selenbp2	0.072063
Chrna4	0.072046
Gbas	0.072005
Shcbp1	0.071997
Defb13	0.071988
Cyp4f16	0.071951
Map2k4	0.071773
Rhod	0.071563
Adck1	0.071563
Trf	0.071536
Agpat3	0.07146
Spaca7	0.071445
Fads3	0.07133
Padi1	0.071239
Ralgapa2	0.071163

Tmem52	0.071146
Padi3	0.071098
Itm2c	0.071085
Emp1	0.071057
Lamc2	0.071028
Cnih2	0.071023
Tnk1	0.071009
Zbtb7c	0.071
Ndufab1	0.070953
Impact	0.070918
Olfr571	0.07089
Stx3	0.070867
Agxt	0.070643
Pla2g5	0.070619
Apold1	0.070459
Tspo	0.070309
2010300C0	0.070306
Pglyrp1	0.070273
Edem1	0.070262
Pdgfa	0.070124
Slco4c1	0.07001
Gm853	0.069991
Prss27	0.069977
Bhlha9	0.069937
Tmem72	0.069934
Olfr448	0.069914
Tcerg1	0.069876
Tmprss2	0.069815
D030018L1	0.069715
Vmn1r200	0.069633
4932411E2	0.069515
Ociad1	0.069426
Sp3	0.069426
Zfp595	0.069416
Zfp119a	0.069151
8430419L0	0.069126
Rhox4g	0.069077
Exog	0.069069
Snx8	0.069004
Plekhb2	0.068909
Klf13	0.068843
Sectm1a	0.068792
Fyttd1	0.068789
Chrna2	0.068756
Rps6	0.06874
1500015O1	0.068686
Lrfn3	0.06866

Optn	0.068579
Olfr561	0.068553
Smad7	0.068521
Lrpprc	0.068509
Prop1	0.068474
Msh6	0.068449
Lmo2	0.068343
Cyth3	0.068342
Metap1	0.068237
Ak4	0.068184
Fscn3	0.068165
Btbd11	0.068102
Irf2bp2	0.067998
Lrba	0.067991
Pmm1	0.067968
Trim58	0.067877
Tamm41	0.067844
Tfap2e	0.067838
Olfr373	0.067763
B3galnt1	0.067755
Ltbr	0.067739
Ctnnal1	0.067661
1700031M	0.067653
Utf1	0.067496
Zkscan3	0.067481
Thoc1	0.067395
4930563D2	0.067377
Ifi47	0.067326
Mcu	0.067246
Iqgap1	0.067217
Bnc1	0.067214
Tnfrsf8	0.067205
Mvb12b	0.067158
Tm9sf3	0.067125
Eps8l1	0.067099
Prss38	0.067055
Mamdc2	0.067036
Dppa4	0.066924
Olfr538	0.066897
Brd3	0.066891
Nckap1	0.066873
Parp16	0.066826
Zfp770	0.066725
Tshz3	0.066724
Rbmxl2	0.066703
Tceb3	0.066688
Lhfpl4	0.066582

Allc	0.066535
1700029P1	0.066483
Ccdc135	0.066462
Hapln2	0.066434
Tmem135	0.066363
Aes	0.066294
Prss30	0.066255
Sorcs2	0.066058
Phf23	0.066052
Rspo4	0.066022
Nkx2-6	0.066014
Tes	0.065935
Atp9a	0.065804
Kcnq2	0.065798
Sh2b3	0.065701
Cldn20	0.065591
Xirp1	0.065574
Corin	0.065555
Ctsw	0.065544
4732456N1	0.065529
Fbxo28	0.065514
Glb1l3	0.065463
Gcsam	0.065326
Tollip	0.065177
Plek2	0.065082
Cntnap2	0.065049
Npnt	0.064993
Cox6b1	0.064981
Ctsz	0.064961
Evi5	0.064913
Scn5a	0.064839
Ccndbp1	0.064813
Vac14	0.064769
Ctxn3	0.064695
Olfr773	0.064693
Ern2	0.064638
Mfsd4	0.06463
Pvrl4	0.064607
Edn2	0.064604
Otof	0.064491
2810021J2	0.064488
Kcna5	0.064466
Adamtsl2	0.064451
Zfp786	0.0644
Camk1	0.064372
Slc9a3	0.064338
Rnf20	0.064303

AI854703	0.064276
Lzts3	0.064164
Morf4l1	0.064026
Robo4	0.063959
Soat1	0.063955
Luzp1	0.063929
Abcg3	0.063902
Lrp2bp	0.063807
Srsf7	0.063802
Cd180	0.063733
Napsa	0.063723
Naa40	0.063712
Nr1i3	0.063683
Wdtc1	0.063679
Trim63	0.063671
Gfra3	0.063614
Krtap4-16	0.063611
Abcc3	0.063596
Myo1b	0.063511
Arl8a	0.063457
Golt1a	0.063456
Asap3	0.063324
Ddx18	0.063302
Top1	0.063284
Scd1	0.063183
Il1f5	0.063136
Car9	0.063118
Gm5414	0.063034
Xrcc6bp1	0.063028
Slfn5	0.062976
F2	0.062733
Prr16	0.062645
Gm12942	0.062639
Bptf	0.062372
Mmp2	0.062367
Il6ra	0.062322
Mios	0.062314
Dap3	0.062273
Gjc3	0.062195
Rbm33	0.062162
Odam	0.062113
Ccl24	0.062024
Hs6st2	0.061918
Olfr877	0.061843
Rph3al	0.061815
Ahsg	0.061766
Gm6251	0.061758

Tomm22	0.061594
Cldn4	0.061592
Trpc4ap	0.061581
B2m	0.061577
Lrrc6	0.061569
Kcnj9	0.061567
Prss32	0.061504
Tm4sf4	0.061444
Ctf2	0.061431
Dicer1	0.061326
Tgm2	0.061227
Psd4	0.061138
Rhox4e	0.061109
Dclk2	0.06101
Lce3b	0.060984
Stmn2	0.060951
Smad9	0.060913
Cox8c	0.060877
Padi4	0.060833
Maneal	0.060736
Arrdc1	0.060719
Abhd17c	0.06069
Ipo7	0.06064
Imp3	0.060633
Ube2e3	0.06059
Sumf2	0.06051
Cap2	0.060345
Wdr63	0.06032
Plekhm2	0.060307
Col9a2	0.060281
Mrap	0.060244
Fbxw4	0.060179
Prkcz	0.060126
Cacna1b	0.060068
E130201H(	0.060003
5031439G(	0.059961
Dsc2	0.059953
Smpdl3a	0.059944
Slc6a17	0.059922
Nobox	0.059882
Wnt8b	0.059858
Kcna2	0.059766
Olfr24	0.059747
Wfdc15b	0.059729
Lum	0.059729
Tlr3	0.059659
Ramp1	0.059634

Mrvi1	0.059523
Gcnt7	0.059468
Acsbg1	0.059429
Ninj2	0.059386
4932438A1	0.059304
Fam196b	0.059255
Mlf1	0.059191
Slc27a6	0.059002
Rab15	0.058867
Tspan1	0.058759
Nlrx1	0.058758
Anxa9	0.05873
Traf3ip1	0.058713
Cd3e	0.0587
Agps	0.058639
4930590J0	0.058546
Il21r	0.05852
Tnfrsf17	0.058424
Pgm2	0.058359
Cyp4a12a	0.058317
Vmn1r87	0.058234
Itgb2	0.058223
Hrh4	0.058202
Klhl41	0.058197
Il3ra	0.058181
C130060K2	0.058158
Slc11a2	0.058147
Tmbim7	0.058123
Smim8	0.058117
Cntn1	0.058019
Mzb1	0.057989
Ddx60	0.057982
1110057K0	0.057805
Dnm1l	0.057743
Olfr94	0.057737
Eif3e	0.05773
Acsl1	0.057685
Vmo1	0.057624
Fam175b	0.057564
Yipf5	0.057489
Tbc1d8	0.057487
Uchl4	0.057432
Gadl1	0.057378
Gsto1	0.057372
Olfr982	0.057349
Slc39a2	0.057268
Tuba4a	0.057267

Pla2g4d	0.057243
Slc22a18	0.056983
Nmu	0.056968
Fam110c	0.056956
Skint3	0.056909
Fam213a	0.056875
Zfp518a	0.056847
Cadm3	0.056769
AU021092	0.056493
Btnl4	0.056444
Efcab6	0.056426
Klrg2	0.056241
Rxfp4	0.056201
Lrat	0.056163
Lrrc10	0.056144
Naa16	0.056044
Iqcf1	0.056003
Lrrc8e	0.056001
2010003K1	0.055909
5-Sep	0.055884
Olfr1392	0.055878
Phyhd1	0.055861
Rgs3	0.055841
Syce1	0.055834
Arpc5	0.055802
S100a16	0.055755
1700067P1	0.055716
Ccdc181	0.05571
Ston1	0.055541
Thap3	0.055525
Phactr1	0.055494
Ccdc65	0.055454
Acsl5	0.055445
Ttll3	0.055444
Gria1	0.055405
Rab3il1	0.055386
Zfp711	0.055352
Sfxn4	0.055332
Hormad1	0.055137
1700023E0	0.055076
Ptgs1	0.055059
Kcns1	0.055022
Itga11	0.054976
Cdk14	0.054959
Tnc	0.054952
Qsox2	0.054921
Dmrt1	0.05468

Wdr74	0.054672
Slc22a7	0.05463
Slc16a7	0.054523
Trpv2	0.054501
Tmem8c	0.054469
Foxe3	0.054461
Ccl22	0.054423
Tacr3	0.054408
Lrriq1	0.054395
Mxd1	0.054373
2410018M	0.054358
Lin9	0.054177
Sec62	0.054048
Ceacam16	0.054018
Rassf9	0.054004
Iws1	0.053965
Bsnd	0.05395
Tmem9	0.053928
Ccnyl1	0.053874
1700080E1	0.053751
Daw1	0.053691
Zfp777	0.053566
Synm	0.053556
Olfr653	0.053547
Csnk2a2	0.05354
Upk3a	0.053393
Upb1	0.053239
Dnah5	0.053218
Tmem30b	0.053191
Olfr8	0.053188
Ccdc64	0.053082
Arhgef38	0.053031
Dennd1c	0.052991
Ero1lb	0.052984
Snhg11	0.052968
Hipk3	0.052905
Krt71	0.05287
Ccin	0.052841
Postn	0.052775
Syt4	0.052755
Nbea	0.052748
Epb4.1l1	0.052668
Sec31b	0.052644
Akr1b8	0.052548
Nyap2	0.052537
Tmem91	0.052471
Slc47a2	0.052466

Atp12a	0.052407
H2-M5	0.052383
Hs3st2	0.052362
Pter	0.052285
Garem	0.052092
Crtam	0.051925
Osbpl1a	0.051886
Scgb1a1	0.051843
Adrb1	0.051765
Hs3st6	0.051695
Emc2	0.051691
Crygc	0.051641
Slc9a9	0.051606
Slc5a4a	0.051605
Pla2g2e	0.051577
Prss43	0.051565
Cbln4	0.05155
Prkag3	0.051346
Prmt6	0.051315
Ppp1r14d	0.051283
Vmn1r222	0.051215
Rab11fip2	0.05117
Pyurf	0.051017
Tas2r144	0.050973
Klk13	0.050893
Rbp4	0.05089
Fermt1	0.050771
Ppapdc3	0.050766
Olfr148	0.050737
Fosl2	0.050632
Dcaf12l2	0.050515
Idi2	0.050446
Rhox4c	0.050404
Zfp954	0.05039
Cebpg	0.050358
Aar2	0.05033
Ccdc166	0.050245
Kcnq3	0.050189
Guca2b	0.050147
Clstn2	0.05011
Chpt1	0.050059
Olfr316	0.050029
Jazf1	0.05
E2f2	0.049942
Htr3b	0.049916
Serpinb6a	0.049891
Arhgef4	0.049849

Nfe2	0.049705
Dapk2	0.049667
Cuzd1	0.04958
Cfb	0.049571
Fam102a	0.049488
Sh2d4b	0.049482
Cmas	0.049478
Tex10	0.049454
Tmprss12	0.049397
Zkscan8	0.049363
Pkdrej	0.049136
Mrgbp	0.049079
Nostrin	0.049077
BC055111	0.048989
Slc22a20	0.048921
6330419J2	0.048899
Hebp1	0.048861
Lrrtm2	0.04884
Abca4	0.048786
Il1r2	0.048685
Hfm1	0.048664
Mrpl16	0.048609
Glyatl3	0.048473
Slc36a4	0.048282
Bsg	0.048245
Pabpc4l	0.048201
Tmbim1	0.048072
Htr5a	0.048034
Ptpn2	0.047848
1-Mar	0.04784
Bend4	0.047805
Map3k4	0.047711
Capza2	0.047683
Gm4956	0.047651
Mgst2	0.04764
Pilrb2	0.047625
Tmem165	0.047584
Krt78	0.047492
Kcnj6	0.047418
Bdkrb1	0.047305
Ido2	0.047289
Il20ra	0.047287
Tmem54	0.047263
Dlg3	0.047252
Slc19a3	0.047149
Cyct	0.047012
Klrb1f	0.046929

Smpdl3b	0.046857
Dlg5	0.046599
Traf6	0.046573
Agtr1b	0.04654
Pga5	0.046447
Eps8l3	0.046334
Myh7	0.046259
Cpvl	0.046199
Mesp2	0.046193
Rev1	0.046113
Iqub	0.046066
Fcgrt	0.045883
Cd84	0.045877
Slc6a13	0.045775
Cox6a2	0.045728
Scin	0.045699
5730409E0	0.045689
Dnaaf3	0.045632
Pgbd5	0.045554
Hid1	0.045554
Sycp2	0.045459
Gbp7	0.045439
Lage3	0.04542
Tnfrsf11a	0.045412
Zfp959	0.045403
Prpf3	0.045398
Galnt1	0.045372
Naa20	0.045297
Ptpn11	0.045279
Slc22a14	0.045267
Myt1	0.045189
Med10	0.045082
Xk	0.045075
Itih3	0.045069
Gast	0.045015
Ceacam18	0.044977
Fmo2	0.044875
Atmin	0.044833
Tmem74b	0.044745
Plin1	0.044694
Rad54b	0.044602
Avpr1b	0.044506
Ctrl	0.044467
Dnajc19	0.044418
Slc34a2	0.044314
Gapvd1	0.044201
Cacng3	0.044137

Ndufaf1	0.044135
Lipe	0.0441
Pomgnt1	0.043866
Pou1f1	0.043813
Daam2	0.043807
Oit1	0.043596
Cd86	0.043592
Bcl2l10	0.043565
Il17d	0.043522
Spats1	0.043509
Defb23	0.043497
Klk7	0.043437
Lrrc3b	0.043409
Wnt7b	0.043321
9130008F2	0.04326
Calcr	0.043222
Olfr527	0.043121
Mbd1	0.04312
Rbm4	0.04305
9230104L0	0.04299
Lgals2	0.042983
2310005G1	0.042943
Sphkap	0.042753
Nars2	0.042626
4933422H2	0.042603
Mfsd6	0.04248
Mfsd9	0.042241
Tex19.1	0.042118
Sell	0.042079
Zfp212	0.042012
Zdhhc13	0.042008
Npy4r	0.041949
Fkbp1a	0.041921
Olfr677	0.041799
Tac2	0.041796
Hrh2	0.04176
Prss52	0.041737
Magohb	0.041559
Abcb1a	0.041473
Inhbc	0.041368
Pramel1	0.041308
Slfn9	0.041282
Gpr65	0.041273
Serpina10	0.04124
Taar9	0.041205
Olfr156	0.041141
Tmed6	0.04114

Nlrc3	0.041103
Gm4794	0.041077
Tmem5	0.041048
Pld5	0.041028
Hnf4a	0.040945
Tead4	0.040906
Cyp2j6	0.0409
Eps8	0.04076
Ptger3	0.040606
Pde6g	0.040545
Grm5	0.040467
Camk2n1	0.040417
Dctn1	0.040417
1700024G1	0.04035
Ndufb8	0.040338
Pcdhb19	0.040337
Olfr981	0.040018
Arhgef5	0.039857
Wdr26	0.039832
Nmbr	0.039743
Paox	0.039696
Stard8	0.039621
1700003M	0.039609
Sec13	0.039603
Tnfrsf13b	0.039552
Eif2d	0.03955
Hormad2	0.039503
Ptafr	0.03947
Rbms2	0.039446
Defb29	0.039407
Kl	0.039398
Ago3	0.039387
Adtrp	0.039341
Nupl2	0.039318
Fgl2	0.039255
A2m	0.039028
Efhd2	0.039024
Agr2	0.038973
Podn	0.038884
Phxr4	0.038694
Relb	0.038659
Cd6	0.038649
Taar7f	0.038618
Cdk9	0.038508
Ap1ar	0.038489
Il22ra2	0.038471
Prss44	0.038314

Zfp709	0.038261
Vps37c	0.038174
Olfr523	0.038155
Serpinb1a	0.037967
Palmd	0.03785
Dnajb3	0.037831
Cul3	0.037798
Cnpy1	0.03768
Scn2a1	0.037676
Zbtb12	0.03734
Plbd1	0.037317
Mas1	0.03713
Mtl5	0.037115
Btf3	0.037071
Htatsf1	0.037031
Wdr60	0.036932
Guca2a	0.036866
Calhm2	0.036854
Cdc16	0.036785
Gabrb3	0.036644
Gca	0.036623
Krt75	0.036453
Palld	0.03643
Vmn1r1	0.036371
Birc3	0.036255
Vmn1r188	0.036204
Syndig1l	0.036153
Vmn1r11	0.036113
Patl1	0.036056
9130401M	0.035984
Xpr1	0.035975
1700012P2	0.035939
Mxi1	0.035887
Nrg4	0.035878
Hmox2	0.035809
Nr0b1	0.035785
Acaa1b	0.035777
Asb14	0.035764
Vsx1	0.035491
Kcnn4	0.035469
Slc5a8	0.035333
Lce3f	0.035322
Scrg1	0.035225
Scml4	0.03519
Isoc1	0.035146
Mfap2	0.035145
Slc22a3	0.035011

Adig	0.034912
Nkx3-1	0.034818
Camkv	0.03464
Synb	0.034611
Mpp7	0.034521
Fgd3	0.034471
Arhgap4	0.034467
Tnfsf14	0.034411
Ano2	0.03434
Psmc4	0.034333
Il3	0.03424
Sprr2e	0.034207
9030617O(	0.034197
Tmem39b	0.034187
Tex29	0.034177
Pabpc2	0.034129
Pld4	0.034121
Bmp8b	0.034075
Areg	0.034069
Tas2r143	0.034032
Cmbl	0.03397
Lmtk2	0.033728
Nfu1	0.033696
Mansc1	0.033693
Olfr95	0.033654
Gabre	0.033627
Efcab12	0.033597
Myh14	0.033534
Pparg	0.033507
Armc12	0.033464
Ffar3	0.033463
Olfr67	0.033443
Fbxo39	0.033401
Abcb11	0.03338
Prrc1	0.033347
Inhbb	0.033283
Mfi2	0.033261
H2-M3	0.033246
Epb4.1l4b	0.033215
Sbno2	0.033181
Acpp	0.033001
Ankef1	0.032741
Fbxo11	0.032738
Pdrg1	0.032708
Rims3	0.03261
Zcchc13	0.032475
Zkscan16	0.032469

Gm7168	0.032355
Ankrd45	0.032275
Slc22a13	0.032265
Cdhr2	0.03217
Olfr572	0.032161
Trim72	0.032157
Coa6	0.03215
Slc9a4	0.031869
Hfe	0.031843
Slc16a12	0.031825
Hpse	0.031786
Irs2	0.031747
Orm3	0.031746
Tor2a	0.031744
Sytl2	0.031726
Kcnrg	0.031635
Slc23a3	0.031614
Pik3ap1	0.031562
Spag6	0.031502
Serpina1c	0.031482
Prss39	0.03145
Rhbdl2	0.031411
Olfr532	0.031364
Gltscr1l	0.031309
Bloc1s4	0.031291
C6	0.031049
Acot4	0.031015
Pdcd6ip	0.03101
Gle1	0.030895
4930402F0	0.030874
Emilin3	0.030849
Slc22a16	0.030762
Ccdc81	0.030744
Atxn2	0.030734
Fam89a	0.03071
Gimap9	0.030694
Srpk3	0.030693
Gramd2	0.030386
Abca17	0.030342
Olfr763	0.030319
Sec14l3	0.030295
Msmb	0.030294
1700024P1	0.030265
Fam162b	0.029991
Dapl1	0.029956
Shisa5	0.029855
Gabra4	0.029848

Eif2s3y	0.029687
Spag5	0.029623
Tmem255b	0.029585
Pla1a	0.029558
Sstr4	0.029492
Olfr39	0.029118
Zfp456	0.029055
Pamr1	0.028947
Cntnap4	0.028905
Tmem215	0.028873
Myl10	0.028855
Cd79a	0.028767
4932435O2	0.028681
Helz2	0.028671
Clcn7	0.028608
Tlcd2	0.0286
Cd80	0.02854
Timp4	0.028537
Ifnk	0.028513
4933412E2	0.028381
Clgn	0.028311
Crybb3	0.028283
Atp13a5	0.028205
Ceacam1	0.02819
Gper1	0.028153
Bdh1	0.027989
Tnni1	0.027921
Olfr462	0.0278
Cpa1	0.027771
Fam135b	0.027758
Pappa2	0.02774
Olfr907	0.027721
Luzp2	0.027649
Lpo	0.027625
C3	0.027547
Spaca1	0.027444
Fam83c	0.027438
Ppm1f	0.02728
Msh4	0.027277
Inpp5f	0.02726
Nkx6-3	0.027222
Pla2g16	0.027216
Csf3r	0.027128
Tssk5	0.02711
Kctd3	0.027067
Cd34	0.027042
Olfr320	0.027028

Rfc3	0.027024
P2ry4	0.026986
Sec22b	0.026917
Lhfpl5	0.026901
Gata1	0.026785
Smim6	0.026769
Nova2	0.026753
Hal	0.026732
Crhr2	0.0267
Mettl10	0.026677
Serpina3n	0.026624
Adcy1	0.026615
Olfr401	0.026569
Ccnb3	0.026537
Tspan8	0.026499
Ackr2	0.026457
Svopl	0.026431
Rhd	0.026426
Ppp1r1a	0.026398
Gm829	0.026189
3110007F1	0.026166
Nsd1	0.026157
Acot3	0.02612
Prl8a1	0.026041
Amz1	0.026032
Nlrp1c-ps	0.025975
Bex6	0.025939
Rundc3b	0.025835
Fgf6	0.025784
Olfr231	0.02576
Ptgfr	0.025722
Urad	0.025426
Ugt8a	0.025421
Vdr	0.025414
Aqp4	0.025298
Ces1e	0.025292
Cldn13	0.025285
Ccrl2	0.025279
Lcn9	0.025184
Ppfibp2	0.025178
Capn8	0.025135
Ap1s3	0.025133
Krtap4-2	0.025074
Rhox4a	0.025067
Paip1	0.024978
Zfp750	0.024952
Rnf103	0.024916

Ajap1	0.024891
Mcc	0.024832
Gm561	0.024829
Fancd2os	0.024668
Cd28	0.024657
Rnls	0.024622
Defb42	0.02462
Blk	0.024585
Ldb3	0.024551
Vmn1r26	0.024505
Dgcr8	0.024464
Fat1	0.024435
Trim30a	0.024406
Pgam5	0.024395
Edn3	0.024381
Col1a2	0.02437
Cyp27a1	0.02427
Dcpp3	0.024269
Scnn1b	0.024221
Cyp11b2	0.024138
Mina	0.024122
Zfp334	0.024109
Gpr119	0.024099
Grap2	0.023966
Cfhr1	0.023922
Rag2	0.023865
Smco2	0.023815
Armc3	0.023814
Hmgcll1	0.023782
Krt28	0.023772
Lrrtm4	0.023689
Npffr2	0.023644
Ceacam20	0.023618
Pbld1	0.023611
Lrrc17	0.023556
Zfp759	0.023483
Zim1	0.023438
Adh7	0.023405
Mettl24	0.023372
Rfx8	0.023317
Ltb4r1	0.023306
Fam175a	0.023153
Acadl	0.023141
Rin2	0.02309
Tspan17	0.023067
Gm11563	0.023042
Lgals3	0.022858

Tmem204	0.022802
Ddx47	0.022747
Opn3	0.022699
Hspb8	0.022639
Dok2	0.0226
1700012B0	0.02259
Fut4	0.02257
A630001G2	0.022479
Retn	0.022451
Cmtm2b	0.022404
Rfk	0.022337
Fbn1	0.022141
Calcb	0.022015
Ubash3a	0.021953
Plac8	0.02188
Marveld1	0.021796
Stfa2l1	0.021787
Gja8	0.021786
Melk	0.021704
Il17re	0.021618
Senp6	0.021617
Arsj	0.021567
Klk5	0.021526
Gapt	0.021481
Psma5	0.021415
Olfr1013	0.021248
Chd4	0.02123
Ddx17	0.021207
Mrgprh	0.021207
Gm6578	0.021194
Gorab	0.021194
Lyzl6	0.021181
Glipr2	0.02105
Slc35f4	0.020942
Wap	0.020925
Trim40	0.020757
Csrp3	0.020651
Gm4894	0.020589
Atp1b4	0.020546
Cpq	0.020531
Dpy19l2	0.020392
Nrbp1	0.020283
4930547C1	0.020276
E130308A1	0.020166
Cyp4f14	0.020045
Serpinb1b	0.020024
Snx20	0.01999

Ski	0.019953
Ildr1	0.019859
Chrm5	0.019839
Xlr4c	0.019801
Asgr2	0.019763
Eif4e1b	0.019749
Olfr311	0.019747
Uba7	0.019729
Cd177	0.01969
B4galnt3	0.019643
Cdx4	0.019458
Serpina1d	0.019417
Rprl1	0.019387
Galntl5	0.019371
Zswim6	0.019223
Drc1	0.019186
Hmgb3	0.018677
Rfwd2	0.018599
Fam159b	0.018444
Mapk10	0.01839
Herc3	0.018373
Olfr297	0.018352
Slc25a3	0.01833
Svep1	0.018261
Wdr91	0.018235
Kcnk6	0.018232
Ola1	0.018232
Ccdc37	0.018222
Dhrs7c	0.018155
B3gnt3	0.018153
Medag	0.01812
Padi6	0.01808
Cdh12	0.018078
Cpa4	0.017966
Ubl4b	0.017865
Usp9x	0.017859
Bpifb5	0.017822
Synrg	0.017808
Bcl2l14	0.017753
Tab3	0.017686
Kcnj11	0.017682
L3mbtl1	0.017663
Rassf10	0.017642
Gabrr1	0.017453
Ccl27a	0.017345
Olfr1413	0.017266
Slfn4	0.017083

Wtip	0.017036
Olfr531	0.016864
Krtap5-2	0.016857
Pde1c	0.016856
Parp12	0.016814
Cmklr1	0.016793
Nlrp9c	0.016741
Nop2	0.016694
Cog2	0.016615
Fcrlb	0.016517
Pde11a	0.016492
Wisp2	0.016458
Olfr881	0.016424
Ccdc86	0.016369
Ccdc73	0.016325
Dnajc11	0.016324
Btnl2	0.016305
Doxl2	0.016118
Pcdh12	0.016065
Oca2	0.016041
Pprc1	0.015903
Npsr1	0.015827
Tbc1d5	0.01579
Hsd17b2	0.015782
Tmem86b	0.015778
Heatr2	0.015629
Skint4	0.015625
Thsd7b	0.01555
Inpp5j	0.015504
BC049635	0.015429
Slc7a9	0.015402
Twf1	0.015295
Tmc3	0.015284
Fam45a	0.015266
Ins2	0.015182
Pax4	0.01506
Fbln7	0.015017
Aldh3a1	0.014984
Nrxn1	0.014978
Mapk8ip3	0.014958
Gm5820	0.01491
Ptx4	0.014774
Gpatch4	0.014703
Olfr707	0.014683
Efhb	0.014495
Cd59a	0.01431
Rrp12	0.014223

Clec3a	0.014053
Stk31	0.013983
Copg2	0.013934
Tpte	0.013858
Ido1	0.013847
Scn10a	0.013833
Jsrp1	0.013616
Gm525	0.013586
Olfr417	0.013536
4930571K2	0.013504
Fam169b	0.013487
Fcer2a	0.013476
Cers3	0.013439
Dyrk4	0.013339
Sepsecs	0.013319
Vwa5b1	0.013305
Psca	0.01319
Eif2s3x	0.013148
Slc9c1	0.013136
Vmn2r-ps5	0.013055
Igsf6	0.013013
Ceacam9	0.013003
Slc1a7	0.012934
Cck	0.012908
Cdh9	0.012763
Tmco4	0.012666
Tmem145	0.012604
Reep5	0.012567
Mall	0.012494
Agrn	0.01246
C5ar2	0.012244
Rft1	0.012211
Agap3	0.012116
AU015836	0.012101
Cd4	0.012066
Stra8	0.012066
Slc22a8	0.011929
Gpx2	0.011674
Pgc	0.011631
Abcb9	0.011444
D1Pas1	0.011418
Itgb7	0.011372
Gm5082	0.011227
Prr18	0.011193
Olfr1028	0.011191
Slc22a1	0.011175
Foxp1	0.011141

Arpc1b	0.010979
Gpr179	0.010897
Map3k7cl	0.010882
Rnf14	0.010842
Krt73	0.010834
Camsap3	0.010815
Pou3f1	0.010808
Rhox4f	0.010768
Syt15	0.010645
Serpina1e	0.01062
Zfp366	0.010581
Fam111a	0.01056
Serpinb6b	0.01043
Klk1	0.010425
Taf10	0.010389
Etnppl	0.010191
Commd7	0.010189
Usp54	0.010181
1700028P1	0.010176
Ikbke	0.010163
Pi4k2b	0.010118
1700021F0	0.009957
Csf2	0.00987
Lrrn4cl	0.009789
Ptprh	0.009763
Klf12	0.009673
Hdc	0.009552
P2ry14	0.009449
Ctcfl	0.009305
Sox13	0.009279
N28178	0.009264
Zfp429	0.009251
Nxnl2	0.009249
2200002J2	0.009089
Gabbr2	0.009069
Fcnb	0.009061
Dock3	0.008994
Olfr314	0.008986
Alppl2	0.008946
Rab12	0.008914
Cd44	0.008872
Olfr313	0.008596
Olfr1361	0.008593
Kcnc1	0.008432
Gm12695	0.008416
Oit3	0.008409
Sdcbp2	0.008369

Fbxo17	0.008274
Srsf11	0.008273
Olfr978	0.008115
Nme5	0.00809
Olfr684	0.007929
Prss28	0.007929
Sh2d1b2	0.007909
Cyp8b1	0.007889
Pik3cg	0.007872
Ralgps1	0.007855
Dagla	0.007852
Sel1l3	0.007829
Olfr984	0.007825
Sbpl	0.007656
Prdm2	0.007615
Serping1	0.00758
Serpina1b	0.007547
Zbtb4	0.007449
Smcp	0.007404
4921517D2	0.007233
H1foo	0.007198
Olfr130	0.007071
F12	0.006991
Vipr2	0.006966
AF366264	0.006962
Sostdc1	0.00696
Slc5a1	0.006867
Acan	0.006861
Creb3l3	0.006774
Lrrc49	0.006729
Ifna4	0.00649
Klrd1	0.00643
Akap2	0.006252
Srp19	0.00622
Dnase1	0.006215
Ccser2	0.006134
Dupd1	0.006118
Olfr525	0.006056
Olfr803	0.005959
Krtap9-3	0.005886
Olfr669	0.005751
Fbxl21	0.005544
Gc	0.005497
Mettl7a3	0.005474
Cr2	0.005471
Ppp1r2	0.005176
Ptprb	0.005175

Olfr91	0.00517
Sit1	0.005169
Prap1	0.00504
Ube2z	0.004949
Lzts1	0.004911
Dcstamp	0.004892
Clec2i	0.004859
Mapre1	0.004842
Irf5	0.004807
Olfr354	0.004787
Prss46	0.004757
Dnm3	0.004634
Epn2	0.004599
Snrpe	0.004578
Sytl3	0.00448
Hils1	0.004464
Pik3r6	0.00444
Gucy1a3	0.004221
Avpr2	0.004123
Olfr545	0.004098
Serpinb10	0.00409
Noc3l	0.00407
Mrps21	0.003978
Dennd4b	0.003933
Olfm3	0.003921
Tyro3	0.003837
Olfr654	0.003826
Rabgap1l	0.003751
Tmem63c	0.003722
Snrpd1	0.003717
Ppargc1a	0.003655
Wdr20rt	0.003557
Gnat2	0.003533
Olfr374	0.003442
Acer1	0.003369
Clca1	0.003193
Miox	0.003047
Crp	0.003028
Adam8	0.002853
Vnn3	0.002709
Tdh	0.002531
Dkk3	0.002491
Prpmp5	0.00247
A930003A1	0.002153
Olfr970	0.002063
Kng1	0.002061
Myo3b	0.002022

Prl8a2	0.001979
Hif1an	0.001914
Zfp551	0.001854
Olfr890	0.001759
Grem2	0.001638
Ppa2	0.001615
Pdzk1ip1	0.001541
Zdhhc23	0.001503
Alox15	0.001395
Ttc39c	0.001395
Tmem180	0.001263
Rhox4b	0.001216
F13a1	0.001199
Was	0.001105
Ang2	0.001086
Samd7	0.001064
Akr1c13	0.000786
Fbxw20	0.000786
Vmn2r15	0.000784
Vtcn1	0.000362
Olfr372	0.000332
Frmpd1	0.000274
Ly6f	0.000116
