# Supplementary material for: Mapping gene regulatory circuitry of Pax6 during neurogenesis
Source: Cell Discov. 2016 Feb 9;2:15045–. doi: 10.1038/celldisc.2015.45 (PMC4860964; doi:10.1038/celldisc.2015.45)
Supplement: Supplementary Table S2 [file celldisc201545-s9.pdf]

accession	geneName	logFC	Pax6.EnrichmentAverage
NM_00100	Slc35d2	1.619768	0.435793
NM_00100	Penk	1.887689	0.277932
NM_00100	A4galt	2.985056	0.267973
NM_00100	Gnptab	1.734885	0.344335
NM_00100	Slc28a1	1.112478	0.136807
NM_00100	Mreg	1.492124	0.165365
NM_00100	Aga	2.075045	0.289303
NM_00100	Mtus1	1.820235	0.320619
NM_00101	Trim6	2.152859	-0.053447
NM_00101	Fam169b	1.102763	0.013487
NM_00101	Gm13154	1.726736	-0.520271
NM_00101	Dppa4	3.139078	0.066924
NM_00103	Pecam1	3.549865	-0.040155
NM_00103	Cln6	1.242756	0.327528
NM_00103	Pls1	1.803125	0.294287
NM_00103	Slc45a4	1.43114	-0.434343
NM_00103	Gxylt1	1.343097	0.445797
NM_00103	Uap1l1	2.116555	0.251971
NM_00103	Mmp25	2.609134	0.119619
NM_00103	Kcnk6	1.750563	0.018232
NM_00103	Smagp	1.544491	0.242251
NM_00103	Cgn	1.254287	0.175399
NM_00103	Zdhhc12	1.017316	0.170773
NM_00103	Gm13051	2.727791	0.102276
NM_00103	Marveld2	1.765058	-0.047909
NM_00103	Elovl1	1.996591	0.238361
NM_00103	Ceacam1	1.891459	0.02819
NM_00103	Myo6	2.828546	0.166953
NM_00103	Slc30a2	1.180525	0.232459
NM_00104	Pitx2	1.0037	0.32846
NM_00104	Akap1	1.232929	0.336407
NM_00104	Sepp1	2.974505	0.185119
NM_00104	Rbpms	1.522258	0.283279
NM_00104	Mfge8	1.228178	0.187881
NM_00108	Myo1c	1.190632	0.316974
NM_00108	Cubn	6.292264	-0.131518
NM_00108	Lrp2	3.652542	0.142678
NM_00108	Lmtk2	1.242287	0.033728
NM_00108	Slc4a11	1.93459	0.334424
NM_00108	Lama5	1.755027	0.269387
NM_00108	Flnc	1.063859	0.381292
NM_00108	L1td1	3.536109	0.139309
NM_00108	Ermp1	1.257644	0.10892
NM_00108	Pigg	1.038612	0.406901
NM_00108	Alg6	1.031338	0.361978
NM_00108	Mdn1	1.136432	0.352317

NM_00108	Tmem248	1.336479	0.230974
NM_00108	Furin	1.815053	0.155902
NM_00108	Tex264	1.428719	0.275033
NM_00108	Sh3d19	1.395866	-0.255815
NM_00108	Spint2	1.952917	0.506578
NM_00108	Pdgfra	4.456926	0.3303
NM_00110	Dab2	4.260552	0.443117
NM_00110	Acp5	1.500098	0.4322
NM_00110	Zfp185	1.389579	0.107232
NM_00111	Cd151	1.159578	0.296676
NM_00111	Ccnb1ip1	2.330258	-0.102859
NM_00111	Ifitm1	1.729895	0.372555
NM_00111	Arhgef16	1.107827	0.182986
NM_00111	Lifr	2.213945	0.481249
NM_00111	Frrs1	2.631006	-0.004309
NM_00111	Lima1	1.783291	0.192768
NM_00111	Mageb16	2.589906	-0.170537
NM_00111	Slc16a10	1.576606	0.291523
NM_00112	Atp13a3	1.661095	0.165618
NM_00112	Psen2	1.097348	0.293873
NM_00113	Tcn2	2.249625	0.174027
NM_00113	Tdrd5	2.518295	0.1069
NM_00113	Slc39a8	3.35504	0.327738
NM_00113	Cd82	2.347223	0.098283
NM_00113	Enpp2	3.18535	0.079687
NM_00113	Tcirg1	1.369894	0.254144
NM_00115	Gaa	1.743901	0.411047
NM_00115	Esrrb	2.730302	0.423689
NM_00115	Slc20a1	1.485442	0.228942
NM_00115	Pvrl2	1.367891	0.394218
NM_00115	Nr5a2	1.548331	0.450503
NM_00116	Triml2	4.087244	-0.077065
NM_00116	Pqlc3	2.233328	0.502487
NM_00116	Rin3	1.096253	0.161476
NM_00116	Sgk1	2.802671	0.32666
NM_00116	Ptk2b	1.507651	0.323738
NM_00116	Abcc4	3.326826	0.439285
NM_00116	Tmem192	1.230412	0.35638
NM_00116	Camsap3	1.020633	0.010815
NM_00116	Erlin1	1.299658	0.189587
NM_00116	Rffl	1.736873	0.2061
NM_00116	Zfp809	1.370658	0.33344
NM_00116	Arhgap8	1.292539	0.373401
NM_00116	Eomes	3.07904	0.613875
NM_00116	Bcas3	1.070504	0.484434
NM_00116	Rhbdf2	1.877152	0.526263
NM_00116	Vrtn	2.088685	-0.001457

NM_00116	Enpp5	1.781709	0.261959
NM_00117	Mta3	1.360267	0.115238
NM_00117	Dhrs7b	1.234957	0.23475
NM_00742	Afp	6.661418	-0.236938
NM_00743	Alpl	2.031153	0.26552
NM_00746	Apoa4	3.149027	0.185808
NM_00746	Apoc1	2.169041	0.417157
NM_00747	Aqp8	4.935116	-0.049883
NM_00749	Ass1	2.245918	0.224152
NM_00751	Slc7a1	1.203748	0.21749
NM_00754	Prdm1	1.01551	0.400119
NM_00758	Anxa2	2.136547	0.148749
NM_00758	Calcr	1.765803	0.043222
NM_00760	Car4	4.05966	0.417293
NM_00764	Cd38	2.984488	0.117427
NM_00765	Cd63	2.293191	0.099626
NM_00765	Cd9	2.925452	0.144029
NM_00770	Cited1	1.866504	0.102578
NM_00772	Coch	1.517161	0.166681
NM_00779	Ctsb	1.943304	0.208407
NM_00780	Ctsh	5.780748	-0.009313
NM_00780	Cyba	1.885369	0.309531
NM_00783	Ddost	1.082862	0.402685
NM_00785	Degs1	2.236093	0.080125
NM_00787	Reep5	1.459139	0.012567
NM_00788	Dsg2	2.483671	0.444685
NM_00788	Slc26a2	2.557574	0.292863
NM_00793	Enpep	3.13252	-0.037695
NM_00794	Eps8	1.628782	0.04076
NM_00796	Mpzl2	2.166543	0.106817
NM_00797	F2rl1	2.665041	0.329329
NM_00797	F5	1.608172	0.197603
NM_00800	Fgf10	1.6316	0.367486
NM_00800	Fgf15	2.335175	0.349027
NM_00803	Folr1	3.849749	0.144538
NM_00808	B4galnt2	3.58566	0.57113
NM_00809	Gata4	2.663594	0.530502
NM_00809	Gba	1.496304	0.078256
NM_00810	Gcnt2	2.928323	0.241225
NM_00810	Gdf3	3.912874	-0.125593
NM_00811	Ggt1	2.071005	0.408214
NM_00812	Gjb3	2.503988	0.277599
NM_00812	Gclm	1.687731	0.07556
NM_00815	Gpc4	1.882716	0.114194
NM_00815	Cmklr1	1.492363	0.016793
NM_00816	Gpx3	3.218637	-0.021805
NM_00817	Grn	1.818403	0.465044

NM_00821	Has2	2.95751	0.636684
NM_00824	Ppap2a	1.2435	0.150853
NM_00826	Hnf4a	2.536592	0.040945
NM_00828	Hpn	1.603497	0.091683
NM_00829	Hsd17b2	2.86954	0.015782
NM_00834	Igfbp3	2.028826	0.346976
NM_00837	Impact	1.122615	0.070918
NM_00839	Itga6	1.586759	0.137112
NM_00840	Stt3a	1.355083	0.443447
NM_00841	Itm2b	1.549084	0.283685
NM_00842	Kcnj3	2.151595	0.493455
NM_00848	Lama1	2.404266	0.527202
NM_00848	Lamb1	3.223828	0.417707
NM_00848	Lamc2	1.323555	0.071028
NM_00851	Lrp5	1.514779	0.120552
NM_00855	Man2b2	2.123024	0.075451
NM_00858	Map3k5	1.440676	0.273421
NM_00858	Mep1b	1.000125	-0.448919
NM_00858	Mertk	1.50714	0.32349
NM_00860	Mme	1.999861	0.130018
NM_00860	Mmp14	1.415603	0.566823
NM_00861	Me1	2.684146	0.1101
NM_00861	Mov10	1.308087	0.143102
NM_00862	Gbp4	2.388645	-0.234778
NM_00863	Mt2	2.677272	0.596744
NM_00863	Map4	1.09536	0.261648
NM_00865	Mybl2	1.850578	0.229807
NM_00869	Nid2	3.546557	0.335973
NM_00872	Npc1	1.642849	0.263253
NM_00875	Ocln	2.174375	0.322675
NM_00880	Pde8a	2.021247	0.254628
NM_00890	Ppfibp2	2.309914	0.025178
NM_00892	Dnajc3	1.646727	0.210674
NM_00893	Prss12	2.804006	0.266284
NM_00899	Abcd4	1.503765	0.218871
NM_00905	Slc50a1	1.431872	0.357589
NM_00909	Rps6ka1	1.221048	0.302599
NM_00911	S100a10	1.956438	0.073485
NM_00914	Nptn	1.205244	0.362294
NM_00916	Sgpl1	1.492198	0.264059
NM_00918	St6galnac2	1.071555	0.353988
NM_00919	Slc12a2	1.661693	0.130913
NM_00919	Slc12a4	1.689626	0.462013
NM_00919	Slc16a1	2.352509	0.285972
NM_00923	Soat1	1.70564	0.063955
NM_00924	Sparc	2.964801	0.309165
NM_00925	Serpinb6a	1.806486	0.049891

NM_00925	Spink3	5.052468	-0.193553
NM_00926	Sptbn1	1.05523	0.481164
NM_00926	Spp1	3.3006	-0.008683
NM_00926	Sptlc1	1.065274	0.444444
NM_00928	Stat6	1.272673	0.437922
NM_00928	Stim1	1.351732	0.089236
NM_00930	Syngr2	1.493276	0.205894
NM_00942	Trh	5.978939	0.214272
NM_00948	Utf1	4.546978	0.067496
NM_00954	Trim25	2.278175	0.130338
NM_00955	Zfp42	3.482433	-0.024611
NM_00957	Zic3	2.932584	0.131846
NM_00960	Chrnb1	1.273352	0.216649
NM_00961	Adam19	2.567105	0.472709
NM_00967	Anxa7	1.529659	0.118842
NM_00967	Ap1m2	1.872008	0.318455
NM_00969	Apoa1	5.649684	0.206117
NM_00969	Apob	6.860253	0.436037
NM_00969	Apoe	3.40938	0.367027
NM_00971	Arsa	1.39646	0.190008
NM_00972	Atp1b1	2.813043	0.272378
NM_00972	Atp6v0c	1.444781	0.347352
NM_00973	Bcat2	1.43027	0.322645
NM_00975	Glb1	1.232503	0.336175
NM_00976	Klf5	1.598465	0.407908
NM_00978	Cacna2d1	1.579249	0.161777
NM_00984	Cd2ap	1.324223	0.501065
NM_00986	Cdh1	2.719154	0.239946
NM_00989	Ckmt1	1.179157	0.264004
NM_00990	Tpp1	1.596114	0.436381
NM_00997	Cst3	1.532651	-0.012553
NM_00998	Ctsc	5.897963	-0.004777
NM_00998	Ctsd	1.865537	0.285365
NM_00998	Ctsl	2.198881	0.134817
NM_01001	Cd55	3.69395	0.138538
NM_01002	Dazl	1.337697	-0.004862
NM_01006	Dnmt3b	3.411563	0.198392
NM_01007	Dpp4	5.001384	0.183222
NM_01009	Lefty1	3.616092	0.231668
NM_01011	Klk1b22	2.205438	0.092551
NM_01014	Ephb3	1.798417	0.396758
NM_01014	Ephb4	1.00279	0.593581
NM_01015	Erbb3	2.005761	0.284902
NM_01017	Fabp3	2.89044	0.409516
NM_01017	Fah	1.648661	0.14385
NM_01018	Fbln1	1.260376	0.380404
NM_01020	Fgf5	3.075536	0.327256

NM_01023	Fn1	1.352822	0.314304
NM_01025	Gata6	1.760944	0.414577
NM_01026	Gbp2	1.889188	-0.014324
NM_01028	Ggta1	2.795865	0.336444
NM_01028	Gja1	1.194596	0.637922
NM_01033	Emb	3.240861	0.538145
NM_01035	Gstm3	1.309532	-0.092435
NM_01041	Hbegf	1.452672	0.173502
NM_01042	Hexa	2.018818	0.151596
NM_01042	Hexb	1.619404	0.104056
NM_01044	Hmox1	1.539109	0.361538
NM_01047	Hs3st1	2.703649	0.335943
NM_01051	Igf2r	1.042013	0.123443
NM_01056	Il6st	1.187969	0.296062
NM_01057	Itga5	2.345333	0.524558
NM_01059	Jup	1.722734	0.31975
NM_01063	Klf9	2.181622	0.073741
NM_01068	Lamc1	2.951218	0.1484
NM_01068	Lamp1	1.853876	0.101907
NM_01068	Laptm5	1.738526	0.208279
NM_01070	Lef1	1.294842	0.507769
NM_01073	Muc13	3.132602	-0.161988
NM_01074	M6pr	1.212614	0.251308
NM_01081	Morc1	2.238796	0.181806
NM_01089	Neu1	2.213312	0.189873
NM_01090	Nfe2l2	1.018601	0.235998
NM_01091	Nid1	3.342717	0.934445
NM_01102	P2rx4	3.212468	0.323772
NM_01103	P4ha2	2.437656	0.172852
NM_01103	P4hb	1.461175	0.345852
NM_01107	Abcb1b	3.753988	0.111452
NM_01107	Abcb1a	2.049923	0.041473
NM_01110	Pla2g1b	3.147422	-0.006677
NM_01111	Plaur	2.771504	0.179732
NM_01111	Pld3	1.498541	0.253487
NM_01112	Plod1	1.701275	0.238509
NM_01115	Srgn	4.906845	-0.252632
NM_01117	Pros1	1.300868	0.24403
NM_01117	Lgmn	2.874306	0.252137
NM_01117	St14	2.386346	0.18663
NM_01119	Ptgfrn	1.24038	0.108399
NM_01120	Ptpn3	2.185264	0.405091
NM_01125	Rbp4	5.040647	0.05089
NM_01134	Sema3e	2.078364	0.159555
NM_01136	Itsn2	1.032978	0.145691
NM_01139	Slc23a1	4.01732	-0.030667
NM_01140	Slc2a3	1.806946	0.352474

NM_01140	Slc34a2	4.341105	0.044314
NM_01140	Slc7a7	4.679925	0.374922
NM_01141	Slit3	1.054761	0.381071
NM_01147	Sptlc2	1.30946	0.230668
NM_01152	Sdc4	1.630689	0.32568
NM_01154	Tcea3	1.035592	0.159133
NM_01156	Tdgf1	6.963501	0.307077
NM_01157	Tgfb1	1.061715	0.227793
NM_01157	Tgfbr3	2.235593	0.197416
NM_01159	Timp3	2.28309	0.096284
NM_01160	Tle4	1.138027	0.362003
NM_01167	Ucp2	1.502928	0.383067
NM_01172	Xlr3c	1.64071	-0.0585
NM_01180	Klf6	1.047311	0.596925
NM_01188	Scamp3	1.021632	0.473741
NM_01189	Spry2	2.216568	0.393873
NM_01189	Spry4	2.648848	0.263165
NM_01192	Abcg2	2.76151	-0.104324
NM_01193	Clcn7	1.654906	0.028608
NM_01193	Gnpda1	1.788013	0.084822
NM_01197	Slc27a2	3.488306	0.250116
NM_01203	Slc9a3r1	1.729898	0.41161
NM_01205	Fkbp9	1.887589	0.088548
NM_01346	Ankrd1	1.296251	-0.1299
NM_01347	Anxa4	1.657858	0.220429
NM_01347	Anxa6	1.477562	0.134978
NM_01348	C2	2.189158	0.115784
NM_01358	Lrpap1	2.92585	0.200366
NM_01360	Mt1	2.212178	0.214899
NM_01361	Nodal	2.776976	0.293345
NM_01363	Pou5f1	5.17744	0.237815
NM_01363	Sypl	1.166738	0.313305
NM_01365	Sema4b	1.10094	0.378697
NM_01366	Sema4d	1.080606	0.126632
NM_01369	Tek	2.298076	-0.132478
NM_01369	Ttr	6.147354	-0.312074
NM_01372	Podxl	2.707569	0.42306
NM_01378	Ptdss2	1.022795	0.251043
NM_01379	Naglu	1.094516	0.196016
NM_01380	Abcc2	3.860683	-0.257176
NM_01382	Ly75	1.95683	0.150276
NM_01384	Ror1	1.759755	0.168217
NM_01573	Galnt3	2.534384	0.154141
NM_01579	Icosl	1.608987	0.269999
NM_01579	Fbxo15	3.809278	-0.124991
NM_01580	Crim1	1.777293	0.109277
NM_01580	Pnpla6	1.255925	0.577097

NM_01669	Gpc3	1.561926	0.334369
NM_01672	Iqgap1	2.021318	0.067217
NM_01679	Vamp8	1.46749	0.56312
NM_01686	Htatip2	1.289743	0.176171
NM_01688	Cldn7	2.772127	0.202024
NM_01688	B3gnt2	2.204377	0.221085
NM_01689	Cd164	1.475199	0.328145
NM_01690	Sec61a1	1.213558	0.209548
NM_01690	Spint1	2.532004	0.09226
NM_01691	Slc40a1	2.394217	0.232784
NM_01692	Atp6v0a1	2.032422	0.217473
NM_01697	Slc7a8	3.590524	0.155838
NM_01873	Gbp3	1.018139	-0.120688
NM_01874	Sema6a	1.989921	0.535768
NM_01877	Crlf3	1.300688	0.535942
NM_01879	Atp6ap1	1.384027	0.092395
NM_01882	Slc23a2	1.503431	0.386815
NM_01886	Slc1a4	1.48408	0.378905
NM_01939	Tyro3	1.17846	0.003837
NM_01944	Ramp2	2.093982	0.435938
NM_01944	Dnmt3l	4.34953	0.132211
NM_01952	Gas6	2.476916	0.12854
NM_01956	Htra1	2.948891	0.214147
NM_01964	Clptm1	1.256111	0.409775
NM_01975	Plp2	1.783963	0.56248
NM_01980	Ggcx	1.658801	0.310848
NM_01992	Crtap	2.407358	0.095205
NM_02000	0610031J0	2.120866	0.426734
NM_02002	Lpar2	1.013697	0.36465
NM_02027	Scamp5	1.277071	0.340132
NM_02027	Tnfrsf10b	1.887427	-0.015815
NM_02057	As3mt	2.742578	-0.043449
NM_02127	Cxcl10	1.706127	-0.101871
NM_02129	Slc7a9	2.909121	0.015402
NM_02139	Hyou1	1.235947	0.290195
NM_02139	Slc43a3	1.398546	-0.131164
NM_02145	Pga5	4.828396	0.046447
NM_02148	Tdh	4.382616	0.002531
NM_02152	Chst12	1.497694	0.361622
NM_02179	Tmem8	1.833742	0.166174
NM_02189	Trp53inp1	1.073513	0.324897
NM_02230	B4galt1	2.473043	0.186546
NM_02232	Ctsz	2.527384	0.064961
NM_02241	Myh9	1.522234	0.383394
NM_02288	Slc29a1	2.426885	0.311928
NM_02288	Lpin3	1.285368	-0.067726
NM_02299	Pmepa1	1.431182	0.326104

NM_02305	Sigirr	2.065255	-0.011678
NM_02306	Ifi30	2.593246	0.367193
NM_02313	Renbp	1.363784	0.207415
NM_02314	Tor3a	1.620952	0.235646
NM_02314	Ranbp17	1.61857	0.145028
NM_02347	Tinagl1	2.147703	0.178848
NM_02353	Pla2g12b	3.279996	0.287842
NM_02355	Slc44a4	2.504412	0.156515
NM_02358	Epha1	3.182123	0.095975
NM_02358	Ptplb	1.693206	0.226073
NM_02359	Arid5b	1.555971	0.933248
NM_02362	Plbd2	1.352761	0.220379
NM_02363	Rab27a	1.947717	0.185886
NM_02371	Spns1	1.422781	0.229777
NM_02375	Tfcp2l1	2.482985	0.099042
NM_02379	Etv5	3.890691	0.381482
NM_02386	Ryr2	1.434644	0.214531
NM_02387	Set	1.500494	0.586362
NM_02416	Fkbp11	1.185721	0.395484
NM_02428	Rrbp1	1.756066	0.139878
NM_02444	Cyp4f16	1.148716	0.071951
NM_02527	Dppa5a	4.253892	-0.010518
NM_02544	Ssr2	1.25442	0.097005
NM_02545	Fam134b	2.979353	-0.058516
NM_02546	Sec11c	1.374706	0.202152
NM_02557	2610528J1	2.866191	0.227613
NM_02565	Ms4a4d	1.44201	-0.149998
NM_02570	Dcbld1	1.786981	0.291389
NM_02579	Fuca2	1.720394	0.089812
NM_02580	Plbd1	2.60413	0.037317
NM_02581	Mfsd1	1.782514	0.201927
NM_02583	Plin3	1.289042	0.582481
NM_02584	Kdelr2	1.345722	0.375187
NM_02597	Naaa	2.522679	0.126471
NM_02603	Cmtm6	1.571124	0.340376
NM_02605	Cers4	1.086385	-0.035237
NM_02616	Plxdc2	1.324011	0.304218
NM_02617	Abhd5	1.226535	0.200701
NM_02621	Agpat2	1.987103	0.126629
NM_02638	Shisa5	1.774916	0.029855
NM_02640	Tmem39a	1.014014	0.279464
NM_02648	Ooep	1.46325	-0.002343
NM_02659	Cgnl1	1.347917	0.158475
NM_02663	Gtsf1l	1.269827	0.139246
NM_02664	Agpat4	1.081702	0.413353
NM_02666	Mfsd10	1.337064	0.280165
NM_02666	Tmbim6	1.500839	0.307095

NM_02695	Vstm5	1.149648	0.237386
NM_02713	Sec24d	1.462332	0.18591
NM_02715	Tmbim1	2.796219	0.048072
NM_02728	Manba	2.482811	0.135998
NM_02733	Mettl7a1	1.423406	0.390326
NM_02738	Tet1	1.073272	-0.135749
NM_02740	Aldh1l1	1.347702	0.224978
NM_02743	Atp6ap2	1.329464	0.147216
NM_02746	Fam213a	2.456874	0.056875
NM_02749	Tmem144	2.877691	0.184407
NM_02751	Pvr	2.376559	0.220257
NM_02753	Kdsr	2.021803	0.322868
NM_02787	Arhgef3	2.139368	-0.149667
NM_02787	Slc46a3	1.385895	0.419926
NM_02787	Dram1	3.476405	0.142768
NM_02789	Rhpn2	1.462127	0.18129
NM_02800	Ppapdc1b	1.027714	0.128896
NM_02801	Tom1l1	1.084077	0.526367
NM_02802	Gatsl3	2.183081	0.195692
NM_02803	Rbpms2	1.844971	0.331722
NM_02803	Tdrd12	3.730876	-0.093076
NM_02805	Cyb5r1	1.931721	0.568669
NM_02806	Slc35f2	2.10567	0.227951
NM_02806	Slc39a4	3.715409	0.181586
NM_02812	Adpgk	1.174156	0.577117
NM_02839	Phykpl	1.367402	0.200903
NM_02847	Rassf6	1.800295	-0.04542
NM_02860	Tex19.1	1.326624	0.042118
NM_02861	Dppa2	2.952879	-0.000481
NM_02865	Mfsd12	1.911754	0.411907
NM_02870	Herc3	1.760329	0.018373
NM_02873	Esyt2	1.059011	0.320202
NM_02874	Npl	2.661926	0.134723
NM_02877	Cyp2s1	2.433903	0.099591
NM_02878	Slc35f5	1.333185	0.238435
NM_02900	Elovl7	2.440031	0.336504
NM_02902	Scpep1	1.535144	0.207636
NM_02909	Pik3cb	1.295069	0.161475
NM_02935	Pcdh1	1.966469	0.300335
NM_02936	Tm4sf5	1.5158	0.08565
NM_02936	Gns	1.551063	0.188656
NM_02944	4930447C0	3.878648	0.10221
NM_02950	Pcgf5	1.888371	-0.252295
NM_02962	Pcolce2	2.344671	0.300238
NM_02977	Unc5b	2.656831	0.398522
NM_02978	Cers2	1.055179	0.222434
NM_02985	Tmco4	1.066957	0.012666

NM_03072	9930013L2	3.413608	0.298358
NM_03117	Dscam	2.126645	0.175905
NM_03118	Klb	2.788217	-0.157967
NM_03119	Tfec	3.425262	-0.29005
NM_03137	Tex15	1.196967	-0.175415
NM_03138	Usp26	2.805658	-0.055073
NM_03140	Crispld1	1.084137	-0.218873
NM_03184	Dpp7	2.102961	0.252476
NM_03187	Ghdc	1.308751	0.095304
NM_03188	Tnk1	1.836037	0.071009
NM_03331	Slco2a1	4.325092	0.213088
NM_03360	Amn	2.587237	0.254632
NM_05307	Car7	2.870028	0.177136
NM_05310	Glrx	1.811314	0.275214
NM_05317	Mcoln1	1.394155	0.139622
NM_05326	Hsd17b11	3.468786	0.299282
NM_05408	Slc19a2	1.40528	0.39941
NM_05409	Pip4k2c	1.233571	0.101158
NM_08055	Itpr3	1.988641	0.362038
NM_13088	Cyp4f13	1.57267	0.27746
NM_13320	Pilrb1	1.599369	-0.043023
NM_13322	Sgk3	2.856244	0.289661
NM_13335	Prss8	2.24496	0.237233
NM_13366	Lad1	1.029461	0.28078
NM_13370	Tmem97	1.188025	0.415822
NM_13388	Smpdl3b	1.368188	0.046857
NM_13389	Lrrc8c	1.182048	0.176454
NM_13393	Rpn1	1.259599	0.167706
NM_13400	Enpp3	3.317585	0.290874
NM_13408	Flnb	1.841666	0.282927
NM_13409	Kdelr3	2.735133	0.29356
NM_13410	Txndc11	1.096875	-0.022184
NM_13415	Actn1	1.017929	0.299945
NM_13443	Il17rd	2.404312	0.190709
NM_13858	Fam3c	1.558724	-0.039854
NM_13859	Gldc	1.689408	0.242052
NM_13860	Pim2	2.259893	0.724545
NM_13867	Edem1	1.545279	0.070262
NM_13895	Rassf3	1.465898	0.323393
NM_13906	Rbm47	2.739917	-0.005624
NM_13919	Plac8	1.620318	0.02188
NM_14452	Tmem214	1.110827	0.205443
NM_14479	Tmem63a	1.424501	0.341977
NM_14481	Cbx7	2.186476	0.176424
NM_14483	Tmem106a	2.24967	-0.176304
NM_14484	Otx2	4.69554	0.515365
NM_14490	Atp1a1	1.117627	0.360088

NM_14490	Slc35a3	1.038605	0.268426
NM_14491	Tmem184a	1.672527	0.173416
NM_14491	Tmem150a	2.433086	0.27951
NM_14495	1700019D0	3.099605	0.163558
NM_14536	Alg1	1.317368	0.318877
NM_14539	Slc44a3	3.4372	0.157947
NM_14541	Hkdc1	3.083366	0.098944
NM_14543	Llgl2	1.312274	0.294526
NM_14549	Me2	1.233882	0.287046
NM_14553	Edem2	1.016834	0.272198
NM_14553	Tmem189	1.27098	0.0881
NM_14555	Ldlrap1	1.277296	0.362229
NM_14583	Lin28a	1.701701	0.408844
NM_14592	Pttg1ip	1.926117	0.269339
NM_14593	Sumf1	1.812609	0.102981
NM_14595	Cth	1.085315	0.073751
NM_14598	Tmem82	1.433586	0.135745
NM_14601	Flcn	1.275813	0.405803
NM_14606	Soat2	3.778388	0.182843
NM_14610	Habp2	3.666181	-0.202533
NM_14611	Tor4a	1.158778	0.098786
NM_14894	Serpinb6c	2.498869	-0.189432
NM_15222	Stx3	2.739093	0.070867
NM_15312	Atf7ip2	2.531215	0.157174
NM_15312	Nat10	1.104452	0.365346
NM_15313	Slc36a1	1.598813	0.106477
NM_15341	Trpm6	1.751239	-0.242896
NM_15350	Nckap1l	1.468972	-0.185404
NM_15351	Pilra	2.628765	-0.065644
NM_15351	Kcng3	2.531617	0.141277
NM_15359	Ugt2b34	3.533541	-0.300903
NM_17211	Entpd6	1.260297	0.122776
NM_17213	Adap2	1.033069	0.163149
NM_17229	Sulf1	3.096317	0.277832
NM_17230	Jade1	1.95171	0.279651
NM_17238	Tmem125	1.601735	0.240779
NM_17239	Akr1b10	1.203911	0.286379
NM_17241	Ostm1	1.169654	0.186713
NM_17246	Zdhhc9	1.5619	0.370085
NM_17246	Clic6	3.924487	0.162666
NM_17247	Tmc7	1.151249	0.178275
NM_17250	Dse	1.337288	0.204352
NM_17258	Serinc5	1.852255	0.246322
NM_17262	Clic5	1.587662	0.116002
NM_17265	Slco4c1	3.276907	0.07001
NM_17267	4932438A1	1.021641	0.059304
NM_17268	Ddx58	1.028052	0.191421

NM_17269	Galnt12	1.176647	0.072856
NM_17269	Inadl	2.202549	0.172921
NM_17271	Sel1l3	2.236563	0.007829
NM_17275	Slc38a7	1.643869	0.35417
NM_17276	Gramd1b	3.376641	0.468462
NM_17277	Slc17a5	1.548221	0.248266
NM_17338	Slc43a2	2.032735	0.539817
NM_17344	Gcnt1	2.54587	0.09768
NM_17387	Mgat4a	1.407587	-0.344618
NM_17496	Gimap9	1.037456	0.030694
NM_17499	Fmr1nb	3.684451	-0.061898
NM_17517	Plekhf2	1.238651	0.09929
NM_17525	Sec24a	1.159702	0.161405
NM_17533	Nt5dc3	1.044951	0.292522
NM_17541	Tspan9	1.692236	0.075601
NM_17565	Cnpy1	1.589489	0.03768
NM_17567	Slc35f6	1.684781	0.21017
NM_17683	Dnajc22	3.164257	0.36243
NM_17707	Relt	1.513594	0.262688
NM_17709	Lefty2	4.027388	0.099051
NM_17715	Lrig3	1.399104	0.597681
NM_17730	Lrrn4	1.792091	0.292892
NM_17732	Mia2	4.770081	-0.144521
NM_17739	Myo1d	1.50107	0.142756
NM_17774	Triml1	3.139245	-0.169605
NM_17776	Slc35e1	1.272261	0.130217
NM_17777	Esyt3	1.203872	0.129365
NM_17778	Dock5	1.704342	0.272966
NM_17781	Zfp459	2.774302	-0.039543
NM_17785	Dennd2c	2.703137	0.099707
NM_17807	Pm20d1	2.397152	0.180488
NM_17814	Prkaa2	1.599383	0.111509
NM_17837	Slc9a8	1.171594	0.605456
NM_17838	Ano9	2.344419	-0.081163
NM_17840	Arap2	1.611633	0.15543
NM_17859	Tagln2	1.199889	0.498653
NM_17860	Vkorc1	1.557741	0.211465
NM_17869	Prrg4	2.822719	0.144923
NM_17871	Tmem30b	2.681871	0.053191
NM_17874	Egflam	1.547785	0.372362
NM_17877	Nceh1	1.133594	0.236398
NM_18058	Reep4	1.151314	0.260839
NM_18154	Nostrin	3.731076	0.049077
NM_18182	Tmc4	1.94362	0.077319
NM_18184	Optn	2.01885	0.068579
NM_18303	Plekhm1	1.059246	0.186673
NM_18309	Xlr4c	1.499254	0.019801

NM_19426	Tbx20	1.547049	0.764088
NM_19829	Tex2	1.580559	0.282006
NM_19860	Them6	1.507738	0.299905
NM_19888	B4galnt3	2.906317	0.019643
NM_19901	Stard8	2.631668	0.039621
NM_20139	Sall4	1.300262	0.311952
NM_20720	Lpcat4	1.568596	0.073627
NM_21244	Kdelc2	1.257771	0.202103
NR_00363	Rps4l	1.161241	0.240221
NR_00396	Atp10d	2.735197	0.277789
NR_02826	Rian	1.727388	0.228682
NR_02832	AU015836	1.541882	0.012101
NR_02855	Snord53	1.161698	-0.442473
NM_00100	Baz2b	-1.197871	0.373243
NM_00100	Megf10	-1.761922	0.155649
NM_00100	Brsk1	-1.14169	0.315909
NM_00100	Senp7	-1.185717	0.354756
NM_00100	Scube3	-2.141555	0.204868
NM_00100	Dbx1	-4.77127	0.877794
NM_00100	Ypel4	-1.237674	0.611584
NM_00100	Zfp663	-2.130348	0.33177
NM_00100	Syne2	-2.271091	0.299177
NM_00100	Fndc3c1	-1.891615	0.202819
NM_00100	Brsk2	-2.64208	0.249679
NM_00101	Mdk	-1.152067	0.253926
NM_00101	Fut10	-1.238202	0.340593
NM_00101	Sesn1	-1.168487	0.370742
NM_00102	Kbtbd7	-1.131382	0.195673
NM_00102	Pdp2	-1.110242	0.425168
NM_00102	Lrp3	-1.579542	0.183001
NM_00102	Rbfox3	-1.019095	0.321626
NM_00102	Trp53i11	-1.990459	0.258209
NM_00102	Tfap2b	-2.790897	0.518586
NM_00102	Lonrf2	-2.296358	-0.082159
NM_00102	Zswim5	-2.741069	0.211247
NM_00103	Cblb	-1.114464	0.124948
NM_00103	Hk3	-1.564786	0.179535
NM_00103	Gm266	-1.377189	0.18929
NM_00103	Agap2	-1.147938	0.151787
NM_00103	5031439G	-1.025557	0.059961
NM_00103	Gm1673	-1.027379	0.244987
NM_00103	Atxn7l3b	-1.381799	0.191288
NM_00103	Klhl32	-1.219324	0.209778
NM_00103	Zc3h12b	-1.218149	0.131321
NM_00103	Dach1	-4.635485	0.231499
NM_00103	Npr3	-3.50061	0.441392
NM_00103	Gria2	-2.411807	0.257747

NM_00103	Lin7a	-3.462206	0.217322
NM_00103	Slc22a15	-1.235865	0.161414
NM_00103	Kif21b	-2.15186	0.541647
NM_00103	Evi5l	-1.09188	0.200305
NM_00104	Sstr2	-2.250592	0.276603
NM_00104	Cadps	-3.299935	0.182756
NM_00104	Rorb	-3.024662	0.235633
NM_00104	Map6	-1.584323	0.271722
NM_00104	Ccnjl	-2.306622	0.295139
NM_00104	Jak2	-1.02932	0.14498
NM_00108	Prdm13	-1.667909	0.843637
NM_00108	Zc3h7b	-1.105107	0.232929
NM_00108	Pard3b	-2.128497	0.150771
NM_00108	Clip3	-2.09303	0.316438
NM_00108	Phf21b	-1.844367	0.312846
NM_00108	Lrch2	-1.767018	0.106436
NM_00108	Mn1	-1.474987	0.075089
NM_00108	Tbc1d4	-1.00354	0.253015
NM_00108	Mfhas1	-1.324942	0.206544
NM_00108	Zkscan2	-1.48566	0.220905
NM_00108	Pcdh9	-1.636045	0.471041
NM_00108	Myo16	-1.59934	0.102599
NM_00108	Unc13b	-1.064617	0.137362
NM_00108	Nckap5	-3.039449	-0.060934
NM_00108	St7	-1.745217	0.12953
NM_00108	Syndig1	-1.929743	0.323145
NM_00109	Tox2	-1.29841	0.295499
NM_00109	Pik3c2b	-1.713025	0.322076
NM_00109	Pnmal2	-1.213869	0.302794
NM_00110	Zfp703	-1.053593	0.284546
NM_00110	Ccdc8	-1.032429	0.359357
NM_00110	Kif21a	-2.018945	0.557217
NM_00110	Ctnna2	-2.248705	0.23967
NM_00111	Echdc1	-1.317194	0.268874
NM_00111	Trim9	-2.982349	0.191225
NM_00111	Ebf4	-1.226928	0.164668
NM_00111	Pclo	-1.489968	0.091986
NM_00111	Cdc25b	-2.596884	0.316243
NM_00111	Arhgef10l	-1.317884	0.373187
NM_00111	Ncam1	-2.278884	0.285597
NM_00111	Ebf3	-2.819831	0.303177
NM_00111	Glo1	-1.508014	0.694573
NM_00111	Stxbp1	-2.917619	0.111296
NM_00111	Plcl1	-1.395239	0.375761
NM_00112	Pde7a	-1.077321	0.257563
NM_00112	Fyn	-1.247509	0.355557
NM_00113	Sos2	-1.153427	0.488161

NM_00113	Cntfr	-2.38932	0.561366
NM_00113	Ednrb	-4.674507	0.535508
NM_00114	Atat1	-1.088345	0.484953
NM_00114	Acss3	-1.524758	0.389782
NM_00114	Pogk	-1.672725	0.191954
NM_00114	Mpped2	-1.305694	0.371431
NM_00115	Llgl1	-1.104577	0.565473
NM_00115	H2afy	-1.583863	0.27394
NM_00115	Cacna1c	-1.228989	0.221606
NM_00115	Efcc1	-1.502129	0.390938
NM_00116	Grm2	-2.151403	0.583681
NM_00116	9330159F1	-1.868888	-0.072437
NM_00116	Zc3h12c	-2.627649	0.238833
NM_00116	Elavl4	-3.729263	0.261376
NM_00116	Pou2f2	-1.734147	-0.036853
NM_00116	Satb1	-1.756313	0.213149
NM_00116	Skor1	-1.080501	0.353507
NM_00116	C530008M	-2.006642	0.167062
NM_00116	Smad5	-1.293924	0.219848
NM_00116	Add3	-1.044841	0.236008
NM_00116	Tcf3	-1.034911	0.46283
NM_00116	Fbxl16	-2.422662	0.202931
NM_00116	Pdzrn4	-2.65944	0.585833
NM_00116	Robo3	-3.265969	0.446197
NM_00116	Cspg5	-2.691754	0.202228
NM_00116	Rfx3	-1.3186	-0.031157
NM_00116	Chn1	-1.304972	0.123932
NM_00116	Zfp882	-1.345009	0.54814
NM_00116	Zfp647	-1.08364	0.157445
NM_00116	Commd6	-1.300876	0.118163
NM_00117	Rcbtb2	-1.413628	0.421582
NM_00117	BC005764	-2.46583	0.314012
NM_00117	Rbms3	-1.252775	0.303627
NM_00746	Apba2	-3.974159	0.137981
NM_00748	Arg1	-1.917869	0.388314
NM_00748	Rhob	-1.685845	0.498164
NM_00748	Arnt2	-1.62352	0.21625
NM_00749	Astn1	-1.607939	0.093356
NM_00749	Zfhx3	-1.593763	0.428728
NM_00750	Neurod4	-4.061145	0.312684
NM_00756	Bpgm	-1.036805	0.411073
NM_00756	Zfp36l1	-2.256476	0.722009
NM_00758	Calb2	-1.458681	0.238509
NM_00760	Car3	-2.867105	0.184574
NM_00763	Ccnd1	-1.780075	0.623613
NM_00766	Cdh8	-3.589575	0.754125
NM_00768	Cfl2	-1.24538	0.400353

NM_00769	Chrm4	-1.724309	0.453844
NM_00772	Ackr3	-1.589732	0.425768
NM_00773	Col12a1	-2.476323	0.416464
NM_00775	Crabp2	-4.641553	0.673126
NM_00776	Crmp1	-1.917593	0.365795
NM_00776	Crp	-1.583686	0.003028
NM_00778	Csn3	-1.419617	-0.05912
NM_00778	Ncan	-1.678164	0.138008
NM_00783	Dcc	-4.602895	0.391946
NM_00786	Dll1	-2.17421	0.729626
NM_00786	Dll3	-2.257091	0.55586
NM_00787	Doc2b	-1.526759	0.255721
NM_00789	E2f5	-1.279416	0.191422
NM_00789	Ebf1	-1.642934	0.734728
NM_00790	S1pr1	-2.295093	0.452041
NM_00790	Efna2	-1.317271	0.375917
NM_00793	Epha5	-1.68528	0.166388
NM_00804	Frat1	-1.331203	0.238815
NM_00805	Fzd8	-1.296075	0.314731
NM_00808	Gap43	-3.251306	0.305094
NM_00808	Gas1	-2.535527	0.146131
NM_00816	Grik5	-1.231332	0.296501
NM_00822	Hdac2	-1.064407	0.349834
NM_00824	Foxd1	-1.663383	0.453662
NM_00825	Hmgb3	-1.570487	0.018677
NM_00826	Hoxa4	-3.041155	0.387237
NM_00826	Hoxb1	-1.436018	0.580479
NM_00826	Hoxb5	-4.475878	0.432198
NM_00826	Hoxb6	-4.761613	0.39459
NM_00827	Hoxb9	-3.230223	0.525926
NM_00830	Sdc2	-1.347899	0.14145
NM_00837	Lrig1	-1.025276	0.347547
NM_00839	Irf2	-1.720344	0.332443
NM_00839	Irx3	-3.123572	0.470949
NM_00844	Kif1b	-1.327523	0.355313
NM_00844	Kif5c	-2.299278	0.095585
NM_00847	L1cam	-1.379448	0.080803
NM_00849	Lfng	-1.930928	0.323044
NM_00849	Lhx1	-1.985163	0.796209
NM_00850	Mycl	-1.728104	0.145646
NM_00851	Lrrn1	-2.038753	0.183904
NM_00853	Marcks	-2.74741	0.746857
NM_00855	Ascl1	-3.192875	0.241721
NM_00859	Mfng	-1.262086	0.356617
NM_00862	Msi1	-3.000304	0.180989
NM_00863	Map2	-3.240324	0.233617
NM_00863	Map1b	-1.998654	0.244668

NM_00866	Myt1	-3.486428	0.045189
NM_00869	Nefm	-3.149521	0.269999
NM_00872	Ctnnd2	-1.9456	0.329125
NM_00873	Nptx1	-2.13181	0.274958
NM_00874	Nsg2	-2.54446	0.223345
NM_00874	Ntrk3	-1.231374	0.361409
NM_00875	Nxn	-1.854455	0.201263
NM_00878	Pax3	-4.409861	0.614336
NM_00880	Pde6d	-1.131164	0.233198
NM_00888	Plxna3	-1.228609	0.212139
NM_00889	Pou3f2	-3.617375	0.26329
NM_00890	Pou3f4	-3.653987	0.255326
NM_00891	Ppp3cc	-2.166794	0.150347
NM_00892	Prkar2a	-1.708439	0.351658
NM_00893	Prox1	-2.858439	0.455534
NM_00898	Igdcc3	-3.351676	0.284101
NM_00901	Raet1a	-1.792958	0.3406
NM_00902	Rasa3	-1.353432	0.413427
NM_00913	Scg3	-3.512394	0.12863
NM_00913	Stmn3	-1.889488	-0.024114
NM_00914	Sfrp2	-1.273783	0.505094
NM_00916	Scg5	-1.969057	0.192492
NM_00916	Shd	-2.046365	0.331048
NM_00918	St8sia2	-1.731925	0.234644
NM_00921	Sst	-2.952149	0.355534
NM_00923	Sox11	-2.531311	0.439673
NM_00923	Sox3	-1.38917	0.095091
NM_00923	Sox4	-1.907093	0.284109
NM_00929	Stra8	-3.464061	0.012066
NM_00930	Syt1	-2.158264	0.299072
NM_00930	Syt2	-1.047384	0.160175
NM_00936	Tgfb1i1	-1.952919	0.183654
NM_00939	Tll1	-1.411807	0.487289
NM_00940	Top2b	-1.844177	0.299004
NM_00945	Ube2e1	-1.127417	-0.006602
NM_00946	Dpysl3	-2.925661	0.539451
NM_00952	Wnt7a	-1.132913	0.357154
NM_00955	Zfand5	-1.164016	0.163593
NM_00955	Zfp37	-1.104067	0.213822
NM_00955	Zfp46	-1.084803	0.398949
NM_00957	Zik1	-1.535612	-0.021216
NM_00959	Abl1	-1.281669	0.285232
NM_00960	Chrnb2	-1.065095	0.132106
NM_00961	Adam11	-1.138399	0.132345
NM_00962	Adcy8	-1.92779	0.414893
NM_00967	Ank3	-1.345752	0.395844
NM_00967	Anp32a	-1.461043	0.487886

NM_00968	Apbb1	-1.04489	0.093285
NM_00971	Neurog2	-1.759158	0.514072
NM_00973	Bche	-1.715106	0.273755
NM_00977	Serping1	-1.364104	0.00758
NM_00978	Cacna2d3	-1.836678	0.317108
NM_00982	Ccnd2	-3.080796	0.375802
NM_00987	Cdkn1c	-2.66252	0.097344
NM_00988	Celsr1	-1.1851	0.201664
NM_00992	Cnih2	-1.030176	0.071023
NM_00998	Cux1	-1.363684	0.139848
NM_01001	Cyp46a1	-1.055136	0.275476
NM_01007	Drd2	-1.643211	0.391567
NM_01009	Sparcl1	-1.223131	0.114651
NM_01010	Edil3	-1.777271	0.245116
NM_01012	Pou6f1	-1.081717	0.213045
NM_01014	Epha3	-4.683295	0.538778
NM_01014	Ephb2	-2.124916	0.208923
NM_01015	Nr2f1	-5.364183	0.225942
NM_01015	Erbb4	-2.643486	0.562496
NM_01015	Khdrbs3	-1.307693	0.303612
NM_01016	Eya2	-1.002311	0.136385
NM_01016	Eya4	-2.938001	0.442476
NM_01019	Lgr5	-2.396112	0.396744
NM_01020	Fgf13	-1.186521	0.296792
NM_01026	Gdap1	-1.900293	0.159402
NM_01027	Gdf11	-1.988497	0.549852
NM_01027	Gfra1	-2.348157	0.523506
NM_01031	Gnb5	-1.137343	0.346815
NM_01034	Aes	-1.722166	0.066294
NM_01040	Hap1	-1.393159	0.504972
NM_01043	Hivep2	-1.96847	0.31006
NM_01044	Hoxa1	-4.34055	0.403766
NM_01045	Hoxa2	-3.307801	0.605472
NM_01045	Hoxa3	-4.372097	0.539727
NM_01045	Hoxa6	-2.809859	0.483587
NM_01045	Hoxb3	-3.95523	0.352614
NM_01046	Hoxb7	-2.091687	0.480297
NM_01046	Hoxb8	-4.808876	0.554075
NM_01046	Hoxc6	-2.55904	0.464762
NM_01046	Hoxd3	-4.212602	0.253886
NM_01046	Hoxd4	-5.26546	0.615836
NM_01048	Elavl3	-2.683869	0.133241
NM_01057	Itga4	-1.21042	0.194229
NM_01072	Lipg	-1.880406	0.307717
NM_01080	Marcksl1	-1.494856	0.199999
NM_01082	Mpdz	-1.496425	0.126268
NM_01083	Msn	-1.102707	0.58836

NM_01083	Msx3	-2.59873	0.531388
NM_01088	Ndn	-1.952584	0.402871
NM_01089	Neurod1	-3.276404	0.813319
NM_01089	Neurog1	-3.996569	0.386036
NM_01091	Nefl	-2.727662	0.434939
NM_01091	Nhlh1	-3.442136	0.385757
NM_01093	Nppc	-1.380881	0.101084
NM_01094	Nsg1	-2.455567	0.325048
NM_01102	Hspa4l	-2.250817	0.198203
NM_01103	Pak1	-1.231344	-0.017332
NM_01103	Pax2	-1.714482	0.271522
NM_01103	Pax7	-2.213231	0.361971
NM_01115	Ppp1r17	-2.002249	-0.043067
NM_01115	Prkar2b	-2.660035	0.312094
NM_01116	Mapk11	-1.071999	0.732869
NM_01116	Mapk8ip1	-1.725971	0.258099
NM_01122	Rab33a	-1.466927	0.658286
NM_01124	Rarb	-5.270493	0.528213
NM_01125	Rbp1	-3.934229	0.284892
NM_01126	Rgs16	-1.972002	0.372384
NM_01130	Dhrs3	-3.439091	0.889011
NM_01130	Uimc1	-1.062306	0.350731
NM_01133	Cxcl15	-2.73436	-0.190068
NM_01137	St8sia1	-1.479826	0.286452
NM_01138	Six5	-1.384516	0.120389
NM_01138	Ski	-1.022461	0.019953
NM_01139	Slc1a2	-1.984307	-0.244325
NM_01142	Snap25	-1.731063	0.355736
NM_01144	Sox9	-2.271378	0.372636
NM_01171	Wiz	-1.076667	0.343389
NM_01172	Dctn6	-1.2833	0.273192
NM_01174	Mkrn3	-1.102746	0.269507
NM_01175	Zfp41	-1.775421	0.496941
NM_01177	Ikzf2	-1.16377	0.153833
NM_01178	Akt3	-2.076632	-0.111092
NM_01178	Apc2	-1.491771	0.600961
NM_01179	Car14	-1.503118	0.827063
NM_01181	Gadd45g	-2.678818	0.703909
NM_01186	Papss1	-1.116268	0.22749
NM_01186	Pde10a	-1.508174	0.38665
NM_01187	Dazap2	-1.457578	0.267986
NM_01188	3-Sep	-2.309542	0.288899
NM_01193	Esrrg	-1.522248	0.231523
NM_01194	Map2k6	-1.384699	0.263637
NM_01199	Dpysl4	-2.855159	0.471626
NM_01199	Abcd2	-3.350724	0.498064
NM_01201	Gprin1	-1.793988	0.283787

NM_01345	Add2	-1.117095	-0.14219
NM_01346	Aldh1a1	-1.259103	-0.108868
NM_01349	Cpe	-2.041423	0.364897
NM_01349	Crabp1	-4.18084	0.371131
NM_01355	Hoxc4	-5.222119	0.541909
NM_01356	Kcnh2	-1.58209	0.287338
NM_01362	Pax6	-3.987382	0.520701
NM_01366	Sema5b	-2.313631	0.639554
NM_01368	Tcf4	-1.576373	0.182796
NM_01378	Npas3	-2.889286	0.664578
NM_01380	Plk3	-3.381749	0.124998
NM_01381	Baz1a	-1.248226	0.231225
NM_01388	Ulk2	-1.876332	0.270815
NM_01388	Scmh1	-1.740279	0.267397
NM_01388	Hdgfrp3	-2.694676	0.169108
NM_01391	Zbtb18	-2.389779	0.442525
NM_01391	Usp25	-1.268284	0.162888
NM_01573	Chrna4	-1.450492	0.072046
NM_01574	Slit1	-1.259746	0.240915
NM_01668	Vezf1	-1.189683	0.2345
NM_01670	Nes	-2.705288	0.391159
NM_01674	Nell2	-3.622551	0.371451
NM_01675	Rundc3a	-1.715311	0.256949
NM_01676	Pbx3	-1.981984	0.100587
NM_01676	Smad3	-1.478579	0.494929
NM_01680	Mtx2	-1.248973	0.163019
NM_01688	Insm1	-2.416234	0.277703
NM_01739	Celsr2	-3.097295	0.509748
NM_01746	Nedd9	-2.948984	0.722694
NM_01874	Igfbpl1	-2.431218	0.078074
NM_01877	Skap2	-2.124524	0.405365
NM_01880	Syt11	-2.308824	0.446614
NM_01880	Hs3st3b1	-1.227469	0.584835
NM_01882	Sh2b2	-1.289861	0.172298
NM_01885	Akr1e1	-1.838028	0.226815
NM_01888	Pdzrn3	-3.042435	0.121665
NM_01941	Pfn2	-2.453496	0.333311
NM_01944	Fmn2	-2.512073	0.558993
NM_01947	Hes6	-1.198518	0.428167
NM_01955	Ptbp2	-1.439981	0.096628
NM_01964	Stmn1	-1.327462	0.320182
NM_01967	Plcb1	-1.606574	0.3289
NM_01974	Zfp113	-1.336025	0.390272
NM_01975	Tagln3	-3.649011	0.600567
NM_01983	Gkap1	-1.118895	0.354446
NM_01994	Mnx1	-1.186382	0.403129
NM_01994	Mgst1	-1.321414	0.172748

NM_01994	Ube2l6	-1.611833	0.370695
NM_01997	Dclk1	-1.365384	0.105821
NM_01998	Habp4	-1.951443	0.280864
NM_01998	Sh3bgrl	-1.921639	0.281947
NM_02003	Raet1d	-1.727735	0.13367
NM_02025	Zbtb33	-1.354365	0.920008
NM_02025	Hhip	-1.926319	0.34358
NM_02049	Glra1	-3.17942	0.428138
NM_02127	Ckb	-1.985618	0.347645
NM_02127	Wnt1	-2.656924	0.458173
NM_02133	Cdon	-1.51968	-0.183119
NM_02142	Fam181b	-1.48021	0.323144
NM_02145	Fzd3	-2.814747	0.371979
NM_02149	Smpd3	-1.461084	0.525102
NM_02149	Ap3b2	-1.917184	0.740748
NM_02150	Sh3rf1	-1.204257	0.38787
NM_02150	Moxd1	-2.141211	0.433428
NM_02156	Bhlhe22	-1.334666	0.19041
NM_02156	Midn	-1.25131	0.308228
NM_02156	Pcbp4	-1.448428	0.447024
NM_02192	Mapk8ip2	-1.51908	0.665803
NM_02231	Clstn2	-1.118083	0.05011
NM_02304	Dpysl5	-2.490832	0.321451
NM_02311	Cacnb2	-1.637451	0.153422
NM_02312	Palm	-1.114079	0.194588
NM_02321	Anp32e	-1.182129	0.358312
NM_02327	Tubb3	-2.664686	0.470903
NM_02332	Plekho1	-1.561973	0.254342
NM_02343	Tmem132e	-2.726346	0.311691
NM_02371	Tubb2b	-2.709879	1.068407
NM_02388	Ralgps2	-1.132792	0.451124
NM_02418	Yaf2	-1.372776	0.101896
NM_02419	Chmp1b	-1.366873	0.180887
NM_02425	Hsdl2	-1.434719	0.36989
NM_02427	Ssbp2	-1.733016	0.294706
NM_02539	Pop4	-1.358347	0.243552
NM_02540	Arl4d	-1.56716	0.245189
NM_02544	Taf13	-1.407505	0.305293
NM_02545	Camk2n1	-1.870507	0.040417
NM_02547	Isoc1	-1.63971	0.035146
NM_02559	Ppdpf	-1.377133	0.288538
NM_02561	Eid1	-1.768694	0.073425
NM_02563	Gdpd1	-1.206483	0.315619
NM_02567	Sike1	-1.094876	0.281873
NM_02569	Sorcs3	-1.076212	0.281116
NM_02571	Rbm4b	-1.025658	0.278848
NM_02582	Eif4e3	-1.387388	0.371615

NM_02590	Dek	-1.011921	0.348375
NM_02590	Polr3k	-1.184213	0.570673
NM_02594	Rps6ka6	-1.429935	-0.260745
NM_02598	Nrarp	-2.692571	0.313263
NM_02605	Fam220a	-1.054013	0.230608
NM_02612	Hmgn3	-1.912204	0.485628
NM_02613	Soga3	-2.725258	0.506946
NM_02628	Mxra7	-1.715989	0.255819
NM_02631	Ift74	-1.753402	0.418529
NM_02636	Caap1	-1.409494	0.425875
NM_02638	Rgs8	-1.776732	0.148397
NM_02638	Pnrc2	-1.114687	0.30535
NM_02639	Nmral1	-1.236697	0.468909
NM_02640	Sncaip	-2.560297	0.159264
NM_02641	Rgs10	-1.323215	0.236604
NM_02648	Tppp3	-2.882025	0.550165
NM_02650	Snrpg	-1.071498	0.36146
NM_02651	2810417H1	-1.421774	0.250297
NM_02651	Tmem178	-1.260899	0.110669
NM_02655	Fam92a	-1.357847	0.164456
NM_02656	Apool	-1.08179	0.532584
NM_02661	Ccdc34	-1.407559	0.233398
NM_02667	Gstm7	-1.218441	0.269202
NM_02673	Ppp1r14a	-1.352031	0.586317
NM_02678	Mthfd2l	-1.803669	0.265087
NM_02681	Upk1a	-1.313254	0.218804
NM_02688	Srrm4	-2.620452	0.433636
NM_02689	Eif1b	-1.057648	-0.011871
NM_02690	Zfp580	-1.552824	0.393484
NM_02693	1810011O1	-1.591409	0.386233
NM_02711	Klhdc2	-1.076919	0.279074
NM_02714	Enho	-1.120501	0.450632
NM_02729	Rab28	-1.890927	0.191912
NM_02740	Fndc5	-3.149613	0.410263
NM_02750	Prdm16	-1.246215	0.170407
NM_02762	Pgm2l1	-1.31851	0.173714
NM_02764	Phf6	-1.70938	0.186098
NM_02768	Rfx4	-3.480081	-0.030652
NM_02788	Smug1	-3.2416	0.398885
NM_02804	Cnn3	-1.210773	0.299299
NM_02822	Wdr17	-1.104065	-0.068762
NM_02826	Fgfbp3	-3.137486	0.129109
NM_02830	Tdrkh	-2.514352	0.592301
NM_02832	Zfp618	-1.756677	0.100742
NM_02834	Ttc39c	-1.370873	0.001395
NM_02845	Gulp1	-2.051799	0.22587
NM_02845	Tm7sf2	-1.256633	0.434876

NM_02849	Rhobtb3	-1.800315	0.389534
NM_02877	Nuak2	-1.171739	0.31602
NM_02881	Vit	-3.098084	0.582793
NM_02889	4931414P1	-1.022491	0.37766
NM_02894	Clvs1	-2.565201	0.286209
NM_02903	Spsb1	-1.476814	0.241948
NM_02905	Tbc1d30	-1.620441	0.356727
NM_02935	Mzt2	-1.024974	0.301309
NM_02938	Nudt16	-1.052094	0.413214
NM_02952	Gpsm2	-1.483068	0.234615
NM_02953	Lamp5	-1.446101	0.247677
NM_02961	Prss23	-1.868509	0.260333
NM_02966	Mfsd2a	-3.235738	0.401851
NM_02984	Tstd3	-1.108431	0.312082
NM_02985	Bcl7a	-1.242908	0.283483
NM_02986	Zkscan1	-1.270151	0.296455
NM_02987	Hnrnpa0	-1.3151	0.152441
NM_02991	Kcnk10	-1.410564	0.236491
NM_02993	Fam115a	-1.767095	0.550689
NM_02994	Prdm8	-4.105503	0.867375
NM_02997	Trim35	-1.059313	0.318888
NM_03008	Hist3h2ba	-1.105629	0.524313
NM_03020	Tspyl4	-1.640627	0.358798
NM_03059	Nbea	-1.32924	0.052748
NM_03350	Vangl2	-1.538433	0.21091
NM_03359	Hist2h4	-1.094515	0.455567
NM_03360	Peli2	-1.688502	0.293066
NM_03360	Dach2	-1.453341	0.276125
NM_05297	Adarb2	-1.443738	0.233991
NM_05299	Spock2	-1.980691	0.438877
NM_05300	Olig3	-3.525692	0.461763
NM_05307	Nrep	-3.926906	0.212802
NM_05309	Setbp1	-1.461135	0.35656
NM_05311	Pard6g	-1.402169	0.273128
NM_05312	Pcdhb2	-1.553728	-0.074295
NM_05312	Pcdhb3	-1.657311	-0.104973
NM_05313	Pcdhb6	-1.28173	-0.174713
NM_05313	Pcdhb11	-2.017898	-0.160127
NM_05313	Pcdhb12	-1.765614	-0.300709
NM_05314	Pcdhb16	-2.370065	-0.055946
NM_05314	Pcdhb17	-2.346495	-0.037086
NM_05314	Pcdhb18	-2.031648	-0.126227
NM_05314	Pcdhb19	-2.426139	0.040337
NM_05314	Pcdhb20	-1.964376	-0.123762
NM_05314	Pcdhb21	-2.024067	-0.206537
NM_05314	Pcdhb22	-2.045096	-0.1372
NM_05404	Rcor2	-1.420967	0.186638

NM_05405	Gpr98	-3.467581	0.501784
NM_05409	Necab2	-1.276802	0.251337
NM_08028	Elmo2	-1.56772	0.516387
NM_08044	Srgap3	-3.142554	0.624799
NM_08046	Zfp358	-1.465535	0.290142
NM_08056	Rnf144a	-1.490308	0.242288
NM_08064	Bhlhe23	-1.007715	0.089739
NM_08072	Rem2	-1.489717	0.379968
NM_13089	Scrt1	-1.478486	0.377372
NM_13322	Ripply3	-2.198348	0.366892
NM_13323	Glcci1	-1.045296	0.230856
NM_13323	Crb1	-1.008605	0.256724
NM_13336	Acaca	-1.242617	-0.010318
NM_13350	Ntng2	-3.645734	0.535198
NM_13371	Metrn	-3.056139	0.292138
NM_13376	Atp6v0e2	-1.007735	0.296904
NM_13385	Olfml3	-2.285243	0.635834
NM_13402	6330403K0	-1.005676	0.087619
NM_13403	Hoxb2	-4.869477	0.562702
NM_13408	Dnajc9	-1.012004	0.175511
NM_13866	Nlgn1	-1.857613	0.628489
NM_13894	Zfp286	-1.175204	0.257209
NM_13922	Rhbdl3	-2.988072	0.104263
NM_14455	Trib2	-1.476263	0.435254
NM_14493	Usp3	-1.327502	0.283309
NM_14512	Chrna3	-4.753816	0.313123
NM_14513	Spsb4	-3.449985	0.814583
NM_14514	Aif1l	-1.015049	0.231026
NM_14514	Frmd4b	-1.941546	0.25714
NM_14515	Emilin2	-1.951983	0.26436
NM_14539	Casd1	-1.245481	0.218941
NM_14541	Fam173a	-1.468778	0.68208
NM_14542	Ube2d1	-1.268521	0.330713
NM_14545	Zswim6	-1.439975	0.019223
NM_14545	Zfp503	-2.081727	0.748776
NM_14551	Mark1	-1.411318	0.135123
NM_14551	Plekhb2	-1.434282	0.068909
NM_14558	Sbk1	-1.69948	0.354824
NM_14561	Lrrc49	-1.559973	0.006729
NM_14571	Tox	-1.461033	0.319982
NM_14600	Lss	-1.313934	0.304662
NM_14604	Cdca7l	-1.570516	0.335212
NM_14605	Ccar2	-1.113107	0.198202
NM_14612	Fam110a	-1.700372	0.259594
NM_14613	Vav3	-2.133326	-0.031462
NM_14618	Kctd15	-1.138075	0.281792
NM_14625	Wdr78	-1.319853	0.18527

NM_14893	Slc6a5	-1.405798	1.175398
NM_14894	Chrnb4	-2.128093	0.517345
NM_15280	Hpse	-1.508898	0.031786
NM_15291	Dner	-2.058766	0.342094
NM_15305	Mapre2	-1.604344	0.249632
NM_15306	Ehd2	-1.352378	-0.020132
NM_15311	Cadm4	-1.149815	0.423991
NM_15313	Unc5a	-4.366007	0.293363
NM_15317	Ago4	-1.185704	0.07955
NM_15339	Wdr19	-1.033638	0.23995
NM_15340	Ago1	-1.044895	0.145053
NM_15340	Csrnp3	-1.823009	0.333809
NM_15341	Gpsm1	-1.059669	0.287257
NM_15345	Rtn1	-1.70357	0.173469
NM_15357	Katnal1	-1.371895	0.477517
NM_15360	Ttc26	-1.406377	0.307397
NM_17229	Tshz3	-2.347984	0.066724
NM_17238	Zfp536	-3.559588	0.359338
NM_17239	Ndnf	-2.787361	0.44139
NM_17243	Celf3	-2.277019	0.282279
NM_17244	Dtx4	-3.807926	0.160339
NM_17247	Dennd2a	-1.109978	0.489798
NM_17250	Boc	-3.047902	0.504258
NM_17254	Nrxn3	-2.818096	-0.115003
NM_17257	Slc25a21	-1.906855	0.686736
NM_17269	N28178	-1.43848	0.009264
NM_17280	Sdk2	-2.420902	0.32076
NM_17281	Lrp12	-1.642779	0.248472
NM_17283	Ccnj	-1.250685	0.154059
NM_17291	Tox3	-2.286062	0.384016
NM_17295	Gphn	-1.424868	0.229352
NM_17298	Nkain3	-1.393599	0.507375
NM_17339	Zbtb5	-1.157744	0.153125
NM_17340	Rgs12	-1.119934	0.316267
NM_17341	Rab8b	-1.359991	0.332524
NM_17373	Galnt18	-2.547099	0.273509
NM_17378	Ubtd2	-1.176406	0.343247
NM_17378	Npr2	-2.398324	0.107297
NM_17386	St18	-2.079461	0.218461
NM_17484	Crybg3	-1.242107	-0.121001
NM_17512	1110051M	-1.473848	0.094573
NM_17513	Rnf122	-1.344267	0.517931
NM_17516	Zdhhc1	-1.310933	0.211109
NM_17516	Stox2	-2.583482	-0.103399
NM_17523	Faxc	-1.868872	0.129764
NM_17525	Heg1	-2.655944	0.377113
NM_17526	Myh10	-1.143765	0.20773

NM_17527	Cntln	-1.181106	0.096568
NM_17527	Fhod3	-1.0051	0.247675
NM_17536	Lyrm2	-1.451999	0.397419
NM_17536	Mex3b	-2.699221	0.3293
NM_17544	Rassf2	-1.594572	0.303199
NM_17547	Cyp26b1	-4.510688	0.521113
NM_17548	Grik4	-1.661712	0.172511
NM_17551	Fam171b	-1.869791	0.141479
NM_17552	E330009J0	-1.115931	0.26485
NM_17564	Bai3	-2.12587	0.434316
NM_17565	Hist1h4d	-1.318711	0.392931
NM_17575	Plxna4	-1.044012	-0.012403
NM_17684	Chrna5	-3.350073	0.333778
NM_17696	Fsd1l	-2.096542	0.215731
NM_17697	Podxl2	-1.362746	0.202113
NM_17698	Simc1	-1.041066	0.19744
NM_17704	Auts2	-1.308059	0.459325
NM_17712	Cntn2	-2.511596	0.174183
NM_17716	Ppm1e	-2.186583	0.257898
NM_17719	Tcp11l1	-1.347659	0.296454
NM_17725	Cstf3	-1.459985	0.536571
NM_17729	Itgb8	-3.475397	0.839651
NM_17732	Agtr1a	-2.348196	0.309788
NM_17735	Vash1	-2.187101	0.329041
NM_17737	Rnf150	-1.01814	0.227296
NM_17743	Adamts20	-1.315316	0.542499
NM_17762	Fam167a	-1.855748	0.154091
NM_17768	Vgll4	-2.201528	0.16697
NM_17772	Ranbp6	-1.159345	0.074765
NM_17772	Lsm14b	-1.040281	0.14417
NM_17775	Sox21	-1.775354	0.426914
NM_17776	Lhfpl4	-1.279638	0.066582
NM_17778	Prex1	-1.003926	0.323698
NM_17789	B4galnt4	-1.030029	0.341119
NM_17811	Dixdc1	-1.131857	0.239708
NM_17820	Hist1h2bm	-1.168383	0.385796
NM_17825	Arhgap33	-1.629708	0.119668
NM_17839	3110035E1	-1.793492	0.400417
NM_17841	Zfp334	-1.985913	0.024109
NM_17860	Reep1	-1.705148	-0.003989
NM_17865	Ank2	-1.502202	0.221261
NM_17867	Fstl5	-1.826221	0.274369
NM_17875	E130309F1	-1.591005	0.491066
NM_17877	Nhlh2	-3.079464	0.333468
NM_17887	Trim36	-1.565296	0.264486
NM_17892	Afap1l1	-2.310576	0.366472
NM_18140	Wdr47	-1.227703	0.25516

NM_18142	Fn3krp	-1.490156	0.438945
NM_18301	Zfp184	-1.282173	0.341276
NM_18317	Zfp811	-2.855453	0.474691
NM_18322	Fat4	-2.969205	0.316204
NM_18330	Zfyve9	-1.037304	-0.049864
NM_18331	Ctxn1	-1.499756	0.332989
NM_18335	Pbx1	-2.778449	0.418507
NM_19426	Onecut2	-1.056963	0.495034
NM_19810	Morn4	-1.342696	0.424555
NM_19811	Dagla	-1.822163	0.007852
NM_19861	Igsf21	-1.899737	0.265931
NM_19886	Slitrk5	-1.015591	0.231781
NM_19888	Zbtb12	-1.583682	0.03734
NM_19920	Fam171a2	-1.011304	0.330496
NM_19944	Zhx2	-1.202079	0.564432
NM_19946	Nexn	-1.81367	0.189131
NM_20137	Prmt8	-4.159076	0.45531
NM_20695	Ltbp1	-1.581267	-0.227769
NM_20700	H2afy2	-2.206576	0.297837
NM_20717	Tes	-1.049797	0.065935
NM_20720	BC068157	-1.575571	0.387737
NM_21243	Foxp2	-3.258753	0.254021
NM_21361	5-Sep	-1.609199	0.055884
NR_00356	Gpr137b-p	-1.68849	0.279277
NR_01554	2810055G	-5.32014	0.584377
NR_02735	Vash2	-2.201289	0.194204
NR_02782	Chd3os	-1.487889	0.141423
NR_02789	Hoxd3os1	-3.544558	0.41219
NR_02797	Gm5607	-1.693726	0.383337
NR_03069	B330016D	-1.585601	0.322926
